# Supplementary material for: Quantum spin-engineering in on-surface molecular ferrimagnets
Source: Nat Commun. 2025 Jun 5;16:5208. doi: 10.1038/s41467-025-60409-w (PMC12137798; doi:10.1038/s41467-025-60409-w)
Supplement: Supplementary file 1 — Supplementary Information [file 41467_2025_60409_MOESM1_ESM.pdf]

Supplementary Information

Quantum Spin-Engineering in On-Surface Molecular Ferrimagnets

Wantong Huang[1], Máté Stark[1], Paul Greule[1], Kwan Ho Au-Yeung[1], Daria Sostina[1,2], José Reina Gálvez[3,4], Christoph Sürgers[1], Wolfgang Wernsdorfer[1,2], Christoph Wolf[3,4], Philip Willke[1,5*]

[1] Physikalisches Institut, Karlsruhe Institute of Technology (KIT), Karlsruhe, Germany
[2] Institute for Quantum Materials and Technologies, Karlsruhe, Germany
[3] Center for Quantum Nanoscience, Institute for Basic Science (IBS), Seoul, Republic of Korea.
[4] Ewha Womans University, Seoul, Republic of Korea.
[5] Center for Integrated Quantum Science and Technology (IQST), Karlsruhe Institute of Technology, Karlsruhe, Germany

* corresponding author: philip.willke@kit.edu,

Table of Contents

1. Building FePc-Fe(C_6H_6) complexes 2
2. Lattice site analysis 3
3. Spin Hamiltonian and IETS dI/dV spectra simulations 4
4. Magnetic sensing 10
5. DFT calculations 12
6. ESR and fitting 18
7. Rabi rate as a function of V_{RF} 19
8. Conductance-dependent spin lifetime 22
9. Spin transport simulations and enhanced spin lifetime 24
10. ESR transitions in a Heisenberg two-spin system with the tip field detuning effect 28
11. Electronic properties of FePc-Fe(C_6H_6) complex on MgO 34

1. Building FePc-Fe(C_6H_6) complexes

The FePc-Fe(C_6H_6) complexes were created using the atom manipulation scheme sketched in Supplementary Fig. 1a. First, the tip is positioned above one ligand of FePc. The tip is then moved $\sim 600\ \mathrm{pm}$ closer to the molecule and a bias voltage of $850\ \mathrm{mV}$ is applied. Next, the tip is retracted from the surface with the FePc sticking to its apex. To drop off the molecule, we used two methods: 1) Perform Z-Spectroscopy starting with setpoints of 10pA and -10mV, gradually moving the tip $250-330\ \mathrm{pm}$ towards the Fe atom. 2) Scan fast across the Fe atom at closer distance ($V_{\mathrm{set}} = -40\ \mathrm{mV}$, $I_{\mathrm{set}} = 1\ \mathrm{nA}$ and scan speed of $300\ \mathrm{nm/s}$). Supplementary Fig. 1b and 1c show the topographic images before and after building an FePc-Fe(C_6H_6) complex, respectively. Supplementary Fig. 1d and 1e show additional cross sections across the complex, in which the protrusion at the position of the Fe atom becomes greatly visible. In Fig. 1a in the main text, we also showed multiple dimers of complexes. These were built using similar atom manipulation schemes. For two coupled FePc-Fe(C_6H_6) complexes, we build two complexes separately first and then move one towards the other. To move the complex, the tip is positioned between two ligands ($V_{\mathrm{set}} = -100\ \mathrm{mV}$, $I_{\mathrm{set}} = 20\ \mathrm{pA}$), next moved $200-400\ \mathrm{pm}$ closer to the surface and finally a bias voltage of $500-750\ \mathrm{mV}$ was applied.

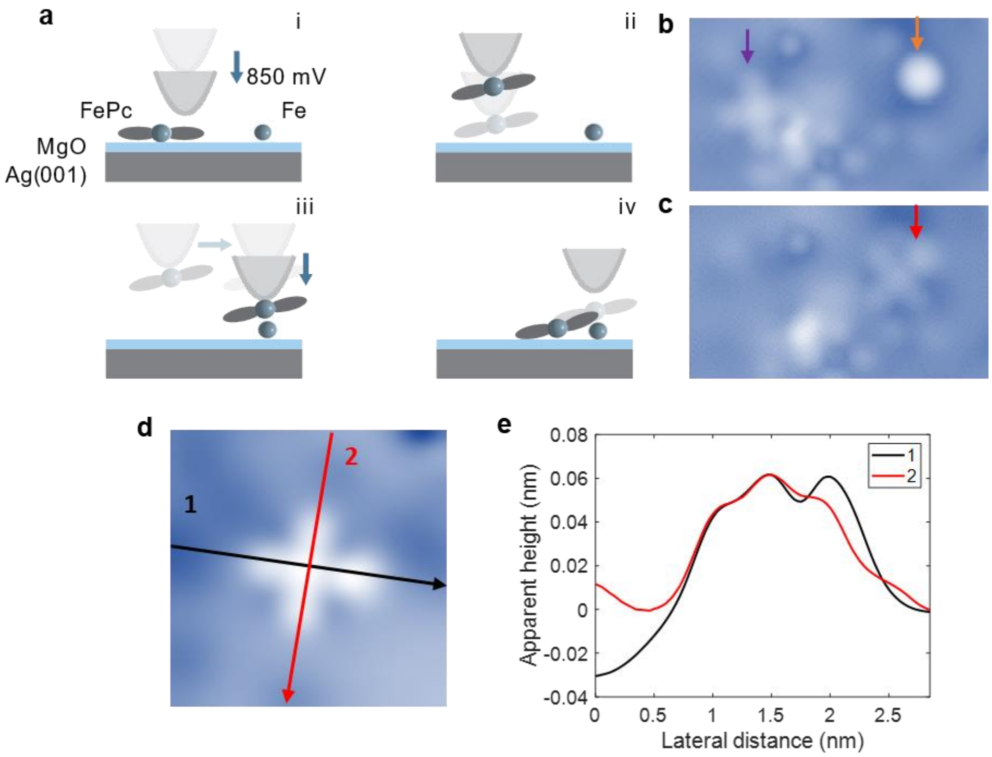

Supplementary Fig. 1. a, Schematic illustrating the manipulation procedures involved in building FePc-Fe(C_6H_6) complexes. **b,** Topographic image consisting of one Fe atom (orange arrow) and one FePc molecule (purple arrow) on 2ML MgO ($V = 100$ mV, $I = 20$ pA). **c,** Topographic image afterwards with a newly formed FePc-Fe(C_6H_6) complex (red arrow). **d,** STM topography image ($V = 100$ mV, $I = 20$ pA) and **e,** the corresponding line profiles comparing the ligands of the FePc-Fe(C_6H_6) complex with (black) and without (red) the Fe adatom underneath the benzene ring.

2. Lattice site analysis

The FePc-Fe(C_6H_6) complexes adsorbed on 2ML MgO/Ag(001) tend to adopt specific site configurations. The precise positioning of the Fe atoms and FePc molecules with respect to the underlying MgO lattice is determined by atomic resolution images of the bare MgO area. In addition, the position of nearby individual Fe atoms, which are adsorbed atop a MgO oxygen lattice site[1], is taken as a reference of the lattice. In Supplementary Fig. 2a, the oxygen lattice is indicated by a white grid overlaid on the FePc-Fe(C_6H_6) complex structure. The Fe atom (marked in red) is situated at a distance of 0.64 nm (2×1 oxygen lattice sites) from the center of FePc. Consequently, both Fe and FePc are still adsorbed atop oxygen sites (Supplementary Fig. 2b), as it is the case for the isolated atom and molecule[1,2]. These specific positions are used as the basis for our DFT calculations.

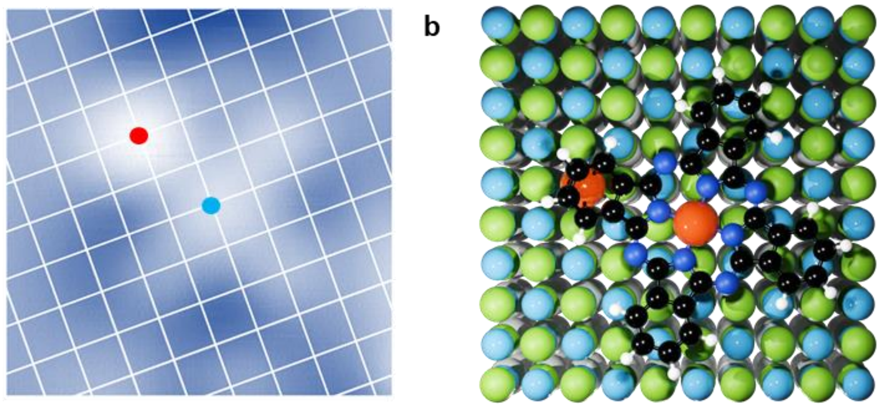

Supplementary Fig. 2. FePc-Fe(C_6H_6) complexes binding site. a, Topographic image of an FePc-Fe(C_6H_6) complex with MgO lattice overlaid (2nm × 2 nm, setpoint: V = 100 mV, I = 20 pA). The positions of Fe atom and molecule center are labeled by red and blue dots, respectively. The position of oxygen atoms in the MgO lattice is indicated by a white grid. **b,** Top view image shows the stable configuration of the FePc-Fe(C_6H_6) complex calculated by DFT.

3. Spin Hamiltonian and IETS dI/dV spectra simulations

In this section, we introduce the IETS d*I*/d*V* spectra simulations and investigate in detail the spin Hamiltonian described for the FePc-Fe(C_6H_6) complexes in the main text. When applying the external magnetic field B in z-direction, we consider the following Hamiltonian for the complex:

$$H = J \cdot \vec{S}_{\mathrm{FePc}} \cdot \vec{S}_{\mathrm{Fe(C6H6)}} + D \cdot \hat{S}^2_{\mathrm{z,Fe(C6H6)}} - g_1 \mu_B B \hat{S}_{\mathrm{z,Fe(C6H6)}} - g_2 \mu_{\mathrm{B}} B \hat{S}_{\mathrm{z,FePc}} \tag{3}$$

Where J is the Heisenberg exchange coupling, D is the out-of-plane zero-field splitting term of the Fe(C_6H_6) spin, μ_{B} is the Bohr magneton, B is the external perpendicular magnetic field and g_1 and g_2 are g-factors of the Fe(C_6H_6) and FePc spins, respectively. In our case, $S_{\mathrm{FePc}} = 1/2$ and $S_{\mathrm{Fe(C6H6)}} = 1$. The energies and eigenstates of this mixed spin-(1/2,1) system can be expressed analytically[3]. The eigen-energies are:

$$E_{1,2} = \frac{1}{2}[J + 2D \mp (g_1\mu_B B + 2g_2\mu_B B)], \tag{4}$$

$$E_{3,4} = -\frac{1}{4}(J - 2D + 2g_2\mu_B B) \mp \frac{1}{4}\sqrt{[J - 2D - 2(g_1\mu_B B - g_2\mu_B B)]^2 + 8J^2}, \tag{5}$$

$$E_{5,6} = -\frac{1}{4}(J - 2D - 2g_2\mu_B B) \mp \frac{1}{4}\sqrt{[J - 2D + 2(g_1\mu_B B - g_2\mu_B B)]^2 + 8J^2}, \quad (6)$$

whereas the corresponding eigenvectors are

$$\psi_{1,2} = | \pm \frac{1}{2}, \pm 1 >, \quad (7)$$

$$\psi_{3,4} = c_1^{\mp} \left| +\frac{1}{2}, 0 > \mp c_1^{\pm} \right| -\frac{1}{2}, 1 >, \quad (8)$$

$$\psi_{5,6} = c_2^{\pm} \left| +\frac{1}{2}, -1 > \mp c_2^{\mp} \right| -\frac{1}{2}, 0 >. \quad (9)$$

The probability amplitudes in the last four eigenvectors (8) and (9) are:

$$c_1^{\pm} = \frac{1}{\sqrt{2}}\sqrt{1 \pm \frac{J - 2D - 2(g_1\mu_B B - g_2\mu_B B)}{\sqrt{[J - 2D - 2(g_1\mu_B B - g_2\mu_B B)]^2 + 8J^2}}},$$
$$c_2^{\pm} = \frac{1}{\sqrt{2}}\sqrt{1 \pm \frac{J - 2D + 2(g_1\mu_B B - g_2\mu_B B)}{\sqrt{[J - 2D + 2(g_1\mu_B B - g_2\mu_B B)]^2 + 8J^2}}}. \quad (10)$$

For $J \gg D, g_1\mu_B B, g_2\mu_B B$, the factors $c_{1,2}^{+} \cong \sqrt{\frac{2}{3}}$, $c_{1,2}^{-} \cong \sqrt{\frac{1}{3}}$.

At zero field $B = 0$ T (and $J \gg D$), this simplifies the energies to:

$$E_{1,2} = \frac{1}{2}(J + 2D), \quad (11)$$

$$E_{3,5} = -\frac{1}{4}(J - 2D) - \frac{1}{4}\sqrt{(J - 2D)^2 + 8J^2} \cong -J + \frac{2}{3}D, \quad (12)$$

$$E_{4,6} = -\frac{1}{4}(J - 2D) + \frac{1}{4}\sqrt{(J - 2D)^2 + 8J^2} \cong \frac{J}{2} + \frac{1}{3}D, \quad (13)$$

This approximation nicely illustrates that the ground state energy is given by $E_{3,5}$ (assuming antiferromagnetic exchange, i.e. $J > 0$). The wave functions of the ground state doublet are given as $\psi_{3,5}$:

$$|0\rangle = \psi_3 = \frac{1}{\sqrt{3}}\left|+\frac{1}{2}; 0\right\rangle - \frac{\sqrt{2}}{\sqrt{3}}\left|-\frac{1}{2}; +1\right\rangle$$

$$|1\rangle = \psi_5 = \frac{\sqrt{2}}{\sqrt{3}}\left|+\frac{1}{2};-1\right\rangle - \frac{1}{\sqrt{3}}\left|-\frac{1}{2};0\right\rangle \tag{14}$$

The corresponding energy level diagram is shown in Supplementary Fig. 3a (see also Fig. 2b). Two state manifolds form with $S_{\mathrm{tot}} \approx 1/2$ and $S_{\mathrm{tot}} \approx 3/2$ which are separated by an energy $\sim \frac{3}{2} \cdot J$. The excited state quartet is further split by $2/3 \cdot D$.

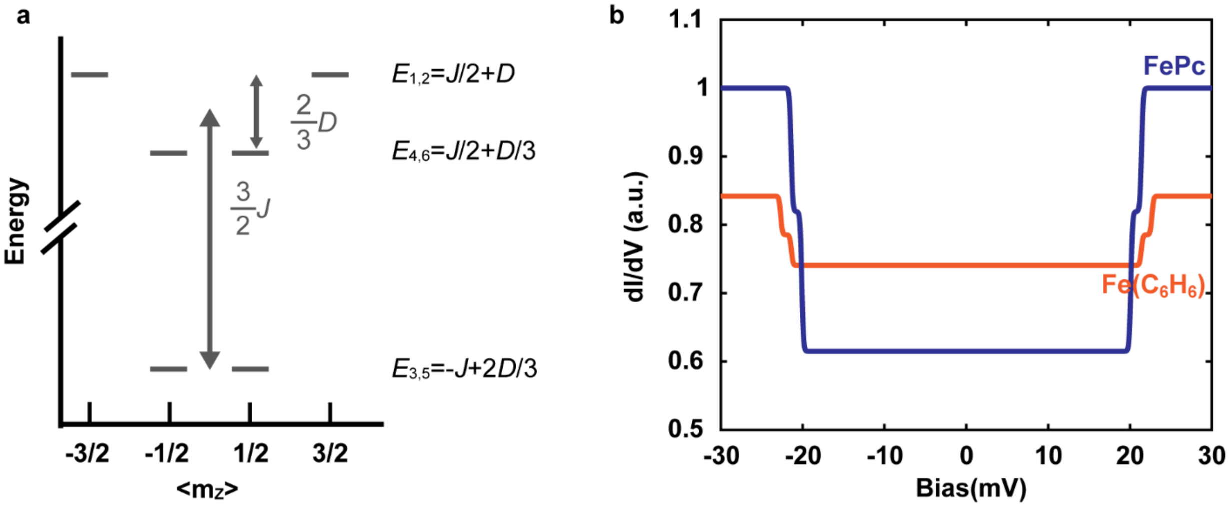

Supplementary Fig. 3. a, Energy level diagram of a mixed-spin (1/2,1) Heisenberg quantum ferrimagnet. **b,** Simulated IETS dI/dV spectra with S = ½ for FePc, S = 1 for Fe(C_6H_6). Fitting parameters: For Fe(C_6H_6): J = 14.65 meV, D = 1.9 meV, U = 0.945. For FePc: J = 13.8 meV. U = 0.494. T=0.8 K.

In the IETS simulations, we take the solution of the Hamiltonian described above to calculate the resulting tunneling current by using the spin simulation code developed in Ref.[4,5] . The latter considers contributions to the tunneling current stemming from 1) potential scattering *U* and 2) inelastic scattering of the tunneling electrons. The values of parameters D, J and U are adjusted for Fe(C_6H_6) and FePc in the complex to match the step heights and positions in the experiment: The simulated IETS dI/dV spectra shown in Supplementary Fig. 3b (see also Fig. 1c) reproduce the double step feature found in the experimental data well.

The IETS calculations and the resulting d*I*/d*V* spectra are crucially influenced by the assumptions made in the initial spin Hamiltonian. As such, the IETS spectrum plays an important role to determine the spin state of the Fe(C_6H_6) in the complex. We screened a wide variety of cases and found that in particular the double step feature (along with

constraints imposed by the results of the ESR measurements, remote sensing experiments as well as DFT calculations), can only be explained assuming $S_{\mathrm{FePc}} = ½$ and $S_{\mathrm{Fe(C6H6)}} = 1$ as shown in Supplementary Fig. 3. In the following, we will exemplarily illustrate, how other reasonable spin models are not in agreement with the data.

1) FePc: S = 1/2, Fe(C_6H_6): S = 2

A first guess for the FePc spin state and the Fe spin would be adapting those of the individual spin systems, i.e. $S_{\mathrm{FePc}} = ½$ and $S_{\mathrm{Fe(C6H6)}} = 2$. In this case, FePc and Fe(C_6H_6) retain their spin states as individual FePc (charged by one electron) and Fe on MgO. For the simulation, we initially assume that Fe has the same magnetic anisotropy ($D = -4.7\ \mathrm{meV}$[6]) as for isolated Fe atoms and that there is Heisenberg exchange coupling between the two spins. For ferromagnetic coupling ($J < 0$), there are two IETS steps and the separation between these steps increases with increasing J, while for antiferromagnetic coupling ($J > 0$), there are three IETS steps (Supplementary Fig. 4). None of these simulations are consistent with the experimental results. Here, we exclude the E-term, which would otherwise potentially split the states and lead to additional steps. A similar double-step feature, consistent with experimental dI/dV, can be qualitatively reproduced by using D ~ 20 meV and |J| ~ 1. However, this is not consistent with our other experimental results, such as remote sensing experiments (Supplementary Section 4).

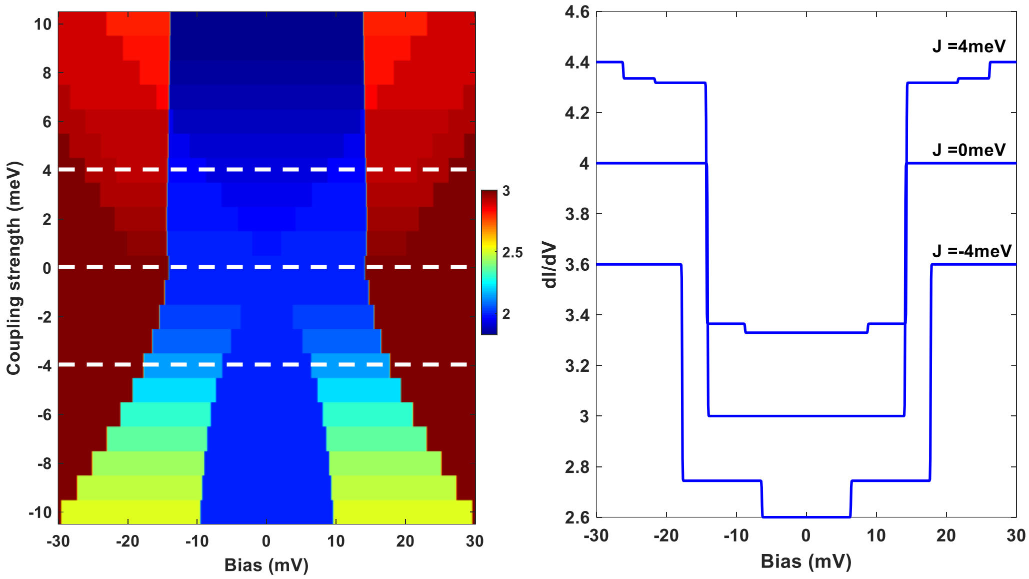

Supplementary Fig. 4. Simulated dI/dV spectra of a coupled spin system consisting of a S=1/2 and a S=2. For the $S = 2$ system, we assume anisotropy parameters $D = -4.7\,\mathrm{meV}$, $E = 0\,\mathrm{meV}$. Left: Colormap of simulated spectra atop the $S = 2$ system using different coupling strengths from $J = -10\,\mathrm{meV}$ to $10\,\mathrm{meV}$. Right: Selected spectra for $J = -4\,\mathrm{meV}$, $0\,\mathrm{meV}$ and $4\,\mathrm{meV}$. The spectra are shifted with an offset for clarity. Simulation uses 0.2 K and 0 T.

2) FePc S = 1, Fe(C_6H_6) S = 2

In this case, we assume a spin of 1 for FePc, as it does in the gas phase, and that Fe retains S = 2. Moreover, we assume Heisenberg exchange coupling between them. An exemplary choice of different anisotropy and coupling parameters is displayed in Supplementary Fig. 5 for coupling strength J ranging from -10 meV to 10 meV. We find that one can adjust J, D, and E-term to reproduce the double steps at around $\pm 20\,\mathrm{meV}$ (Supplementary Fig. 5), but there are more IETS steps at larger energies and always one step at around zero bias, which is absent in our experimental data.

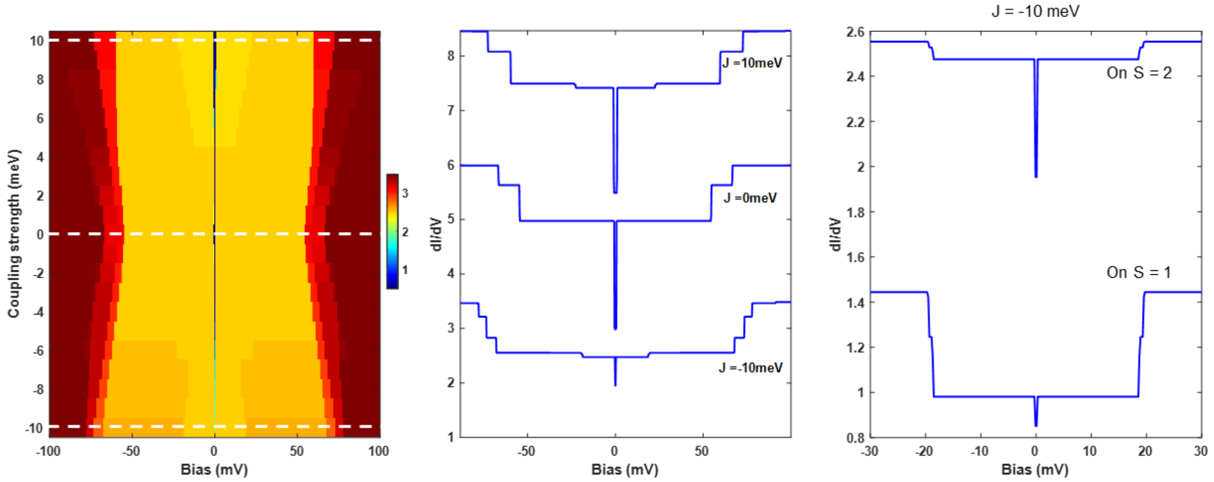

Supplementary Fig. 5. Simulated dI/dV spectra of a coupled spin system consisting of a S=1 and a S=2. Anisotropy parameters: $D = -20\ \mathrm{meV}$, $E = 2\ \mathrm{meV}$ (for $S = 2$) and $D = -3\ \mathrm{meV}$, $E = 2\ \mathrm{meV}$ (for $S = 1$) were exemplarily chosen, but a wide set of parameters was tested**.** Left: Colormap of simulated spectra for the $S = 2$ system using different coupling strengths from $J = -10\ \mathrm{meV}$ to $10\ \mathrm{meV}$. Middle: Selected spectra for $J = -10\ \mathrm{meV}$, $0\ \mathrm{meV}$ and $+10\ \mathrm{meV}$. Right: Zoom in plot for both FePc and Fe with $J = -10\ \mathrm{meV}$, showing two IETS steps at around $\pm 20\ \mathrm{meV}$. The spectra are shifted with an offset for clarity. Simulation uses 0.2 K and 0 T.

Besides the cases illustrated here, we also tested for instance a spin S = 1/2 coupled to a spin S= 3/2 or 5/2, which always results in more than two IETS steps or of two steps with incorrect position or intensity. Further constraints on the spin system will be discussed in section 4 and 5, which is only fulfilled by the spin model proposed and discussed at the beginning of this chapter.

3) Negativity

To explore the entanglement of the mixed spin-(1/2, 1) Heisenberg ferrimagnet, we employ the *negativity* serving as a measure of the pairwise entanglement [3,7,8]

$$\eta = \sum_j (|\lambda_j| - \lambda_j)/2 \qquad (15)$$

which is defined through eigenvalues λ_j of a partially transposed density matrix $\rho^{T_{1/2}}$ where ρ denotes the full density matrix. Here, $T_{1/2}$ denotes a partial transposition with respect to one subsystem (i.e. the spin-1/2).

Using the respective density operator $\rho = (|0\rangle\langle 0| + |1\rangle\langle 1|)/2$, one gets the following zero-temperature value of the negativity at zero magnetic field[3]:

$$\eta = \frac{\sqrt{\left(1-2\frac{D}{J}\right)^2+8}-(1-2\frac{D}{J})}{4\sqrt{\left(1-2\frac{D}{J}\right)^2+8}} \times \left[\sqrt{\frac{5\sqrt{\left(1-2\frac{D}{J}\right)^2+8}+3(1-2\frac{D}{J})}{\sqrt{\left(1-2\frac{D}{J}\right)^2+8}-(1-2\frac{D}{J})}}-1\right] \tag{16}$$

For $J \gg D, \eta = \frac{1}{3}$.

4. Magnetic sensing

To gain further insight into the spin state and magnetic moment of the $Fe(C_6H_6)$ in the complex, we used a close-by single Fe atom as a magnetic sensor to sense the complexes magnetic field[9]. The magnetic dipole–dipole interaction between the sensor Fe atom and the target spin causes a change of the resonance frequency of the sensor Fe, that is shifted to higher or lower frequencies depending on the spin direction and whether it is in its ground or excited state. Due to its large magnetic anisotropy, the magnetic moment μ_{Fe} of the sensor Fe is oriented out-of-plane along the applied magnetic field. Therefore, only the z-component of the target magnetic moment μ_{t}^z is measured and the frequency difference Δf between its ground and excited state is given by[9]

$$\Delta \mathrm{f} = \frac{4\mathrm{E}_{\mathrm{dd}}}{\mathrm{h}} = \frac{1}{\mathrm{h}} \cdot \frac{\mu_0}{\pi} \frac{1}{r^3} \mu_{\mathrm{Fe}}\, \mu_{\mathrm{t}}^z \tag{17}$$

where h is Planck's constant, μ_0 is the vacuum permeability and r is the distance between the two magnetic moments μ_{Fe} and μ_{t}. In the experiment, the ESR signals are measured using tip-field sweeps[10,11].

As a reference, we first measured the ESR signal of a single Fe atom that shows a single resonance peak (Supplementary Fig. 6, top). Second, we investigated an Fe-Fe dimer with a distance of 0.91 nm. The splitting of the resonance peak is $\Delta f = (2.1 \pm 0.3)$ GHz (Supplementary Fig. 6, middle). From this, the magnetic moment of Fe is determined to

be $\mu_{\text{Fe}} = (5.6 \pm 0.4)\ \mu_{\text{B}}$, which agrees well with previous measurements of $\mu_{\text{Fe}} = 5.4\ \mu_{\text{B}}$[9]. This good agreement indicates that magnetic dipole-dipole interaction is dominant at this distance, despite the close distance between the two spins - below 1 nm. Here, exchange interaction can often contribute significantly to the coupling[9].

Next, we position a sensor Fe atom 0.91 nm from the Fe site of the complex, the same distance as in the previous experiment. The influence of the FePc spin is negligible as its distance to the sensor Fe is roughly twice that of the $Fe(C_6H_6)$ in the complex. We find a frequency splitting of $\Delta f = (0.9 \pm 0.2)\ \text{GHz}$ (Supplementary Fig. 6, bottom), which is roughly half of the frequency splitting observed for a single Fe atom at the same distance. This corresponds to a magnetic moment of $\mu_{\text{Fe(C6H6)}} = (2.8 \pm 0.8)\ \mu_{\text{B}}$. This significantly smaller magnetic moment is incompatible with a $S = 2$ of the Fe atom. While the value of 2 μ_{B}, indicative of a spin 1 system without orbital contributions, is still within the error bar, we believe that likely exchange interaction is increasing the splitting due to the small distance between sensor and target[2,9]. This subsequently leads to a higher apparent magnetic moment in the sensing experiments.

Consequently, this reduction strongly suggests that the spin state of $Fe(C_6H_6)$ in the complexes is lower than S = 2.

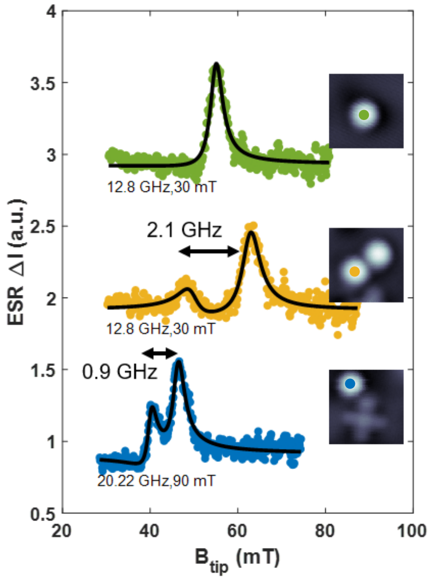

Supplementary Fig. 6. Magnetic sensing experiment. Tip-field sweep ESR measurements on a single Fe (green), a sensor Fe atom close to another Fe (yellow) and a sensor Fe atom close to the $Fe(C_6H_6)$ of an FePc-$Fe(C_6H_6)$ complex (blue). All data was taken with the same magnetic microtip. Insets: topographic

images (size: 2 nm × 2 nm). The inserted numbers display the used external field and RF frequency for the tip-field sweep. The resonance peaks are split by 2.1 GHz (yellow) and 0.9 GHz (blue), respectively. A linear background was subtracted from the data stemming from a general increase in signal with increase of the setpoint current. The black line indicates fits to one or two Lorentz functions.

5. DFT calculations

1) A $Fe(C_6H_6)$ toy model

To qualitatively understand the effect of Fe with an FePc ligand ring atop, we employ a toy model by placing a benzene ring on top of the Fe atom (as in Fig. 1d/1e but without MgO). Our DFT calculations show that without the presence of a benzene ring, the Fe *d*-states of individual Fe atoms are mostly found close to the Fermi level. For Fe with benzene ring atop, $Fe(C_6H_6)$, the crystal field generated by the benzene ring leads to a change of the orbital order and spin state that leads to bonding between Fe and (C_6H_6) and new molecular orbitals. Combining simple molecular orbital theory with our DFT (Supplementary Fig. 7), we find a strong overlap between the benzene E1 orbitals and d_{xz}, d_{yz} of the Fe, forming a strong set of π-bonds. This is also commonly the case for other metallocenes[12] and also rationalizes the stability of the $Fe(C_6H_6)$ complex. Similarly, the frontier orbitals are formed by an overlap between d_{xz}, d_{yz} and the (C_6H_6) E1 antibonding states. Still, the frontier orbitals maintain a strong *d*-character (Supplementary Fig. 8a). For instance, the d_{xz}-, d_{yz}-like orbitals account for 76% of $2e_1$ near E_F.

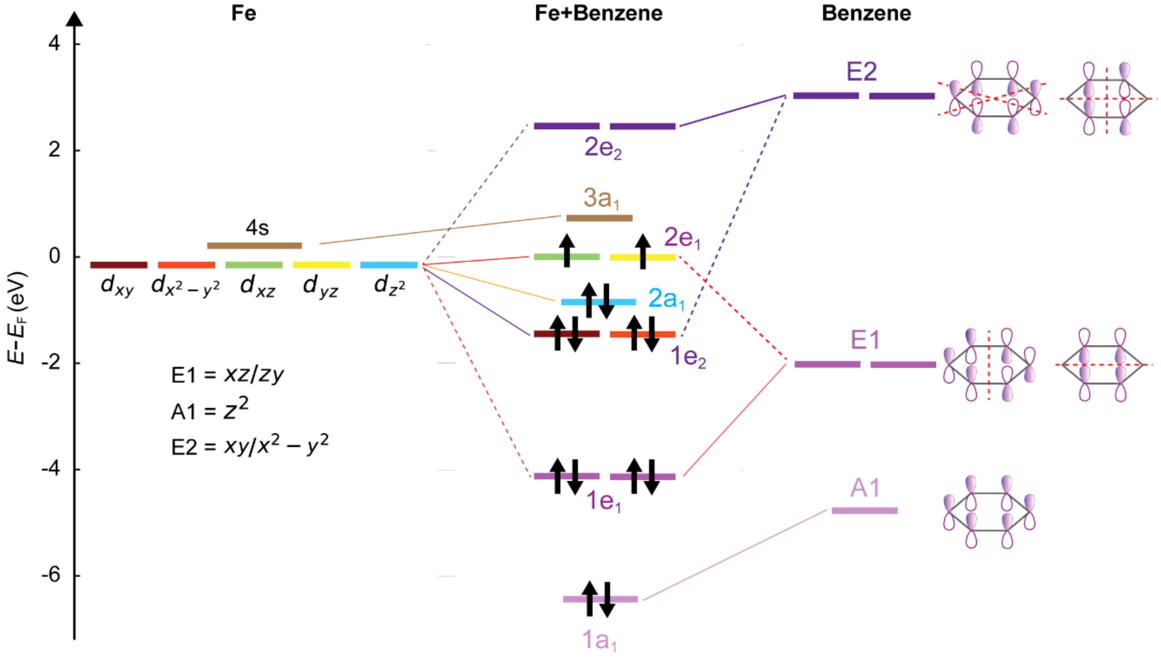

Supplementary Fig. 7. Molecular orbital energy diagram of Fe and Benzene (in vacuum). On the left side, Fe 3*d* and 4*s* states are shown with their respective symmetry group, on the right side the π molecular orbitals of benzene. In the center the combined molecular orbitals of Fe(C_6H_6) are shown. The lowest lying states are dominated by the benzene states ($1a_1$ 90%; $1e_1$ 84%) with some overlap of the 3*d* and 4*s* states. For the higher ones, the main contribution stems from the *d* states ($1e_2$ 72%; $2a_1$ 93%; $2e_1$ 76%). Energies and orbital character are obtained from DFT calculations. The addition of the MgO/Ag(001) surfaces leads to further hybridization of all states (See supplementary Fig. 8).

DFT calculations of the full system

We first confirmed that DFT accurately reproduced S = 2 of individual Fe atoms on the MgO/Ag(100) surface as discussed elsewhere[13]. From the local density of states (LDOS) in Fig. 1d, the Fe d-states are mostly found close to the Fermi level with the d_{z^2} orbital strongly hybridizing with the $4s$ orbital. This hybridization results in the d_{z^2} orbital being half-filled due to on-site repulsion. As a consequence, Fe has a final electron occupation of $3d^{6.5}4s^{0.8}$ obtained from a Lowdin charge analysis and four orbitals ($d_{z^2}/d_{xy}/d_{xz}/d_{yz}$) are close to being half-filled (Supplementary Table 1). This consequently indicates a spin $S = 2$, consistent with previous works[1,13]. To understand how the Fe(C_6H_6) spin state in the complex becomes $S = 1$, we employ again the simple model by placing a benzene ring on top of the Fe atom (Fig. 1e). We find that the benzene ring does not change the charge state of the Fe atom: Here, Fe exhibits a $3d^{6.8}4s^{0.3}$ configuration, indicating no significant charge transfer (Supplementary Table 1). However, the crystal field generated by the benzene ring changes the d orbital order: the d_{z2}, d_{xy} and d_{x2-y2} like orbitals are now shifted and lowered in energy, and the $4s - 3d_{z^2}$ hybridization is eliminated due to the depletion of the 4s in the presence of benzene ring. Consequently, only the d_{xz}/d_{yz} like orbitals are close to the Fermi energy and half-filled, suggesting an Fe spin state of $S = 1$.

**Configuration**	d_{z2}	d_{xy}	d_{xz}	d_{yz}	d_{x2-y2}	total
Fe (↑)	0.9263	0.9965	0.9962	0.9962	0.9836	**4.9**
Fe (↓)	0.5274	0.0034	0.0472	0.0472	0.9628	**1.6**
Fe+benzene (↑)	0.8525	0.8542	0.959	0.9592	0.8386	**4.5**
Fe+benzene (↓)	0.6793	0.6932	0.1314	0.1323	0.6826	**2.3**

Supplementary Table 1. Filling of the 3*d* shell obtained from a Lowdin charge analysis for Fe on 2ML MgO/Ag(001) without and with a benzene ring on top.

To shed light on the role of the substrate, we compare Fe-benzene formation in vacuum and on the MgO/Ag(001) surface (Supplementary Fig. 8). While there is more overlap with other states, the simple molecular orbital picture derived from the toy model still remains valid: Figure S8 shows that both deliver similar results. However, the surface

decreases the energy splitting between *d* states and further mixes the states by hybridization with the substrate.

DFT calculations including the full FePc molecule do not change our observations (see Supplementary Fig. 9). The 3*d* states near the Fermi level show a similar trend to $Fe(C_6H_6)$, however the tilted final geometry makes the analysis of the orbital character of the Fe 3*d* states less straightforward.

For completeness, we also show the spin-polarized DOS plots in Supplementary Fig. 10 where both spin-up and spin-down orbitals are included.

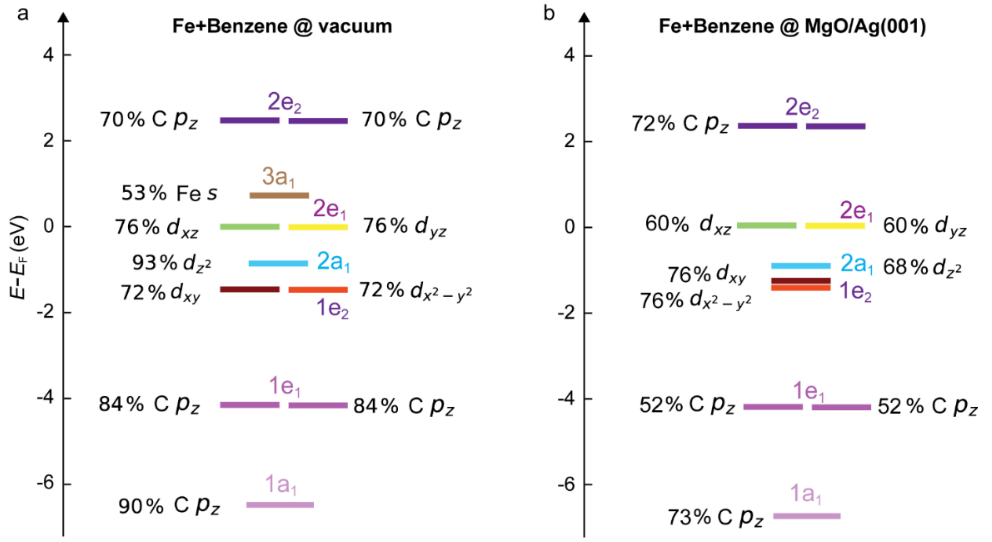

Supplementary Fig. 8. Molecular orbital energy diagrams of $Fe(C_6H_6)$ in different environments. a, in vacuum and **b,** on a MgO/Ag(001) substrate. The orbital weights are given in percent (%) listing the orbital of the dominating weight. The remaining contributions to 100% are hybridizations with other orbitals. Energies and character are obtained from DFT calculations.

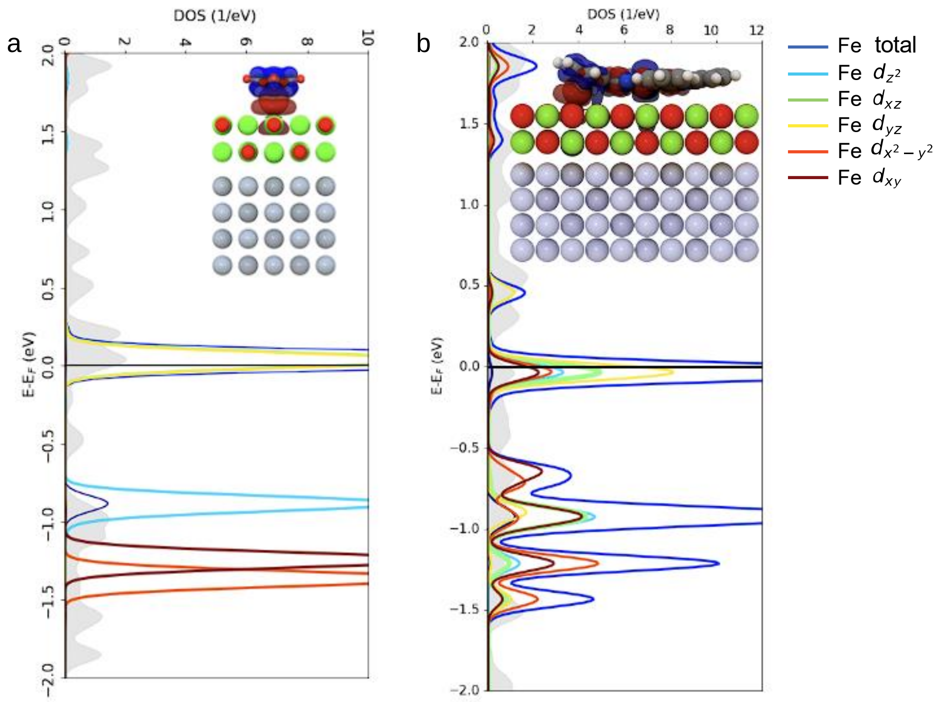

Supplementary Fig. 9. Atomic orbital projected DOS of Fe(C_6H_6) and FePc-Fe(C_6H_6). a, Fe 3*d* orbitals in the Fe-benzene complex used in the main text. **b,** the same plot but now with an FePc-Fe(C_6H_6) complex. The resulting states show more mixing but the behavior of the states close to E_F follows a similar trend as in a.

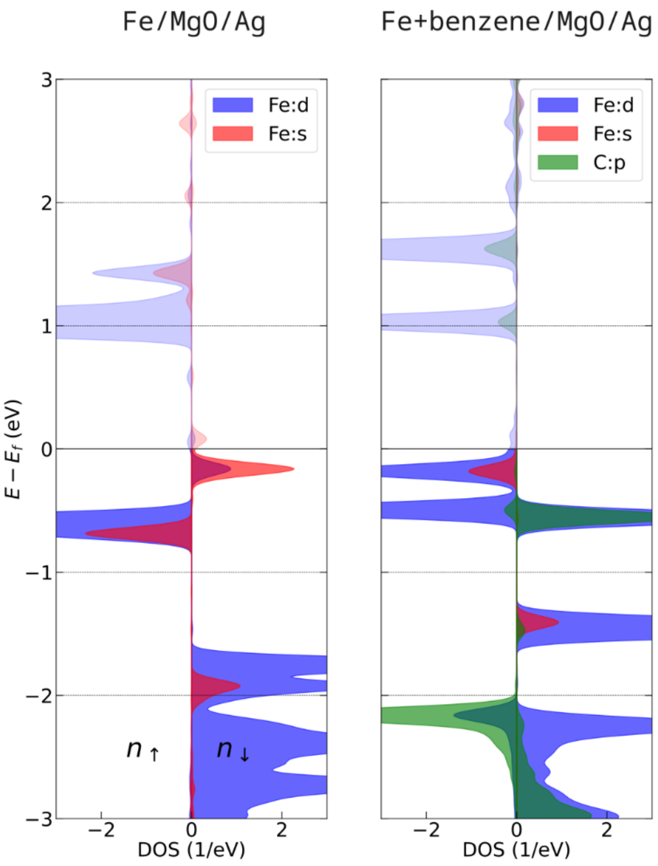

Supplementary Fig. 10. Projected density of states (PDOS) for Fe/MgO/Ag (left) and Fe+benzene/MgO/Ag (right) from a spin-polarized calculation. The contributions of Fe:d (blue), Fe:s (red), and C:p (green) orbitals are shown. Negative values corresponding to spin-up ($n_\uparrow$) and positive values to spin-down ($n_\downarrow$). The inclusion of benzene modifies the electronic structure, introducing additional contributions from C:p states and shifting the Fe:d-state distribution.

2) Exchange interaction within the complex

We use DFT calculations of an FePc-Fe(C_6H_6) complex to estimate the exchange coupling between the two spin centers using the broken-symmetry approach[2,14]. The magnetic exchange coupling strength is obtained by comparing the energy of the high spin (HS) and low-spin or broken symmetry (BS) states as:

$$J = \frac{-[E(HS) - E(BS)]}{s_{max}^2} \quad (18)$$

The high-spin solution corresponds to FM coupling whilst the BS solution is obtained from an antiferromagnetically coupled configuration and s_{max} is the total spin of the high-

spin state. We obtain an exchange coupling J for the Fe-FePc complexes of around 7 meV, which is of the same order as the experimental results (14 meV). We emphasize that this is the largest J observed for spins on MgO and is mediated via the FePc ligand and by far exceeds the exchange coupling of two Fe atoms at the same distance but without the ligand. This indicates that the exchange coupling is mediated through the ligand as also observed in FePc-FePc dimers[2] and FePc-Ti dimes[15]. We confirm that the exchange coupling in this system is strongly mediated by the ligand by slowly lifting the ligand up from the Fe atom in the calculation (Supplementary Fig. 11). In such a model, the exchange coupling constant is approximately exponentially dependent on the Fe atom – ligand distance. As the distance increases by tilting the ligand from 0° to 20°, the exchange coupling J decreases exponentially from 7 meV to 0.29 meV.

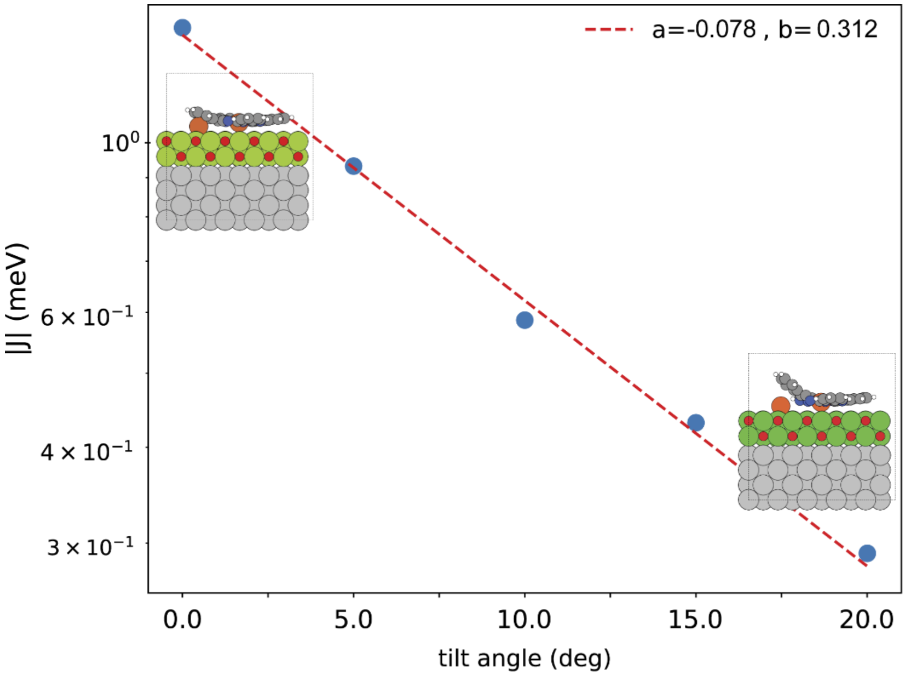

Supplementary Fig. 11. Exchange energy as a function of Fe atom – ligand distance. Inset: sketch showing 0° (left) and 20°(right) tilt angle configurations. The red dash line is a linear fit to data in log scale.

6. ESR and fitting

Figure 2(c-d) in the main text show the external magnetic field dependent ESR measurement at the FePc site and $Fe(C_6H_6)$ site of the same FePc-$Fe(C_6H_6)$ complex with the same tip. Fitting f_0 linearly as a function of B_z gives the magnetic moment of (1.008±0.007) μ_B at the $Fe(C_6H_6)$ site (Supplementary Fig. 12a) and (1.004±0.012) μ_B at the FePc center (Supplementary Fig. 12b), which is consistent to the effective spin ½ ground state doublet. Supplementary Fig. 12c shows the magnetic moments at both sites obtained for different FePc-$Fe(C_6H_6)$ complexes, giving an averaged magnetic moment of $(1.003 \pm 0.025)\mu_B$. While this is close to an effective magnetic moment of 1 μ_B, the variations are larger than the individual errorbars. We suggest that this results from the influence of the magnetic tip field[16] as well as changes in the local environment of the spin center, i.e. given by defects and strain in the substrate.

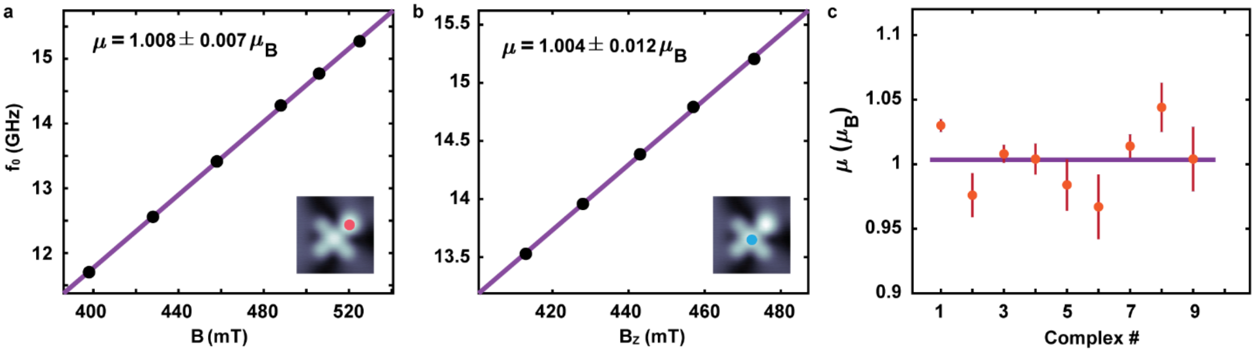

Supplementary Fig. 12. Magnetic moment of FePc-$Fe(C_6H_6)$ complexes. Resonance frequency f_0 as a function of an out-of-plane external magnetic field B. **a,** $Fe(C_6H_6)$ site ($I_{\mathrm{set}} = 5$ pA / $V_{\mathrm{set}} = 25$ mV); **b,** FePc site ($I_{\mathrm{set}} = 50$ pA / $V_{\mathrm{set}} = 70$ mV) extracted from Fig. 2e,f in the main text. **c,** Statistics of magnetic moments in different FePc-$Fe(C_6H_6)$ complexes.

7. Rabi rate as a function of V_{RF}

We extract the Rabi rate at different V_{RF} from Fig. 3b in the main text, and a linear fit yields a slope of $9.6 \pm 0.3\ \mathrm{rad}/(\mu s \cdot mV)$ (Supplementary Fig. 13). This slope is 5 times higher than that of pristine FePc [$1.86 \pm 0.13\ \mathrm{rad}/(\mu s \cdot mV)$ in Ref.[17]] and 3-7 times larger than values reported for Ti atoms [$\sim 1.3\ \mathrm{rad}/(\mu s \cdot mV)$ in Ref.[18] and $\sim 2.8\ \mathrm{rad}/(\mu s \cdot mV)$ in Ref.[19]]. In the following we discuss possible reasons for these results.

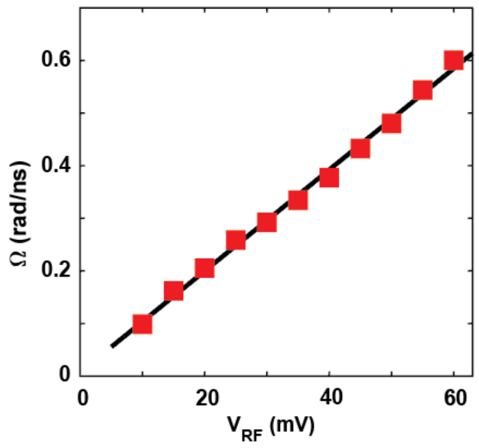

Supplementary Fig. 13. Extracted Rabi rate Ω as a function of V_{RF} from Fig. 3b in main text. The black line is a linear fit to the data with a slope of $9.6 \pm 0.3\ \mathrm{rad}/(\mu s \cdot mV)$.

The driving mechanism of individual atoms and molecules in ESR-STM is still subject to current research, both theoretically and experimentally[19-23] . We first discuss the ESR driving mechanism in the framework of the frequently employed piezoelectric displacement model[20], which relies on the displacement of the surface spin and a subsequent modulation of one of the parts of the Hamiltonian. Employing the same formalism as presented in Ref. [20], we write

$$\Omega(t) = \frac{F}{h} z(t)$$

where the Rabi rate Ω depends on the oscillating displacement $z(t)$ and the Rabi force F. The latter can have different origins[20]. The dominating term was found theoretically[20] and experimentally[18] to originate from exchange interaction of the tip magnetic moment and the surface spin leading to an effectively oscillating local magnetic field. Thus, a higher Rabi rate can originate either from an increase in $z(t)$ or F.

A straightforward description of the displacement is given by

$$z(t) = \frac{q}{k} \frac{V_{RF}(t)}{d}$$

Here, it is assumed that the time-dependent electric field $E(t) = \frac{V_{RF}(t)}{d}$ couples via Hooke's law to the surface spin with an effective charge q and effective spring constant k, i.e. the stiffness of the vibrational mode of the spin center on the surface.

Thus, a larger displacement can either be caused by a different q of the surface spin, a less stiff interaction to the surface k, or simply a different initial distance d between the surface spin and the tip.

In addition, the Rabi force itself is potentially stronger for the $Fe(C_6H_6)$ site in the complex:

$$F = \frac{\partial J(z)}{\partial z} \langle \vec{S}_T \rangle \cdot \langle 0 | \vec{S} | 1 \rangle$$

Here, $\frac{\partial J(z)}{\partial z}$ is the gradient of the exchange interaction between the surface spin and the tip magnetic moment $\langle \vec{S}_T \rangle$. $\langle 0 | \vec{S} | 1 \rangle$ is the transition matrix element, emphasizing that a finite overlap between two states is necessary for their connection. Thus, both $\frac{\partial J(z)}{\partial z}$ and $\langle 0 | \vec{S} | 1 \rangle$ can cause an increase in Ω. While the former is difficult to estimate, the latter can be calculated from the Hamiltonian of the FePc-$Fe(C_6H_6)$ complex given in the main text for the $Fe(C_6H_6)$ site. When comparing this to a normal spin ½, we find a 33% increase (see Supplementary Table 2). This increase is very likely to play a role in the faster Rabi rate observed for the FePc-$Fe(C_6H_6)$ complex, but cannot explain the full effect.

Beyond the piezoelectric displacement model, it was proposed by some of the authors[22], that the electric field of the tip can directly modulate the charges of the atoms without displacement. This leads for spin dimers to a modulation of the J-coupling and thus explains experimental work on Fe-Ti spin pairs quite well[19]. Thus, for the FePc-$Fe(C_6H_6)$ complex, J-modulation between the Fe and FePc spin adds another possibility for a higher Rabi rate.

Lastly, in another recent work, some of the authors proposed an electron transport model[24], which predicts that the magnitude of the Rabi term mainly depends on tip polarization and the DC bias magnitude compared to the ionization energy of the adsorbate. In particular, the latter changes for the new molecular orbitals emerging in the complex (Fig. 1e).

In general, we believe that a combination of the different origins discussed here play a role for the enhanced Rabi rate: This includes the summarized effects to the displacement $z(t)$, differences in the Rabi force, the strong exchange coupling as well as a change in ionization energy in the dimer.

Matrix Element	Spin ½ (FePc)	Spin (1, ½) Ferrimagnet (Complex)
$\lvert\langle 0 \lvert S_x \rvert 1 \rangle\rvert$	0.5	0.667
$\lvert\langle 0 \lvert S_y \rvert 1 \rangle\rvert$	0.5	0.667
$\lvert\langle 0 \lvert S_z \rvert 1 \rangle\rvert$	0	0

Supplementary Table 2. Table of transition matrix elements of the respective ESR transition for different spin systems. For the FePc-Fe(C_6H_6) complex, the transition matrix element is determined for the Fe site which corresponds to the case in the Rabi measurements.

8. Conductance-dependent spin lifetime

To further investigate the processes that limit the spin lifetime T_1, we performed pump-probe measurements as a function of tip-sample conductance, $G_{ts} = I/V$, for both pristine FePc (Supplementary Fig. 14a) and FePc in the complex (Supplementary Fig. 14b). In both cases, the lifetime drops dramatically as the tip approaches the spin centers. At large tip-sample distances (i.e. low G_{ts}), T_1 reaches a plateau where it becomes independent of tip-sample distance. This plateau, defined as T_1^0, is also independent of the specific tip and molecules in use. We therefore conclude that T_1^0 represents the intrinsic relaxation lifetime on a 2ML MgO surface. The effect of the tip height on T_1 can be understood by a model based on electron-hole generation in the nearby electrodes, i.e. the metallic substrate and tip, as described in Ref.[25] for single Fe atoms on MgO/Ag(001). According to this model, T_1 is a function of sample–sample (G_{ss}), tip–sample (G_{ts}) and tip–tip ($G_{tt} = \frac{G_{ts}^2}{G_{ss}}$) conductance: $T_1 = \frac{T_1^0}{(1+\frac{G_{ts}}{G_{ss}})^2}$ (see Ref.[25]). Fitting T_1 as a function of G_{ts} yields T_1^0 of (0.41 ± 0.05) μs for pristine FePc (solid blue curve in Supplementary Fig. 14a) and (1.6 ± 0.6) μs for the complex (solid red curve in Supplementary Fig. 14b). In Supplementary Fig. 14c, we show the longest T_1 achieved at a large tip-sample distance ($G_{ts} = \frac{20}{200}$ pA/mV). The data reveals a T_1 of (1428 ± 323) ns for FePc in the complex and (363 ± 26) ns for pristine FePc, which are consistent with the values from our model. At large tip-sample distance, the lifetime is limited by the sample-sample conductance G_{ss}.

We believe that T_1 can be further enhanced by increasing the MgO layer thickness as it is the case for individual Fe atoms[25].

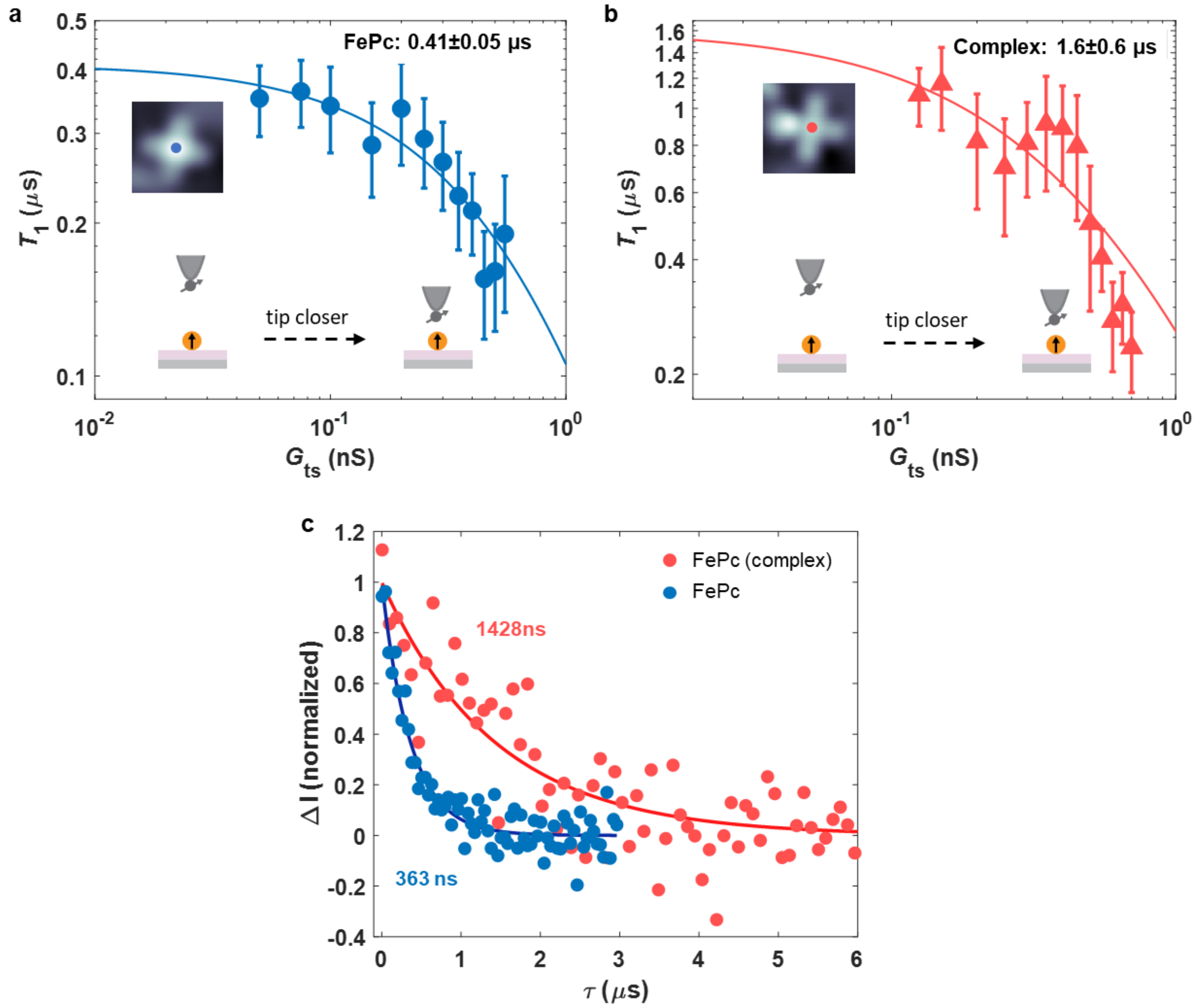

Supplementary Fig. 14. Conductance-dependent spin lifetime T_1 as a function of tip-surface conductance. **a**,**b** T_1 as a function of tip-surface conductance for pristine FePc and FePc site in the complex. (setpoint: $V_{set} = 200$ mV, $I_{set} = = 10 - 100$ pA for FePc, $I_{set} = 25 - 140$ pA for the complex). All data are measured with the same tip. Error bars are determined by exponential fits to pump–probe data. The solid lines are fits. Fitted parameters: $T_1^0 = (1.6 \pm 0.6)$ μs, $G_{ss} = 0.4$ nS (complex), and $T_1^0 = (0.41 \pm 0.05)$ μs, $G_{ss} = 0.21$ nS (FePc). **c**, T_1 for a pristine FePc (blue) and FePc in the complex (red) on 2 ML MgO/Ag(001) at low G_{ts}. [parameters: $I_{\text{set}} = 20$ pA, $V_{\text{set}} = 200$ mV, $B = 600$ mT, $V_{\text{pump}} = 80$ mV, $V_{\text{probe}} = 40$ mV, $\tau_{\text{cycle}} = 6$ μs (FePc site in the complex), $\tau_{\text{cycle}} = 3$ μs (FePc)].

9. Spin transport simulations and enhanced spin lifetime

1. Spin transport simulations

In the following, we discuss the spin transport simulation in greater detail. These are employed to explain the improvement in spin lifetime, which in the main text, we ascribe to the correlation of the two spins in the complex. For an electron tunneling process through an STM tunnel barrier featuring a single spin, the intensity of the transition from a given initial state $|\varphi_i, \psi_i\rangle$ to a final state $|\varphi_f, \psi_f\rangle$, i.e. the transition matrix element, is given as (Eq. 13 and 14 in Ref.[5])

$$|M_{if}|^2 = \left|\left\langle \varphi_f, \psi_f \middle| \frac{1}{2}\boldsymbol{S}\cdot\boldsymbol{\sigma} + U \middle| \varphi_i, \psi_i \right\rangle\right|^2 = |m_{if}|^2 + |U|^2\delta_{if} + 2\cdot \mathrm{Re}[u \times m_{if}]\delta_{if} \qquad (19)$$

Here, $|\varphi\rangle$ ($|\psi_i\rangle$) describes states of the electron baths (spin system). The interaction between the tunneling electron and the localized spin is described as an exchange interaction of the form $\frac{1}{2}\boldsymbol{S}\cdot\boldsymbol{\sigma}$. Here, $\boldsymbol{S}$ and $\boldsymbol{\sigma}$ are the spin vector operators of the local spin and the tunneling electron, respectively. The (dimensionless) parameter U accounts for spin-independent components of the interaction, i.e. Coulomb potential scattering. $m_{if} = \left|\left\langle \varphi_f, \psi_f \middle| \frac{1}{2}\boldsymbol{S}\cdot\boldsymbol{\sigma} \middle| \varphi_i, \psi_i \right\rangle\right|$ also contains the inelastic scattering between the two spins. Supplementary Fig. 15 illustrates the composition of the different contributions to a d*I*/d*V* measurement including elastic and inelastic channels as derived in Ref.[5]. From the d*I*/d*V* measurement, the ratio of inelastic scattering (blue area in Supplementary Fig. 15) and spin conserving transport processes can be determined in an external magnetic field. In order to obtain the probability for inelastic scattering we thus simulated both FePc and complex spectra and extracted the ratio between inelastic and elastic tunneling channels. We take the spin configuration from the d*I*/d*V* and ESR analysis performed in the main text (Complex: $S_{\mathrm{Fe(C6H6)}} = 1$, $S_{\mathrm{FePc}} = ½$, $J = 14\,\mathrm{meV}$, $D = 1.8\,\mathrm{meV}$, $U_{\mathrm{Fe}} = 1$, $U_{\mathrm{FePc}} = 0.5$; isolated FePc: $S_{\mathrm{FePc}} = ½$, $U_{\mathrm{FePc}} = 0.5$ taken form Ref.[2]). In the simulation, we additionally apply a magnetic field of 5 T in order to separate inelastic and elastic contributions at around zero bias (As shown in Supplementary Fig. 15). The exemplary ratios from these simulations are shown in Supplementary Fig. 16a for both the complex and the pristine FePc. As it can be seen, the probability of inelastic scattering, i.e. the height of the IETS

step is significantly reduced for the complex, in particular for the FePc site. In Fig. 3f in the main text we show the continuous evolution of this ratio as a function of exchange coupling between the two spins using the code in Ref.[5], which tunes the system continuously from the pristine FePc into the ferrimagnet configuration.

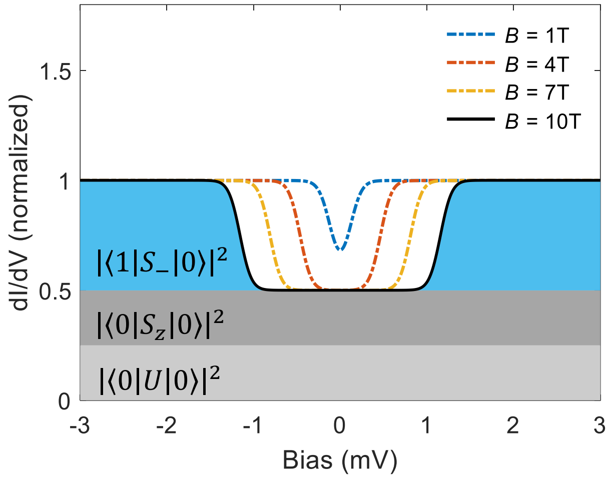

Supplementary Fig. 15. Simulated tunneling spectra for a S=1/2 system at different magnetic fields. The different contributions to the conductance are labeled. Blue: Conductance due to inelastic spin-spin scattering with changing the localized spin, Dark gray: elastic spin-spin scattering without changing the spin, light gray. Elastic Coulomb potential scattering with $|U| = 0.25$. The spectra are normalized and g = 2 here. T was set to 0.5 K.

While the continuous evolution is obtained numerically, in the limiting case of large J (maximum entanglement) and J = 0 (pristine FePc), the ratio can also be calculated directly: The transfer matrix element is determined by the initial and final states and the spin operators:

$$\hat{\boldsymbol{\sigma}} \cdot \hat{\boldsymbol{S}} = \frac{1}{2}\hat{\sigma}_+\hat{S}_- + \frac{1}{2}\hat{\sigma}_-\hat{S}_+ + \hat{\sigma}_z\hat{S}_z,$$

which governs spin-exchange processes between tunneling electrons and the surface spin (Supplementary Fig. 15). In the FePc-Fe(C_6H_6) complex, the exchange interaction modifies the initial and final states, ultimately changing the transfer matrix elements as summarized in the table below. Since the U parameter remains the same for pristine FePc and FePc in the complex, the elastic scattering caused by potential scattering U is the same.

Pristine FePc	FePc in the complex	$Fe(C_6H_6)$ in the complex
Ground state: $\lvert 0\rangle = \left\lvert +\frac{1}{2}\right\rangle$, $\lvert 1\rangle = \left\lvert -\frac{1}{2}\right\rangle$	Ground state: $\lvert 0\rangle = \frac{1}{\sqrt{3}}\left\lvert +\frac{1}{2};0\right\rangle - \frac{\sqrt{2}}{\sqrt{3}}\left\lvert -\frac{1}{2};+1\right\rangle$, $\lvert 1\rangle = \frac{\sqrt{2}}{\sqrt{3}}\left\lvert +\frac{1}{2};-1\right\rangle - \frac{1}{\sqrt{3}}\left\lvert -\frac{1}{2};0\right\rangle$	
$\langle 0\lvert \hat{S}_+\rvert 1\rangle = 1$	$\langle 0\lvert \hat{S}_+\rvert 1\rangle = -1/3$	$\langle 0\lvert \hat{S}_+\rvert 1\rangle = 4/3$
$\langle 1\lvert \hat{S}_-\rvert 0\rangle = 1$	$\langle 1\lvert \hat{S}_-\rvert 0\rangle = -1/3$	$\langle 1\lvert \hat{S}_-\rvert 0\rangle = 4/3$
$\langle 0\lvert \hat{S}_z\rvert 0\rangle = 1/2$	$\langle 0\lvert \hat{S}_z\rvert 0\rangle = -1/6$	$\langle 0\lvert \hat{S}_z\rvert 0\rangle = -2/3$
$\langle 1\lvert \hat{S}_z\rvert 1\rangle = -1/2$	$\langle 1\lvert \hat{S}_z\rvert 1\rangle = 1/6$	$\langle 1\lvert \hat{S}_z\rvert 1\rangle = 2/3$
$\mathrm{U} = 1/2$	$\mathrm{U} = 1/2$	$\mathrm{U} = 1$

Following Ref.[5], we can rewrite the spin-exchange scattering term in equation (19) as

$$\left\lvert m_{if}\right\rvert^2 = \left\lvert\left\langle \varphi_f, \psi_f \middle\vert \frac{1}{2}\boldsymbol{S}\cdot\boldsymbol{\sigma} \middle\vert \varphi_i, \psi_i\right\rangle\right\rvert^2$$

$$= \frac{1}{4}\left(\frac{1}{2}\left\lvert\left\langle \varphi_f, \psi_f \middle\vert \hat{S}_- \middle\vert \varphi_i, \psi_i\right\rangle\right\rvert^2 + \frac{1}{2}\left\lvert\left\langle \varphi_f, \psi_f \middle\vert \hat{S}_+ \middle\vert \varphi_i, \psi_i\right\rangle\right\rvert^2 + \left\lvert\left\langle \varphi_f, \psi_f \middle\vert \hat{S}_z \middle\vert \varphi_i, \psi_i\right\rangle\right\rvert^2\right)$$

The probability for inelastically scattered tunneling electrons is then given by the ratio of spin-flip processes and all tunneling channels (Supplementary Fig. 15). This results in

$$P = \frac{\frac{1}{2}\left\lvert\left\langle 1\middle\vert\hat{S}_-\middle\vert 0\right\rangle\right\rvert^2}{\frac{1}{2}\left\lvert\left\langle 1\middle\vert\hat{S}_-\middle\vert 0\right\rangle\right\rvert^2 + \left\lvert\left\langle 0\middle\vert\hat{S}_z\middle\vert 0\right\rangle\right\rvert^2 + 4\lvert\langle 0\vert U\vert 0\rangle\rvert^2}$$

Which could equivalently be formulated for $\left\lvert\left\langle 0\middle\vert\hat{S}_+\middle\vert 1\right\rangle\right\rvert^2$. Using this expression, we obtain P = 28.6% for pristine FePc, 16.7% for $Fe(C_6H_6)$ in the complex, and 5.1% for FePc in the complex, as shown in Supplementary Fig. 16a. This demonstrates the effect of exchange interaction in the complex on inelastic scattering.

Thus, compared to pristine FePc, the reduction in inelastic spin-flip fraction for FePc in the complex is mostly attributed to the large *J* which leads to strong correlation between two spins. This emphasizes that the correlation in the spin system is crucial to protect the spin from inelastic scattering contributions in the environment. While these simulations are based solely on the parameters extracted from the zero-field d*I*/d*V* spectra measured with non-spin polarized tips, we can additionally obtain these fractions from experimental d*I*/d*V* spectra which are discussed in the following section.

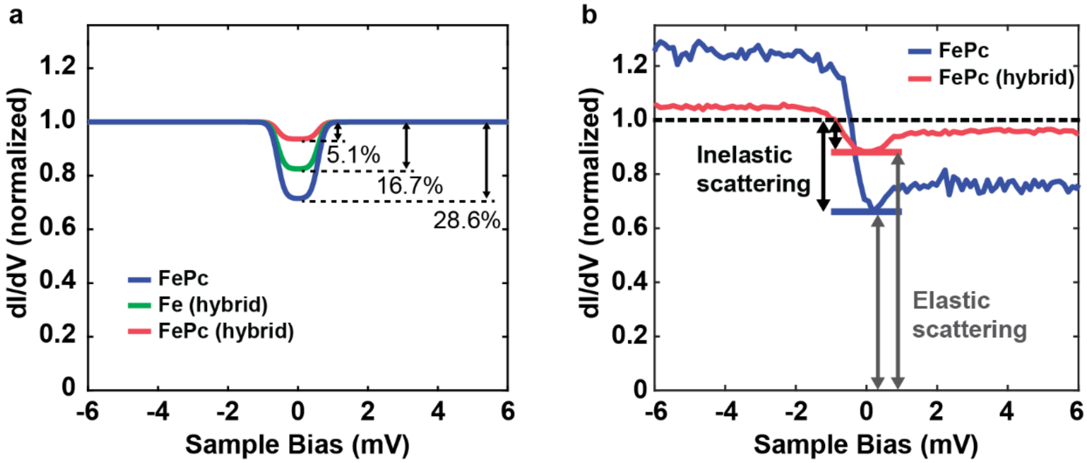

Supplementary Fig. 16. Inelastic Scattering in d*I*/d*V*. a, Simulated dI/dV of FePc and of the complex: $S_{\mathrm{Fe(C6H6)}} = 1$, $S_{\mathrm{FePc}} = ½$, $J = 14$ meV, $D = 1.8$ meV, $U_{Fe} = 1$, $U_{\mathrm{FePc}} = 0.5$; isolated FePc: $S_{\mathrm{FePc}} = ½$, $U_{\mathrm{FePc}} = 0.5$ taken form Ref. [2] . T = 0.8 K, B = 5 T. The probability for inelastically scattered tunneling electrons is calculated and shown in the inset. **b,** Normalized d*I*/d*V* measurements with a spin-polarized tip to determine the fraction of inelastic scattering for the pristine FePc, as well as $Fe(C_6H_6)$ and FePc in the complex. Data taken with a magnetic tip, in order to induce a large Zeeman splitting (Tunneling Parameter: FePc: $I_{\mathrm{set}} = 1$ nA / $V_{\mathrm{set}} = -30$ mV; FePc (complex): $I_{\mathrm{set}} = 0.5$ nA / $V_{\mathrm{set}} = -30$ mV).

2. Experimental determination of inelastic scattering probability

Besides the spin transport simulations, which offer a detailed picture of the influence of the exchange coupling and correlation on inelastic scattering processes, we can also experimentally determine the probability of inelastic scattering by performing d*I*/d*V* measurements on the FePc and the complex.

Supplementary Fig. 16b shows d*I*/d*V* spectra, which were recorded using a magnetic tip with a field strong enough to split the doublet ground state. Usually, an external magnetic field is used for this kind of measurements, which however in our experimental setup is

not strong enough to sufficiently split the two states. The spectra in Supplementary Fig. 16b include both the isolated FePc and measurements on the FePc site in the complex. For all spectra, the signal above the inelastic transition is asymmetric for different bias polarity due to the additional spin-polarization of the magnetic tip[5]. Thus, we normalize the spectra to the average of the two step heights, which corresponds to the level in the non-spin-polarized case. As can be seen, the fraction of inelastic scattering (blue) is largest for FePc, and decreases for the FePc site in the complex. The ratio of inelastic scattering can be read off and is shown for different measurements (different FePcs/complexes and different tips) in the main text in Fig. 3f.

10. ESR transitions in a Heisenberg two-spin system with the tip field detuning effect

In the main text, we argue that the two coupled complex spins (four spins) can be treated effectively as two coupled spin ½, such as found in Ref. [2,26]. In the following, we will derive the essential equations necessary to describe the two coupled complexes as effectively two coupled spin ½. Moreover, we will discuss the antiferromagnetically (AFM) coupled case, in contrast to the ferromagnetically (FM) case discussed in the main text.

Using the secular approximation, the Hamiltonian in the main text can be rewritten as

$$H = -g_1\mu_{\mathrm{B}}\left(B_{\mathrm{tip}} + B\right)\cdot S_1^z - g_2\mu_{\mathrm{B}}B\cdot S_2^z + \left(J_2^{\mathrm{eff}} + 2D\right)\cdot\ S_1^zS_2^z + (J_2^{\mathrm{eff}} - D)\left(S_1^xS_2^x + S_1^yS_2^y\right)$$

Here z is aligned with $\boldsymbol{B}$, μ_{B} is the Bohr magneton, and J and D represent the exchange and dipole coupling strengths, respectively. Labels 1 and 2 indicate the first and second complex. Thus, the first two terms are the Zeeman splittings of the respective complexes. The detuning, i.e. the difference between the Zeeman splittings of the two complex spins, is given by the imbalance of these two terms, i.e. $\delta = g_1\mu_{\mathrm{B}}\left(B + B_{\mathrm{tip}}\right) - g_2\mu_{\mathrm{B}}B$. The coupling constant for the dipolar coupling is given by $D = \frac{D_0}{2}(1 - 3\cos^2\theta)$ with $D_0 = \frac{\mu_0\gamma_1\gamma_2\hbar^2}{4\,\pi\,r^3}$, where $\gamma_{1,2} = g_{1,2}\mu_B/\hbar$ are the gyromagnetic ratios of the two complex spins. Here, $\theta = 90°$ represents the angle between the external field and the connection vector $\boldsymbol{r}$ of the two complex spins, resulting in $D = \frac{D_0}{2}$. The magnetic dipolar coupling treats the effective coupling between the two complex spins. Using Zeeman product states $|00\rangle, |10\rangle, |01\rangle, \text{and } |11\rangle$ as a computational basis, the eigenstates and corresponding eigenenergies of the coupled complex Hamiltonian are:

n	Eigenstate $|n\rangle$	Eigenenergy E_n
0	$|00\rangle$	$-\frac{1}{2}\left(g_1\mu_\mathrm{B}\left(B+B_\mathrm{tip}\right)+g_2\mu_\mathrm{B}B\right)+\frac{1}{4}\left(J_2^\mathrm{eff}+2D\right)$
1	$|-\rangle: -\frac{\alpha}{\sqrt{1+\alpha^2}}\,|01\rangle\,+\,\frac{1}{\sqrt{1+\alpha^2}}\,|10\rangle$	$-\frac{1}{2}\sqrt{\delta^2+\left(J_2^\mathrm{eff}-D\right)^2}-\frac{1}{4}\left(J_2^\mathrm{eff}+2D\right)$
2	$|+\rangle: \frac{1}{\sqrt{1+\alpha^2}}\,|01\rangle\,+\,\frac{\alpha}{\sqrt{1+\alpha^2}}\,|10\rangle$	$\frac{1}{2}\sqrt{\delta^2+\left(J_2^\mathrm{eff}-D\right)^2}-\frac{1}{4}\left(J_2^\mathrm{eff}+2D\right)$
3	$|11\rangle$	$\frac{1}{2}\left(g_1\mu_\mathrm{B}\left(B+B_\mathrm{tip}\right)+g_2\mu_\mathrm{B}B\right)+\frac{1}{4}\left(J_2^\mathrm{eff}+2D\right)$

The coupling terms cause the formation of two mixed states $|+\rangle$ and $|-\rangle$, which are a superposition of the two Zeeman states $|01\rangle$ and $|10\rangle$ and can be characterized by the parameter α, which depends on the detuning δ and the exchange coupling J_2.

$$\alpha=\frac{\delta+\sqrt{\delta^2+\left(J_2^\mathrm{eff}-D\right)^2}}{J_2^\mathrm{eff}-D}$$

$$\delta=g_1\mu_\mathrm{B}\left(B+B_\mathrm{tip}\right)-g_2\mu_\mathrm{B}B$$

Four possible transitions could occur, labeled by the corresponding ESR frequencies: f_1 for $|00\rangle\rightarrow|-\rangle$, f_2 for $|+\rangle\rightarrow|11\rangle$, f_3 for $|00\rangle\rightarrow|+\rangle$ and $f_4$for $|-\rangle\rightarrow|11\rangle$.

These four transitions are those shown in Fig. 4c-f in the main text and in Figs. S17-19. The frequency difference between $f_1(f_3)$ and $f_2(f_4)$ depends on J_2^eff and D given by:

$$\Delta f_1=f_2-f_1=f_4-f_3=J_2+2D=J_2^\mathrm{eff}+D_0.$$

When the exchange coupling is dominant ($\left|J_2^\mathrm{eff}\right|\gg D_0$), Δf_1 can be either positive ($J_2^\mathrm{eff}>0$, AFM coupling) or negative ($J_2^\mathrm{eff}<0$, FM coupling). In the main text, we demonstrate that the two complex spins are coupled ferromagnetically. However, they can also be coupled antiferromagnetically when the two $Fe(C_6H_6)$ in each complex are positioned close together. The coupling sign can be determined by comparing the relative ESR peak heights of f_3 (f_1) and $f_4(f_2)$. Since for the AFM case (Supplementary Figs. 17a and 18)

the ESR peak at $f_3(f_1)$ coresponds to the ground state of the neighboring complex spin (the spin under the tip), the ESR peak at f_3 (f_1) is taller and appears at a lower frequency than at $f_4(f_2)$. In the FM case, the mixed states $|-\rangle$ and $|+\rangle$ move to higher energies, causing $f_3(f_1)$ to appear at a higher frequency than $f_4(f_2)$. Generally speaking, the peak at lower frequencies is taller for AFM (Supplementary Figs. 17a and 18), while the taller peak is at higher frequencies in the FM case (Fig.4 and Supplementary Fig. 17b).

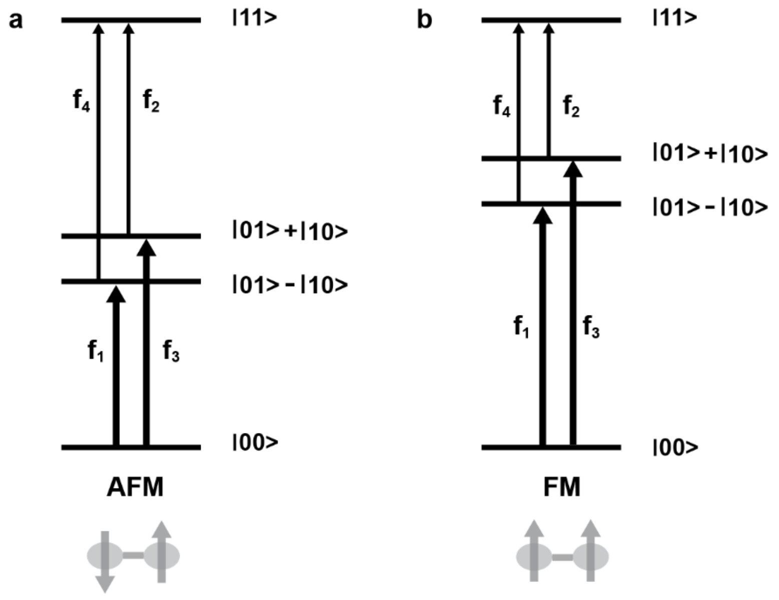

Supplementary Fig. 17. Energy levels of a pair of AFM or FM coupled spin 1/2. The arrows indicate the possible ESR transitions. The frequency and intensity are represented by the length and thickness of the arrows. The peak at lower frequencies (f_1 or f_3) is taller for AFM (a), while it is higher in the FM case (b).

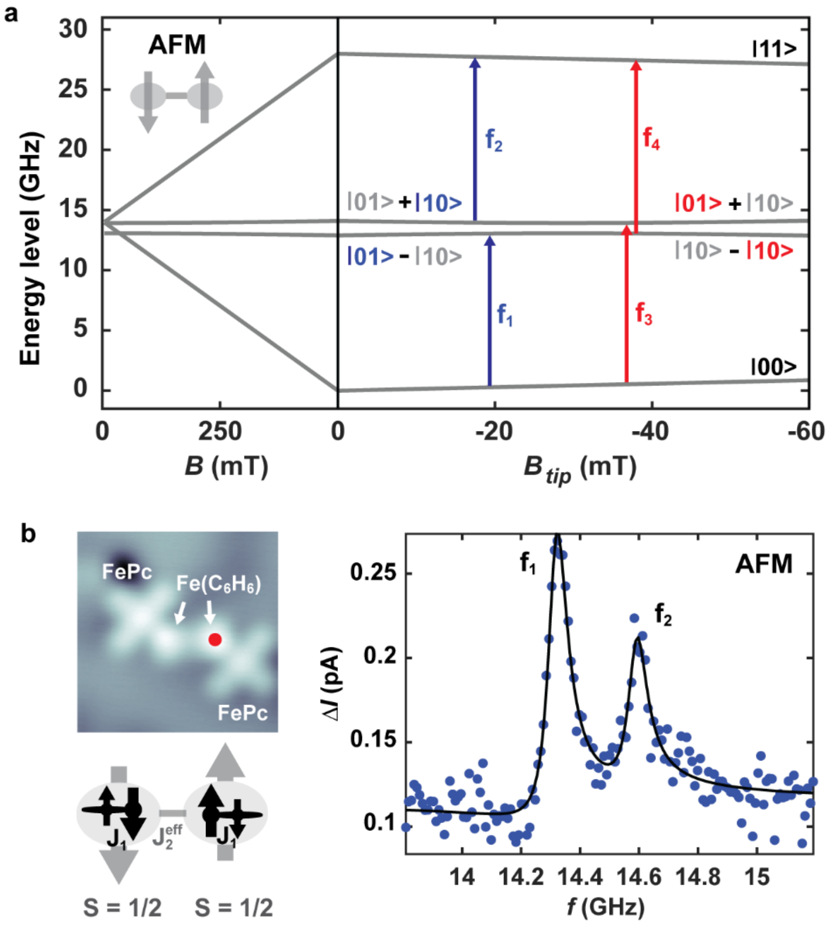

Supplementary Fig. 18. AFM Coupling in a Dimer of Complexes. a. Energy level diagram of two AFM coupled spin ½ in the presence of B and B_{tip}. **b.** Topographic image of two coupled complexes built by atom manipulation ($I = 10\ \mathrm{pA}$, $V_{\mathrm{DC}} = -100\ \mathrm{mV}$, image size: $3\ \mathrm{nm}$). The two Fe atoms in the complexes are closest together. **c.** The ESR spectrum measured on Fe site in the right complex marked by red dot in b. ($V_{\mathrm{DC}} = 60\ \mathrm{mV}$, $V_{\mathrm{rf}} = 12\ \mathrm{mV}$, $B = 484\ \mathrm{mT}$, $B_{\mathrm{tip}} = 34\ \mathrm{mT}$)

The qualitative behavior of the ESR peaks at the point of no detuning $\delta = 0$ can be understood by considering the frequency difference between the second and third ESR resonance, specifically f_2 (f_1) and f_3 (f_4) in AFM (FM) case:

$$f_3 - f_2 = \sqrt{\delta^2 + \left(J_2^{\mathrm{eff}} - D\right)^2} - \left(J_2^{\mathrm{eff}} + 2D\right),$$

$$f_4 - f_1 = \sqrt{\delta^2 + \left(J_2^{\mathrm{eff}} - D\right)^2} + \left(J_2^{\mathrm{eff}} + 2D\right)$$

Since $\left|J_2^{\mathrm{eff}}\right| > D$, we have for $\delta = 0$:

$$\Delta f_2 = f_3 - f_2 = -3D = -3/2D_0 \ \ (J_2^{\mathrm{eff}} > 0, \text{AFM})$$

$$\Delta f_2 = f_4 - f_1 = 3/2D_0 \ \ (J_2^{\text{eff}} < 0, \text{FM})$$

Since $D_0 > 0$, $f_3 > f_2$ for the AFM case for $\delta = 0$, which causes the resonances to cross twice as the tip approaches (Supplementary Fig. 19). In contrast, in the FM case, $f_4 > f_1$, and the two resonances remain separated, as shown in Fig. 4. Thus, crossed or separated transitions at $\delta = 0$ are another indication of AFM and FM coupling, respectively.

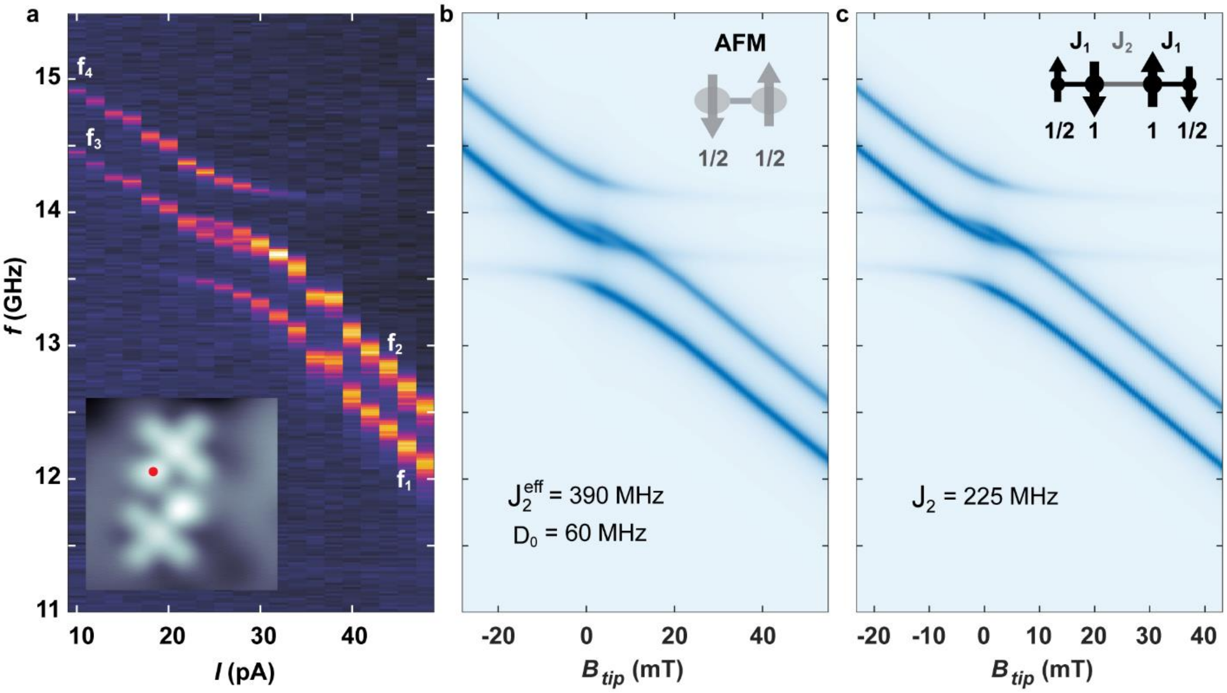

Supplementary Fig. 19. AFM Coupling in a Dimer of Complexes. a, ESR measurements at various tip heights, showing an avoided level crossing at $\delta = 0$ ($V_{\text{DC}} = -60$ mV, $V_{\text{rf}} = 12$ mV, $B = 473$ mT). Inset: topographic image ($I = 10$ pA, $V_{\text{DC}} = -100$ mV, image size: 3 nm × 3 nm). **b,** Simulation of the coupled spin system using a two-spin model (inset) of spins (1/2, 1/2), with $J_2^{\text{eff}} = 390$ MHz, $D_0 = 60$ MHz (corresponding to 1.54 nm, roughly (2,5) MgO lattice) and $B = 495$ mT. **c.** Simulation of the coupled spin system using a four-spin model (inset) of spins (1/2, 1, 1, 1/2) with alternating antiferromagnetic coupling strengths J_1 and J_2. The fitting parameters are: $J_1 = 14.65$ meV, $J_2 = 225$ MHz, $g_{\text{FePc}} = 2$, $g_{\text{Fe}} = 2$, $B = 495$ mT, $T = 1.5\,K$.

We can use the equations of the two spin-model to analyze the data in Fig. 4e and Supplementary Fig. 19a directly: the magnetic dipolar coupling can be determined by $D_0 = \frac{2}{3}|\Delta f_2|$ (extracted directly at $\delta = 0$) while the inter-complex coupling J_2^{eff} can be determined by the ESR splitting $\Delta f_1 = J_2^{\text{eff}} + D_0$. For the FM case in Fig. 4, we obtain $D_0 = 131$ MHz, $J_2^{\text{eff}} = -531$ MHz. For the AFM case in Supplementary Fig. 19, we find $D_0 =$

$60\ \mathrm{MHz}$, $J_2^{\mathrm{eff}} = 390\ \mathrm{MHz}$. The two-spin model is verified through our ESR simulation based on the spin Hamiltonian of two coupled spin ½ (dotted line in Fig. 4e and Supplementary Fig. 19b). In the simulation, the amplitude of the ESR signal is determined by calculating the matrix element of the transitions, with each state thermally populated according to the Boltzmann distribution of its eigenenergy.

To capture minute details in the position and intensity of the peaks, we employ a simulation of the full four-spin system (Fig. 4f and Supplementary Fig. 19c), where the coupling between adjacent spins still remains antiferromagnetic and the magnetic dipolar couplings are calculated based on the distances (Supplementary Fig. 20) between all spin centers. The full four-spin model does not show any significant deviations from the two-spin model for both the FM case discussed in the main text and the AFM case discussed in this section. Only the effective exchange coupling changes of the effective two-spin model compared to a four-spin model, since it now incorporates the different spin structure of the dimers. Thus, we conclude that the complexes can be effectively treated as spin ½ systems and that they can serve as building blocks for larger spin structures.

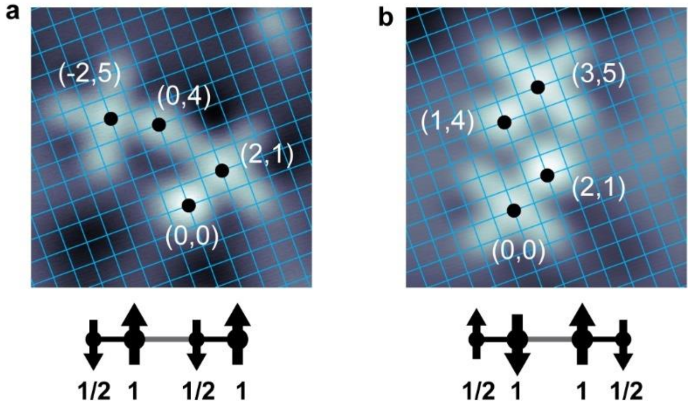

Supplementary Fig. 20. Lattice fit of two coupled complexes. a. Topographic image of two ferromagnetically coupled complexes with MgO lattice overlaid as shown in Fig. 4 in the main text. The distances between spins are used for the four-spin model in Fig. 4f. **b.** Topographic image of two antiferromagnetically coupled complexes with MgO lattice overlaid (Supplementary Fig. 19). The distances between spins are used for the four-spin model in Supplementary Fig. 19c. Both images: The positions of the Fe and molecule spins are labeled by black dots and are defined relative to the oxygen lattice, which is indicated by a blue grid. Size: 3 nm × 3 nm, setpoint: V = -100 mV, I = 20 pA.

We conducted the same ESR measurements on the $Fe(C_6H_6)$ site of the other complex and observed similar behavior (Supplementary Fig. 21). Supplementary Fig. 21a shows data measured on the left complex, similar as in Fig. 4 but with a negative V_{DC}, while Supplementary Fig. 21b displays data measured on the right complex using the same tip and parameters as in Supplementary Fig. 21a. Both exhibit avoided level crossing behavior, with the difference being a frequency shift attributed to variations in the tip magnetic field B_{tip}. Additionally, we attempted measurements on the FePc center; however, detecting ESR signals at FePc center requires a very large B_{tip}, which pushes the system out of the avoided level crossing regime, making the measurements challenging.

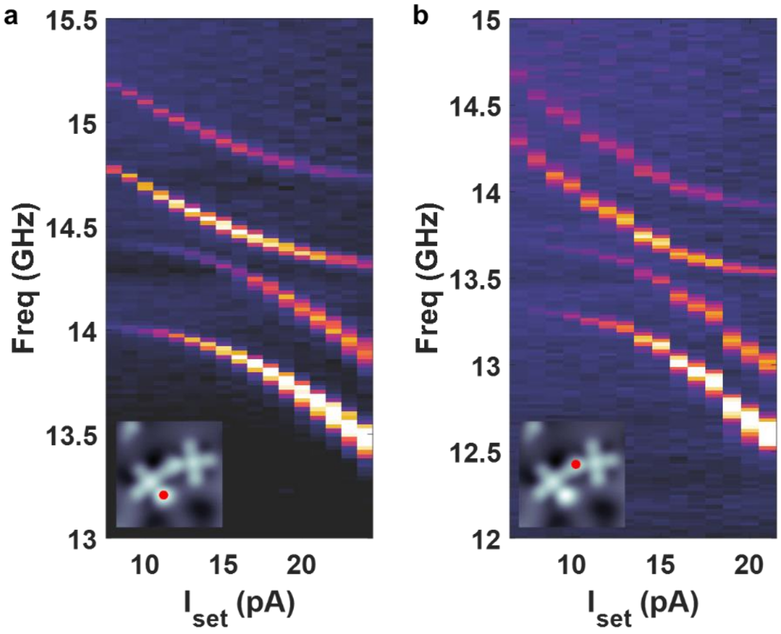

Supplementary Fig. 21. ESR frequency sweep measurements at different setpoint currents (B_{tip}) showing an avoided level crossing in both complexes within a dimer. ESR spectra are recorded at the $Fe(C_6H_6)$ site, indicated by a red dot in the inset: **a.** for the left complex and **b**. for the right complex. ($V_{DC} = -60$ mV, $V_{RF} = 12$ mV, $B = 484\ mT$)

11. Electronic properties of FePc-$Fe(C_6H_6)$ complex on MgO

We measured the dI/dV spectrum of an individual FePc- $Fe(C_6H_6)$ complex on MgO within a large bias range to get the information of orbital states, as shown in Supplementary Fig. 22a. Moreover, we measured topographic images of the complex on

MgO at corresponding energies and then compared with those calculated topographic images, respectively, as shown in Supplementary Fig. 22b. We found good agreement between the experimental topographic images and the calculated ones: At negative bias voltages, we observe a strong feature localized on the Fe(C_6H_6), associated with the states shown in Fig. 1e in the main text. In contrast, the highest occupied molecular orbitals (HOMO) of FePc only appear at around -2 V[2]. At positive bias voltages, we mostly observe the lowest unoccupied molecular orbital (LUMO) of FePc[2]. The limitations of the bias range measured is limited by the stability of the complex, which destabilizes at high voltages.

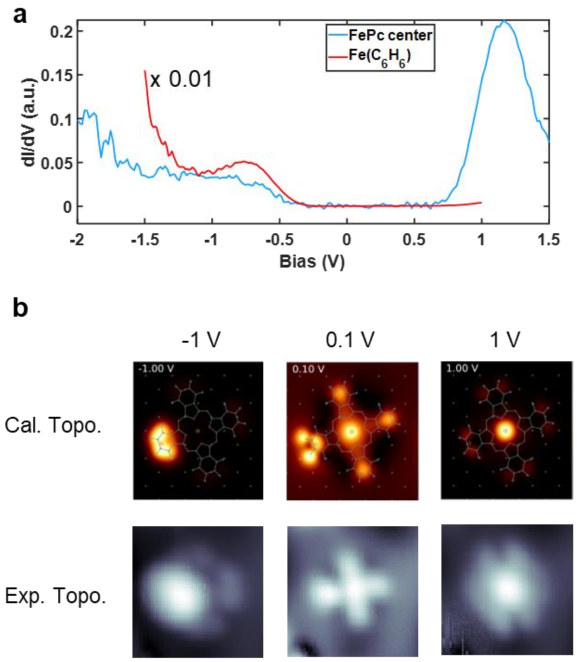

Supplementary Fig. 22. Comparison of the experimental and DFT calculated topographic of FePc-Fe(C6H6) on MgO/Ag. a. Large-range dI/dV spectrum at the FePc center and Fe(C_6H_6) site (setpoint: V = 1.5 V, I = 5 pA). **b.** Upper panel: the DFT-calculated STM images at different biases. calculated in the Tersoff-Hamann approximation. Lower panel: the experimental STM images measured at different biases (constant-current mode, I = 5 pA) corresponding to HOMO, in-gap and LUMO energy, respectively.

Supplementary References

1 Baumann, S. *et al.* Origin of Perpendicular Magnetic Anisotropy and Large Orbital Moment in Fe Atoms on MgO. *Physical Review Letters* **115**, 237202 (2015). https://doi.org/10.1103/PhysRevLett.115.237202

2 Zhang, X. *et al.* Electron spin resonance of single iron phthalocyanine molecules and role of their non-localized spins in magnetic interactions. *Nature Chemistry* **14**, 59-65 (2022). https://doi.org/10.1038/s41557-021-00827-7
3 Čenčariková, H. & Strečka, J. Unconventional strengthening of the bipartite entanglement of a mixed spin-(1/2,1) Heisenberg dimer achieved through Zeeman splitting. *Physical Review B* **102**, 184419 (2020). https://doi.org/10.1103/PhysRevB.102.184419
4 Loth, S., Lutz, C. P. & Heinrich, A. J. Spin-polarized spin excitation spectroscopy. *New Journal of Physics* **12**, 125021 (2010). https://doi.org/10.1088/1367-2630/12/12/125021
5 Ternes, M. Spin excitations and correlations in scanning tunneling spectroscopy. *New Journal of Physics* **17**, 063016 (2015).
6 Baumann, S. *et al.* Electron paramagnetic resonance of individual atoms on a surface. *Science* **350**, 417-420 (2015). https://doi.org/doi:10.1126/science.aac8703
7 Ternes, M. Probing magnetic excitations and correlations in single and coupled spin systems with scanning tunneling spectroscopy. *Progress in Surface Science* **92**, 83-115 (2017). https://doi.org/https://doi.org/10.1016/j.progsurf.2017.01.001
8 Peres, A. Separability Criterion for Density Matrices. *Physical Review Letters* **77**, 1413-1415 (1996). https://doi.org/10.1103/PhysRevLett.77.1413
9 Choi, T. *et al.* Atomic-scale sensing of the magnetic dipolar field from single atoms. *Nature Nanotechnology* **12**, 420-424 (2017). https://doi.org/10.1038/nnano.2017.18
10 Willke, P. *et al.* Tuning Single-Atom Electron Spin Resonance in a Vector Magnetic Field. *Nano Letters* **19**, 8201-8206 (2019). https://doi.org/10.1021/acs.nanolett.9b03559
11 Singha, A. *et al.* Engineering atomic-scale magnetic fields by dysprosium single atom magnets. *Nature Communications* **12**, 4179 (2021). https://doi.org/10.1038/s41467-021-24465-2
12 Crabtree, R. H. *The Organometallic Chemistry of the Transition Metals*. (John Wiley & Sons, 2009).
13 Wolf, C., Delgado, F., Reina, J. & Lorente, N. Efficient Ab Initio Multiplet Calculations for Magnetic Adatoms on MgO. *The Journal of Physical Chemistry A* **124**, 2318-2327 (2020). https://doi.org/10.1021/acs.jpca.9b10749
14 Noodleman, L. Valence bond description of antiferromagnetic coupling in transition metal dimers. *The Journal of Chemical Physics* **74**, 5737-5743 (1981). https://doi.org/10.1063/1.440939
15 Zhang, X. *et al.* Influence of the Magnetic Tip on Heterodimers in Electron Spin Resonance Combined with Scanning Tunneling Microscopy. *ACS Nano* **17**, 16935-16942 (2023). https://doi.org/10.1021/acsnano.3c04024
16 Seifert, T. S., Kovarik, S., Gambardella, P. & Stepanow, S. Accurate measurement of atomic magnetic moments by minimizing the tip magnetic field in STM-based electron paramagnetic resonance. *Physical Review Research* **3**, 043185 (2021). https://doi.org/10.1103/PhysRevResearch.3.043185
17 Willke, P. *et al.* Coherent Spin Control of Single Molecules on a Surface. *ACS Nano* **15**, 17959-17965 (2021). https://doi.org/10.1021/acsnano.1c06394
18 Yang, K. *et al.* Coherent spin manipulation of individual atoms on a surface. *Science* **366**, 509-512 (2019). https://doi.org/doi:10.1126/science.aay6779
19 Phark, S.-h. *et al.* Electric-Field-Driven Spin Resonance by On-Surface Exchange Coupling to a Single-Atom Magnet. *Advanced Science* **10**, 2302033 (2023). https://doi.org/https://doi.org/10.1002/advs.202302033
20 Lado, J. L., Ferrón, A. & Fernández-Rossier, J. Exchange mechanism for electron paramagnetic resonance of individual adatoms. *Physical Review B* **96**, 205420 (2017). https://doi.org/10.1103/PhysRevB.96.205420

21 Delgado, F. & Lorente, N. A theoretical review on the single-impurity electron spin resonance on surfaces. *Progress in Surface Science* **96**, 100625 (2021). https://doi.org/https://doi.org/10.1016/j.progsurf.2021.100625
22 Reina-Gálvez, J. *et al.* Efficient driving of a spin-qubit using single-atom magnets. *arXiv preprint arXiv:2408.07289* (2024).
23 Chen, Y., Bae, Y. & Heinrich, A. J. Harnessing the Quantum Behavior of Spins on Surfaces. *Advanced Materials* **35**, 2107534 (2023). https://doi.org/https://doi.org/10.1002/adma.202107534
24 Reina-Gálvez, J., Wolf, C. & Lorente, N. Many-body nonequilibrium effects in all-electric electron spin resonance. *Physical Review B* **107**, 235404 (2023). https://doi.org/10.1103/PhysRevB.107.235404
25 Paul, W. *et al.* Control of the millisecond spin lifetime of an electrically probed atom. *Nature Physics* **13**, 403-407 (2017). https://doi.org/10.1038/nphys3965
26 Veldman, L. M. *et al.* Free coherent evolution of a coupled atomic spin system initialized by electron scattering. *Science* **372**, 964-968 (2021). https://doi.org/doi:10.1126/science.abg8223
