# Supplementary figures and images for: Quantum spin-engineering in on-surface molecular ferrimagnets (part 2 of 2)
Source: Nat Commun. 2025 Jun 5;16:5208. doi: 10.1038/s41467-025-60409-w (PMC12137798; doi:10.1038/s41467-025-60409-w)

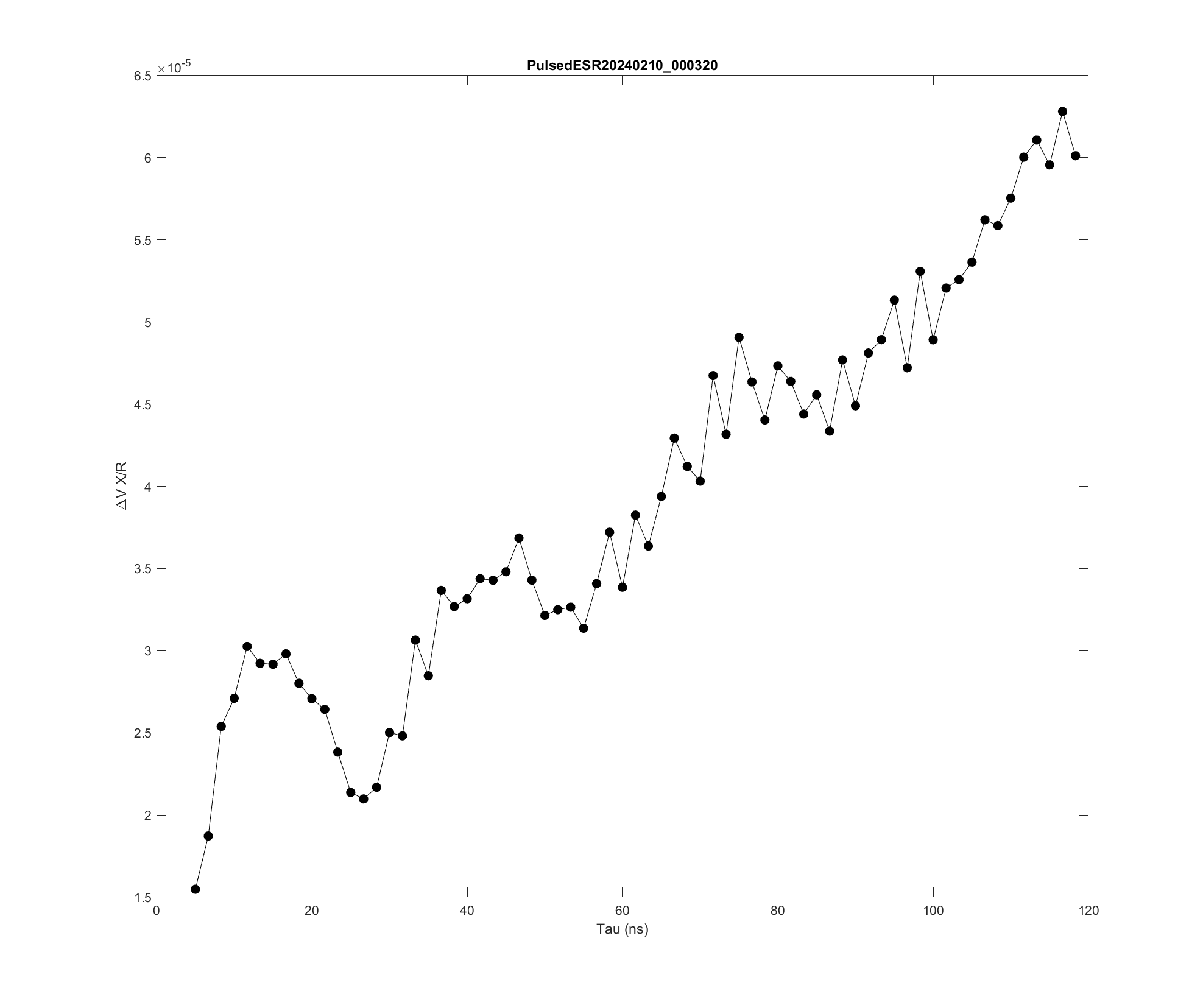

Supplement: Supplementary file 3 — Source Data [file 41467_2025_60409_MOESM3_ESM.zip › SupplementaryData1/Figure3/Fig3c/PulsedESR20240210_000320.png]

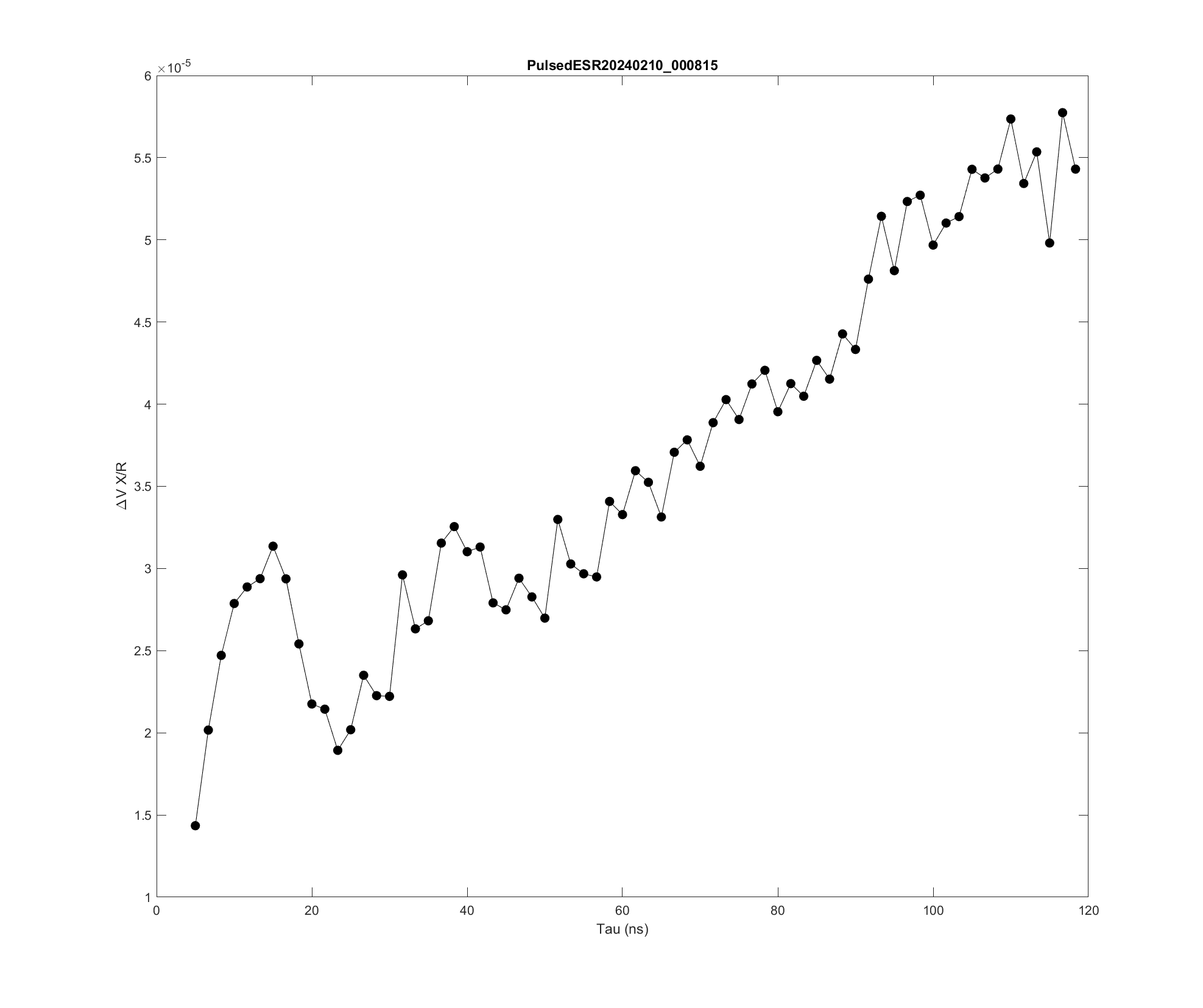

Supplement: Supplementary file 3 — Source Data [file 41467_2025_60409_MOESM3_ESM.zip › SupplementaryData1/Figure3/Fig3c/PulsedESR20240210_000815.png]

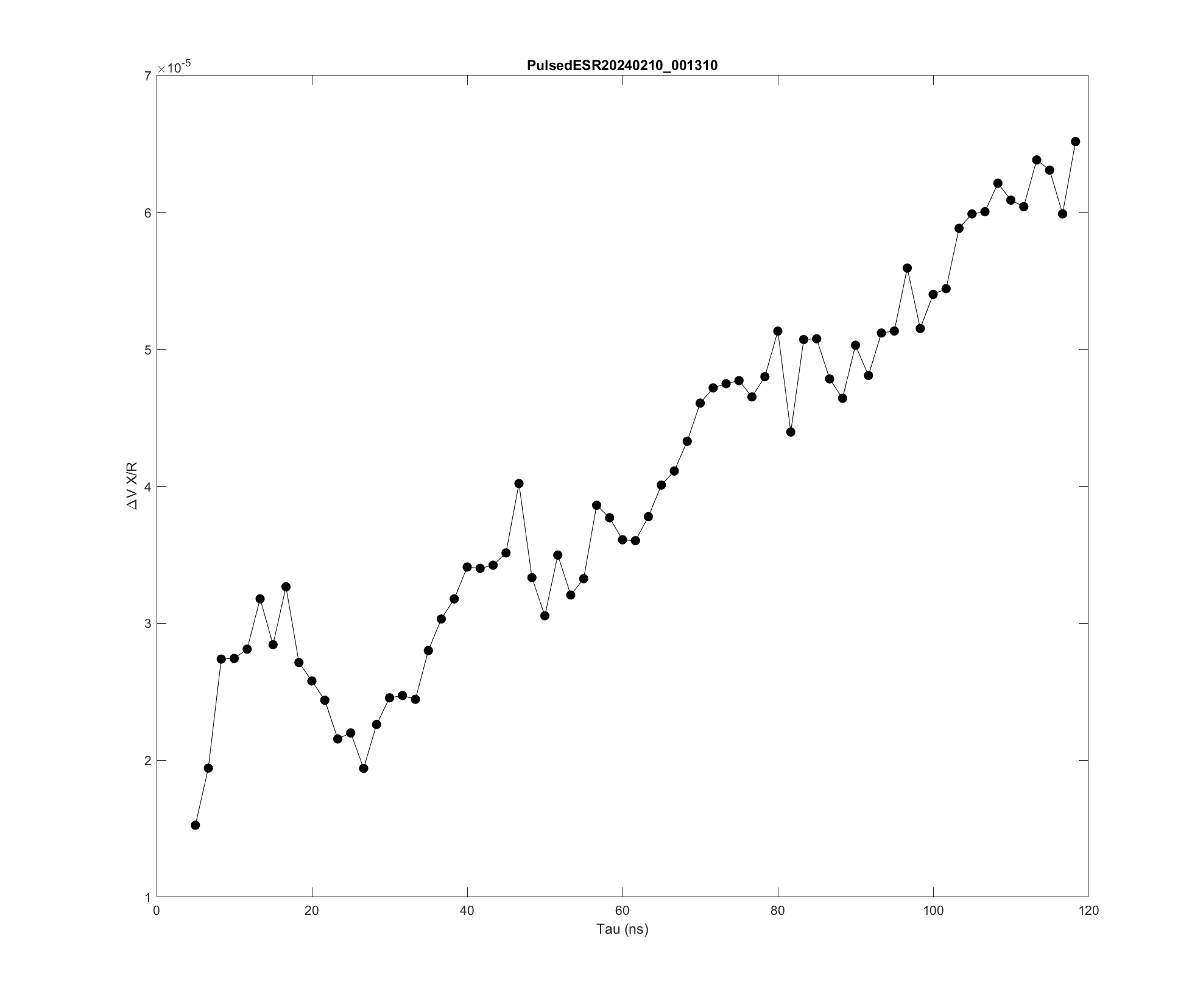

Supplement: Supplementary file 3 — Source Data [file 41467_2025_60409_MOESM3_ESM.zip › SupplementaryData1/Figure3/Fig3c/PulsedESR20240210_001310.png]

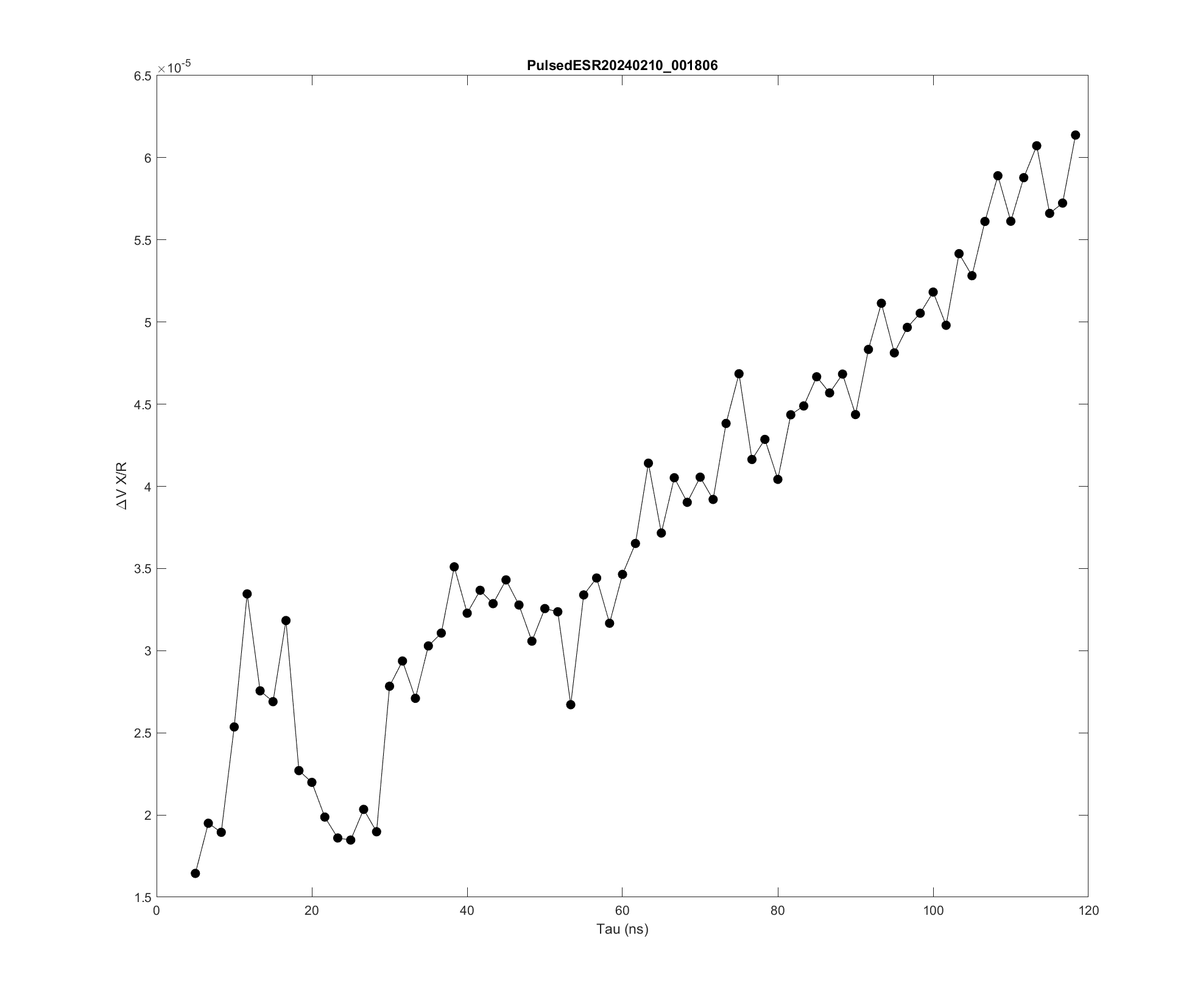

Supplement: Supplementary file 3 — Source Data [file 41467_2025_60409_MOESM3_ESM.zip › SupplementaryData1/Figure3/Fig3c/PulsedESR20240210_001806.png]

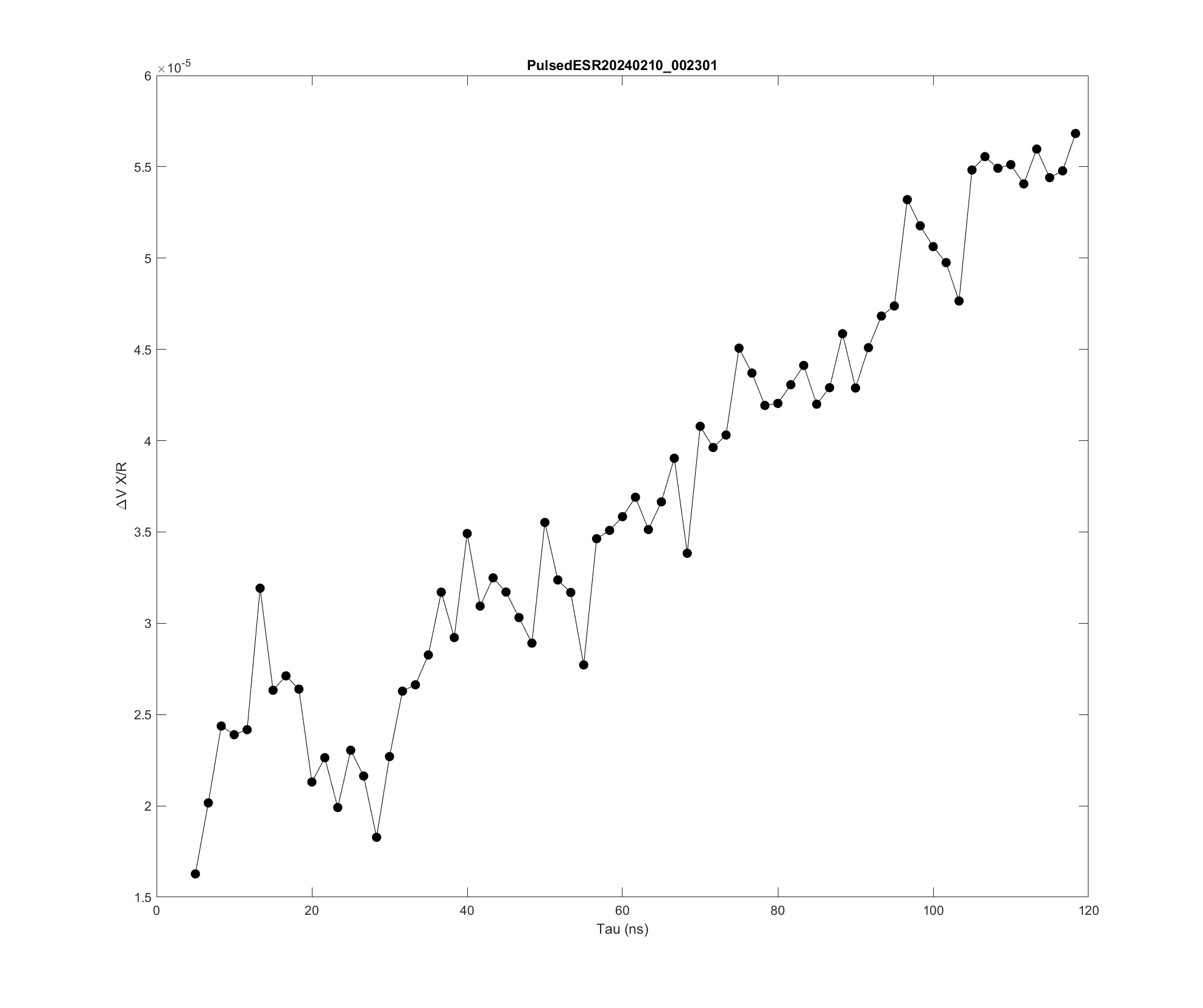

Supplement: Supplementary file 3 — Source Data [file 41467_2025_60409_MOESM3_ESM.zip › SupplementaryData1/Figure3/Fig3c/PulsedESR20240210_002301.png]

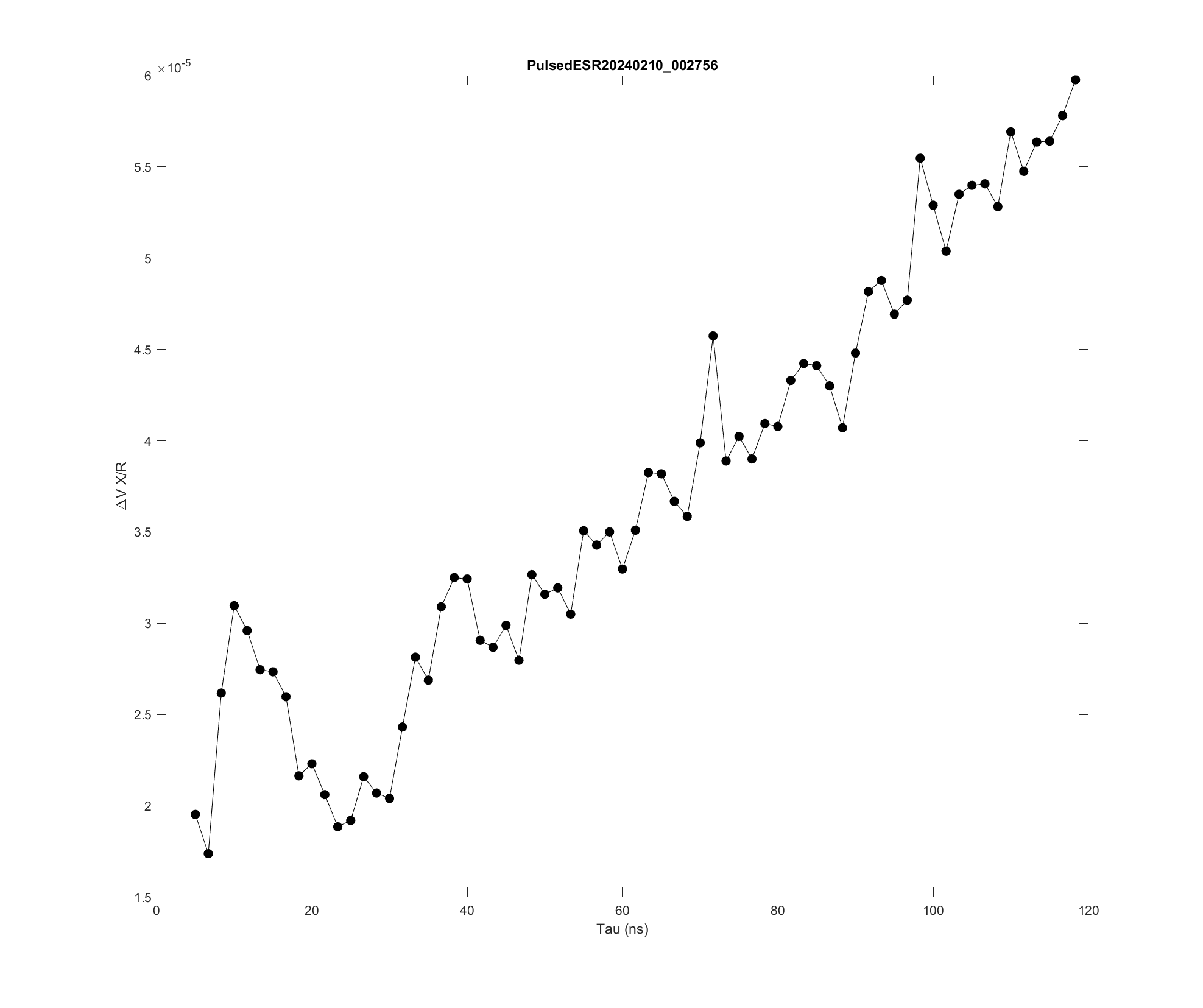

Supplement: Supplementary file 3 — Source Data [file 41467_2025_60409_MOESM3_ESM.zip › SupplementaryData1/Figure3/Fig3c/PulsedESR20240210_002756.png]

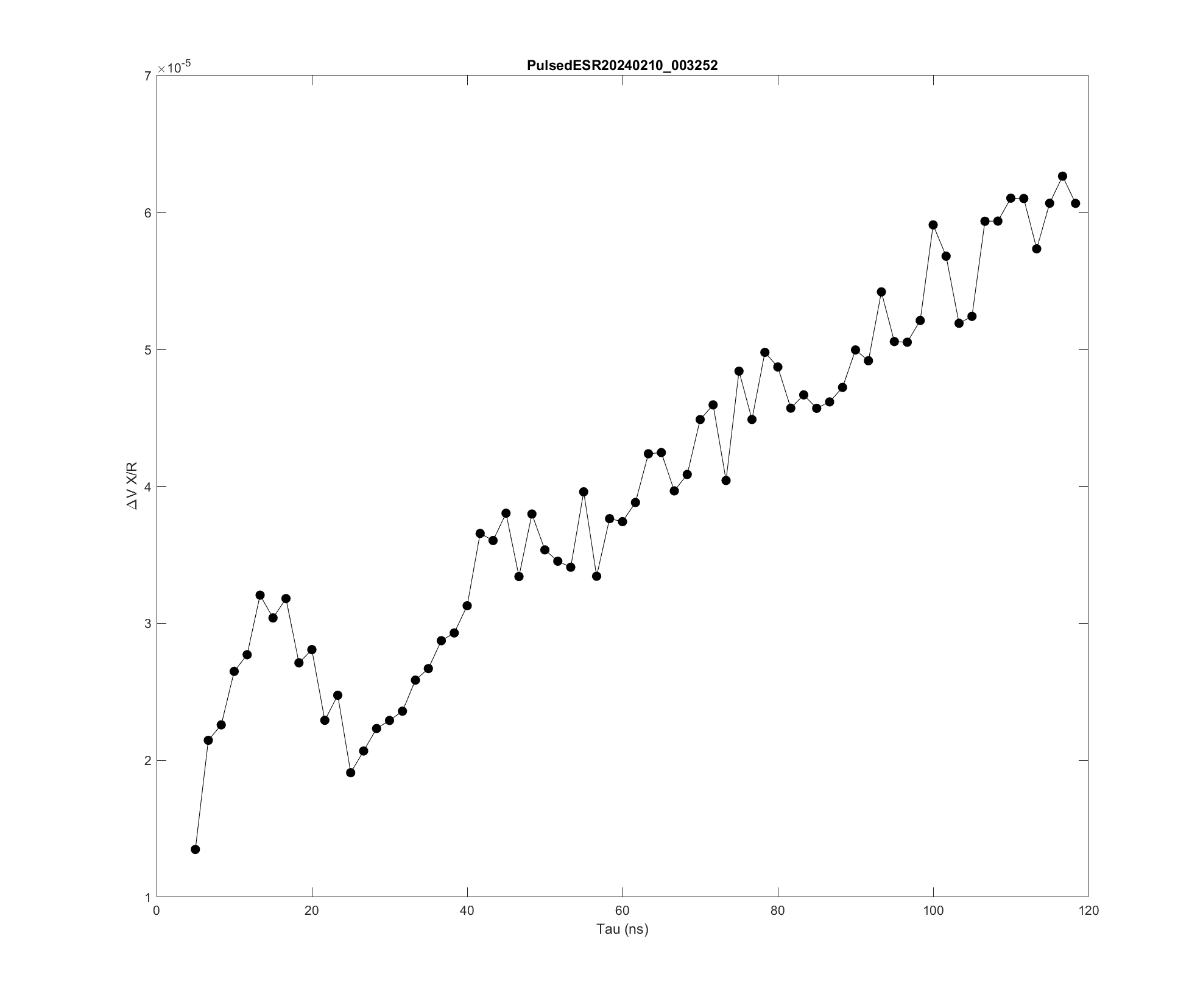

Supplement: Supplementary file 3 — Source Data [file 41467_2025_60409_MOESM3_ESM.zip › SupplementaryData1/Figure3/Fig3c/PulsedESR20240210_003252.png]

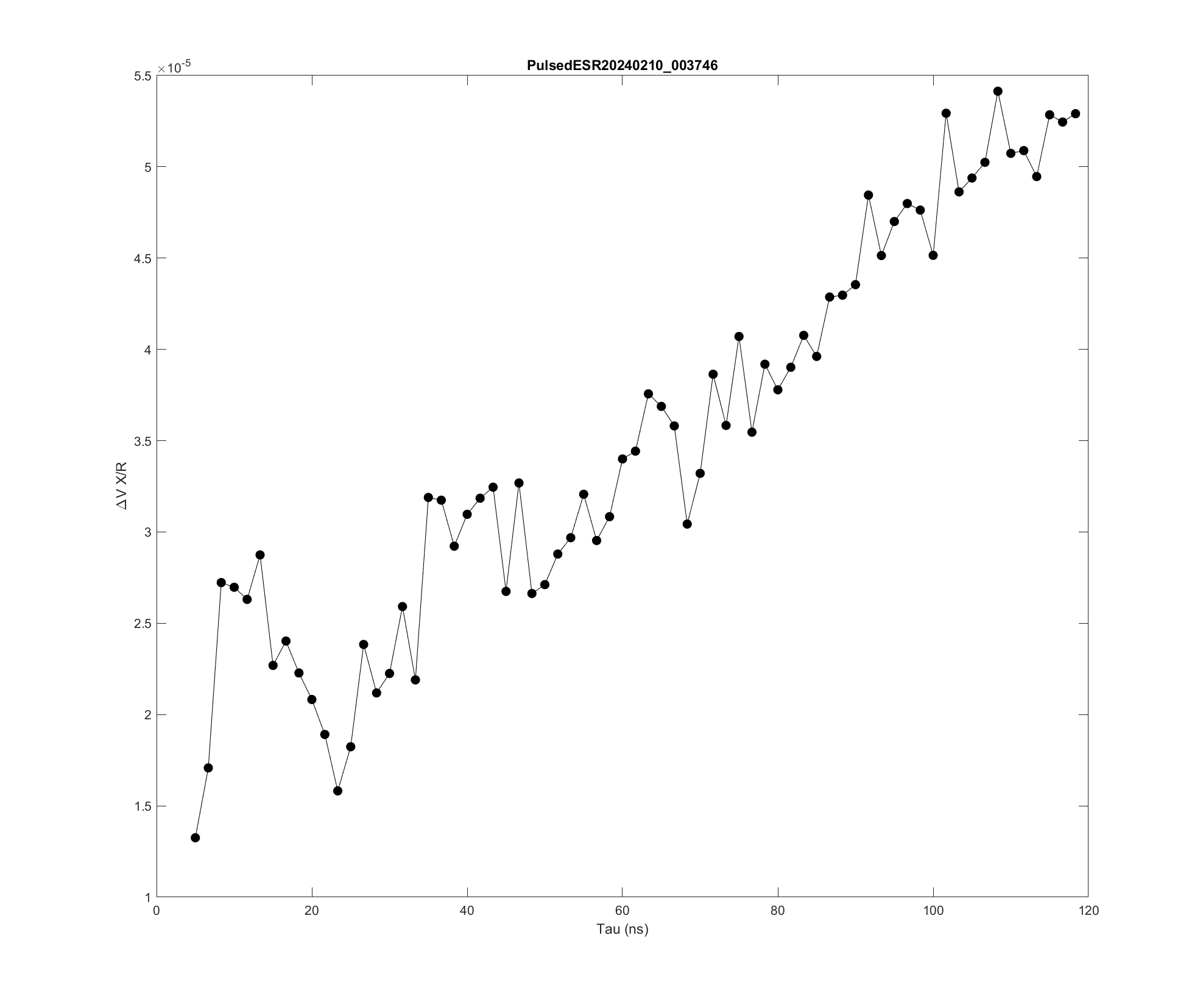

Supplement: Supplementary file 3 — Source Data [file 41467_2025_60409_MOESM3_ESM.zip › SupplementaryData1/Figure3/Fig3c/PulsedESR20240210_003746.png]

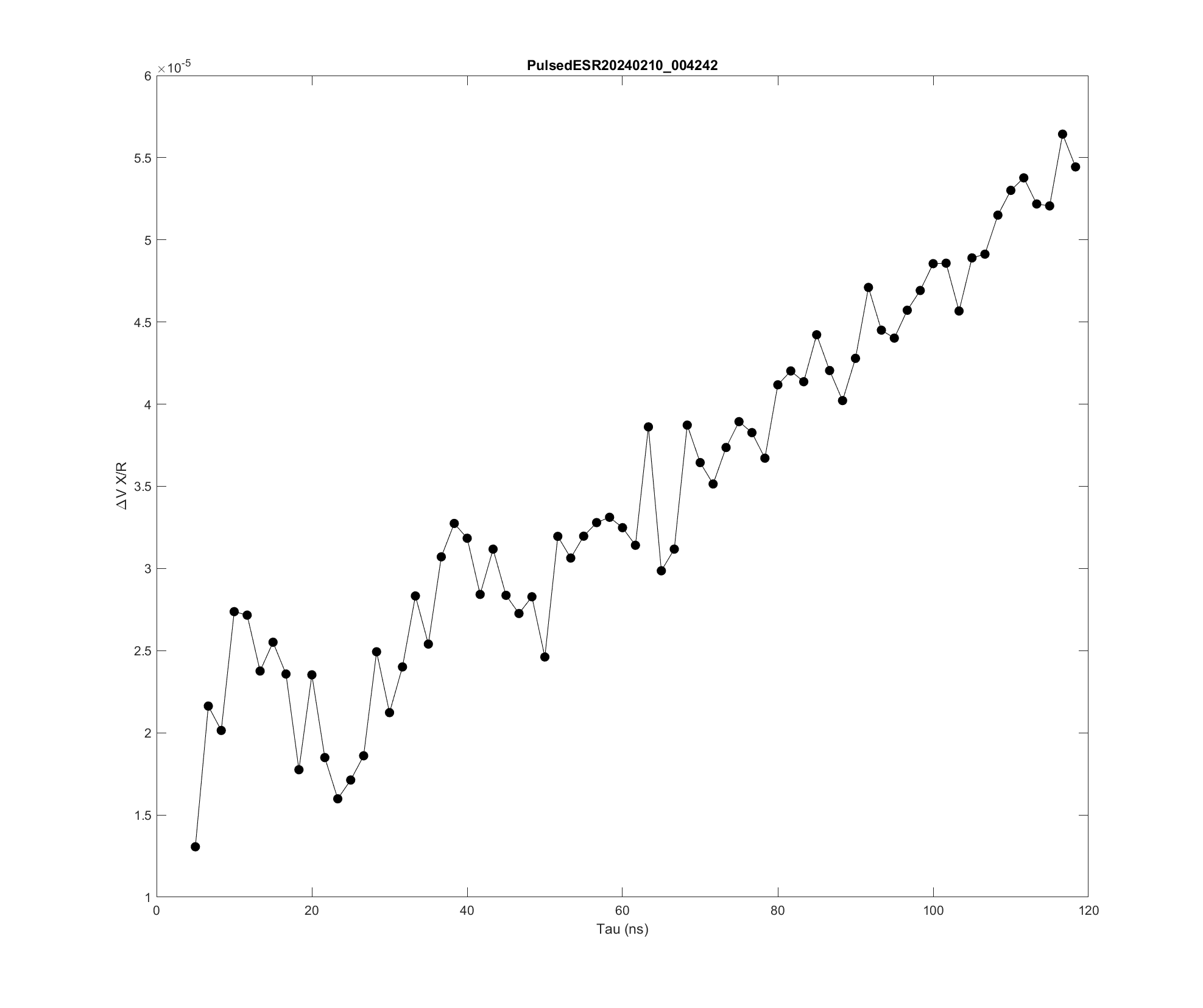

Supplement: Supplementary file 3 — Source Data [file 41467_2025_60409_MOESM3_ESM.zip › SupplementaryData1/Figure3/Fig3c/PulsedESR20240210_004242.png]

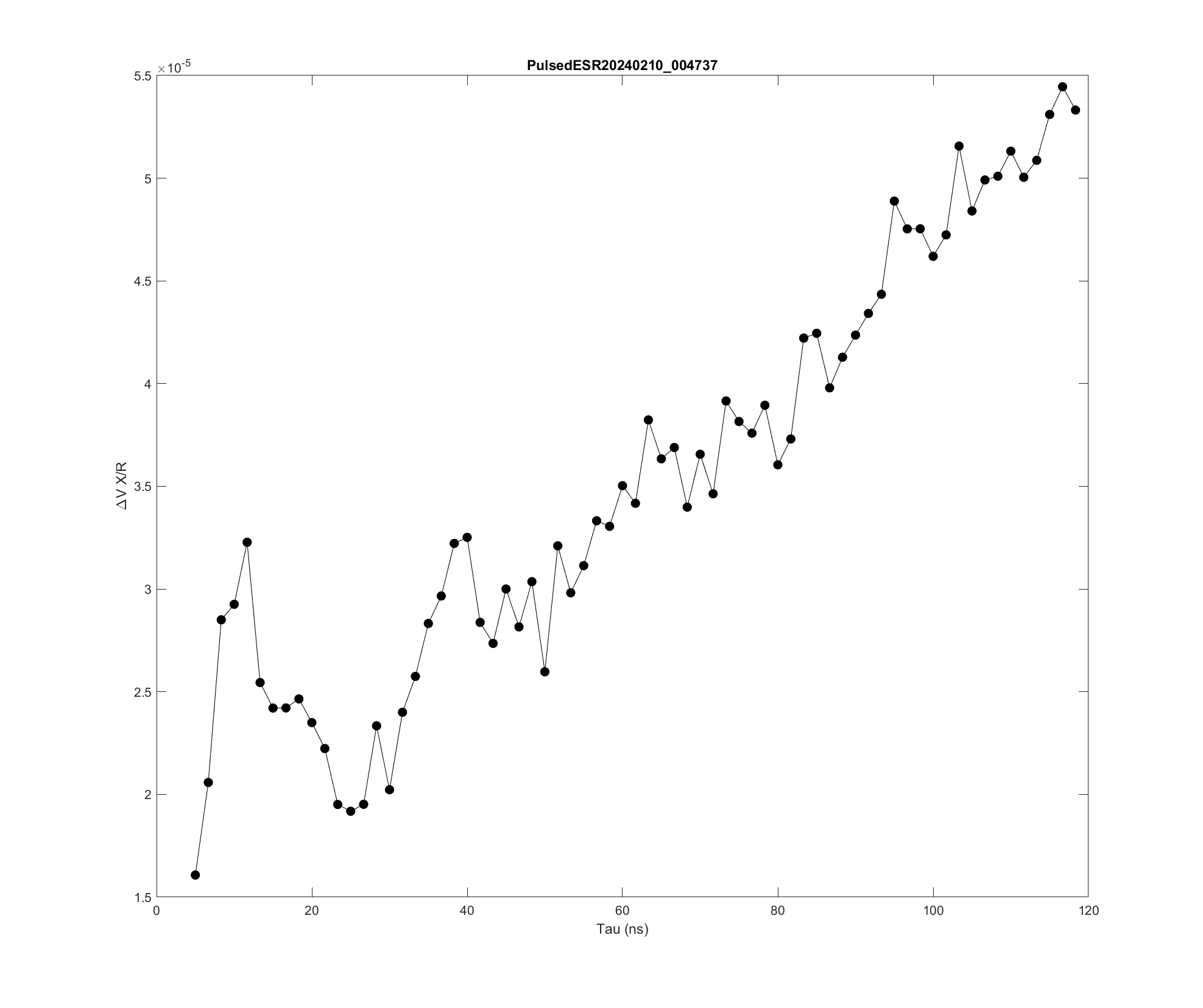

Supplement: Supplementary file 3 — Source Data [file 41467_2025_60409_MOESM3_ESM.zip › SupplementaryData1/Figure3/Fig3c/PulsedESR20240210_004737.png]

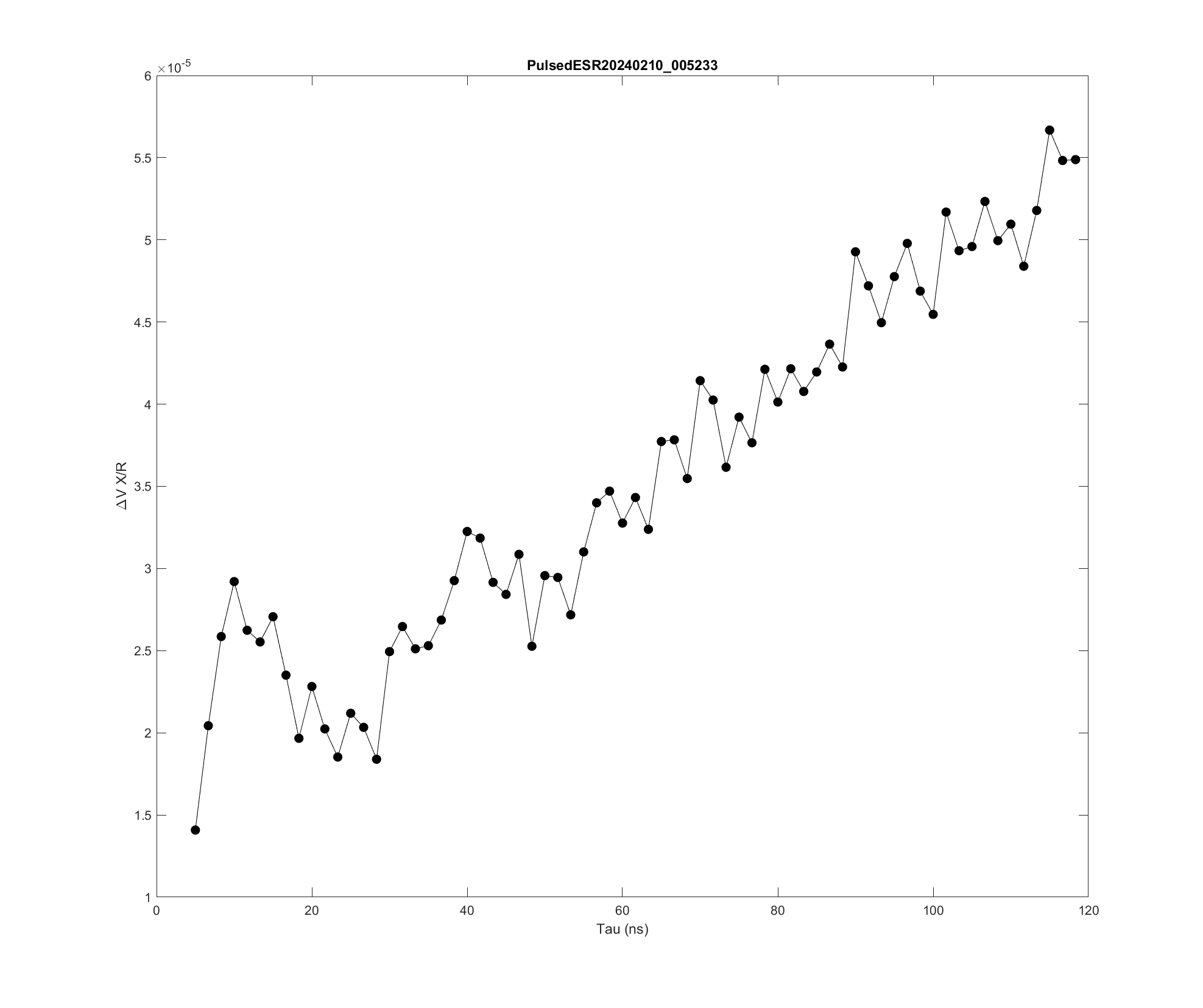

Supplement: Supplementary file 3 — Source Data [file 41467_2025_60409_MOESM3_ESM.zip › SupplementaryData1/Figure3/Fig3c/PulsedESR20240210_005233.png]

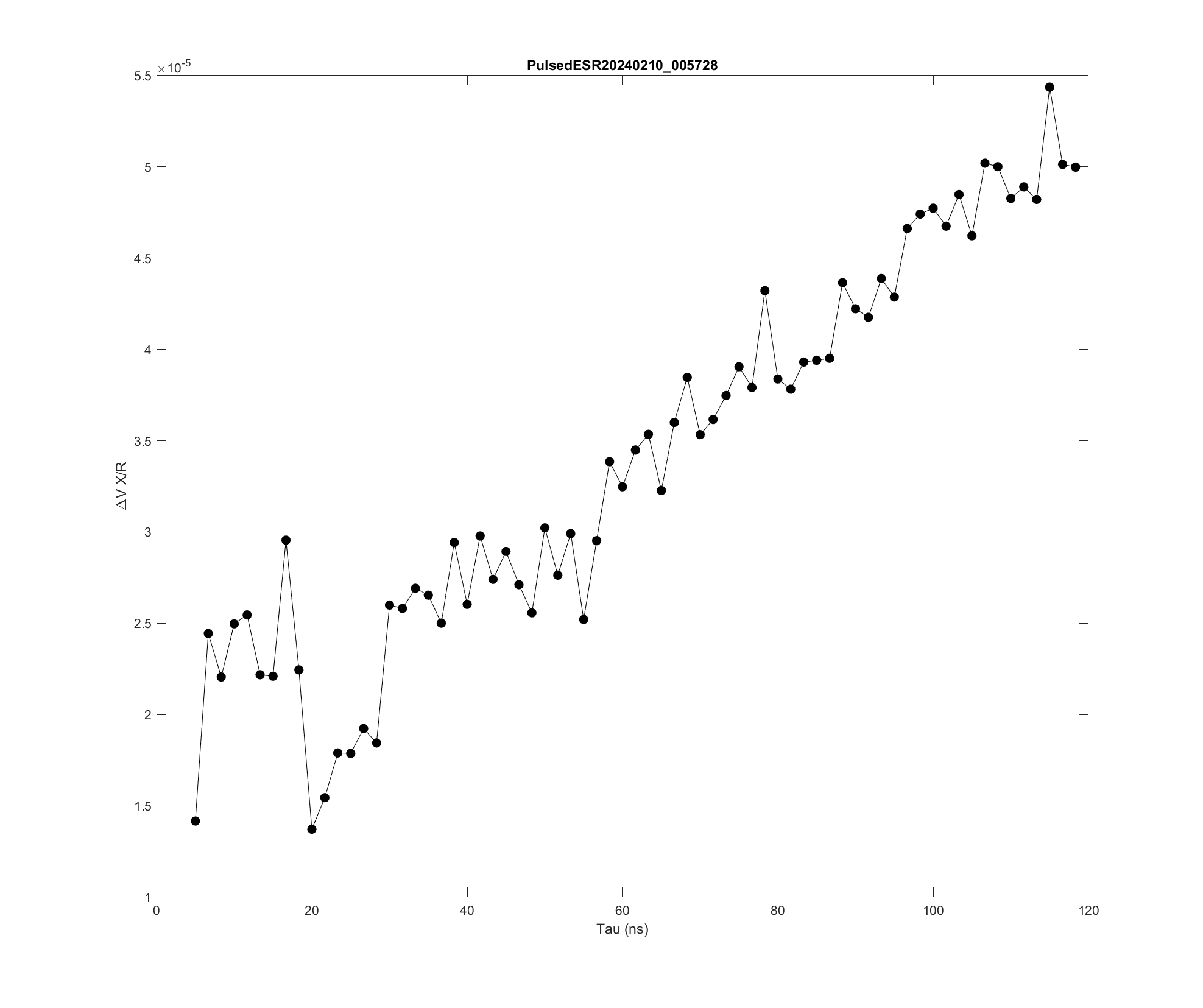

Supplement: Supplementary file 3 — Source Data [file 41467_2025_60409_MOESM3_ESM.zip › SupplementaryData1/Figure3/Fig3c/PulsedESR20240210_005728.png]

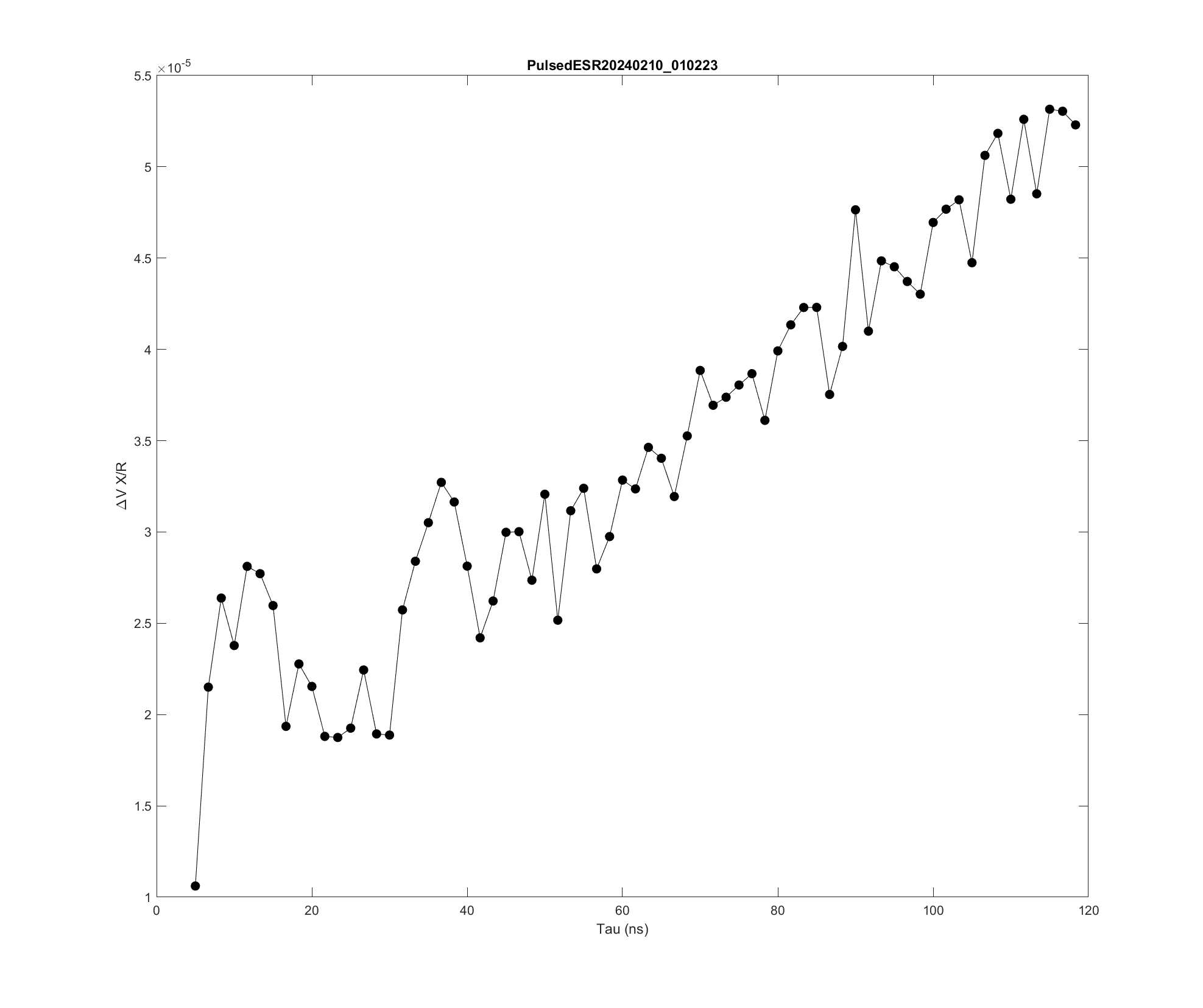

Supplement: Supplementary file 3 — Source Data [file 41467_2025_60409_MOESM3_ESM.zip › SupplementaryData1/Figure3/Fig3c/PulsedESR20240210_010223.png]

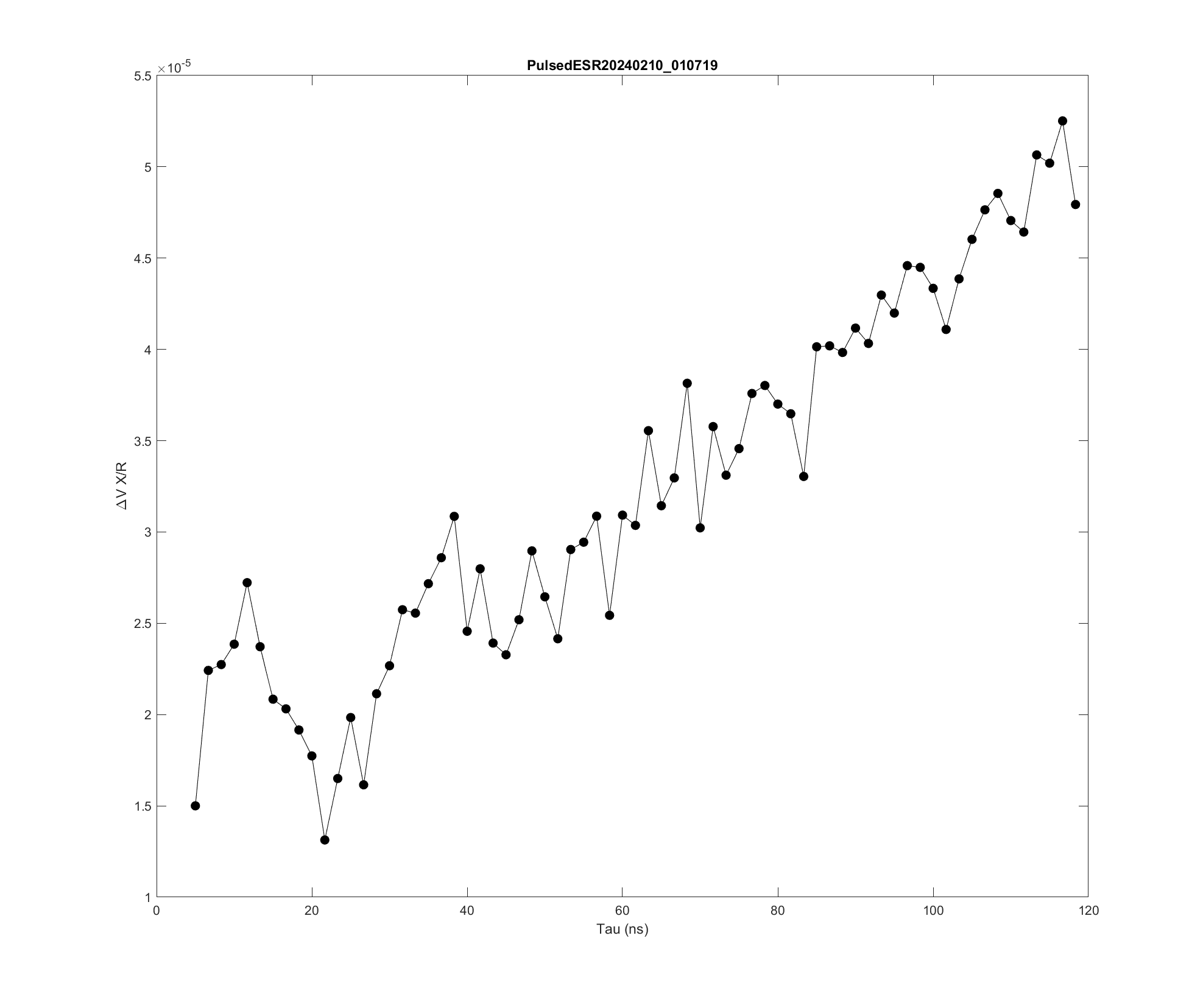

Supplement: Supplementary file 3 — Source Data [file 41467_2025_60409_MOESM3_ESM.zip › SupplementaryData1/Figure3/Fig3c/PulsedESR20240210_010719.png]

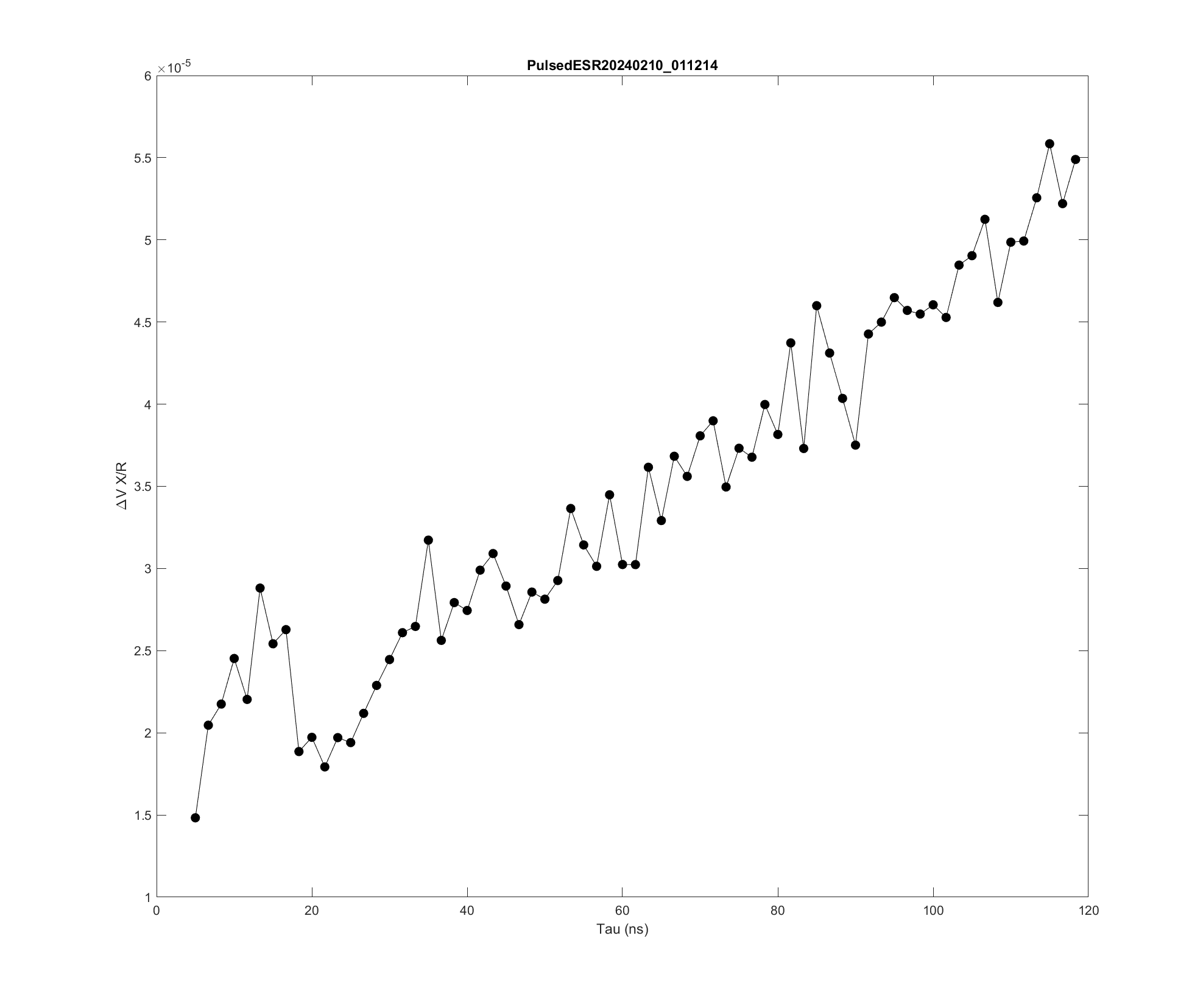

Supplement: Supplementary file 3 — Source Data [file 41467_2025_60409_MOESM3_ESM.zip › SupplementaryData1/Figure3/Fig3c/PulsedESR20240210_011214.png]

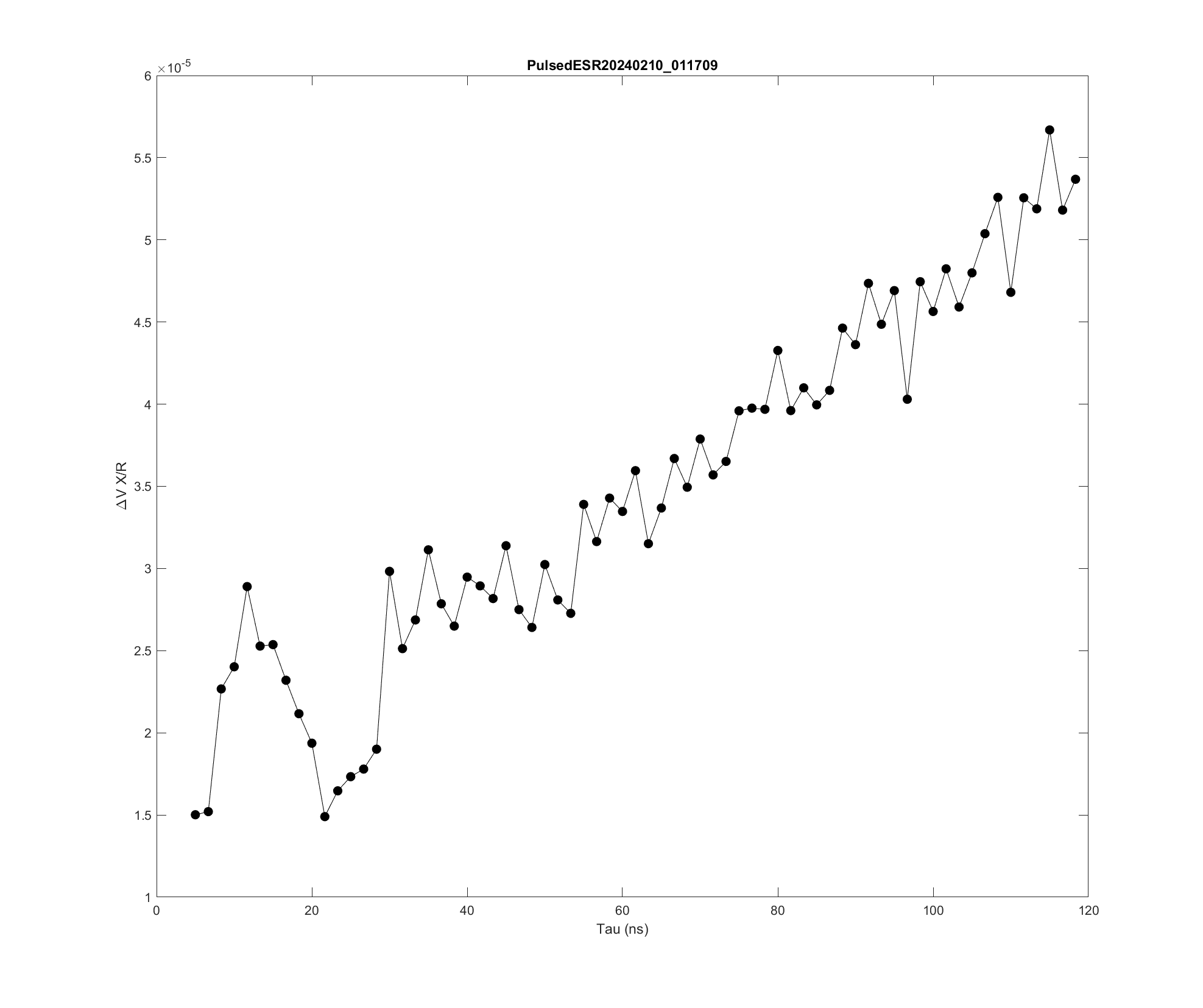

Supplement: Supplementary file 3 — Source Data [file 41467_2025_60409_MOESM3_ESM.zip › SupplementaryData1/Figure3/Fig3c/PulsedESR20240210_011709.png]

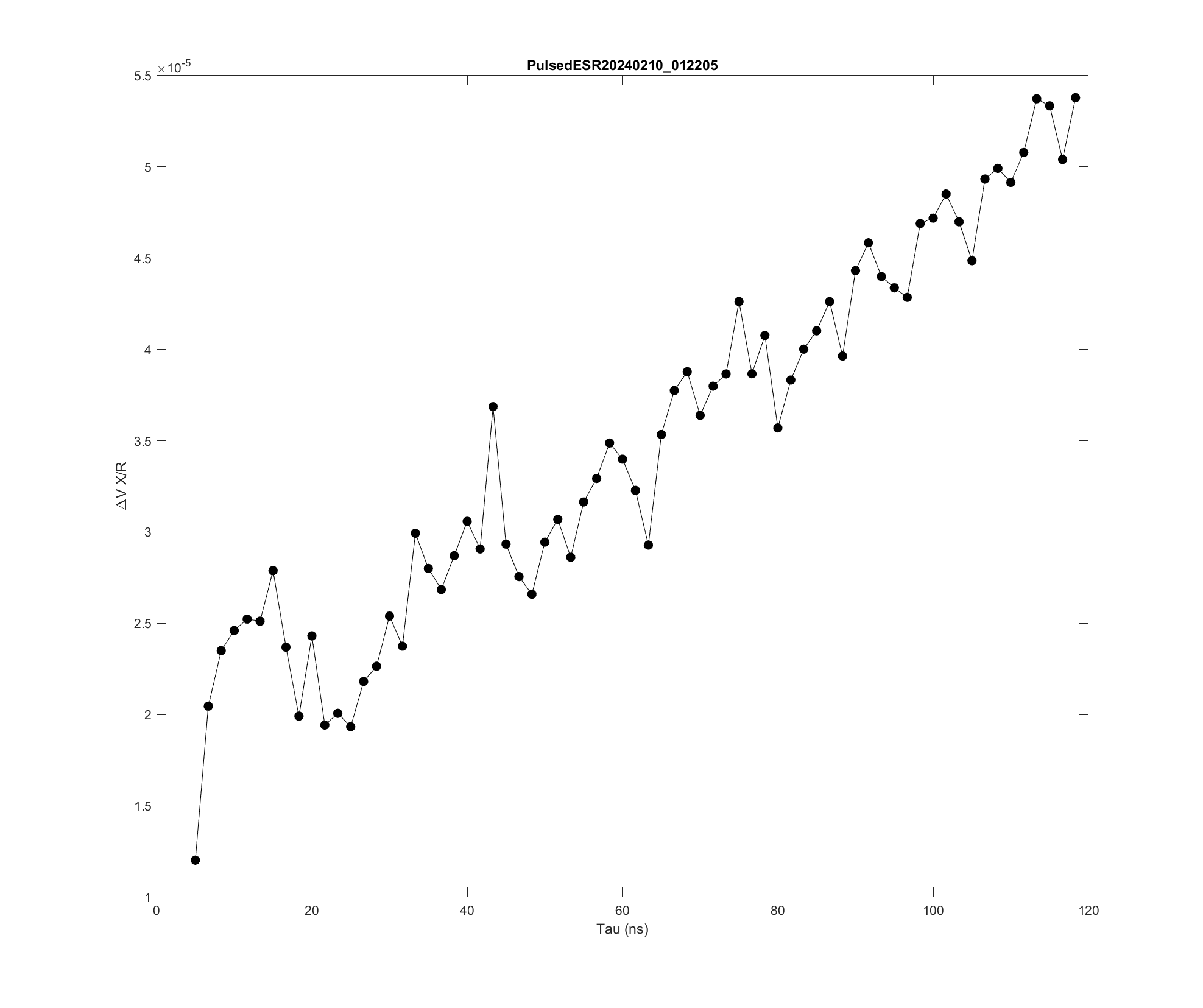

Supplement: Supplementary file 3 — Source Data [file 41467_2025_60409_MOESM3_ESM.zip › SupplementaryData1/Figure3/Fig3c/PulsedESR20240210_012205.png]

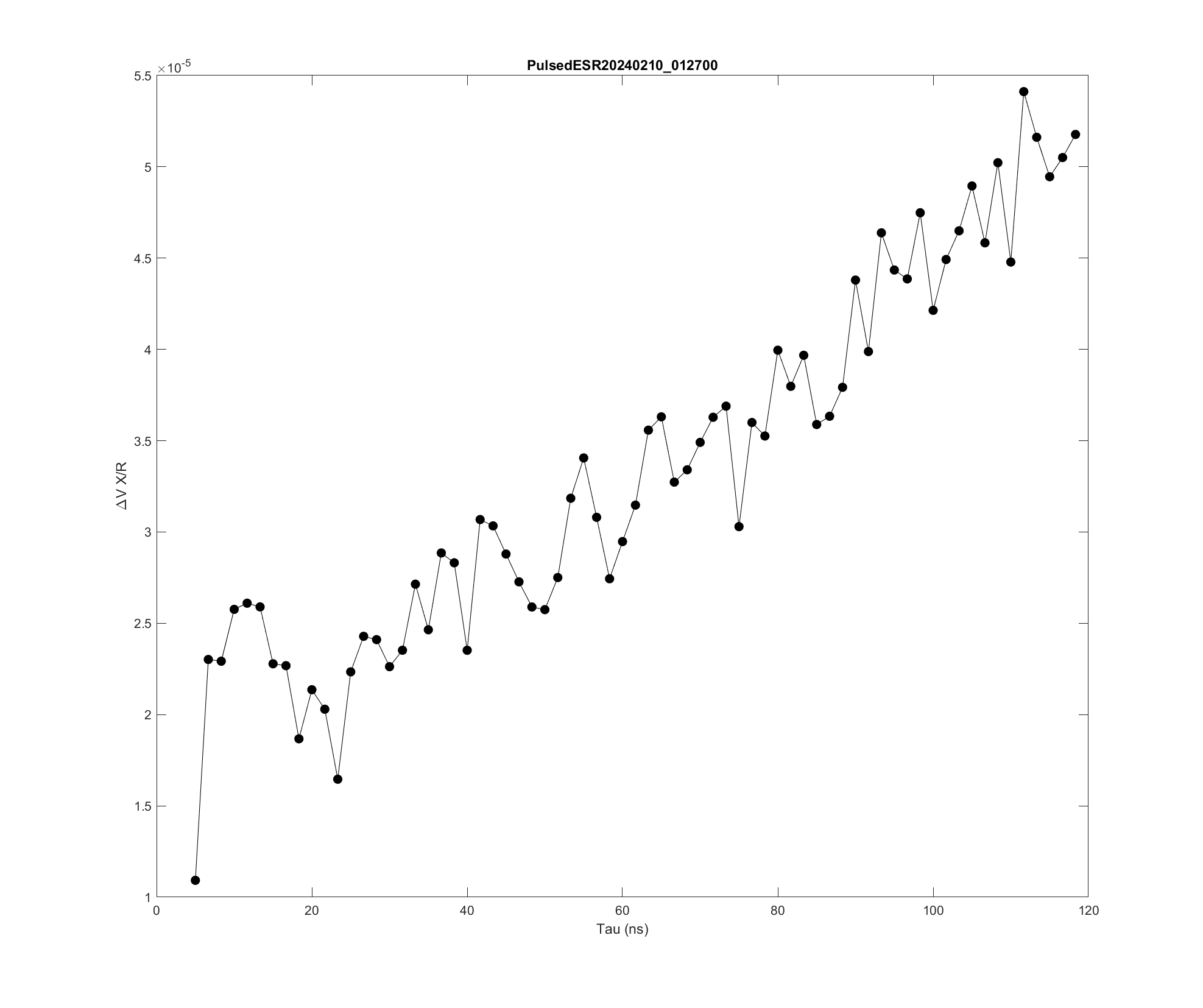

Supplement: Supplementary file 3 — Source Data [file 41467_2025_60409_MOESM3_ESM.zip › SupplementaryData1/Figure3/Fig3c/PulsedESR20240210_012700.png]

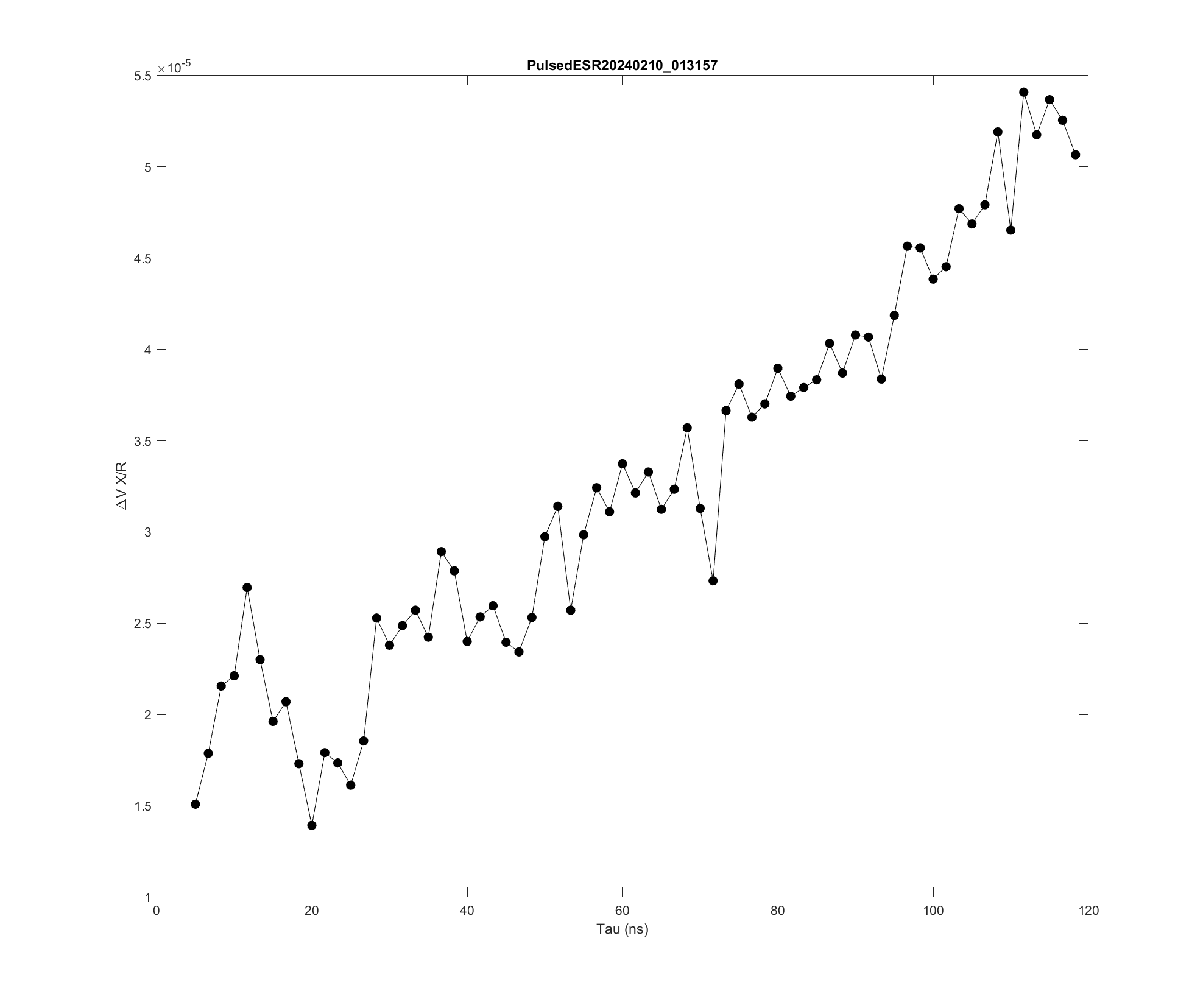

Supplement: Supplementary file 3 — Source Data [file 41467_2025_60409_MOESM3_ESM.zip › SupplementaryData1/Figure3/Fig3c/PulsedESR20240210_013157.png]

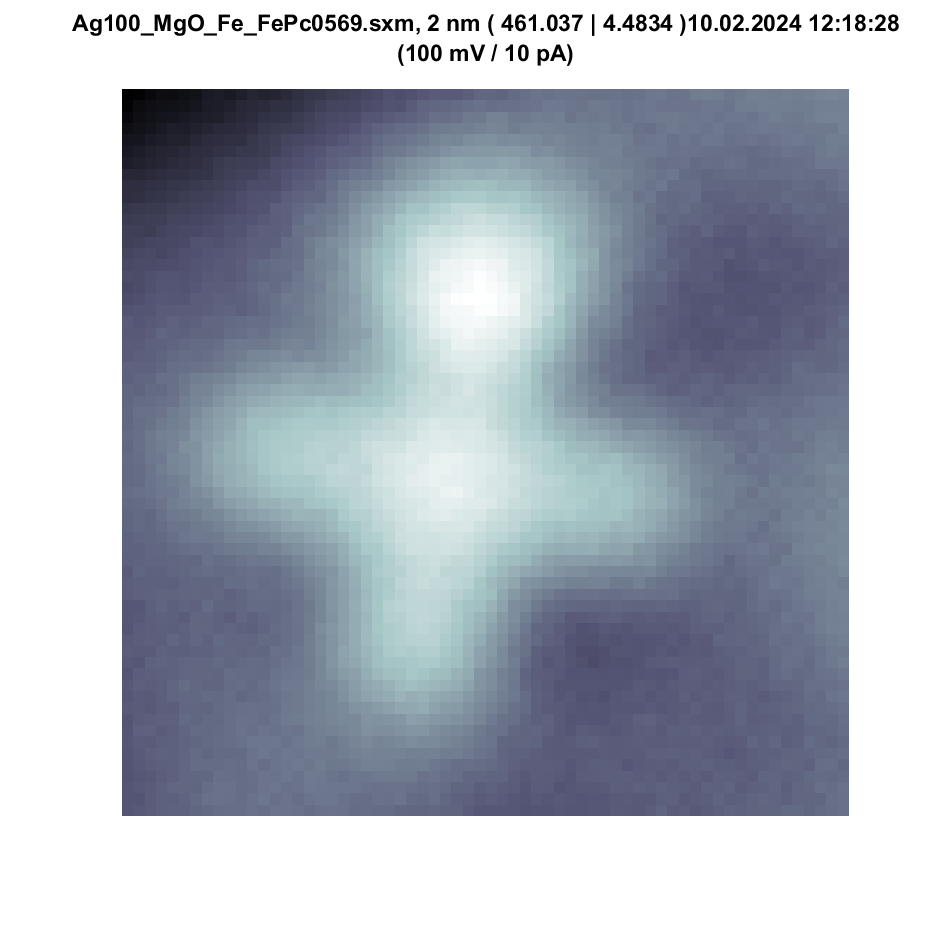

Supplement: Supplementary file 3 — Source Data [file 41467_2025_60409_MOESM3_ESM.zip › SupplementaryData1/Figure3/Fig3d/Ramsey/Ag100_MgO_Fe_FePc0569.png]

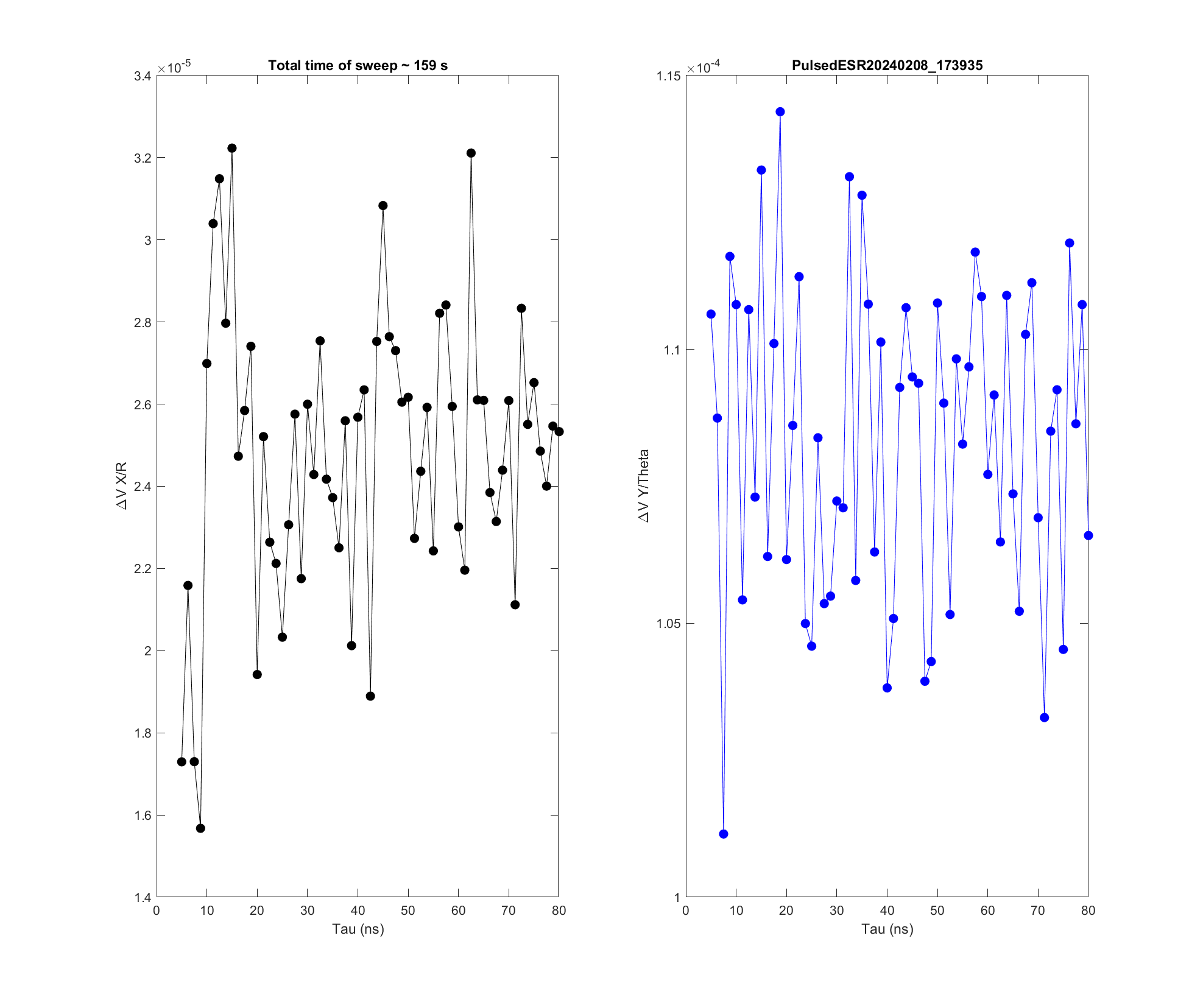

Supplement: Supplementary file 3 — Source Data [file 41467_2025_60409_MOESM3_ESM.zip › SupplementaryData1/Figure3/Fig3d/Ramsey/PulsedESR20240208_173935.png]

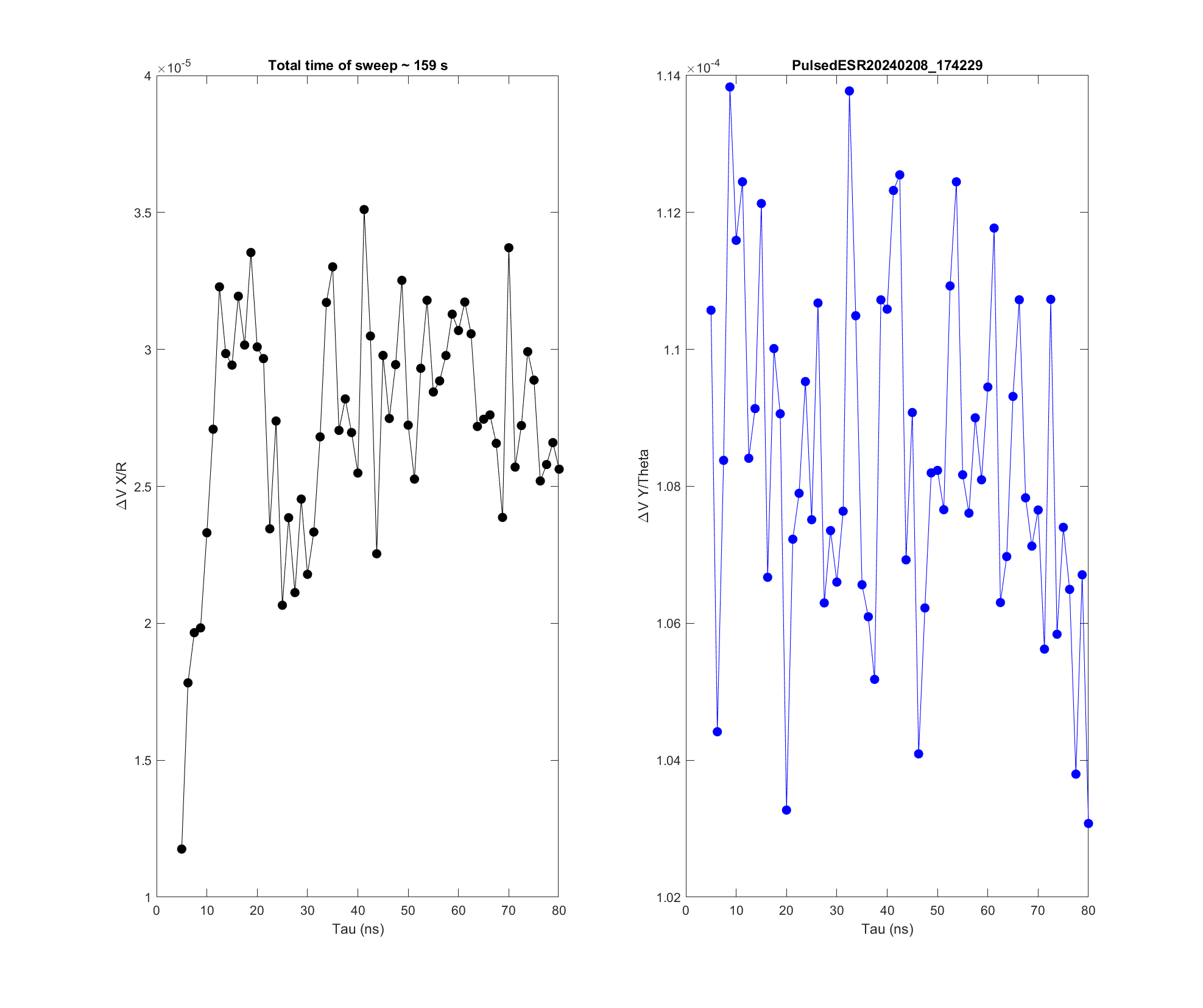

Supplement: Supplementary file 3 — Source Data [file 41467_2025_60409_MOESM3_ESM.zip › SupplementaryData1/Figure3/Fig3d/Ramsey/PulsedESR20240208_174229.png]

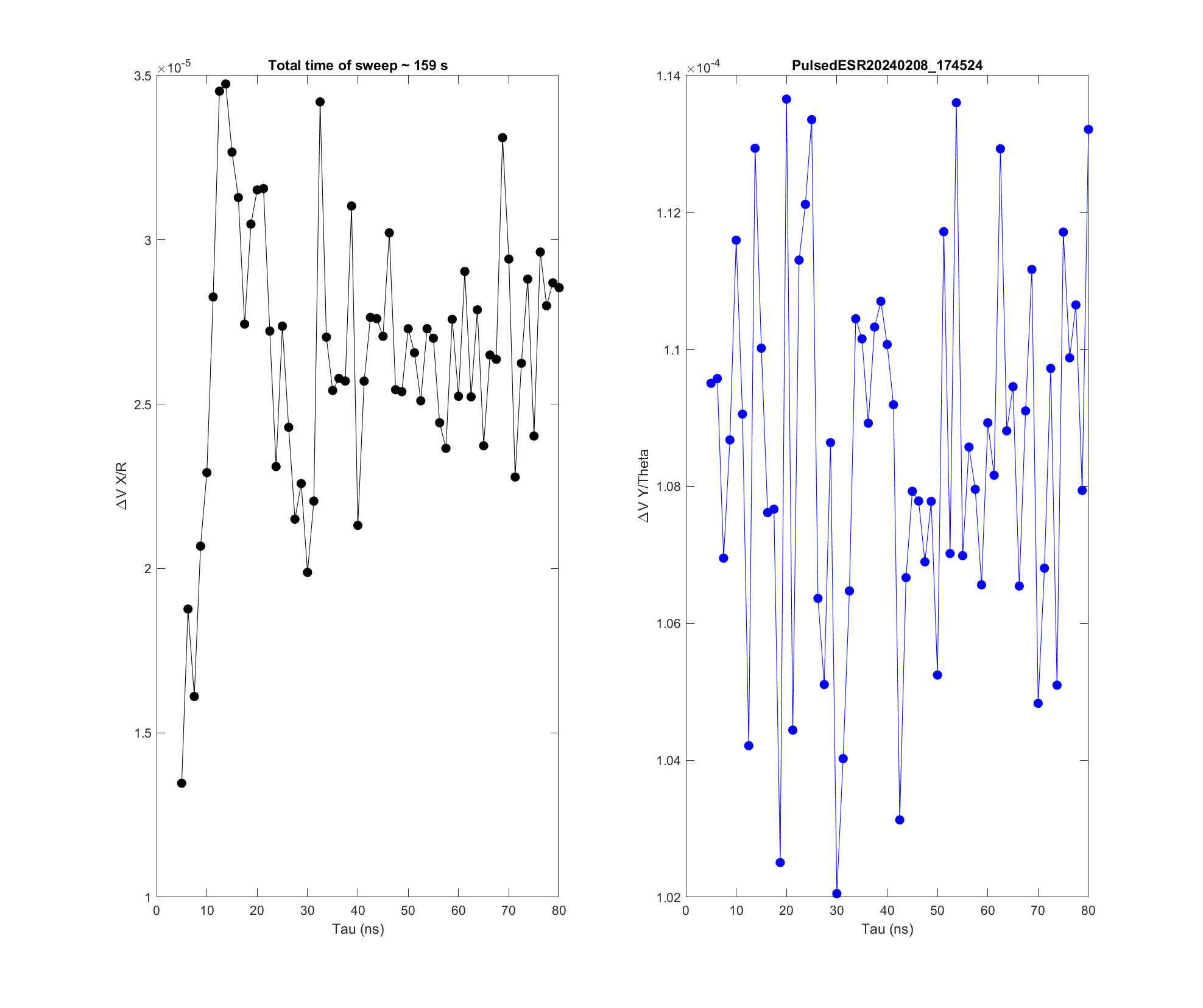

Supplement: Supplementary file 3 — Source Data [file 41467_2025_60409_MOESM3_ESM.zip › SupplementaryData1/Figure3/Fig3d/Ramsey/PulsedESR20240208_174524.png]

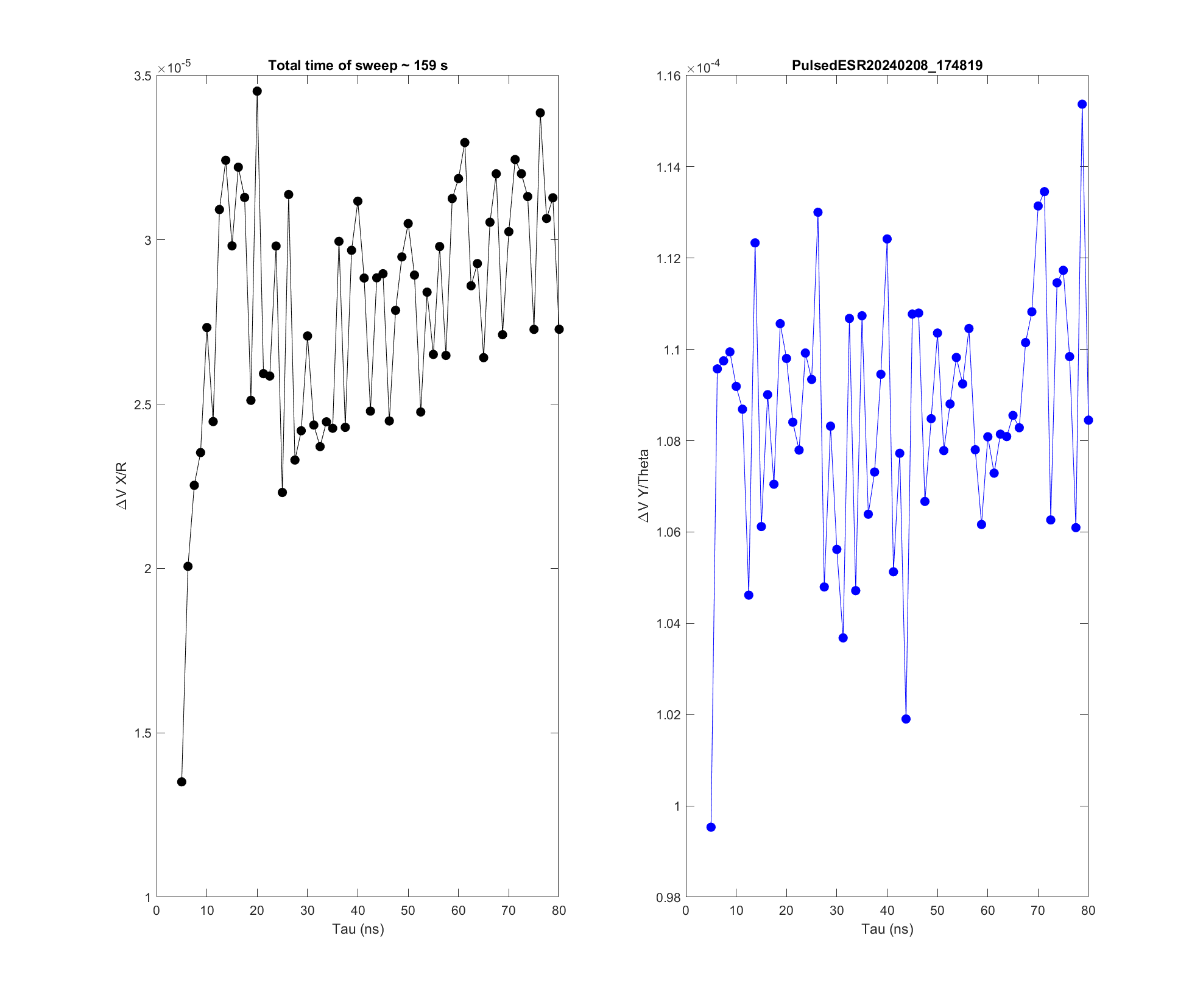

Supplement: Supplementary file 3 — Source Data [file 41467_2025_60409_MOESM3_ESM.zip › SupplementaryData1/Figure3/Fig3d/Ramsey/PulsedESR20240208_174819.png]

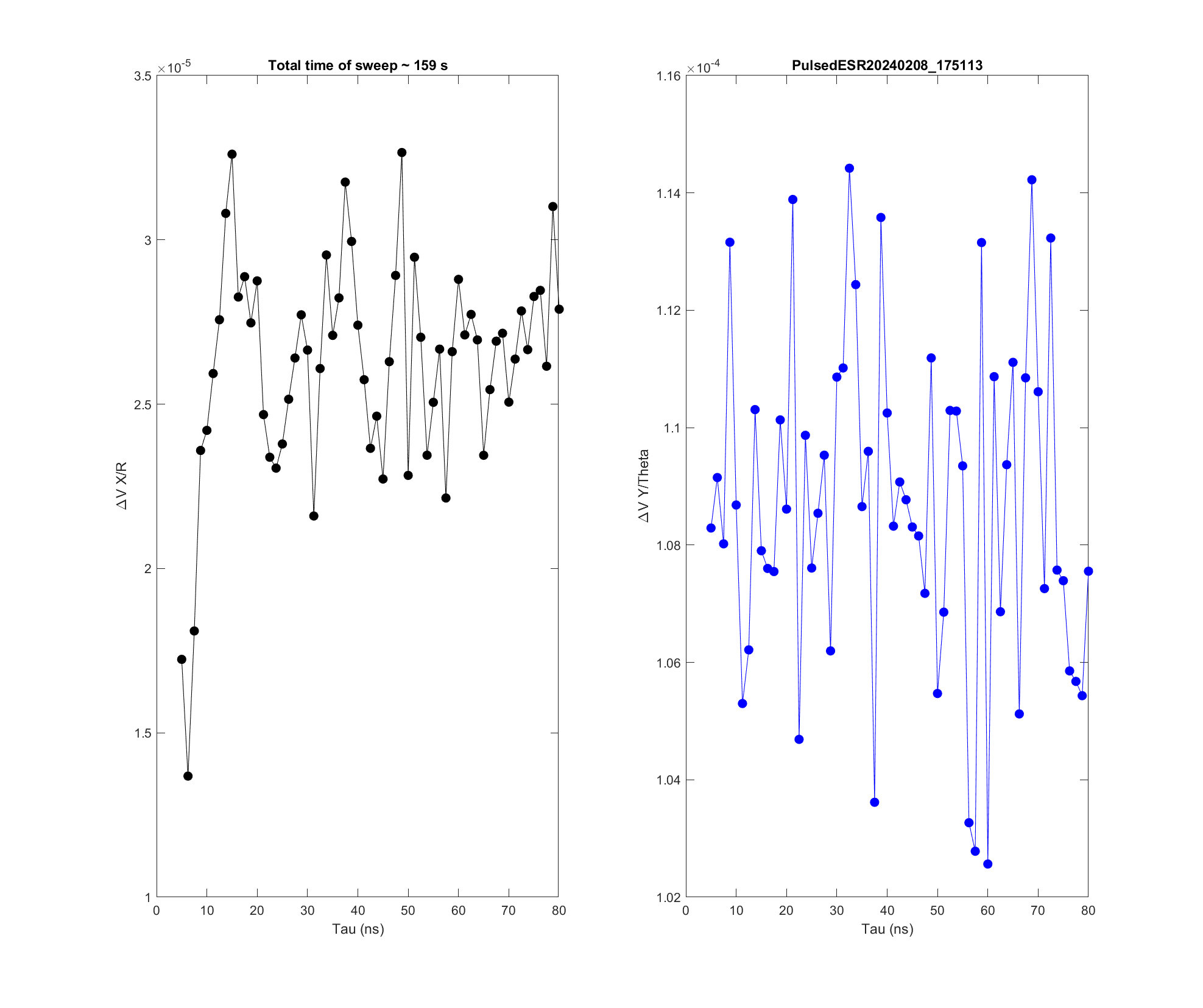

Supplement: Supplementary file 3 — Source Data [file 41467_2025_60409_MOESM3_ESM.zip › SupplementaryData1/Figure3/Fig3d/Ramsey/PulsedESR20240208_175113.png]

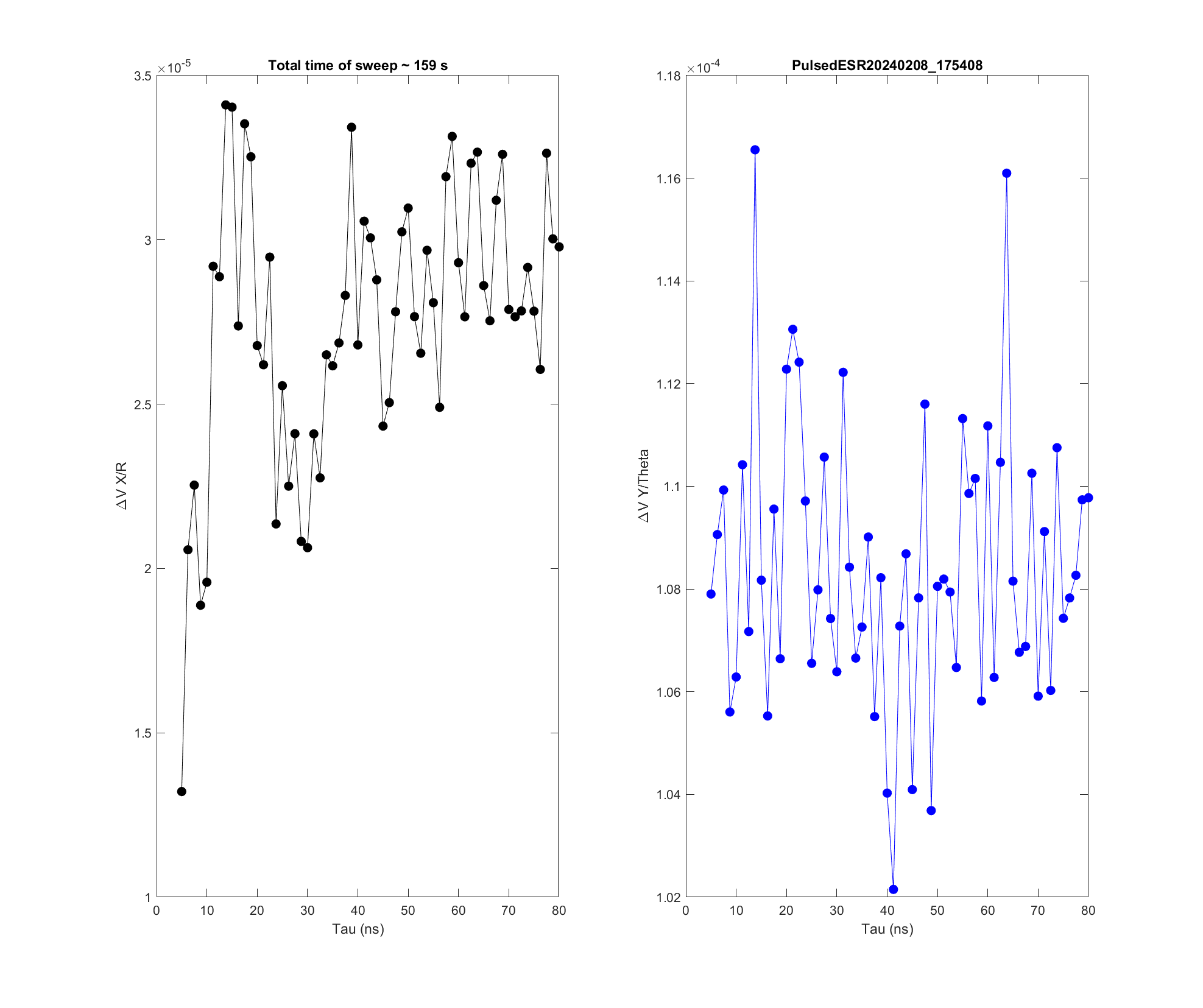

Supplement: Supplementary file 3 — Source Data [file 41467_2025_60409_MOESM3_ESM.zip › SupplementaryData1/Figure3/Fig3d/Ramsey/PulsedESR20240208_175408.png]

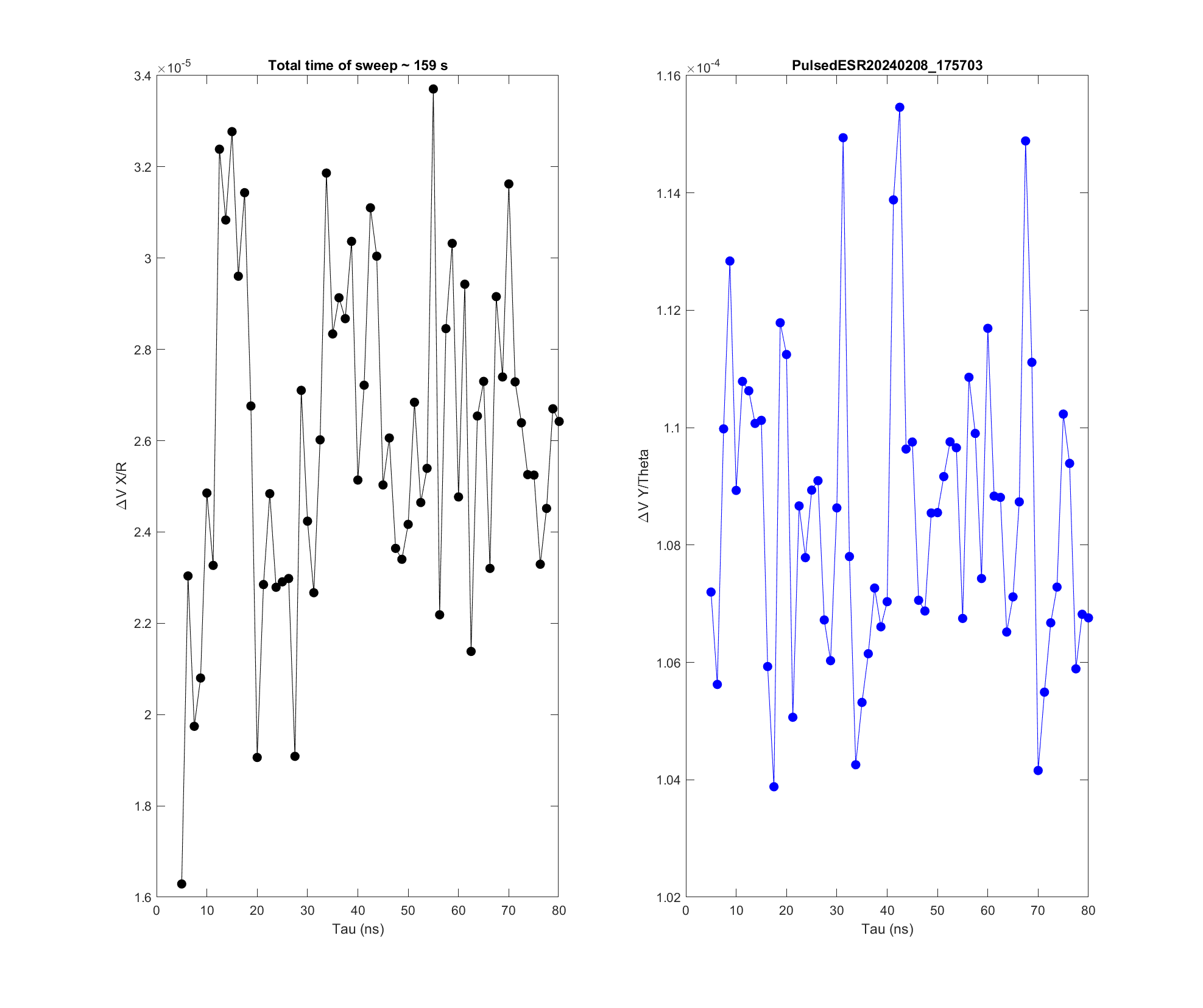

Supplement: Supplementary file 3 — Source Data [file 41467_2025_60409_MOESM3_ESM.zip › SupplementaryData1/Figure3/Fig3d/Ramsey/PulsedESR20240208_175703.png]

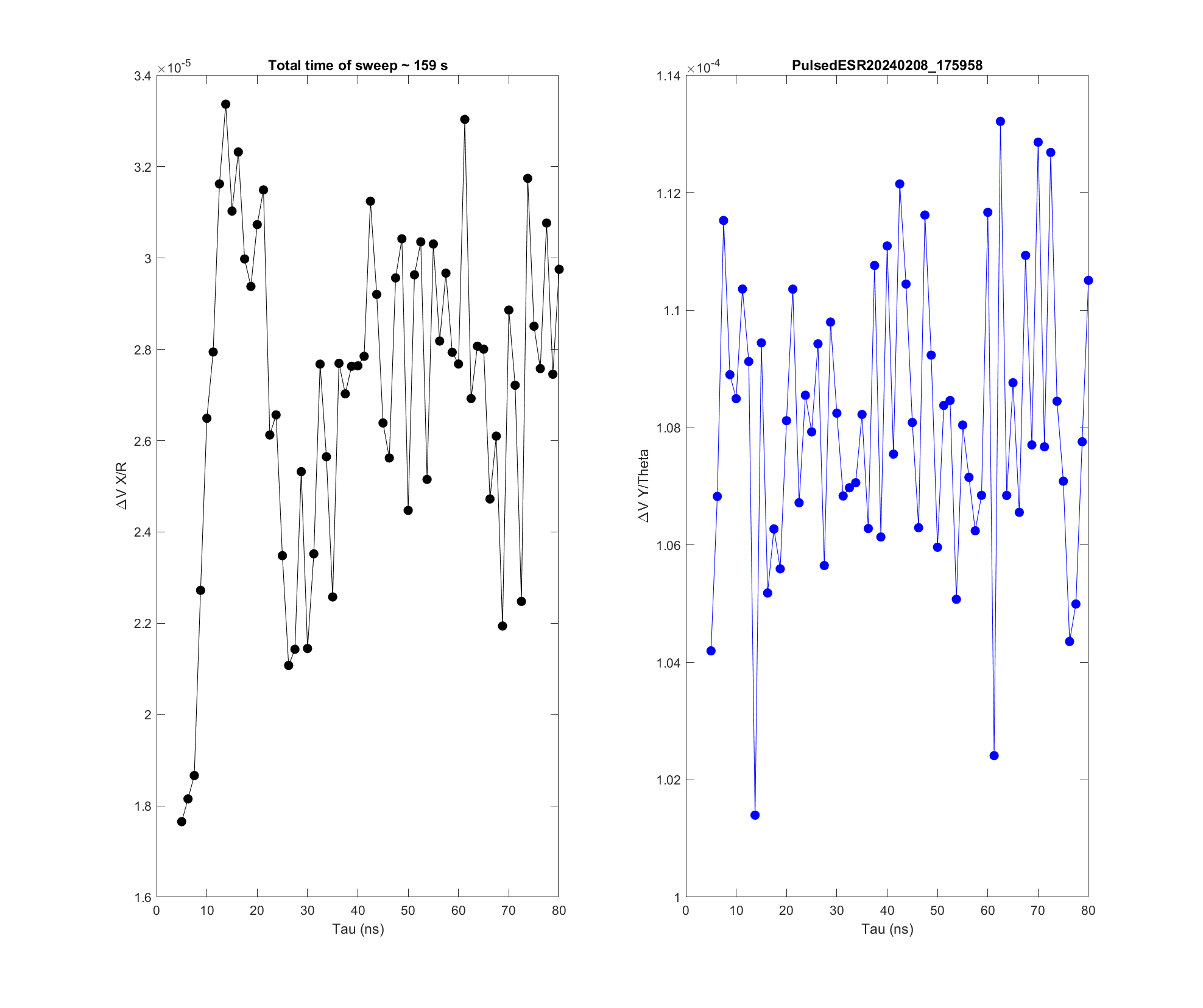

Supplement: Supplementary file 3 — Source Data [file 41467_2025_60409_MOESM3_ESM.zip › SupplementaryData1/Figure3/Fig3d/Ramsey/PulsedESR20240208_175958.png]

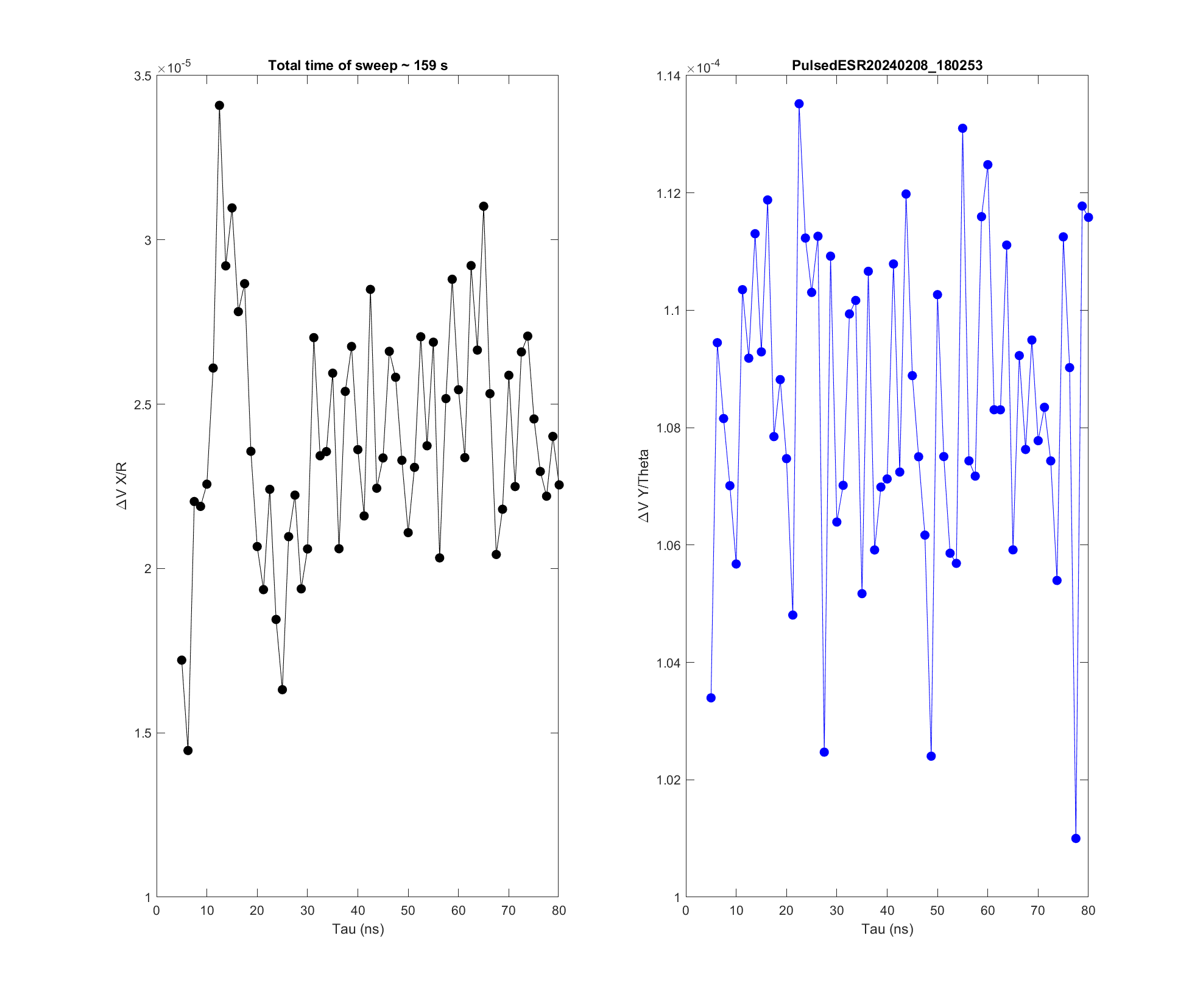

Supplement: Supplementary file 3 — Source Data [file 41467_2025_60409_MOESM3_ESM.zip › SupplementaryData1/Figure3/Fig3d/Ramsey/PulsedESR20240208_180253.png]

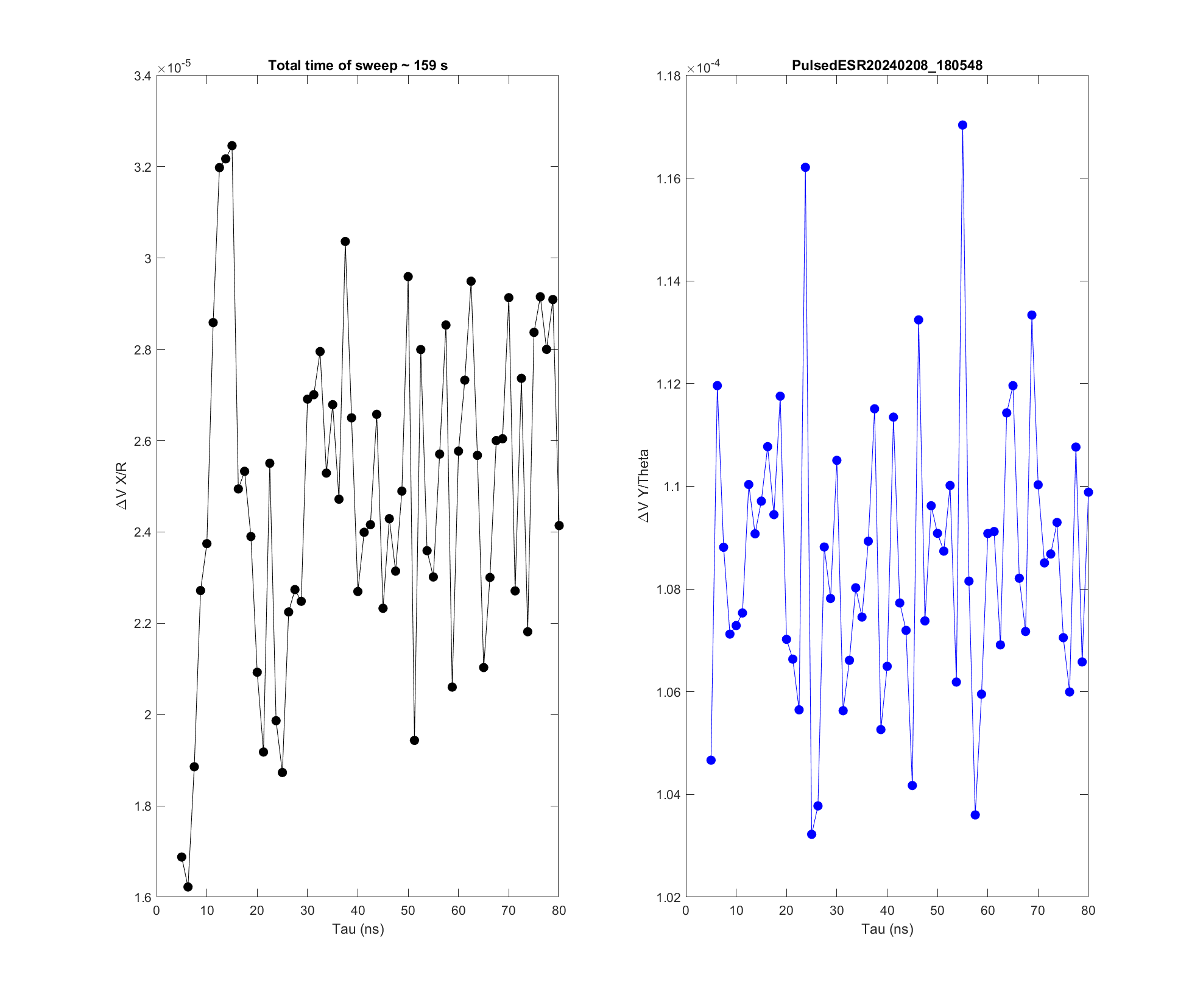

Supplement: Supplementary file 3 — Source Data [file 41467_2025_60409_MOESM3_ESM.zip › SupplementaryData1/Figure3/Fig3d/Ramsey/PulsedESR20240208_180548.png]

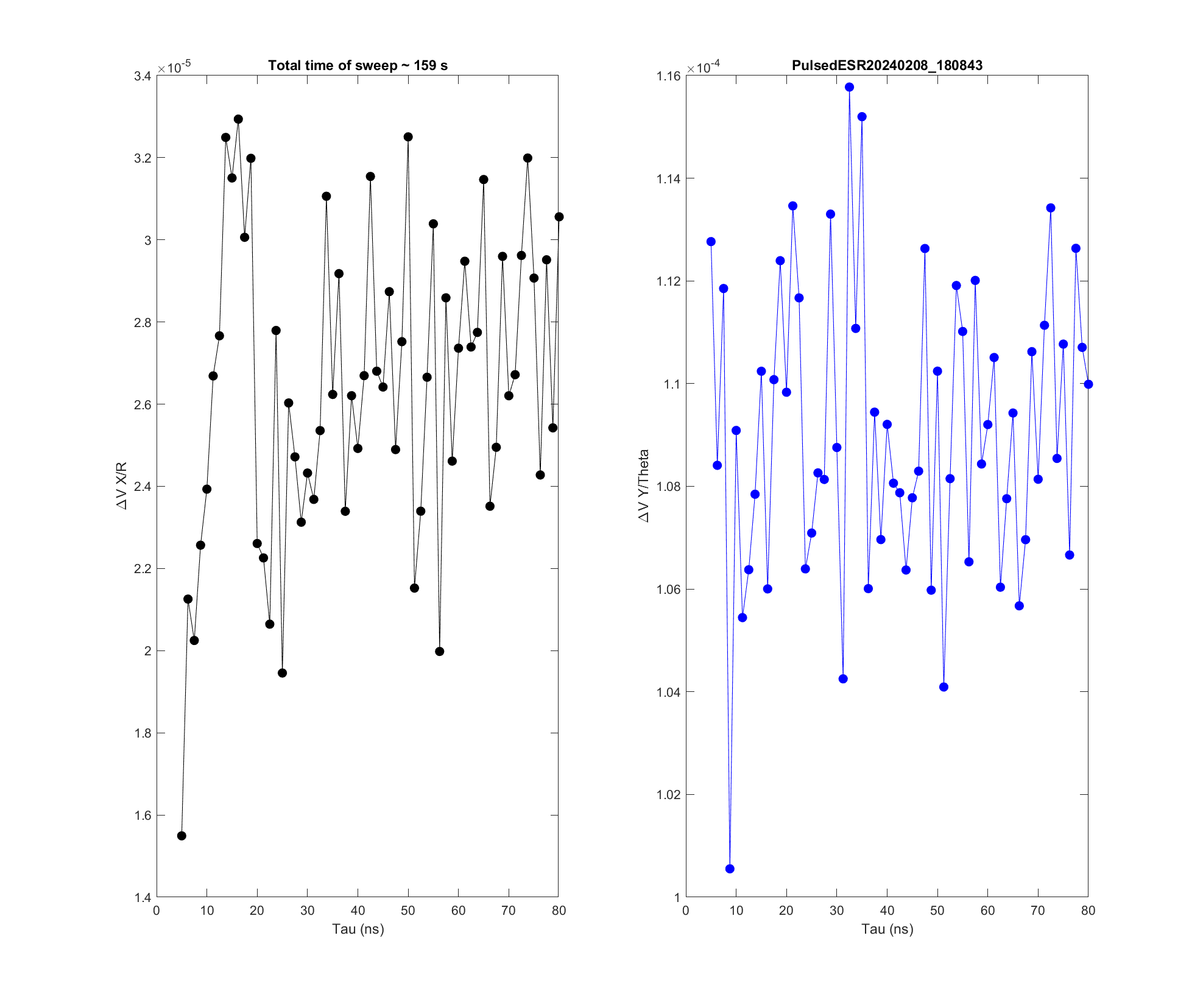

Supplement: Supplementary file 3 — Source Data [file 41467_2025_60409_MOESM3_ESM.zip › SupplementaryData1/Figure3/Fig3d/Ramsey/PulsedESR20240208_180843.png]

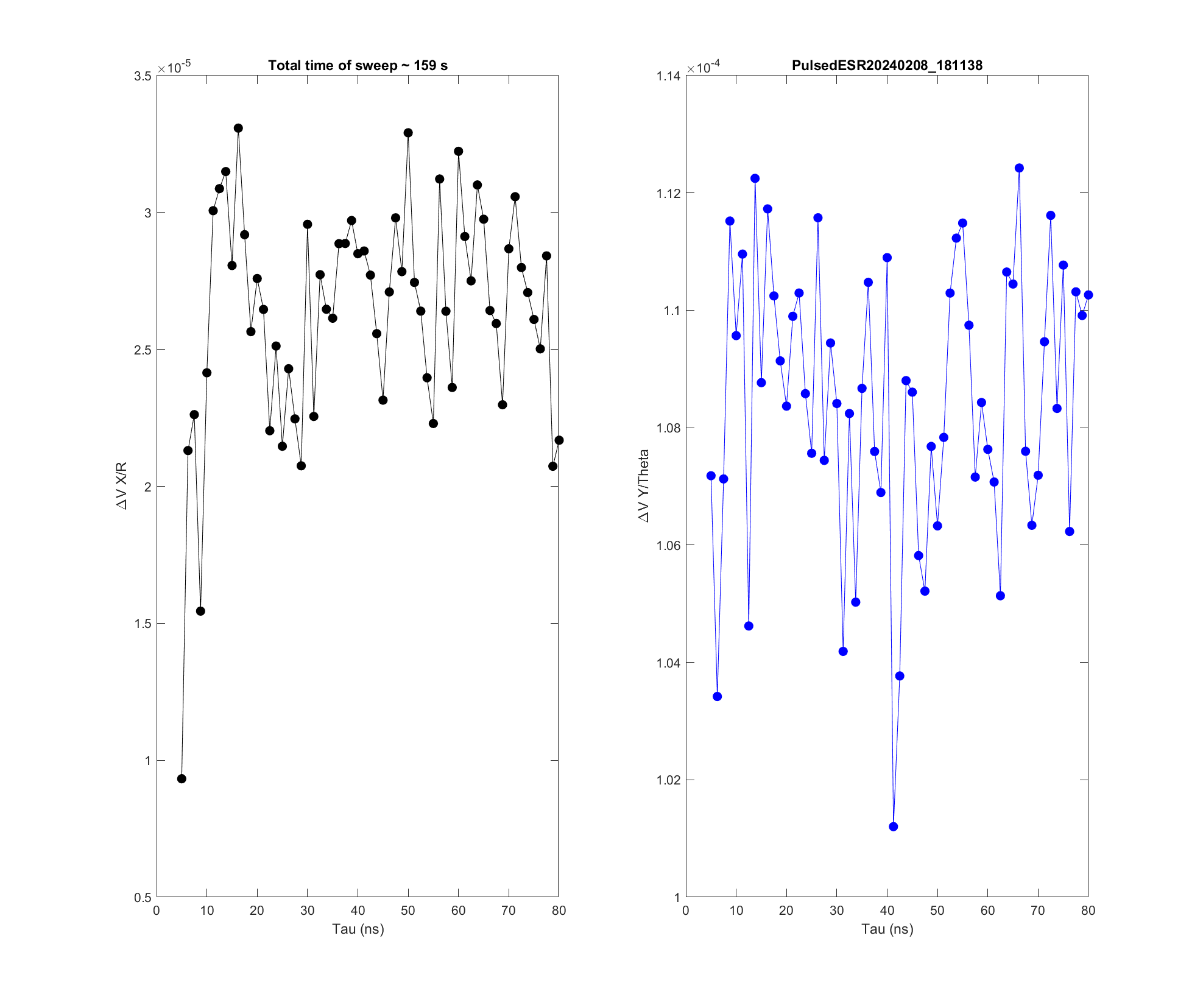

Supplement: Supplementary file 3 — Source Data [file 41467_2025_60409_MOESM3_ESM.zip › SupplementaryData1/Figure3/Fig3d/Ramsey/PulsedESR20240208_181138.png]

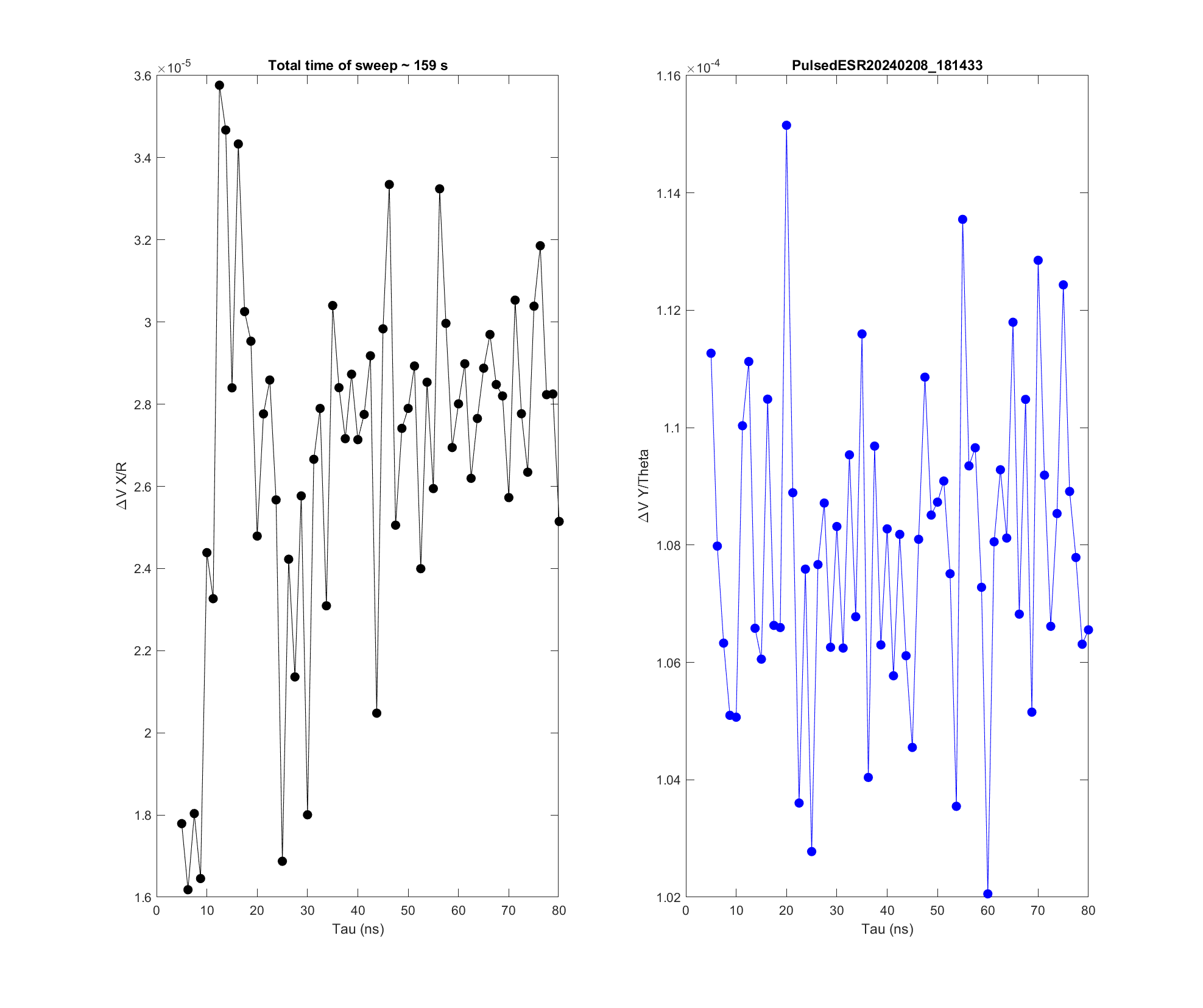

Supplement: Supplementary file 3 — Source Data [file 41467_2025_60409_MOESM3_ESM.zip › SupplementaryData1/Figure3/Fig3d/Ramsey/PulsedESR20240208_181433.png]

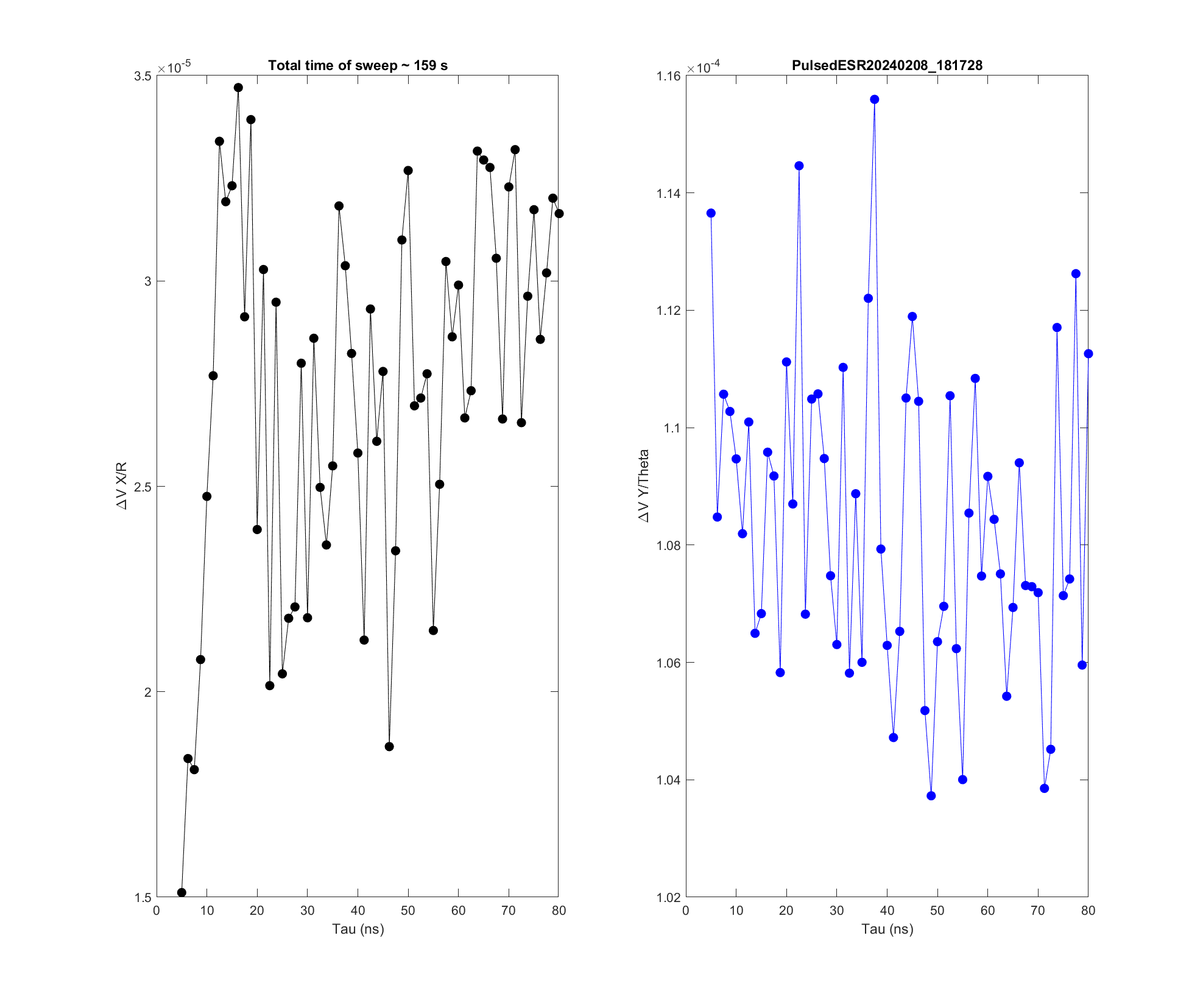

Supplement: Supplementary file 3 — Source Data [file 41467_2025_60409_MOESM3_ESM.zip › SupplementaryData1/Figure3/Fig3d/Ramsey/PulsedESR20240208_181728.png]

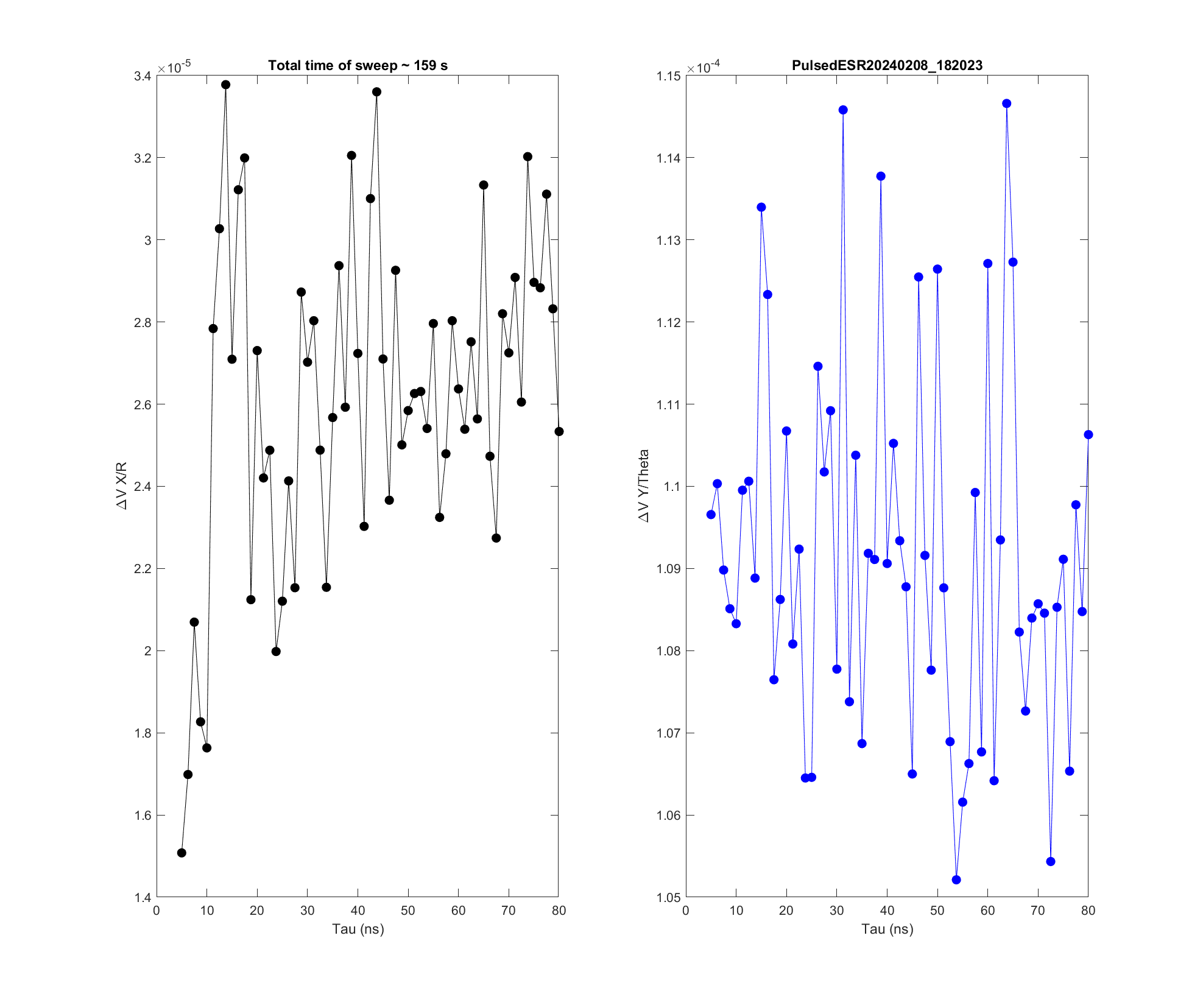

Supplement: Supplementary file 3 — Source Data [file 41467_2025_60409_MOESM3_ESM.zip › SupplementaryData1/Figure3/Fig3d/Ramsey/PulsedESR20240208_182023.png]

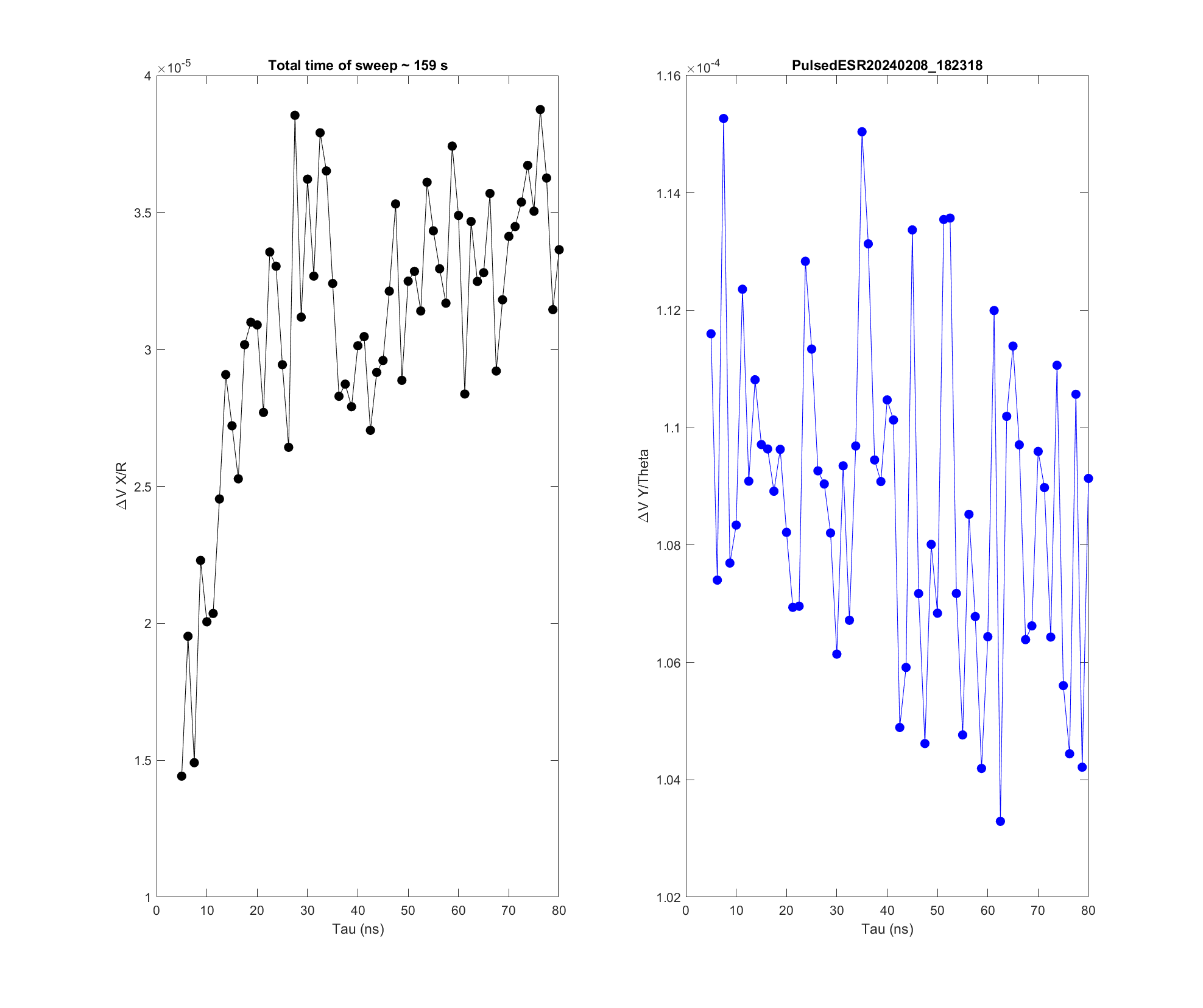

Supplement: Supplementary file 3 — Source Data [file 41467_2025_60409_MOESM3_ESM.zip › SupplementaryData1/Figure3/Fig3d/Ramsey/PulsedESR20240208_182318.png]

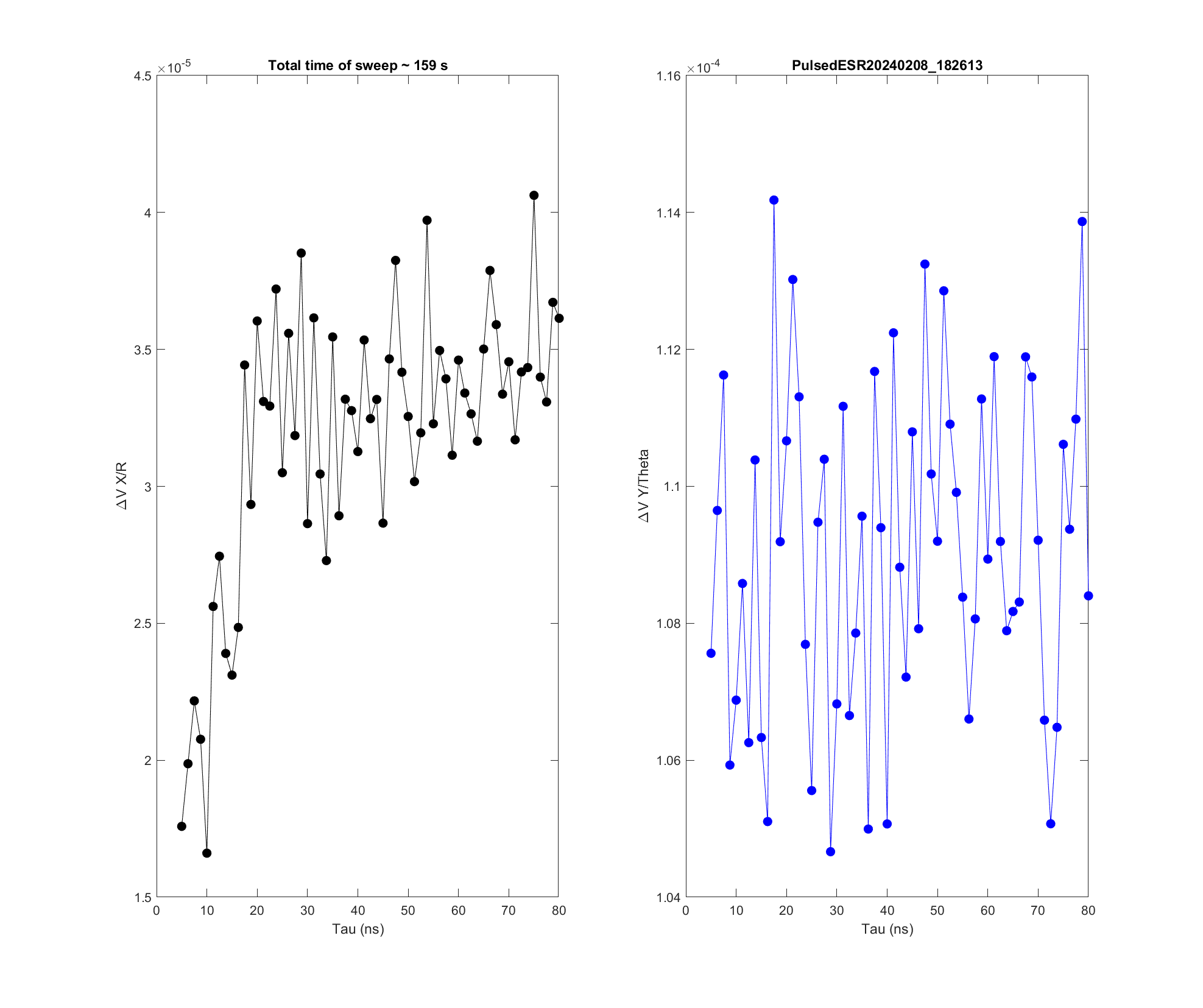

Supplement: Supplementary file 3 — Source Data [file 41467_2025_60409_MOESM3_ESM.zip › SupplementaryData1/Figure3/Fig3d/Ramsey/PulsedESR20240208_182613.png]

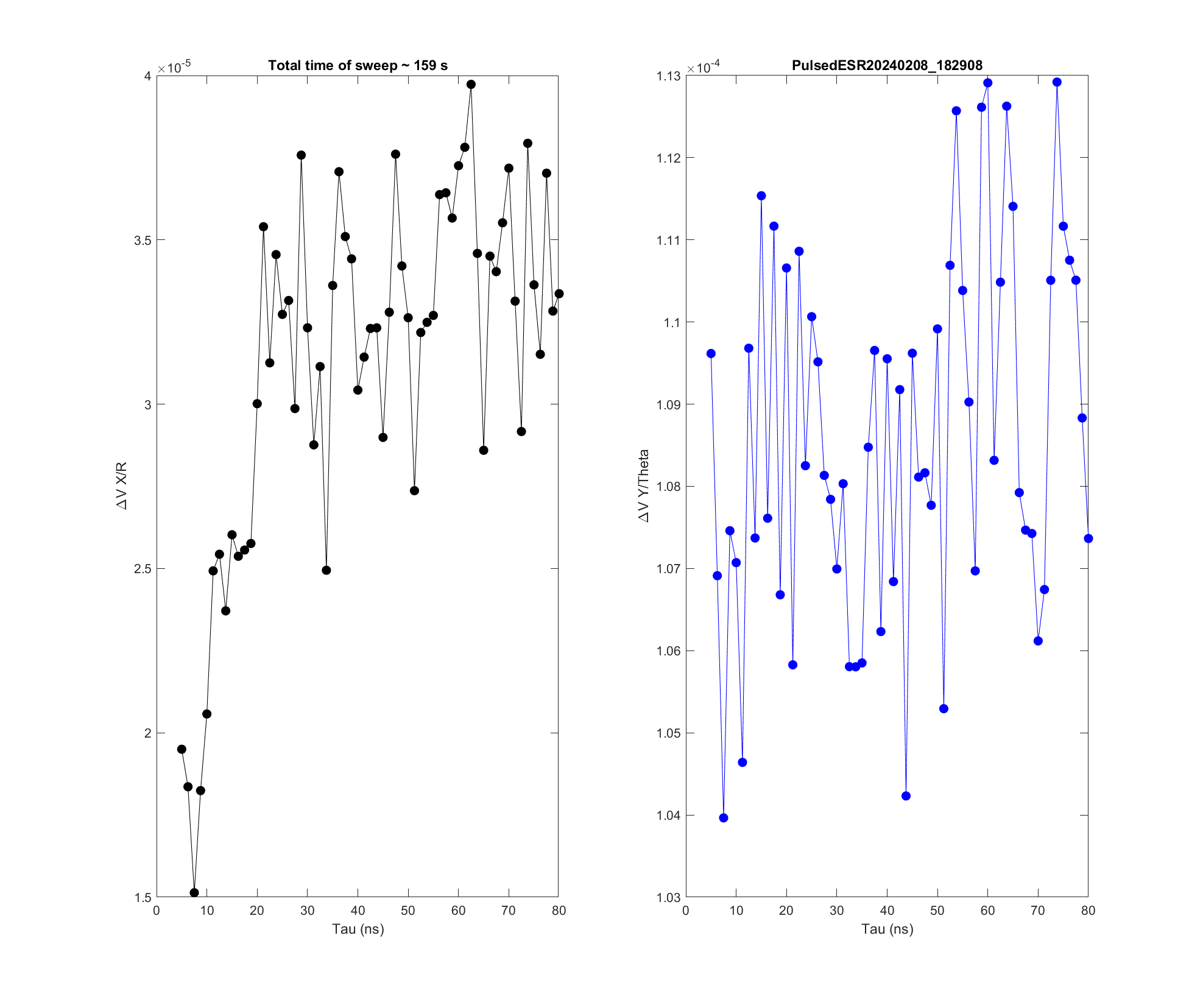

Supplement: Supplementary file 3 — Source Data [file 41467_2025_60409_MOESM3_ESM.zip › SupplementaryData1/Figure3/Fig3d/Ramsey/PulsedESR20240208_182908.png]

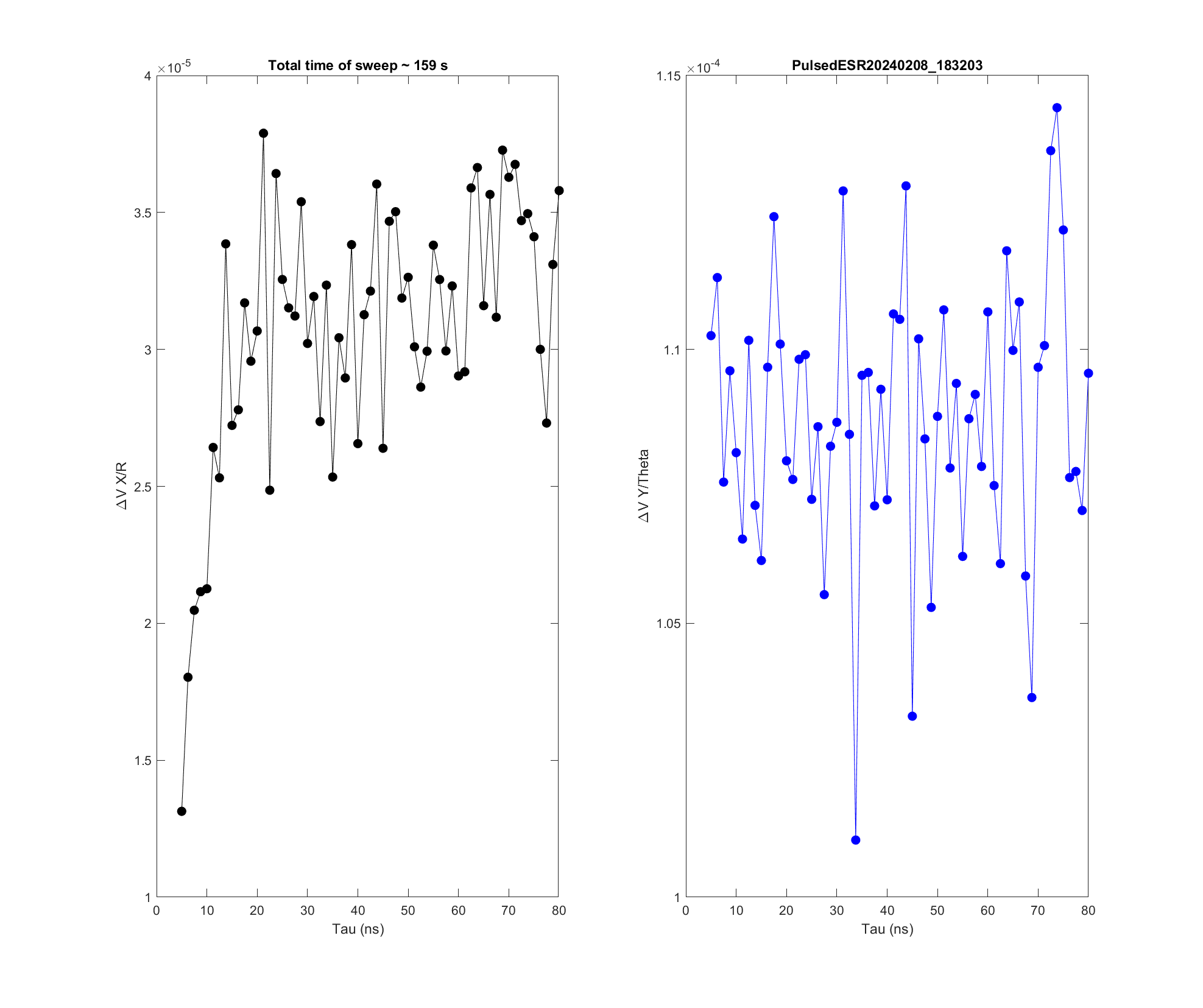

Supplement: Supplementary file 3 — Source Data [file 41467_2025_60409_MOESM3_ESM.zip › SupplementaryData1/Figure3/Fig3d/Ramsey/PulsedESR20240208_183203.png]

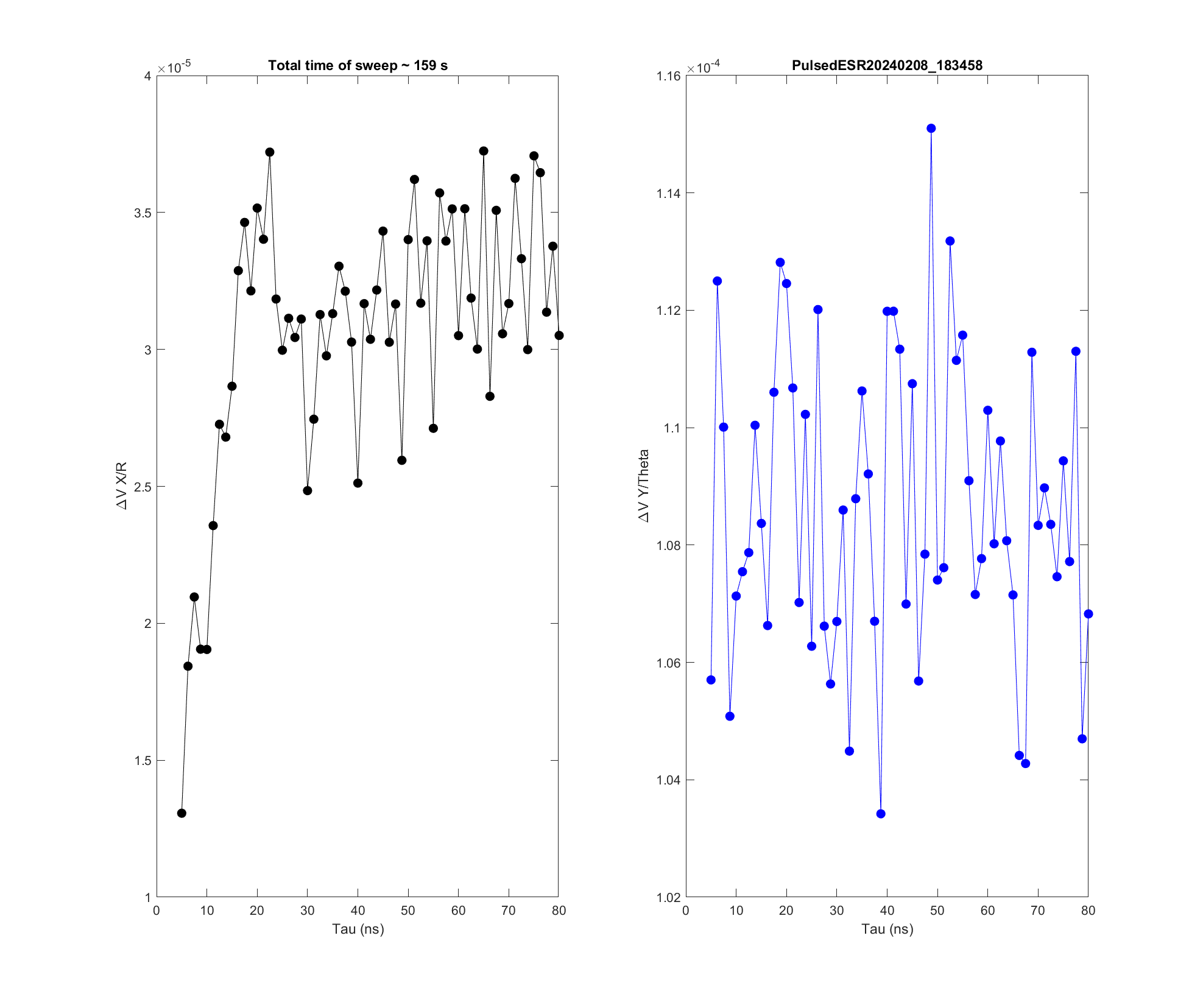

Supplement: Supplementary file 3 — Source Data [file 41467_2025_60409_MOESM3_ESM.zip › SupplementaryData1/Figure3/Fig3d/Ramsey/PulsedESR20240208_183458.png]

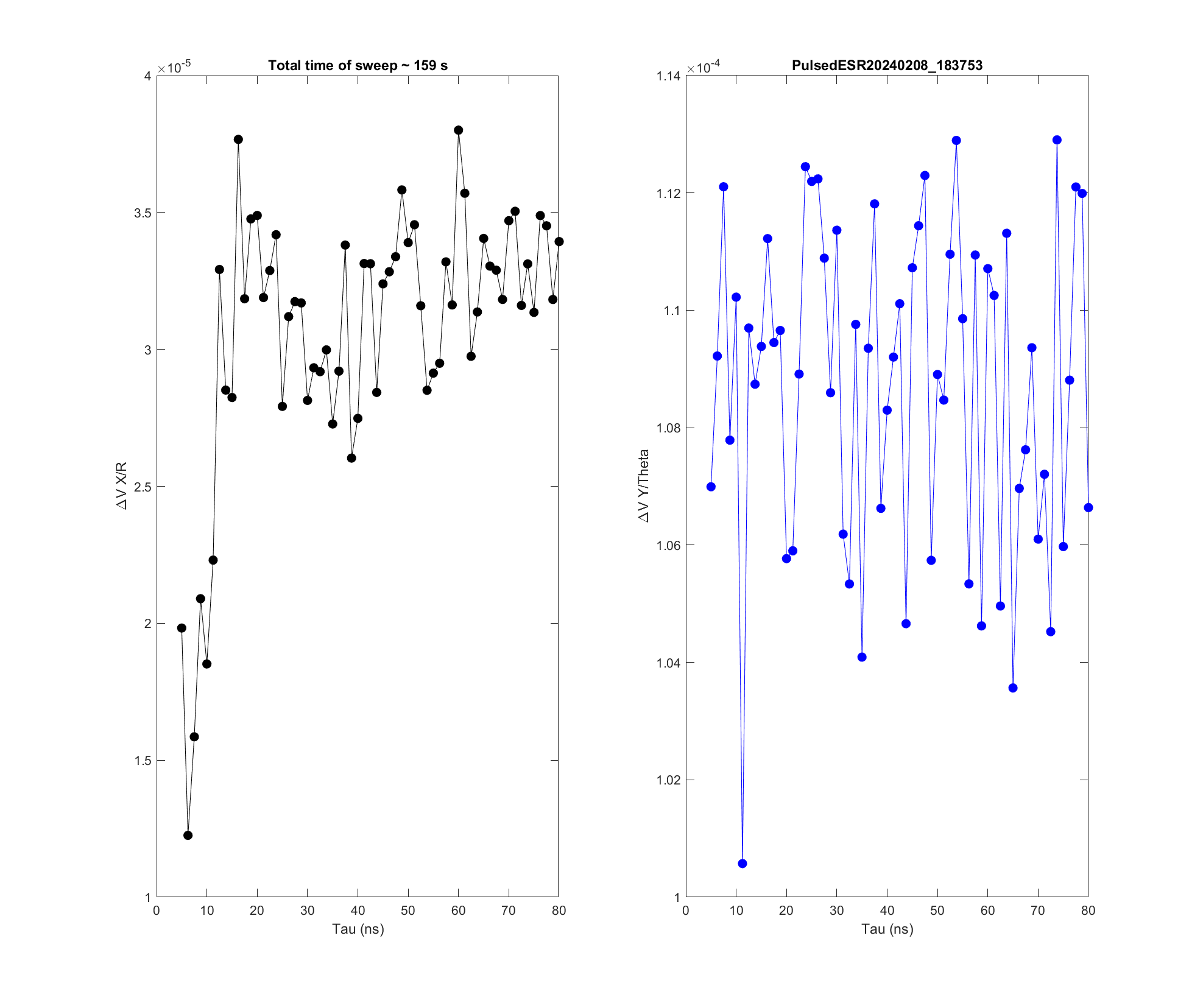

Supplement: Supplementary file 3 — Source Data [file 41467_2025_60409_MOESM3_ESM.zip › SupplementaryData1/Figure3/Fig3d/Ramsey/PulsedESR20240208_183753.png]

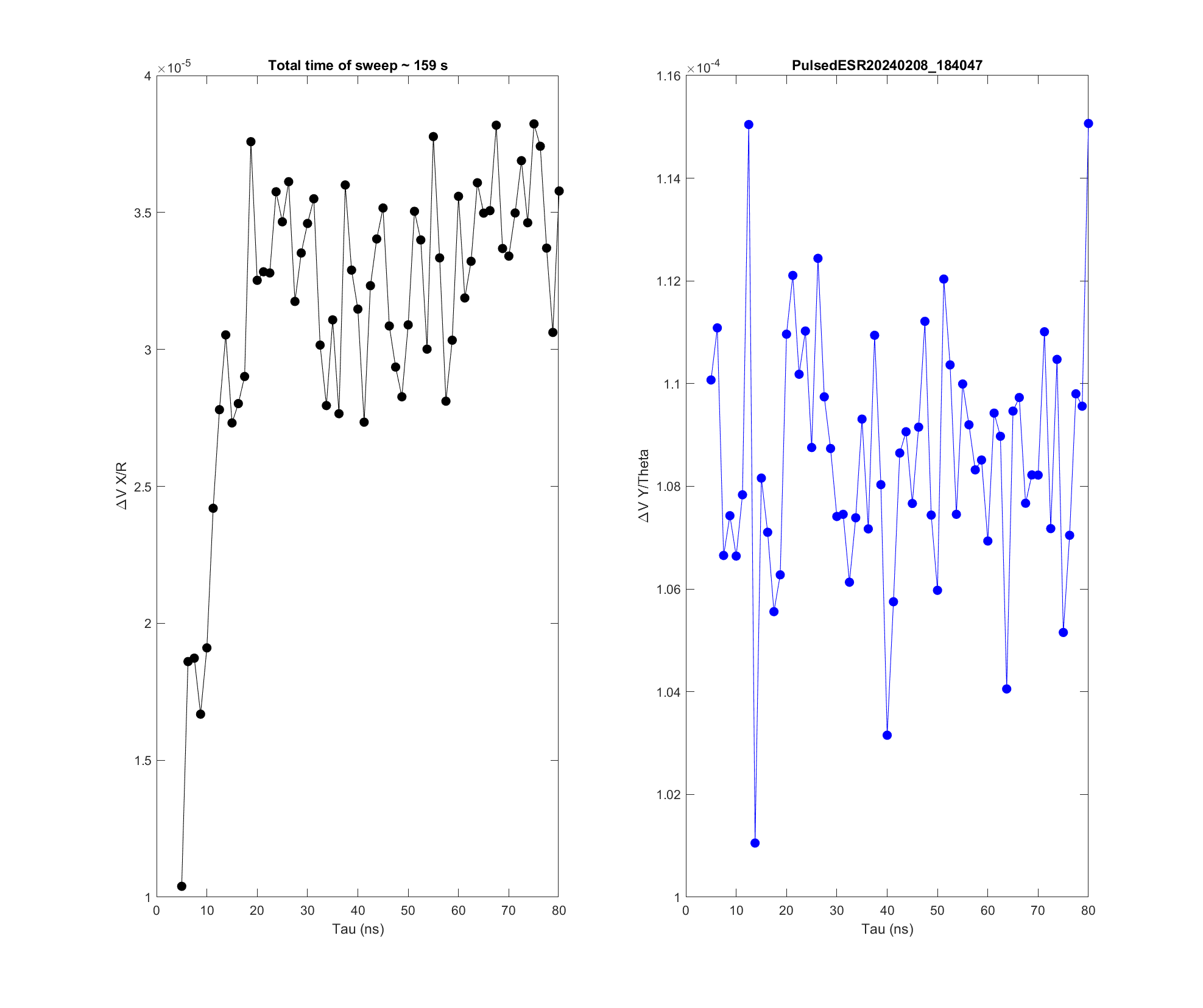

Supplement: Supplementary file 3 — Source Data [file 41467_2025_60409_MOESM3_ESM.zip › SupplementaryData1/Figure3/Fig3d/Ramsey/PulsedESR20240208_184047.png]

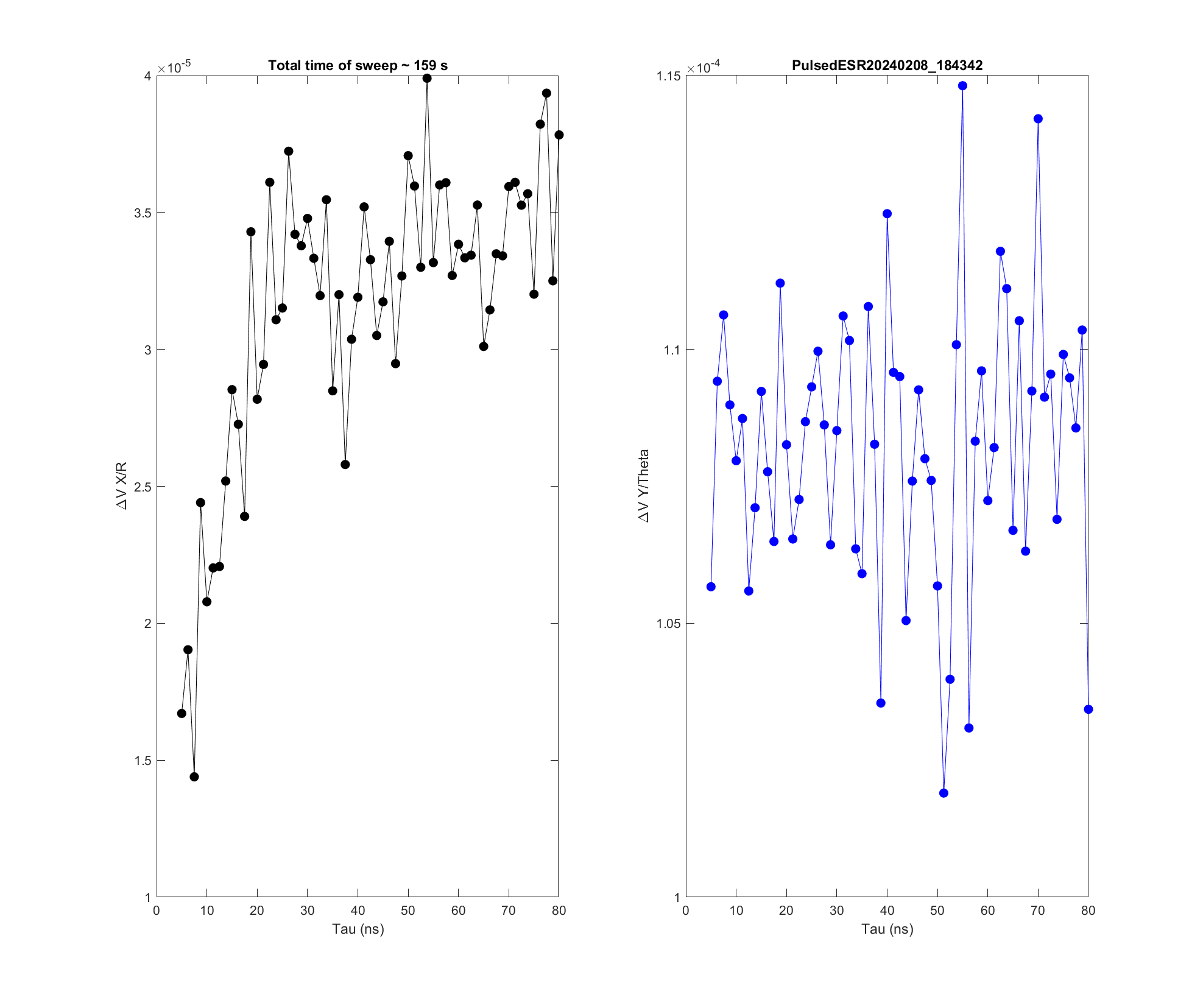

Supplement: Supplementary file 3 — Source Data [file 41467_2025_60409_MOESM3_ESM.zip › SupplementaryData1/Figure3/Fig3d/Ramsey/PulsedESR20240208_184342.png]

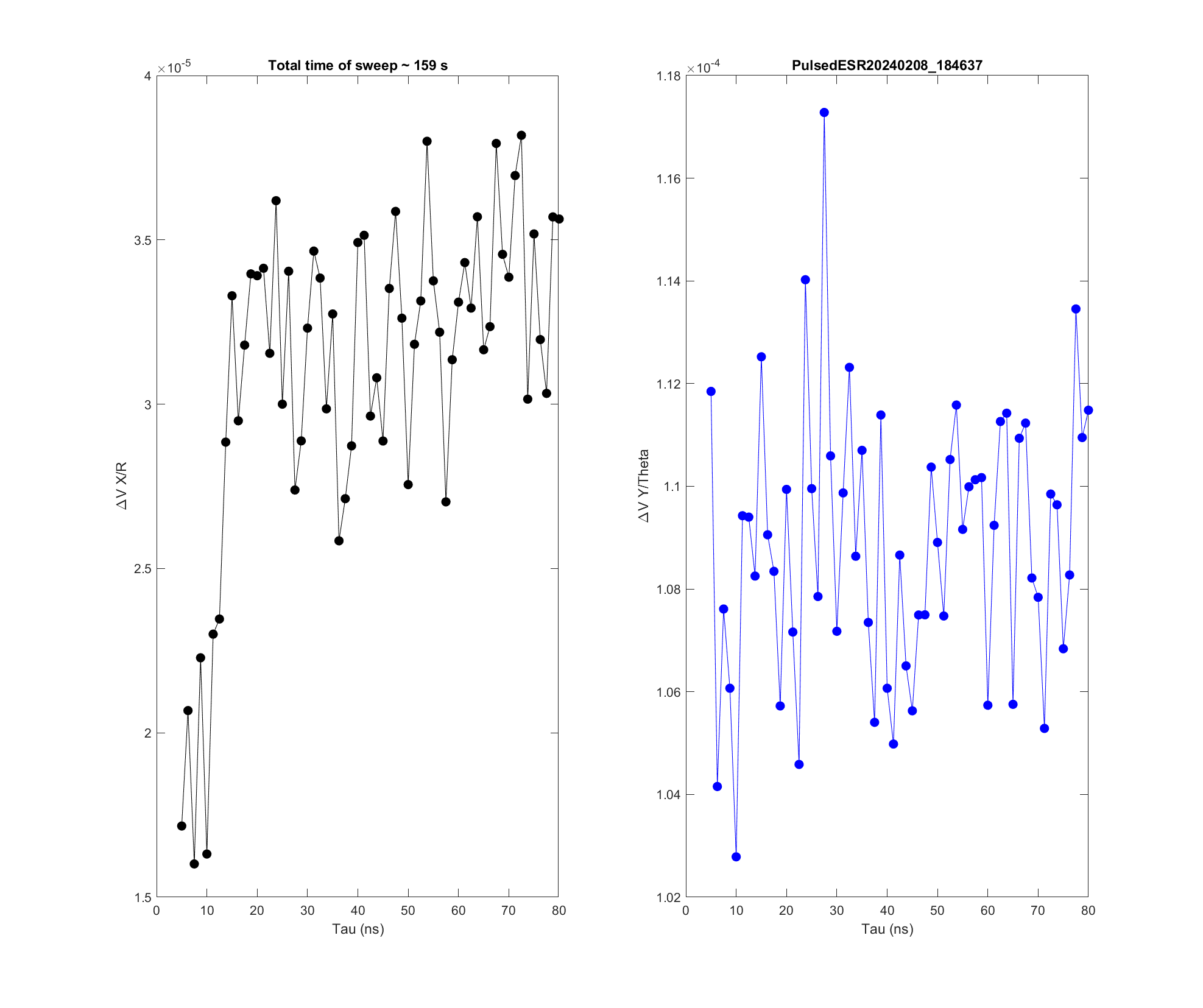

Supplement: Supplementary file 3 — Source Data [file 41467_2025_60409_MOESM3_ESM.zip › SupplementaryData1/Figure3/Fig3d/Ramsey/PulsedESR20240208_184637.png]

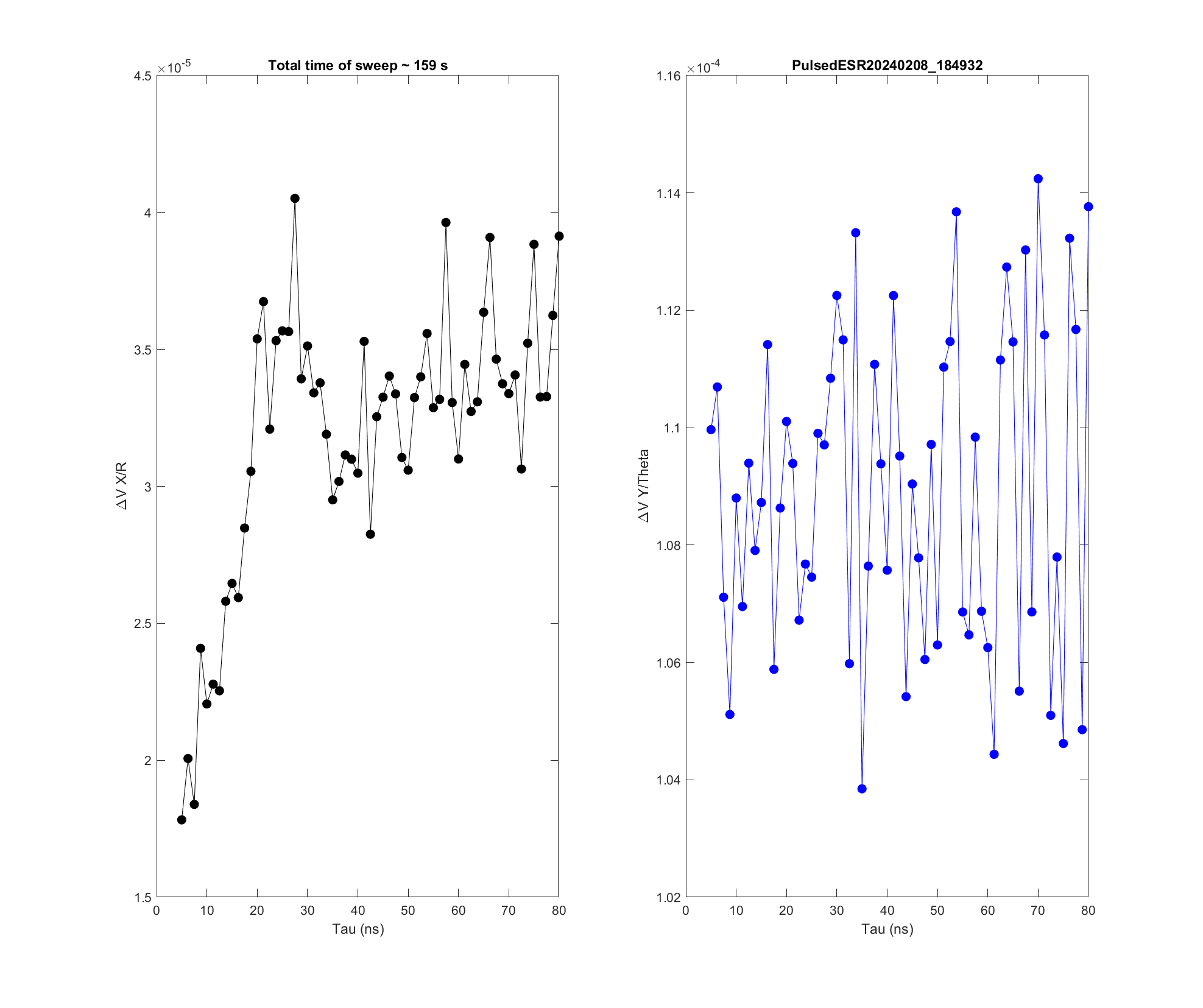

Supplement: Supplementary file 3 — Source Data [file 41467_2025_60409_MOESM3_ESM.zip › SupplementaryData1/Figure3/Fig3d/Ramsey/PulsedESR20240208_184932.png]

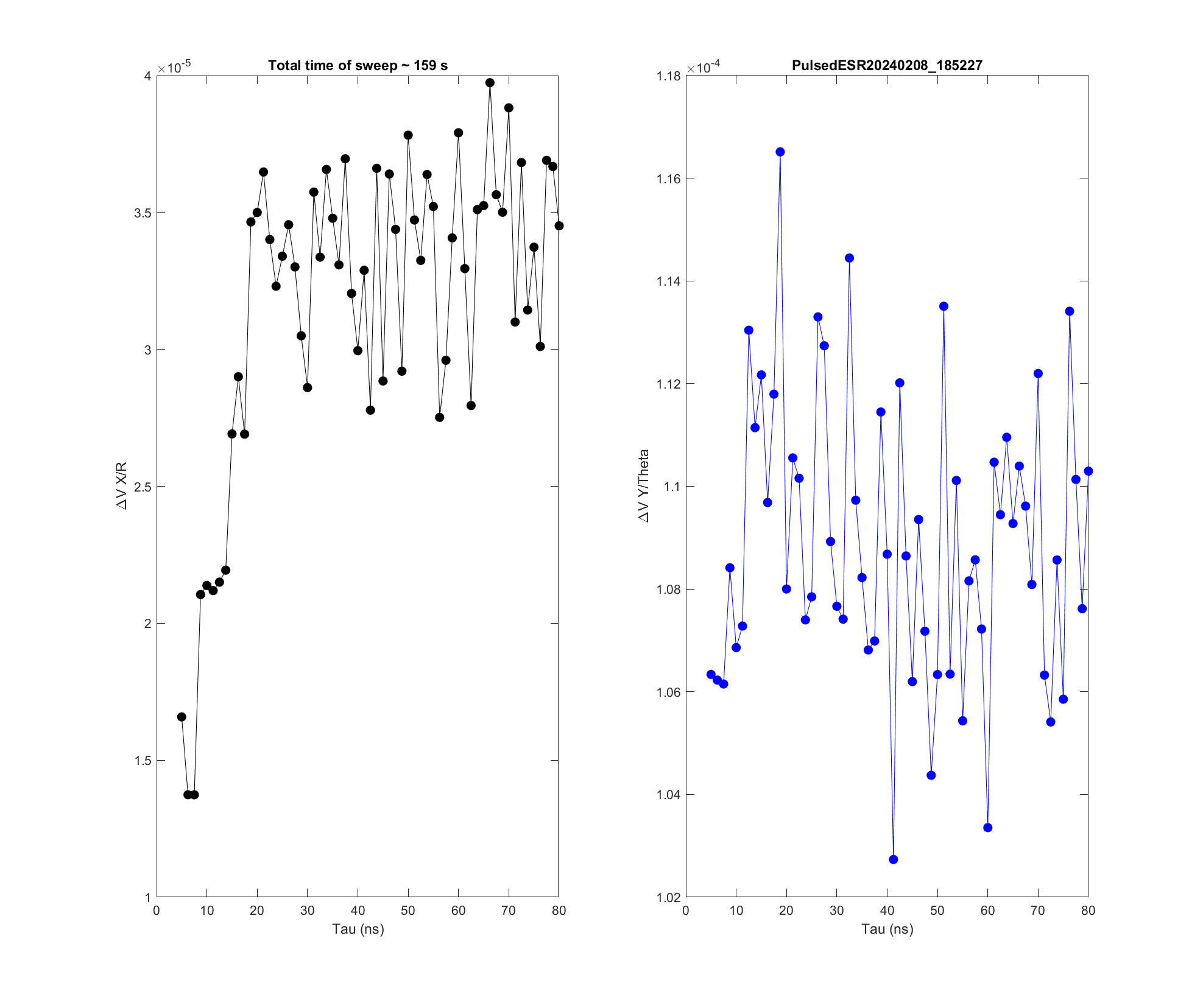

Supplement: Supplementary file 3 — Source Data [file 41467_2025_60409_MOESM3_ESM.zip › SupplementaryData1/Figure3/Fig3d/Ramsey/PulsedESR20240208_185227.png]

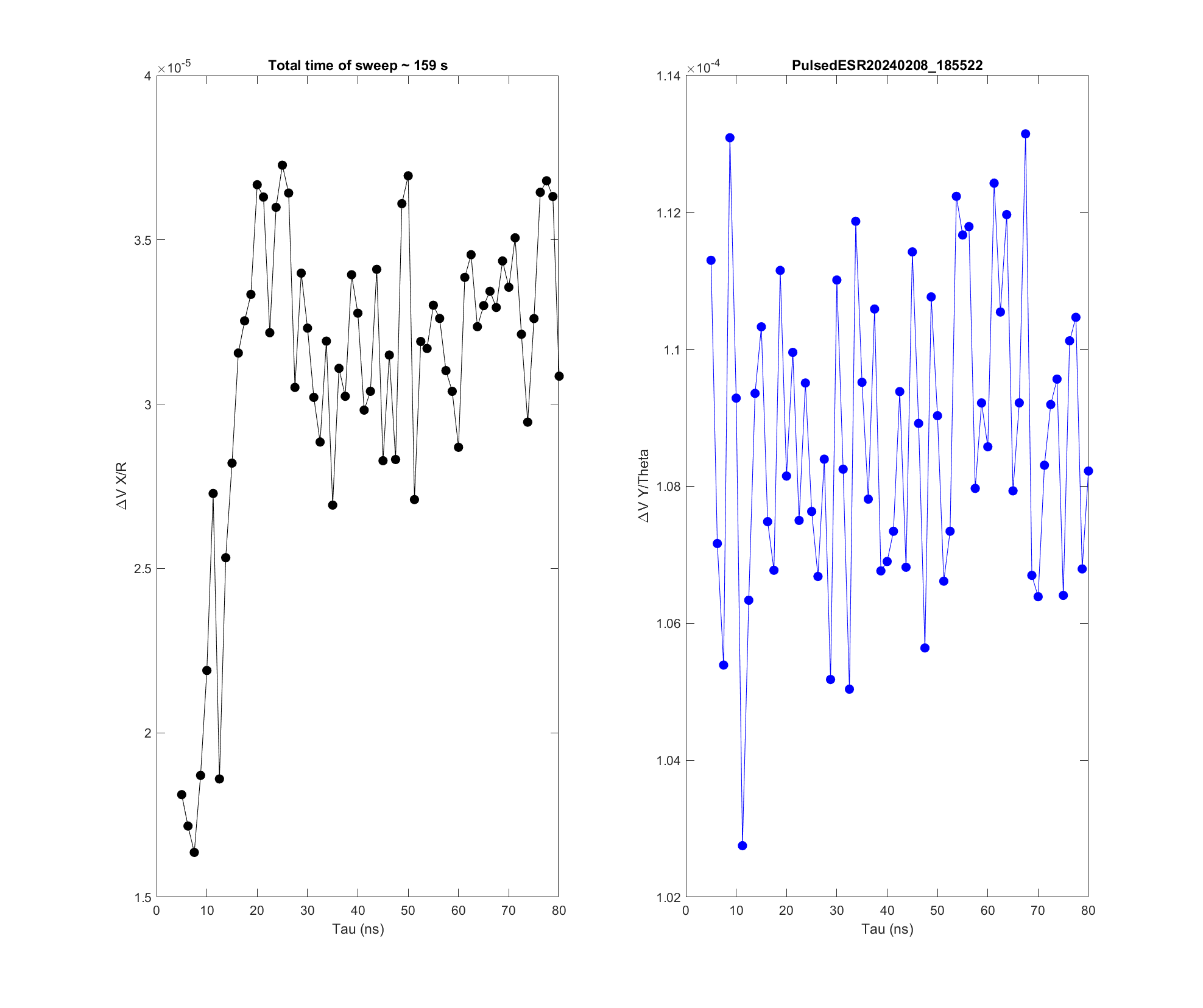

Supplement: Supplementary file 3 — Source Data [file 41467_2025_60409_MOESM3_ESM.zip › SupplementaryData1/Figure3/Fig3d/Ramsey/PulsedESR20240208_185522.png]

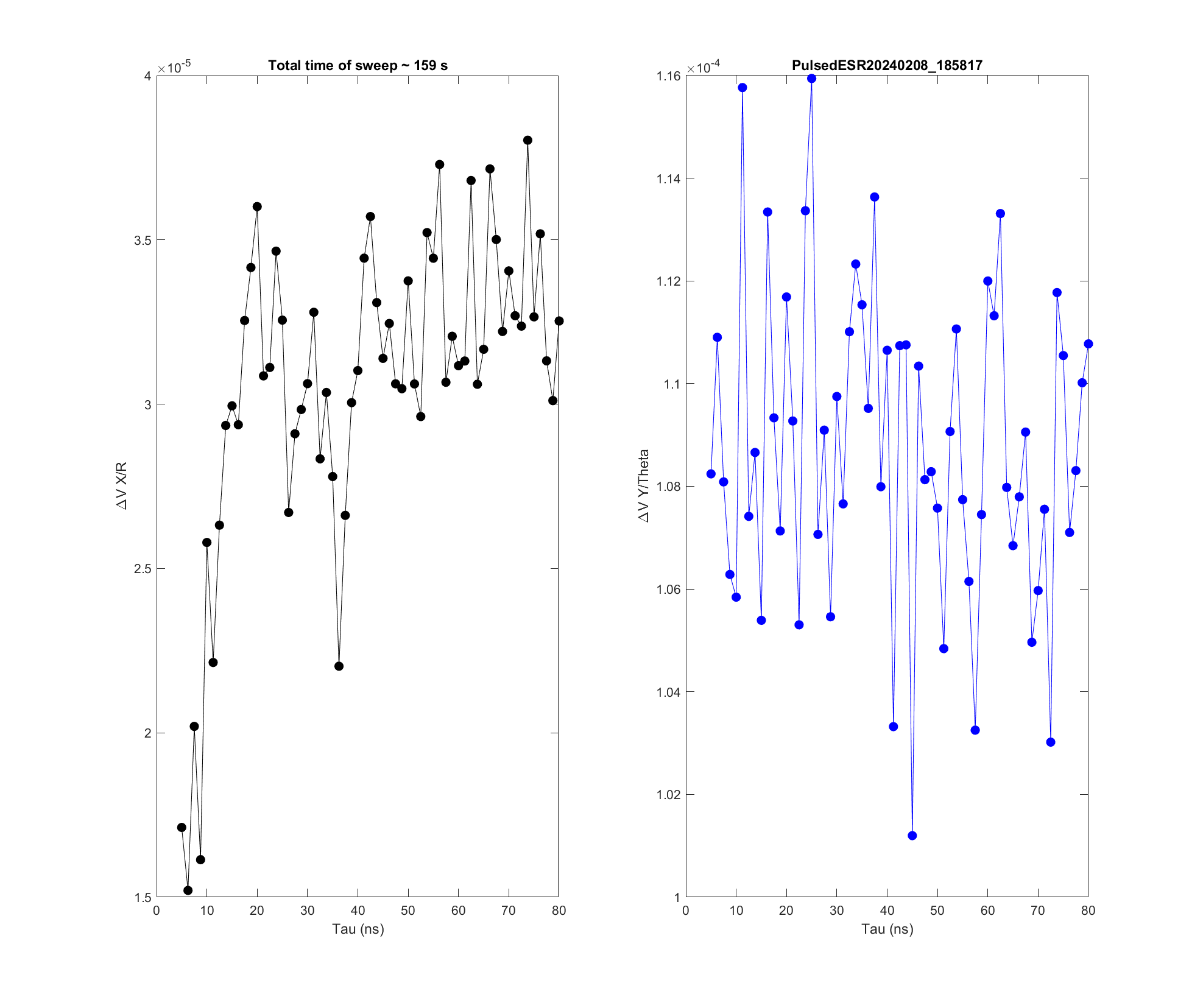

Supplement: Supplementary file 3 — Source Data [file 41467_2025_60409_MOESM3_ESM.zip › SupplementaryData1/Figure3/Fig3d/Ramsey/PulsedESR20240208_185817.png]

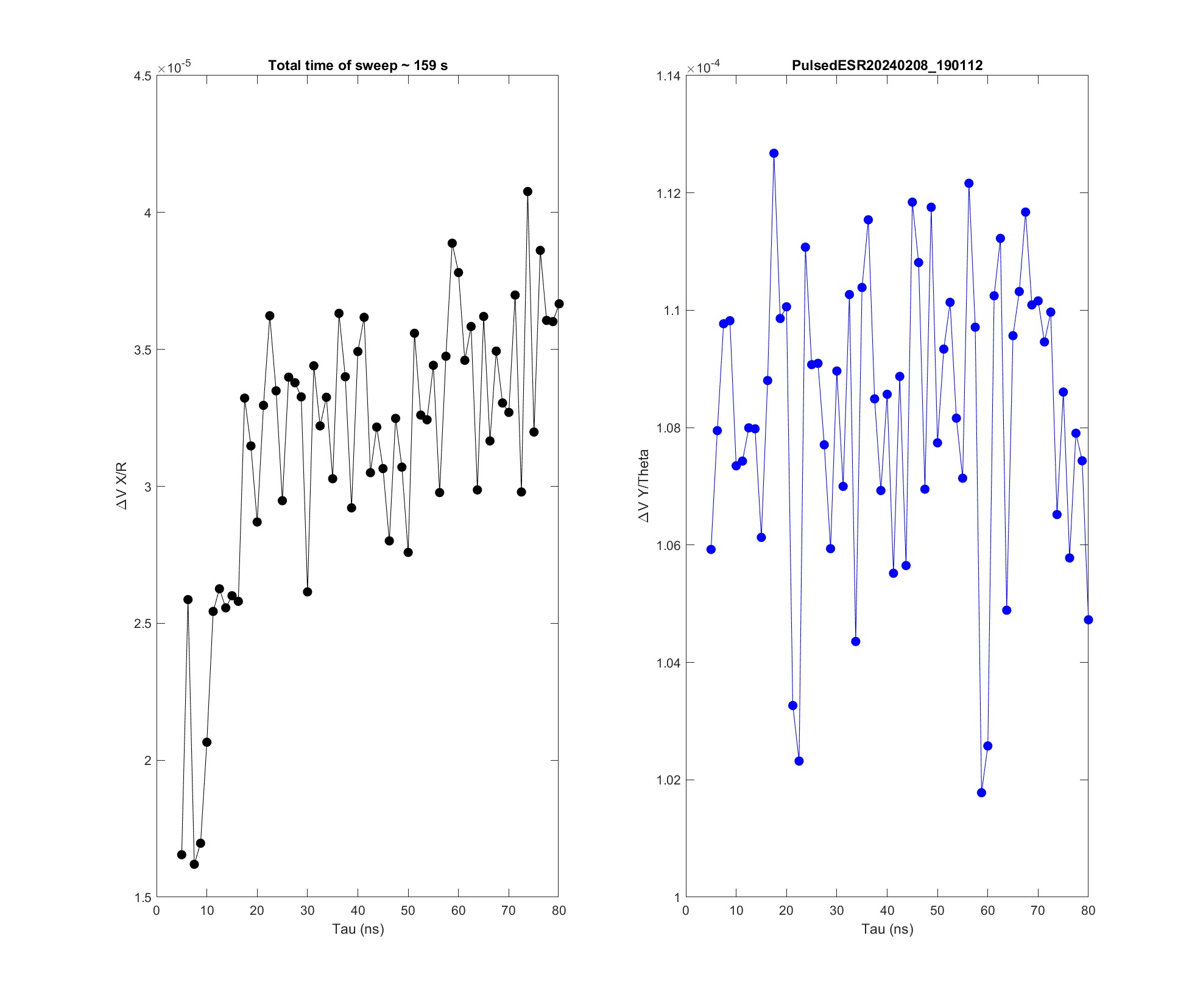

Supplement: Supplementary file 3 — Source Data [file 41467_2025_60409_MOESM3_ESM.zip › SupplementaryData1/Figure3/Fig3d/Ramsey/PulsedESR20240208_190112.png]

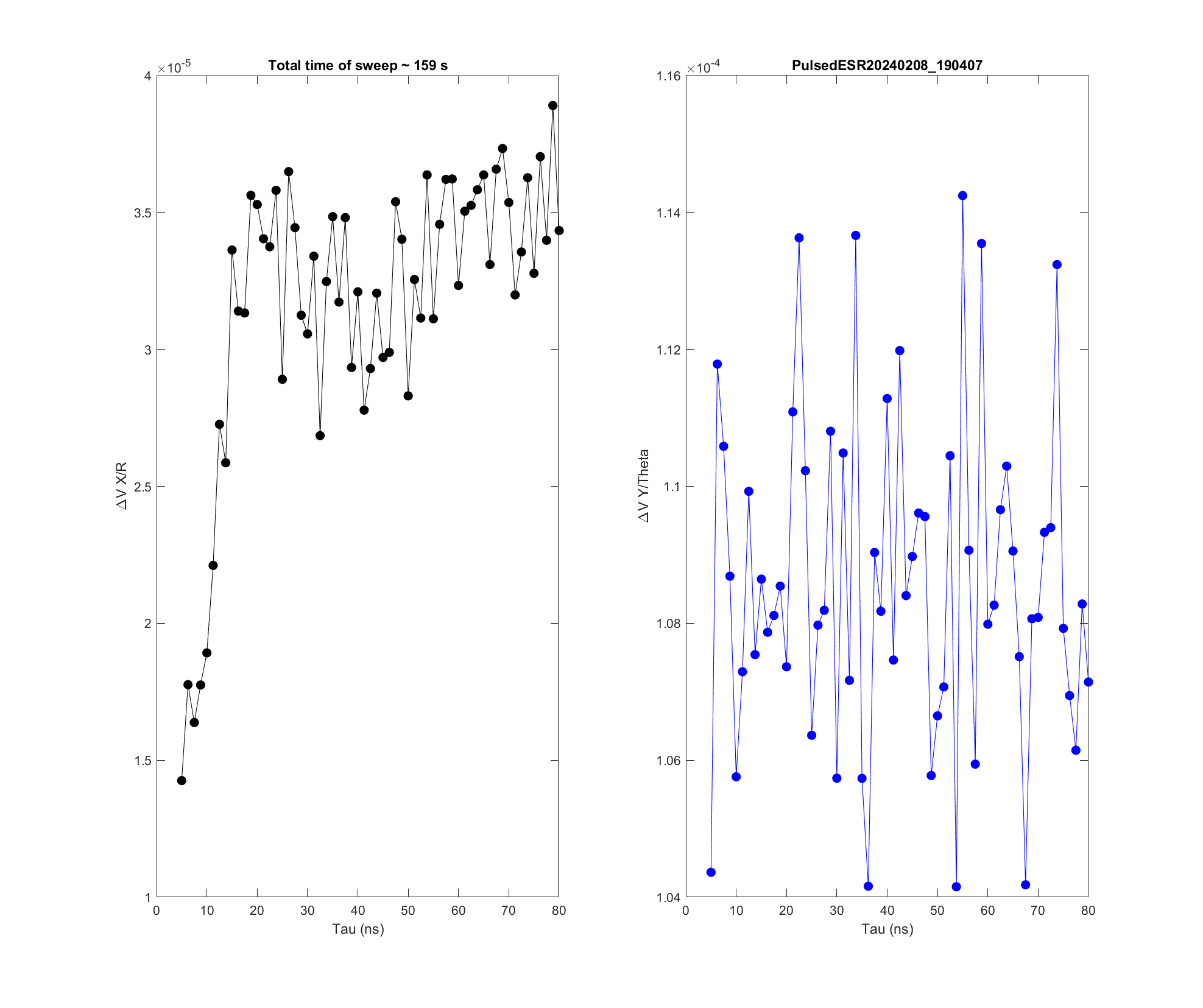

Supplement: Supplementary file 3 — Source Data [file 41467_2025_60409_MOESM3_ESM.zip › SupplementaryData1/Figure3/Fig3d/Ramsey/PulsedESR20240208_190407.png]

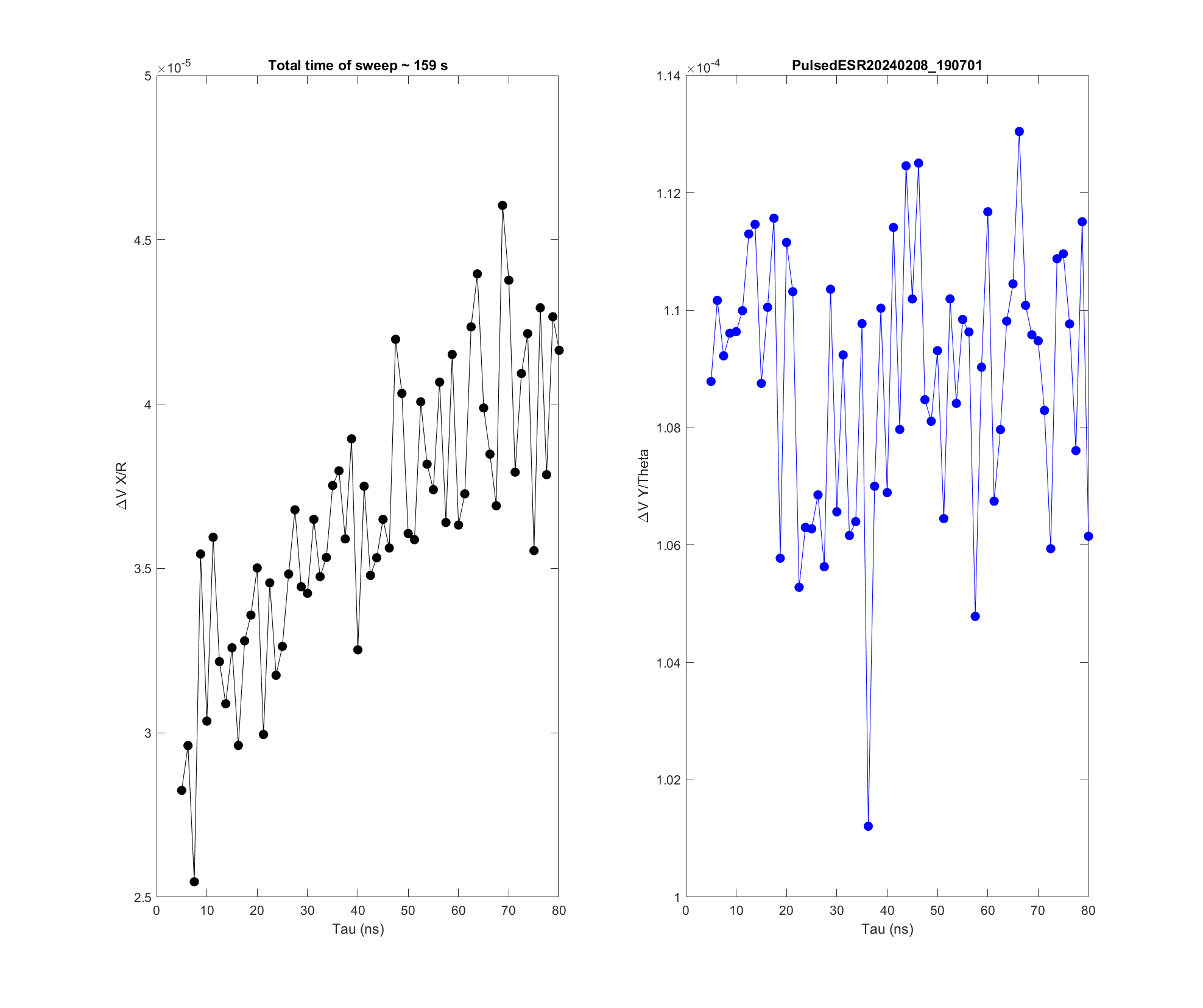

Supplement: Supplementary file 3 — Source Data [file 41467_2025_60409_MOESM3_ESM.zip › SupplementaryData1/Figure3/Fig3d/Ramsey/PulsedESR20240208_190701.png]

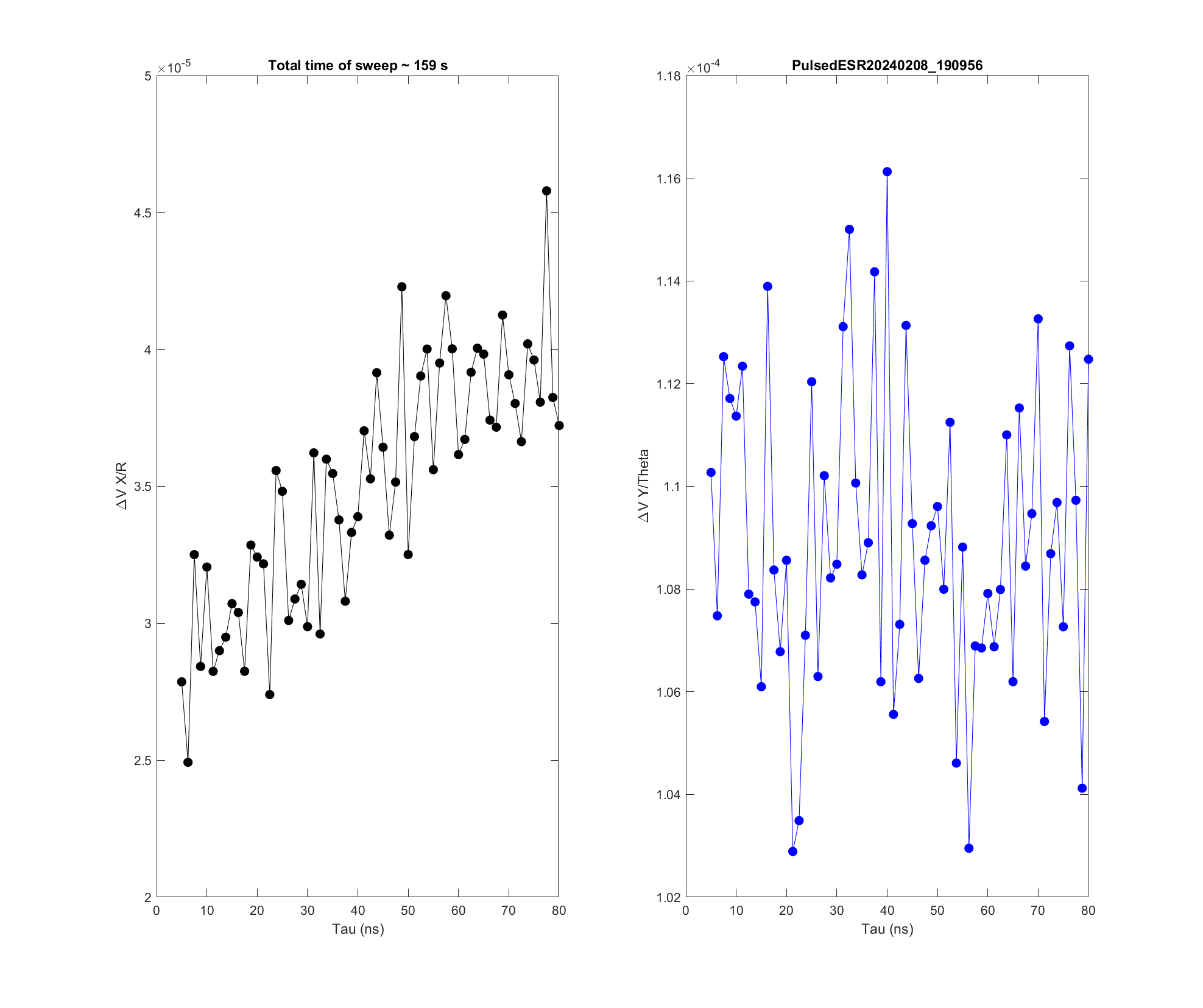

Supplement: Supplementary file 3 — Source Data [file 41467_2025_60409_MOESM3_ESM.zip › SupplementaryData1/Figure3/Fig3d/Ramsey/PulsedESR20240208_190956.png]

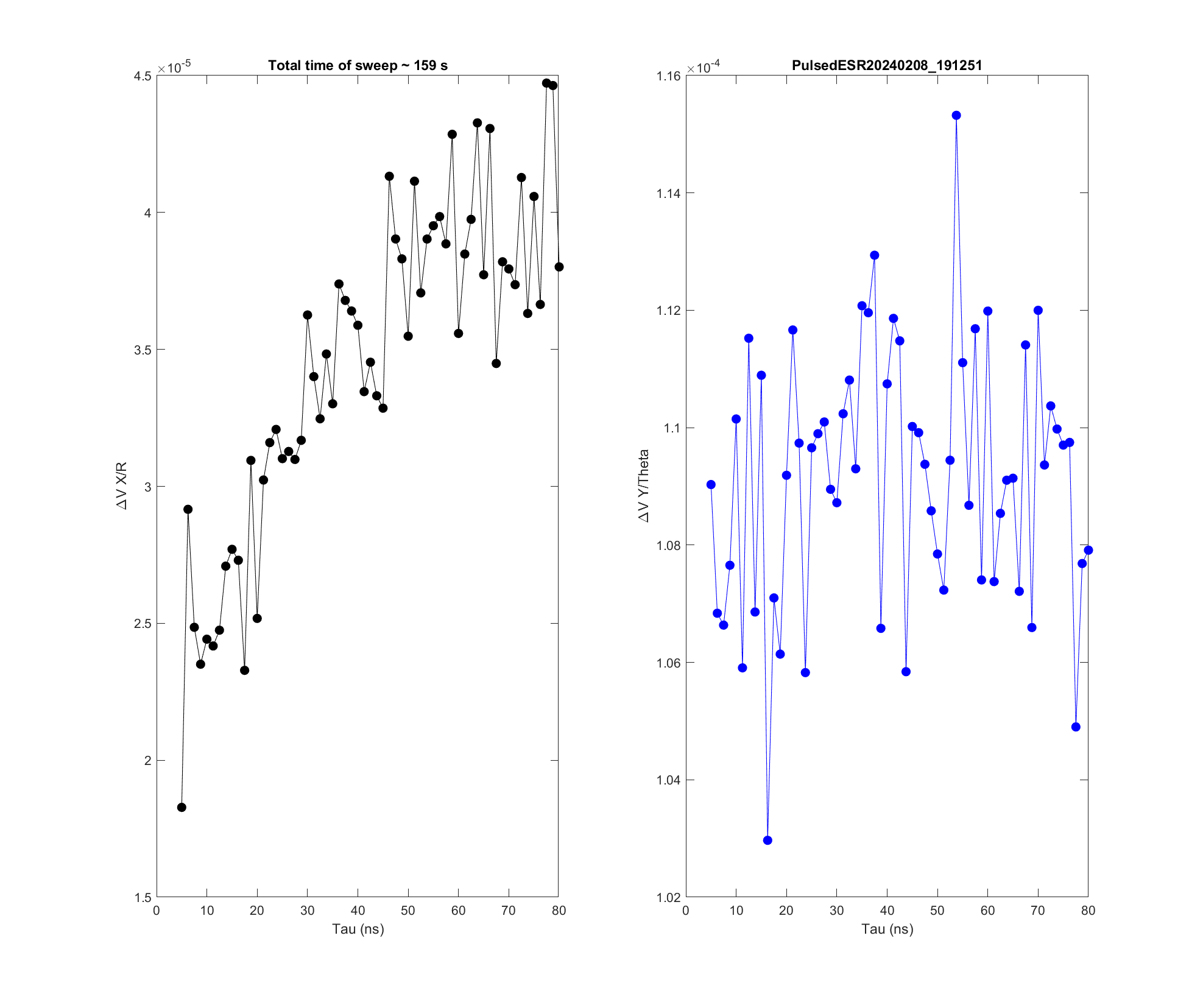

Supplement: Supplementary file 3 — Source Data [file 41467_2025_60409_MOESM3_ESM.zip › SupplementaryData1/Figure3/Fig3d/Ramsey/PulsedESR20240208_191251.png]

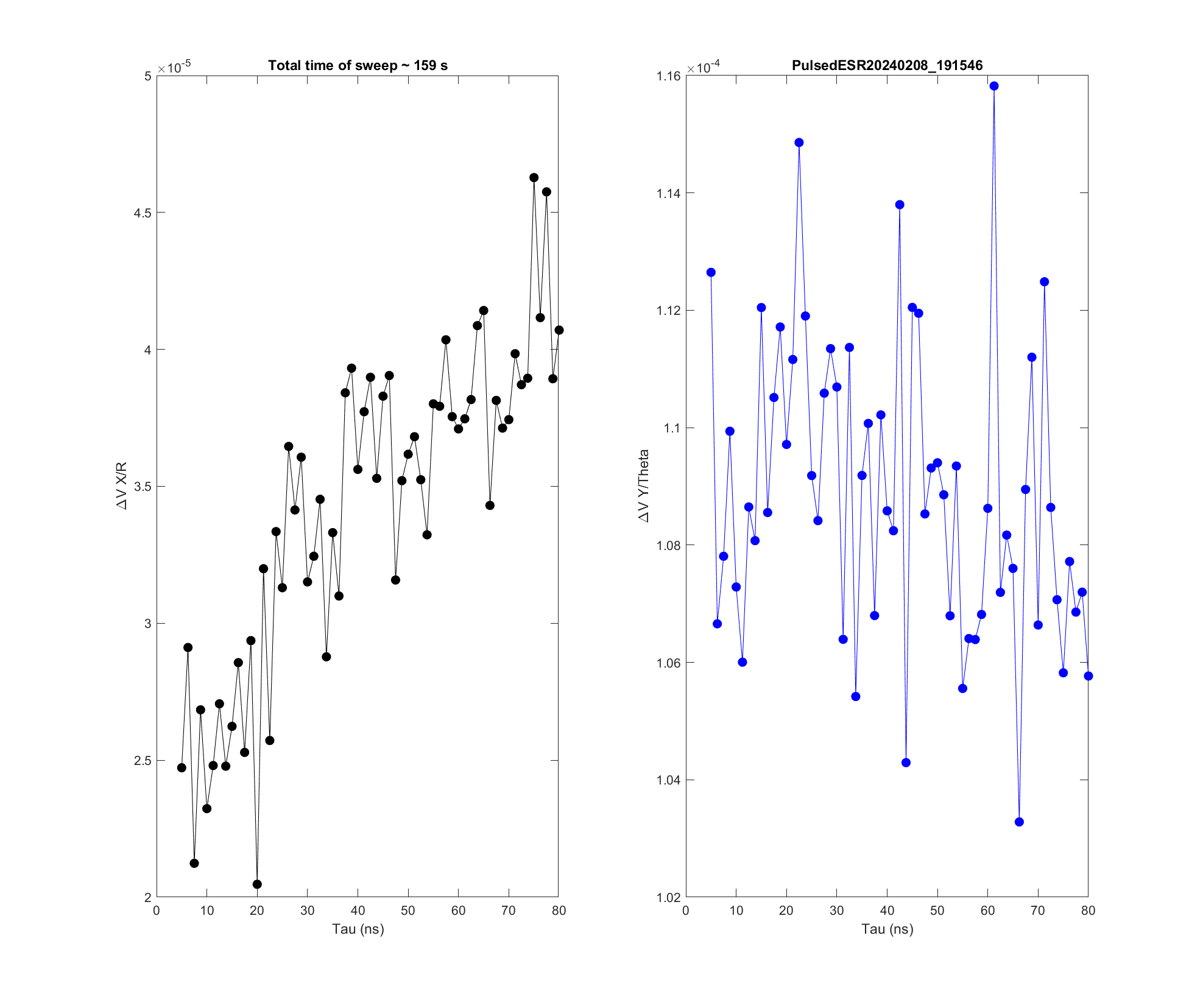

Supplement: Supplementary file 3 — Source Data [file 41467_2025_60409_MOESM3_ESM.zip › SupplementaryData1/Figure3/Fig3d/Ramsey/PulsedESR20240208_191546.png]

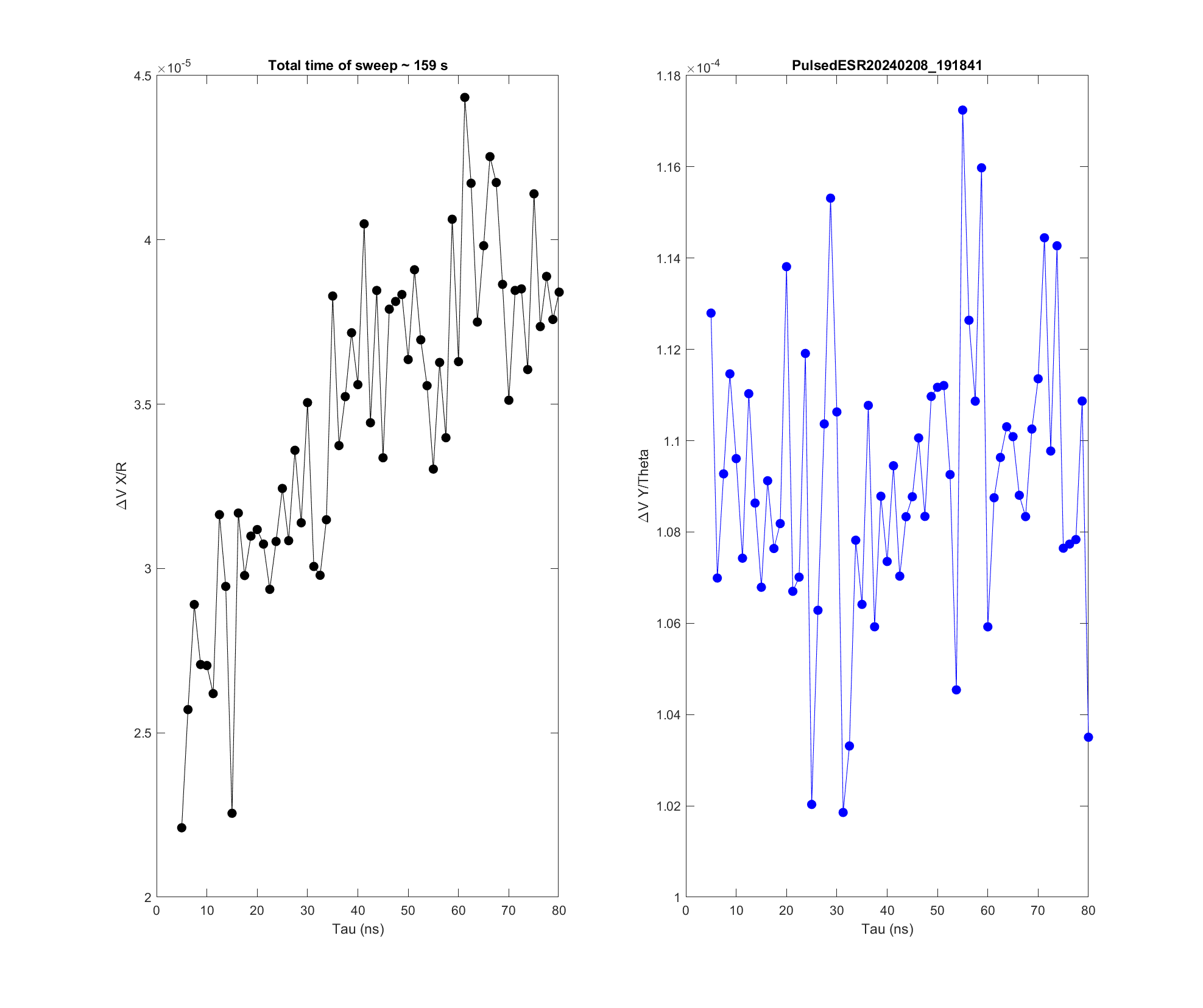

Supplement: Supplementary file 3 — Source Data [file 41467_2025_60409_MOESM3_ESM.zip › SupplementaryData1/Figure3/Fig3d/Ramsey/PulsedESR20240208_191841.png]

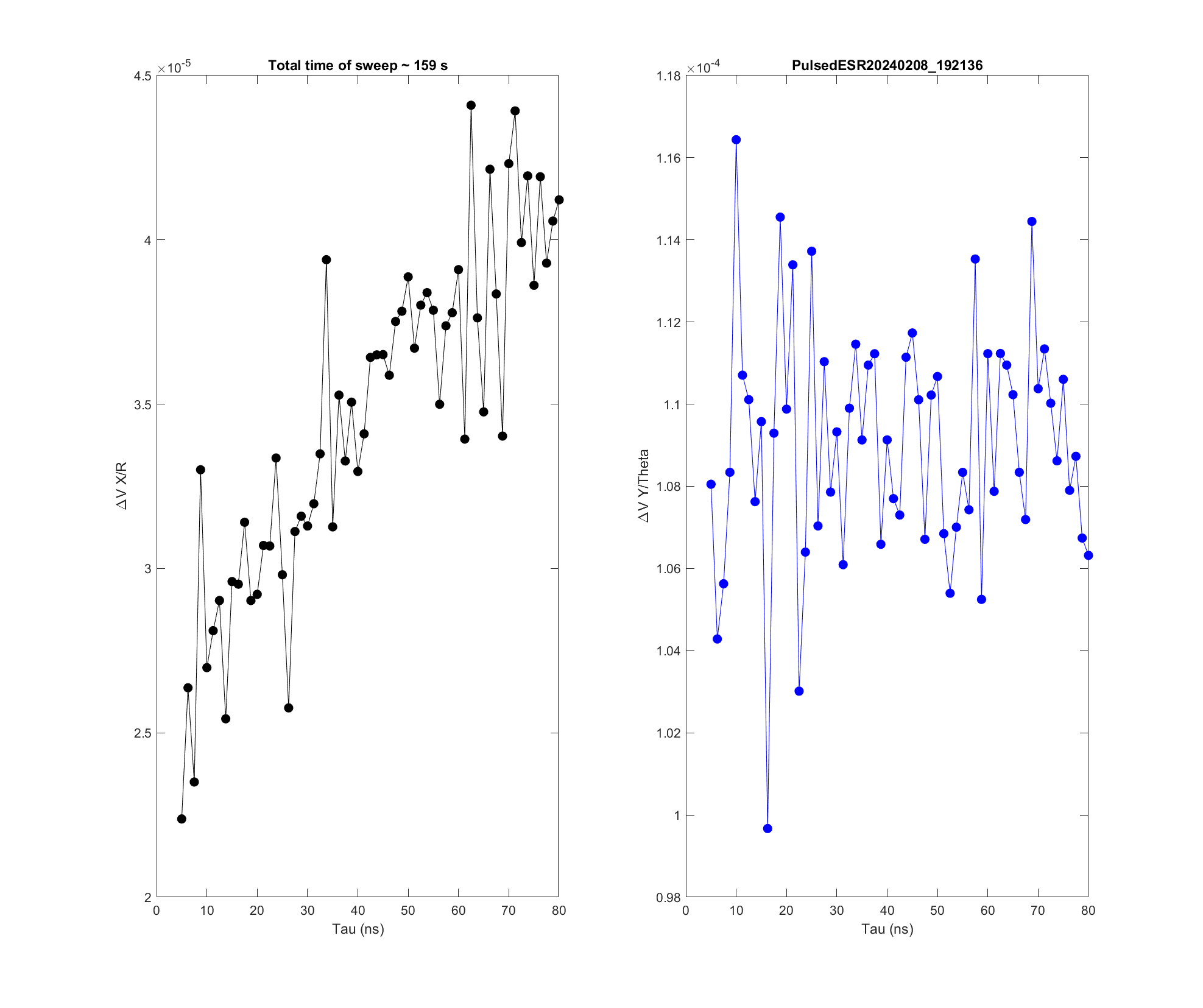

Supplement: Supplementary file 3 — Source Data [file 41467_2025_60409_MOESM3_ESM.zip › SupplementaryData1/Figure3/Fig3d/Ramsey/PulsedESR20240208_192136.png]

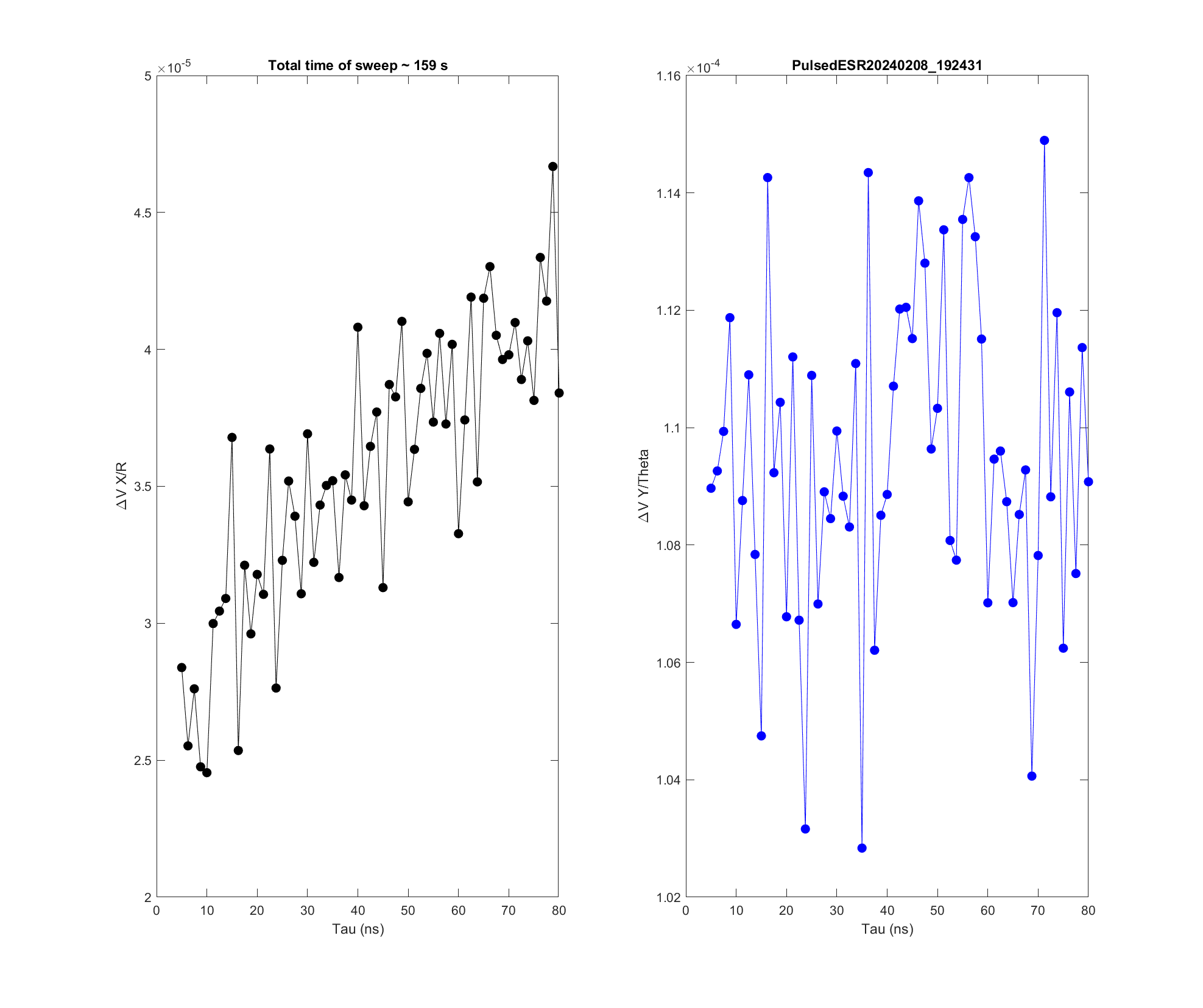

Supplement: Supplementary file 3 — Source Data [file 41467_2025_60409_MOESM3_ESM.zip › SupplementaryData1/Figure3/Fig3d/Ramsey/PulsedESR20240208_192431.png]

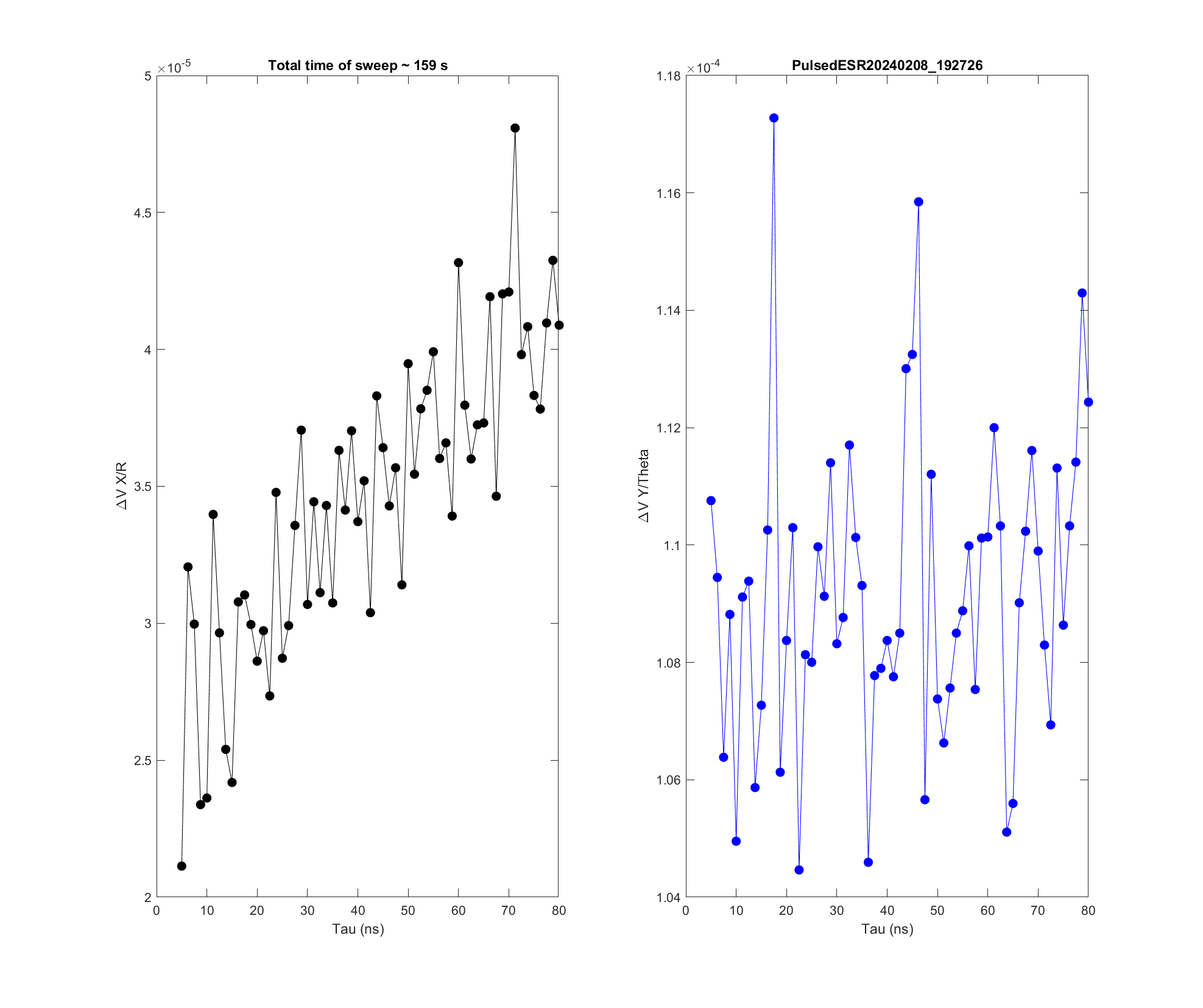

Supplement: Supplementary file 3 — Source Data [file 41467_2025_60409_MOESM3_ESM.zip › SupplementaryData1/Figure3/Fig3d/Ramsey/PulsedESR20240208_192726.png]

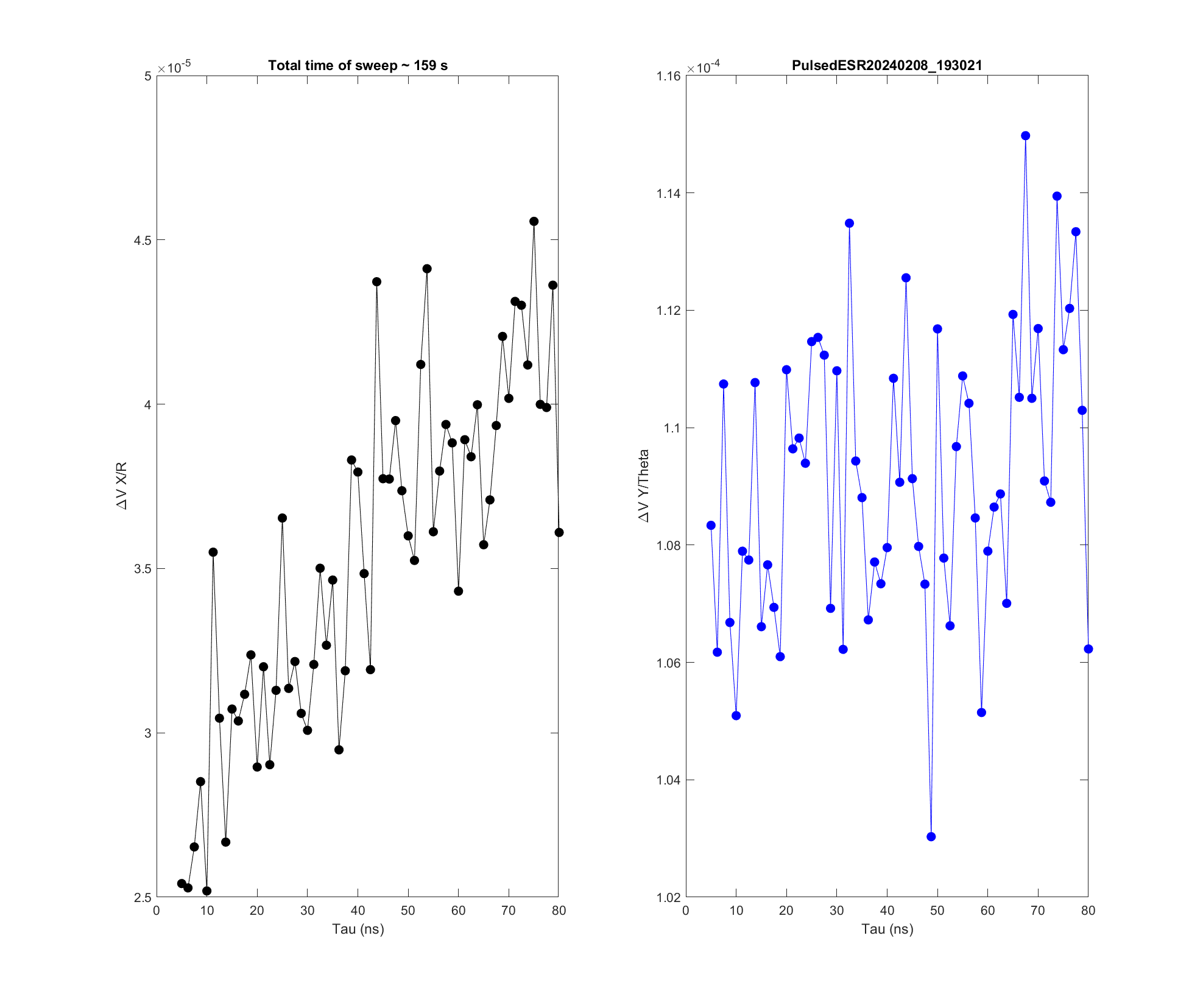

Supplement: Supplementary file 3 — Source Data [file 41467_2025_60409_MOESM3_ESM.zip › SupplementaryData1/Figure3/Fig3d/Ramsey/PulsedESR20240208_193021.png]

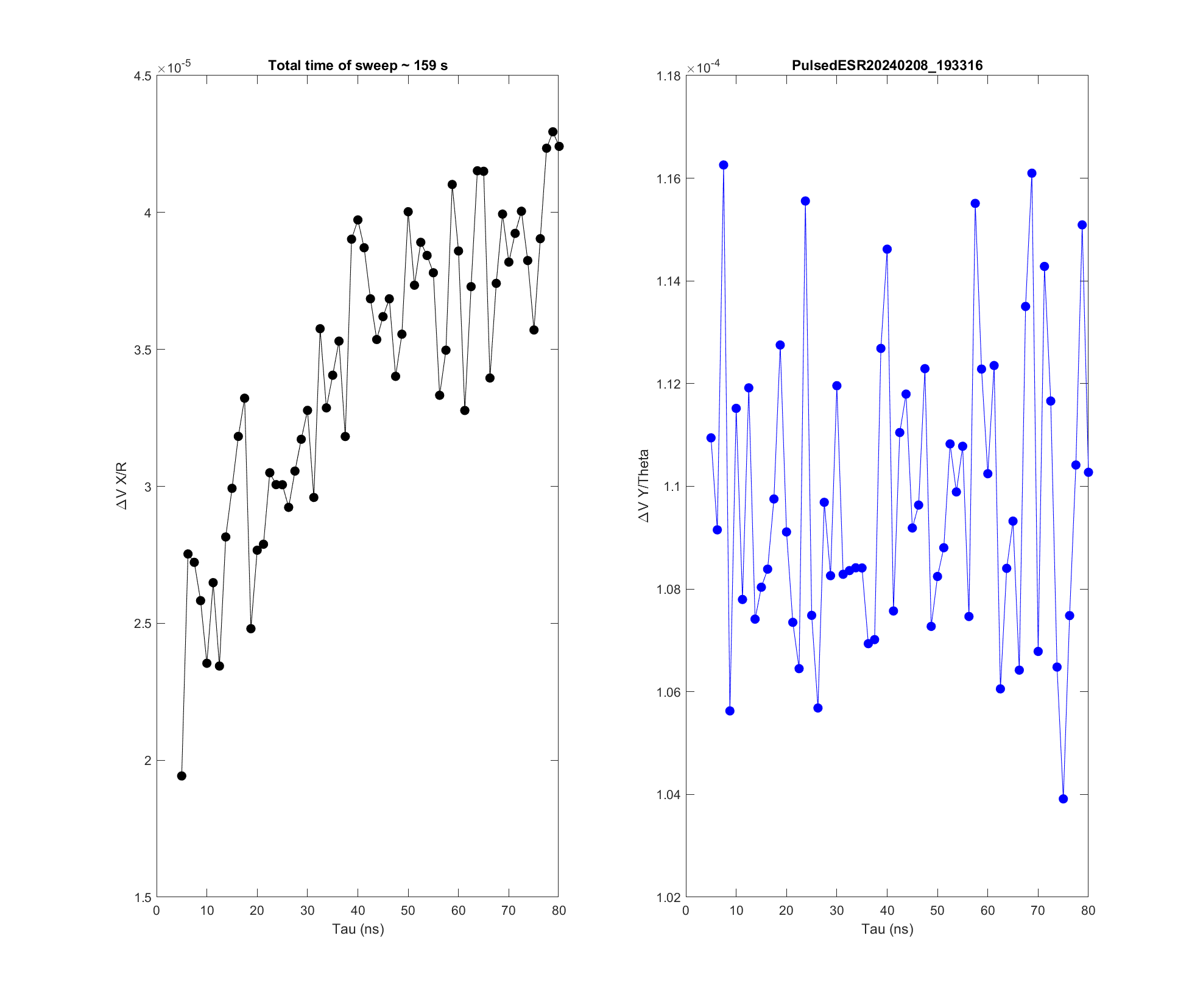

Supplement: Supplementary file 3 — Source Data [file 41467_2025_60409_MOESM3_ESM.zip › SupplementaryData1/Figure3/Fig3d/Ramsey/PulsedESR20240208_193316.png]

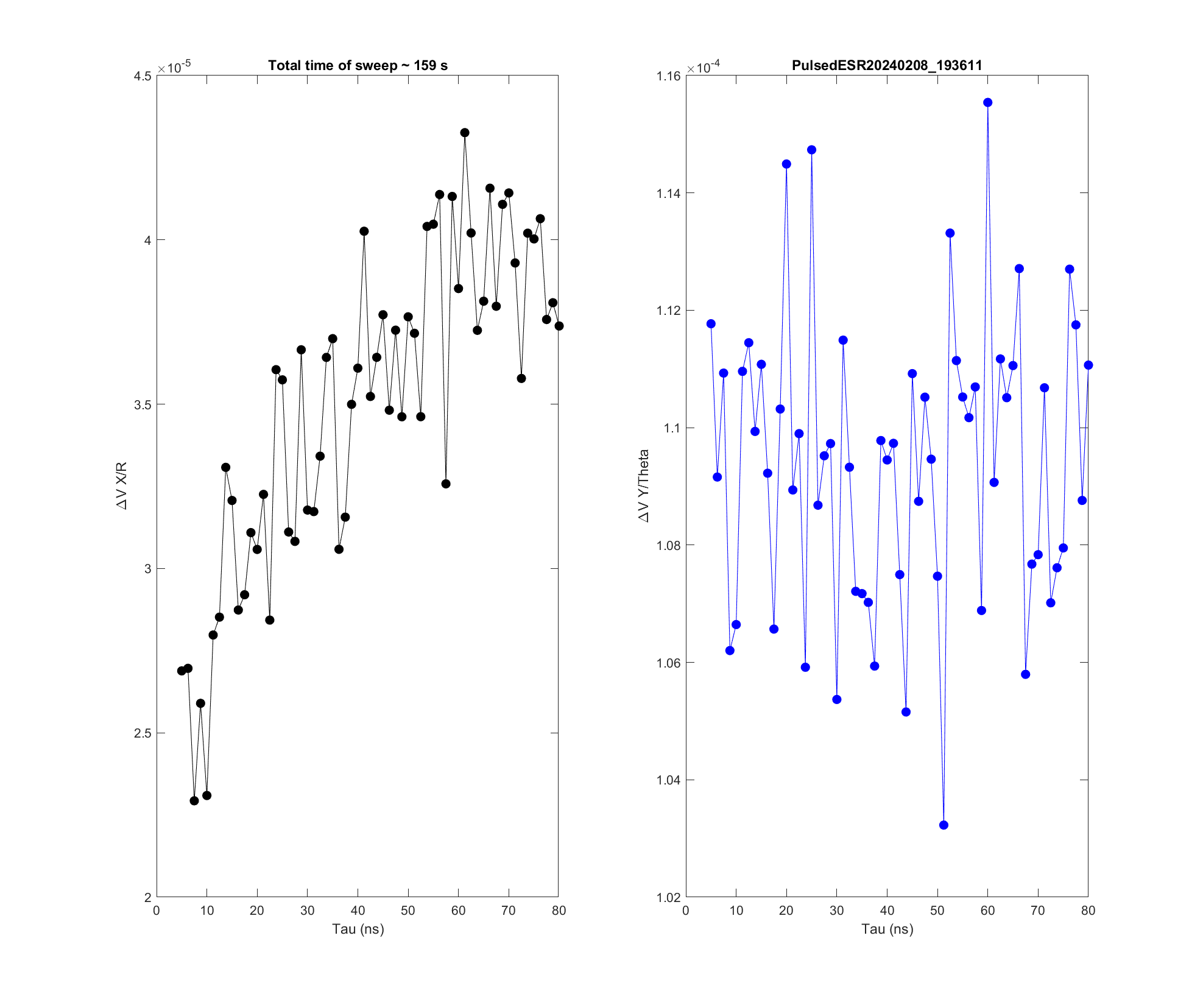

Supplement: Supplementary file 3 — Source Data [file 41467_2025_60409_MOESM3_ESM.zip › SupplementaryData1/Figure3/Fig3d/Ramsey/PulsedESR20240208_193611.png]

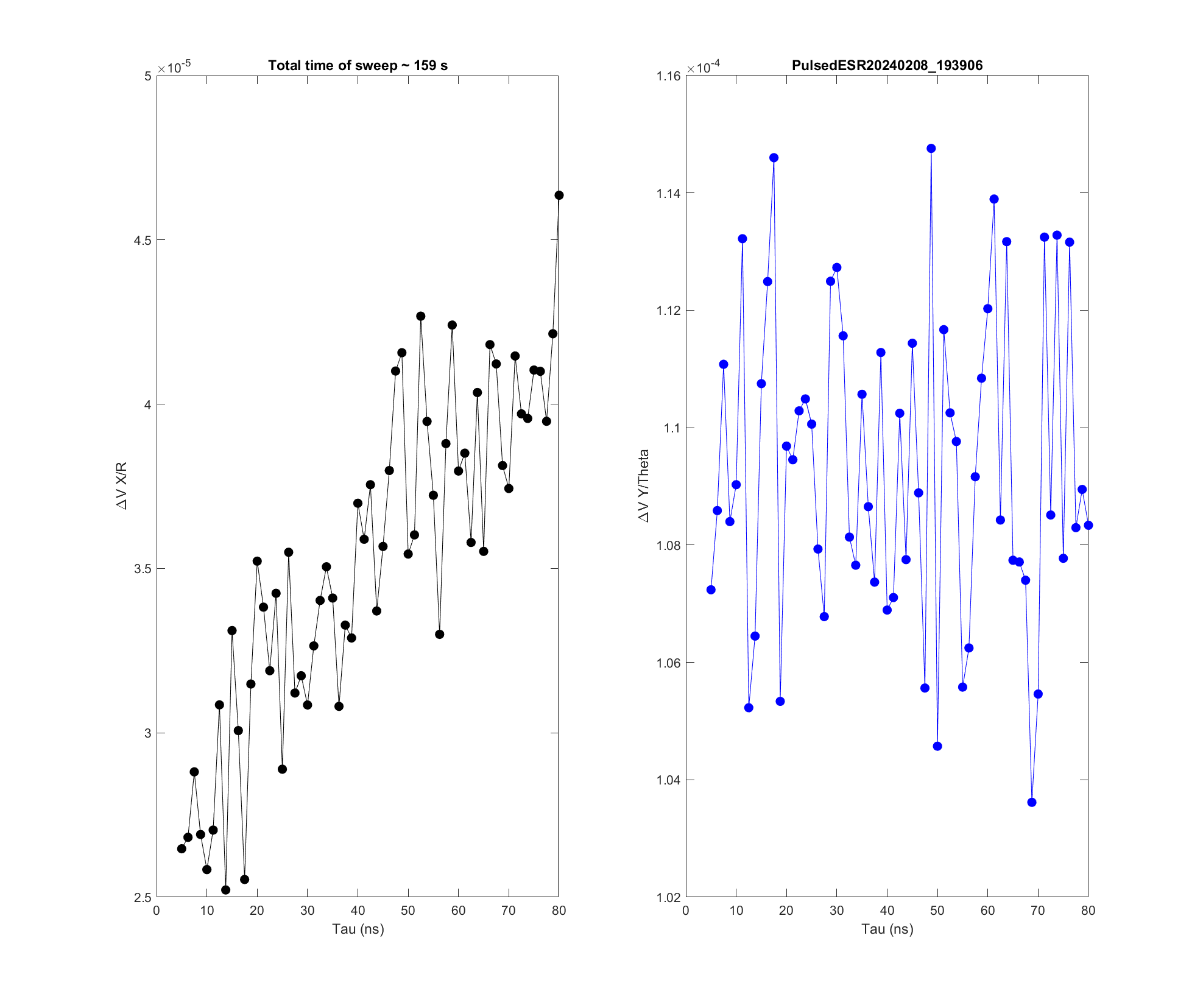

Supplement: Supplementary file 3 — Source Data [file 41467_2025_60409_MOESM3_ESM.zip › SupplementaryData1/Figure3/Fig3d/Ramsey/PulsedESR20240208_193906.png]

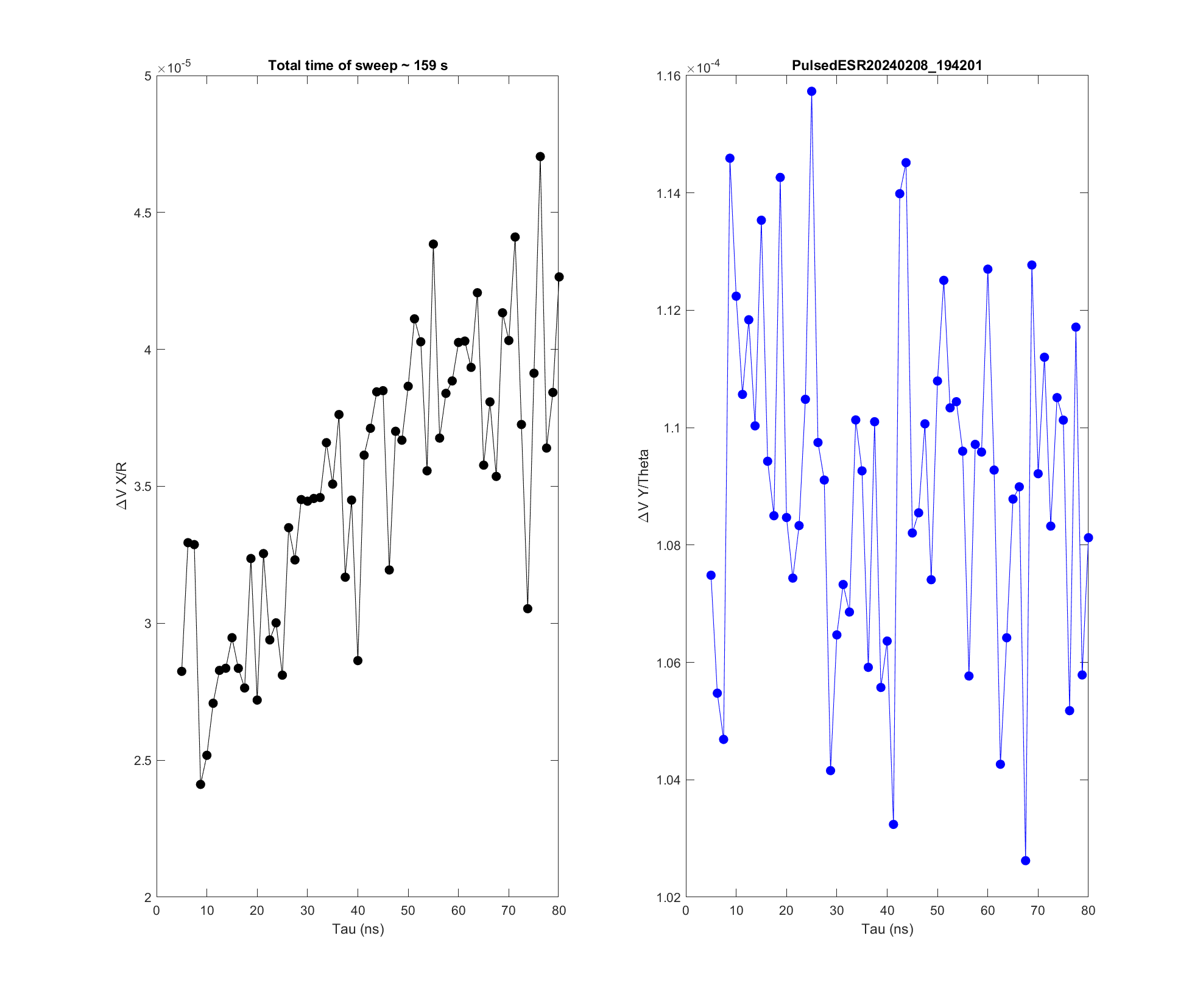

Supplement: Supplementary file 3 — Source Data [file 41467_2025_60409_MOESM3_ESM.zip › SupplementaryData1/Figure3/Fig3d/Ramsey/PulsedESR20240208_194201.png]

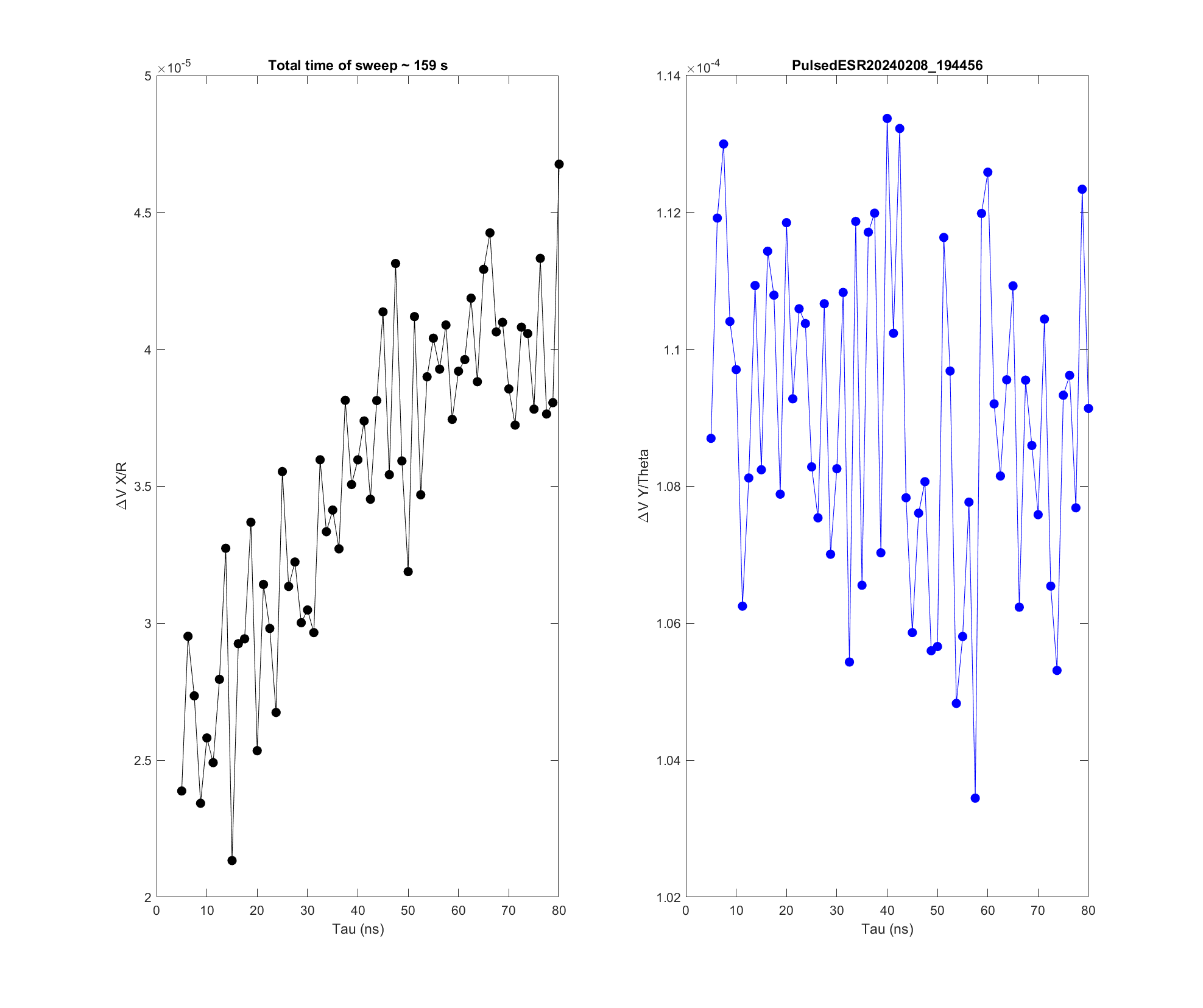

Supplement: Supplementary file 3 — Source Data [file 41467_2025_60409_MOESM3_ESM.zip › SupplementaryData1/Figure3/Fig3d/Ramsey/PulsedESR20240208_194456.png]

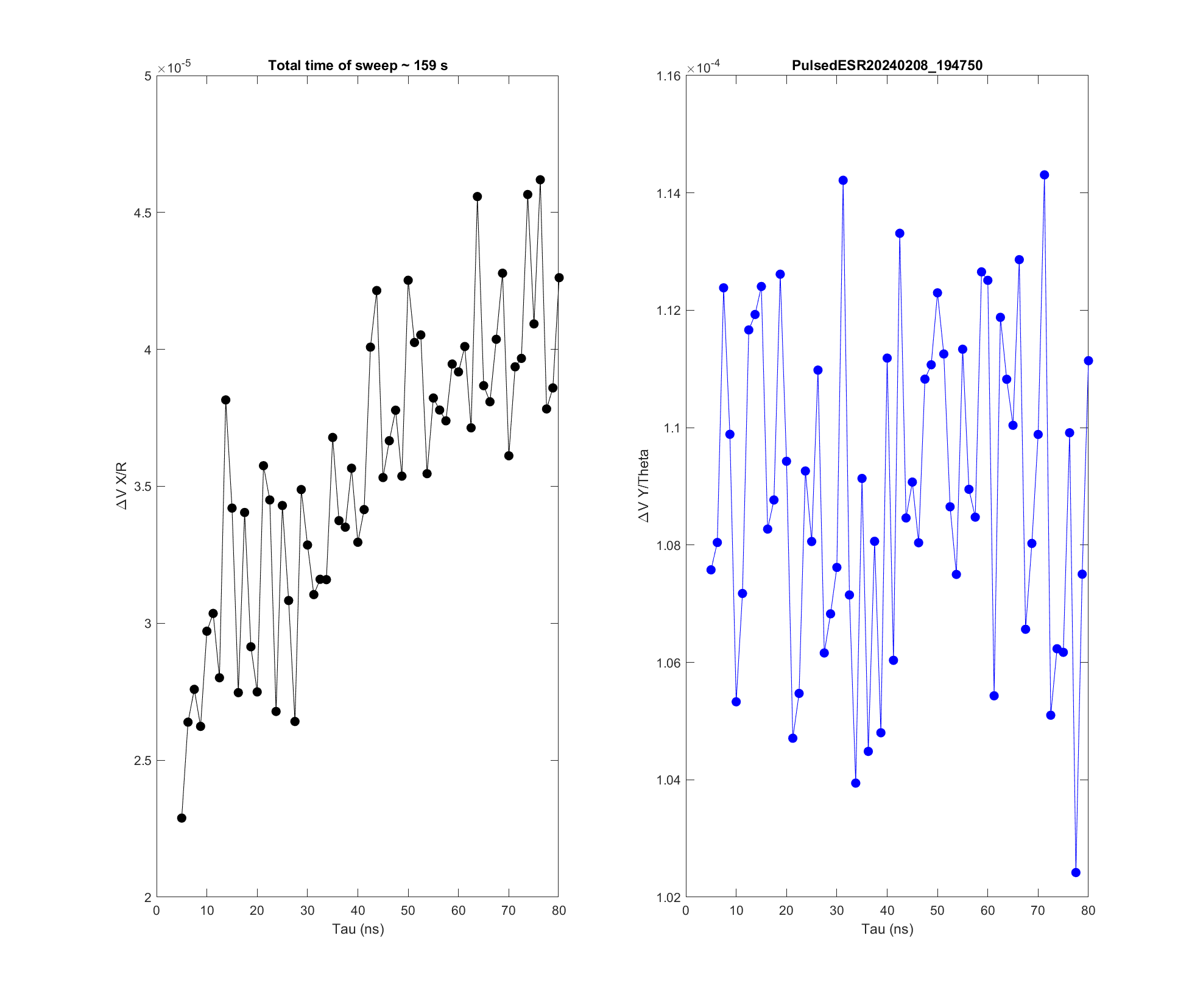

Supplement: Supplementary file 3 — Source Data [file 41467_2025_60409_MOESM3_ESM.zip › SupplementaryData1/Figure3/Fig3d/Ramsey/PulsedESR20240208_194750.png]

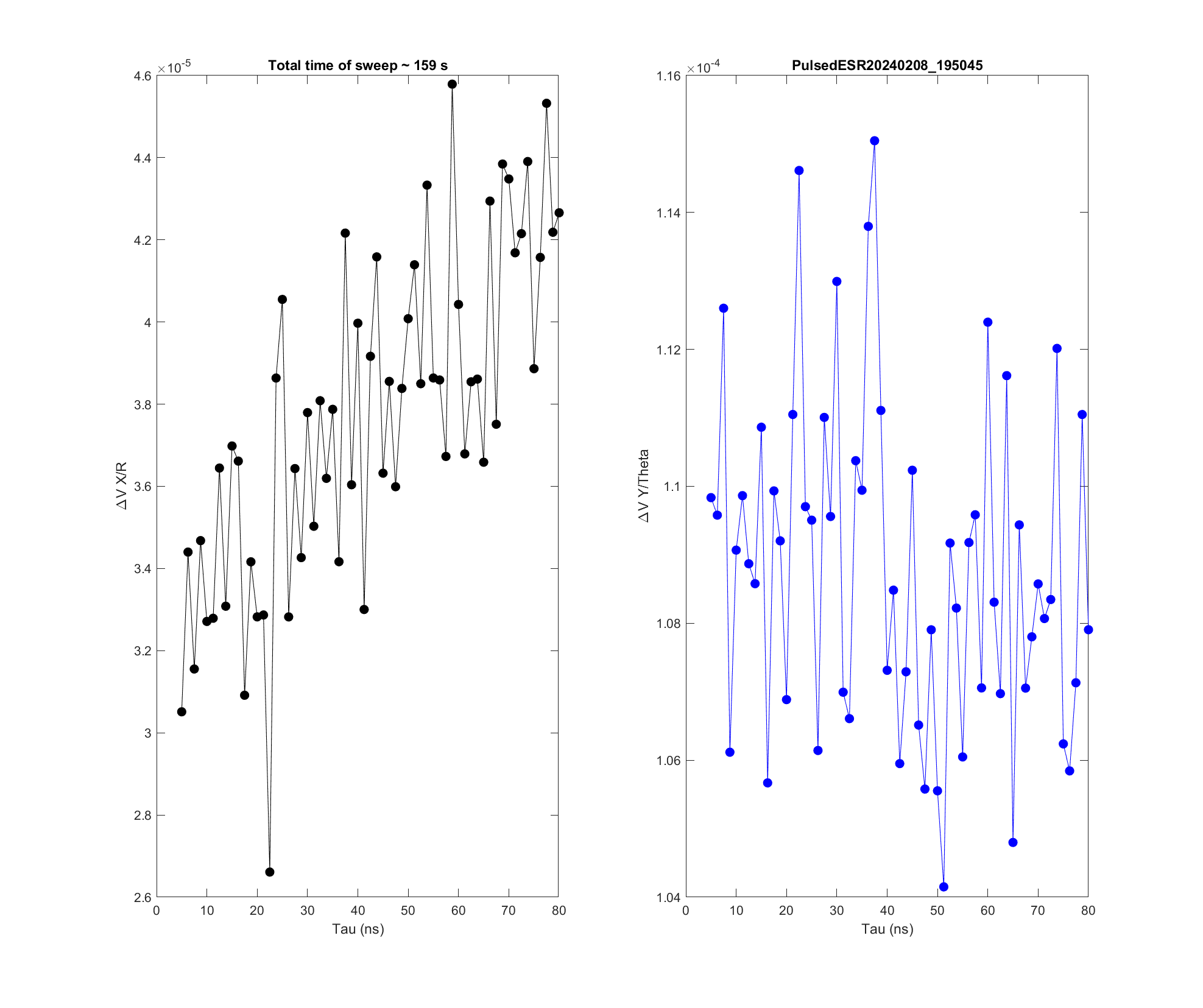

Supplement: Supplementary file 3 — Source Data [file 41467_2025_60409_MOESM3_ESM.zip › SupplementaryData1/Figure3/Fig3d/Ramsey/PulsedESR20240208_195045.png]

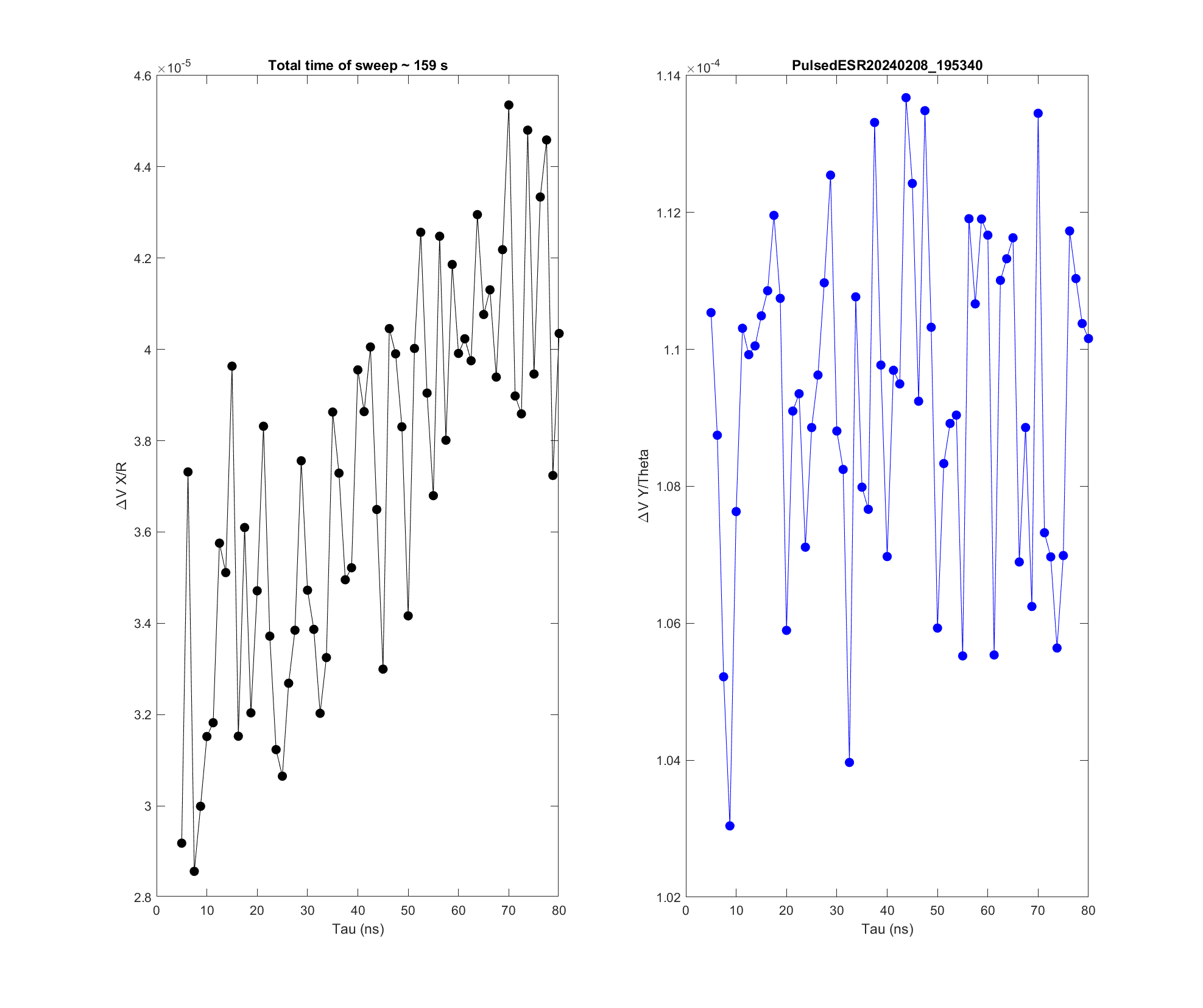

Supplement: Supplementary file 3 — Source Data [file 41467_2025_60409_MOESM3_ESM.zip › SupplementaryData1/Figure3/Fig3d/Ramsey/PulsedESR20240208_195340.png]

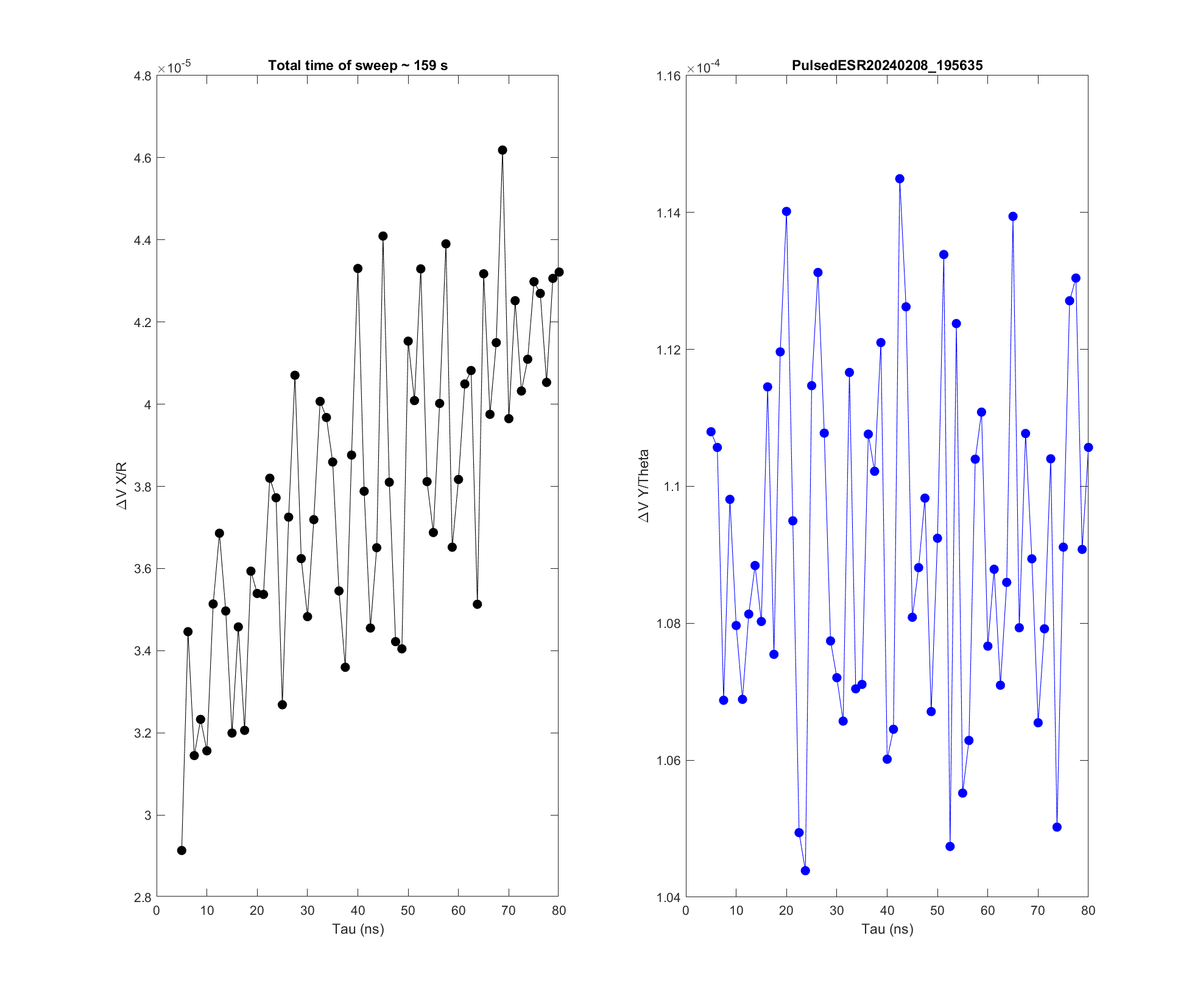

Supplement: Supplementary file 3 — Source Data [file 41467_2025_60409_MOESM3_ESM.zip › SupplementaryData1/Figure3/Fig3d/Ramsey/PulsedESR20240208_195635.png]

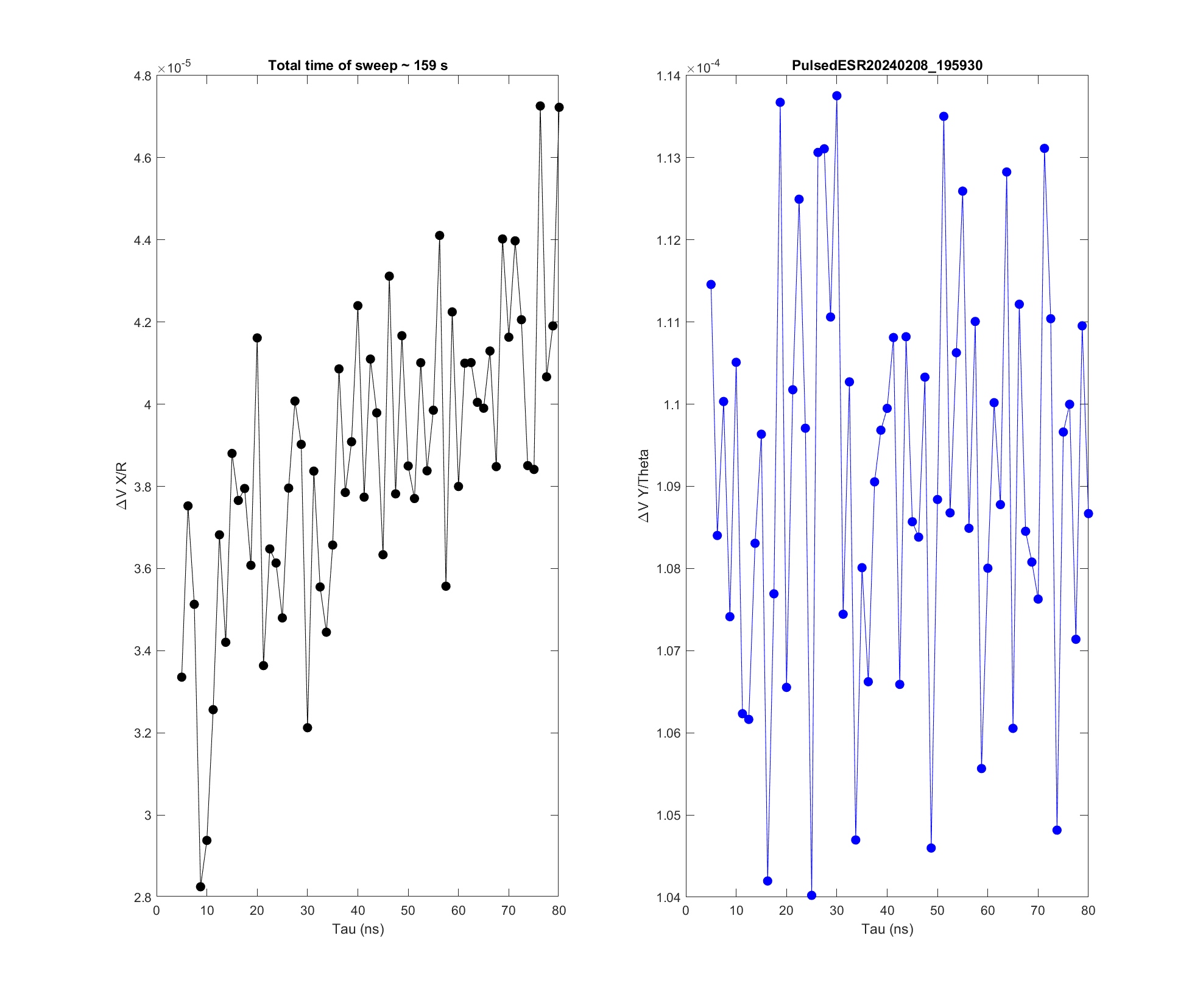

Supplement: Supplementary file 3 — Source Data [file 41467_2025_60409_MOESM3_ESM.zip › SupplementaryData1/Figure3/Fig3d/Ramsey/PulsedESR20240208_195930.png]

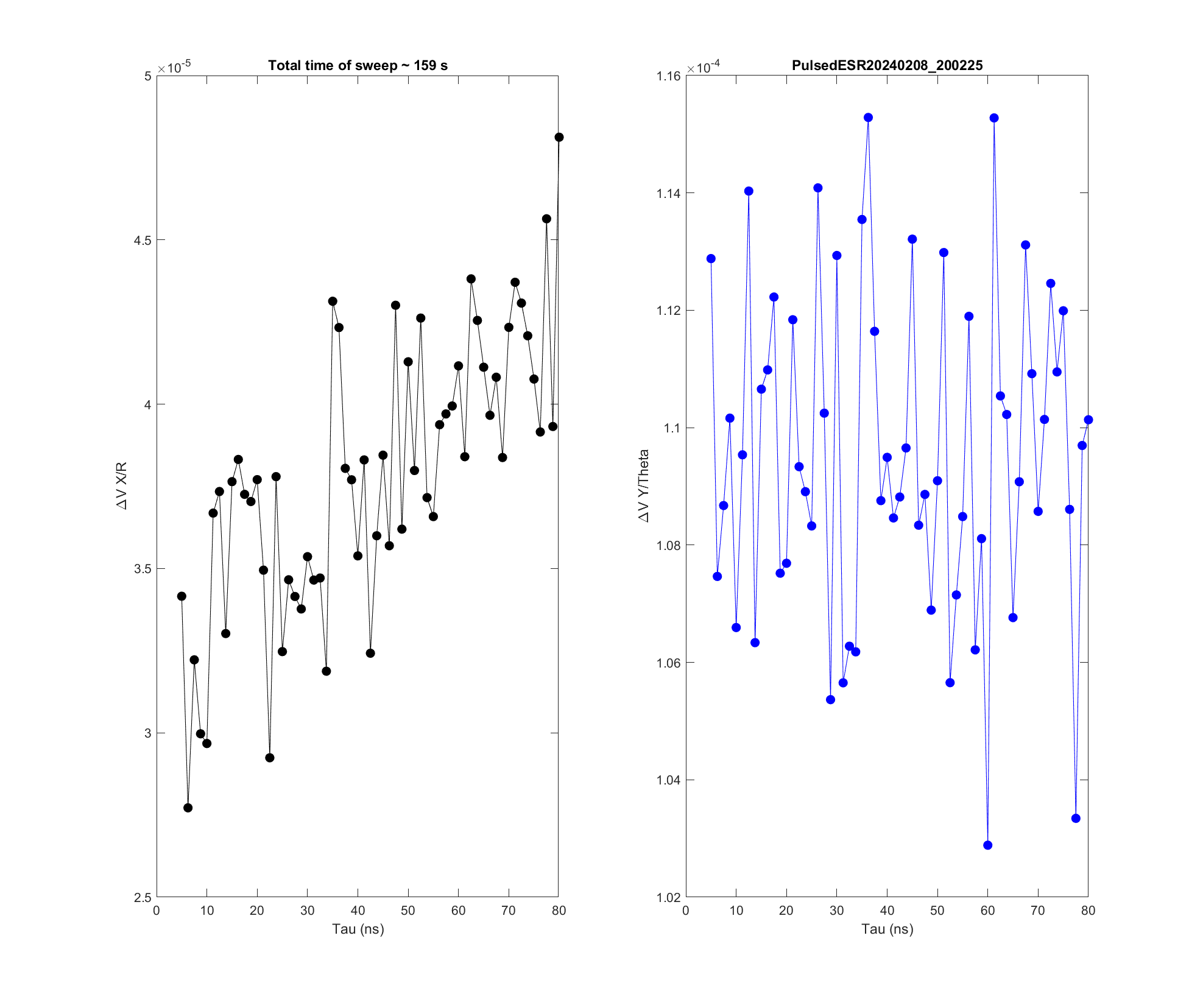

Supplement: Supplementary file 3 — Source Data [file 41467_2025_60409_MOESM3_ESM.zip › SupplementaryData1/Figure3/Fig3d/Ramsey/PulsedESR20240208_200225.png]

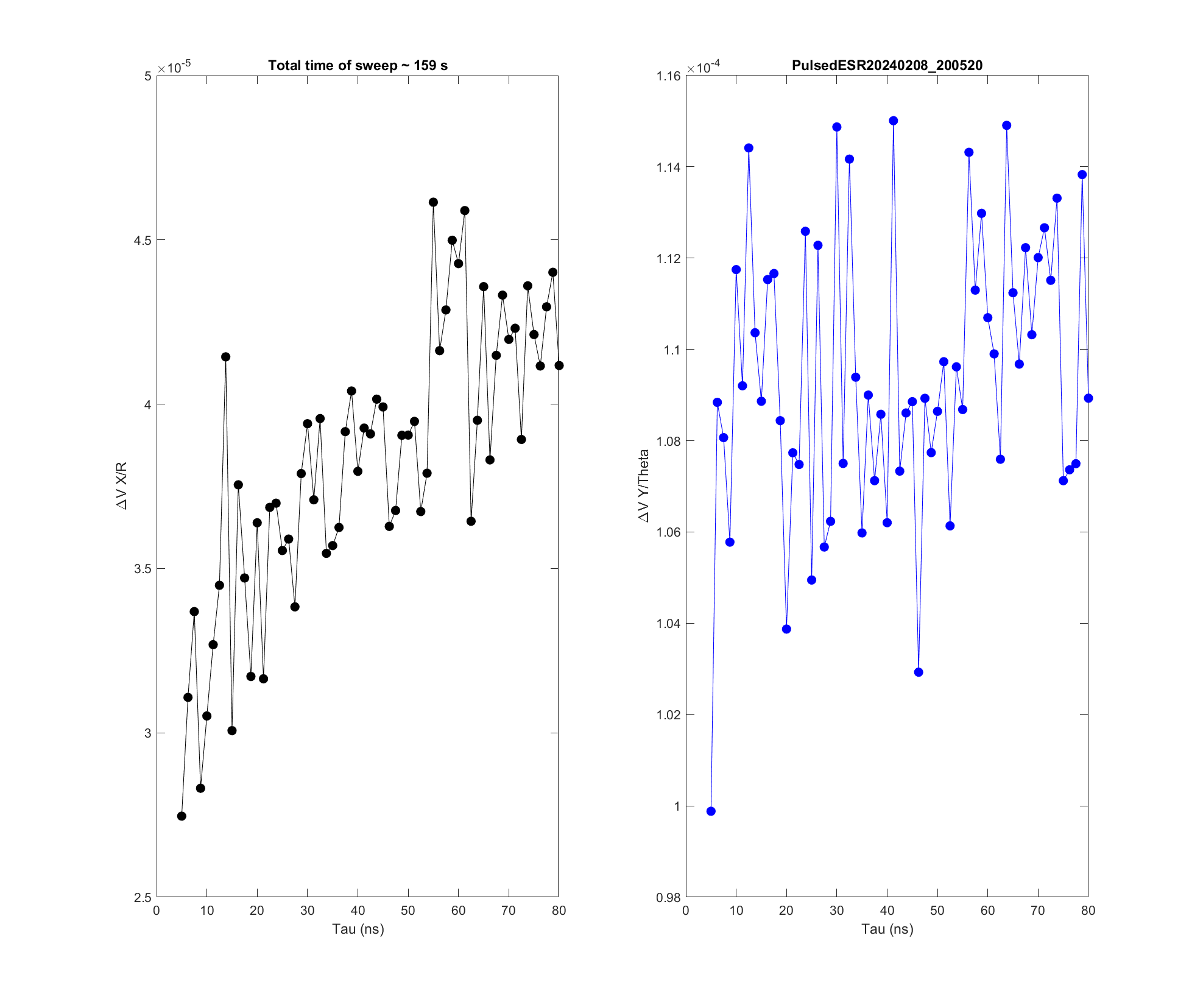

Supplement: Supplementary file 3 — Source Data [file 41467_2025_60409_MOESM3_ESM.zip › SupplementaryData1/Figure3/Fig3d/Ramsey/PulsedESR20240208_200520.png]

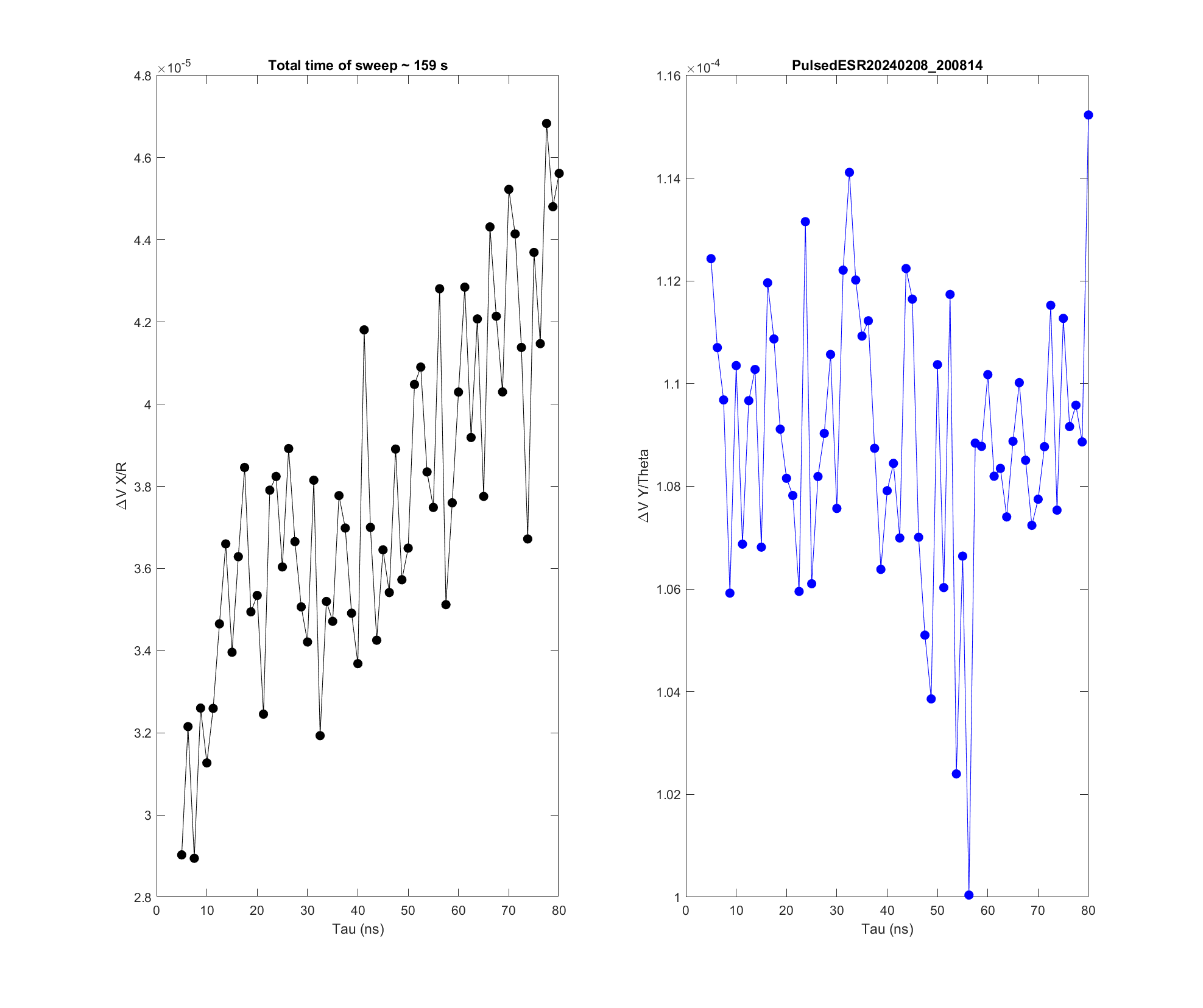

Supplement: Supplementary file 3 — Source Data [file 41467_2025_60409_MOESM3_ESM.zip › SupplementaryData1/Figure3/Fig3d/Ramsey/PulsedESR20240208_200814.png]

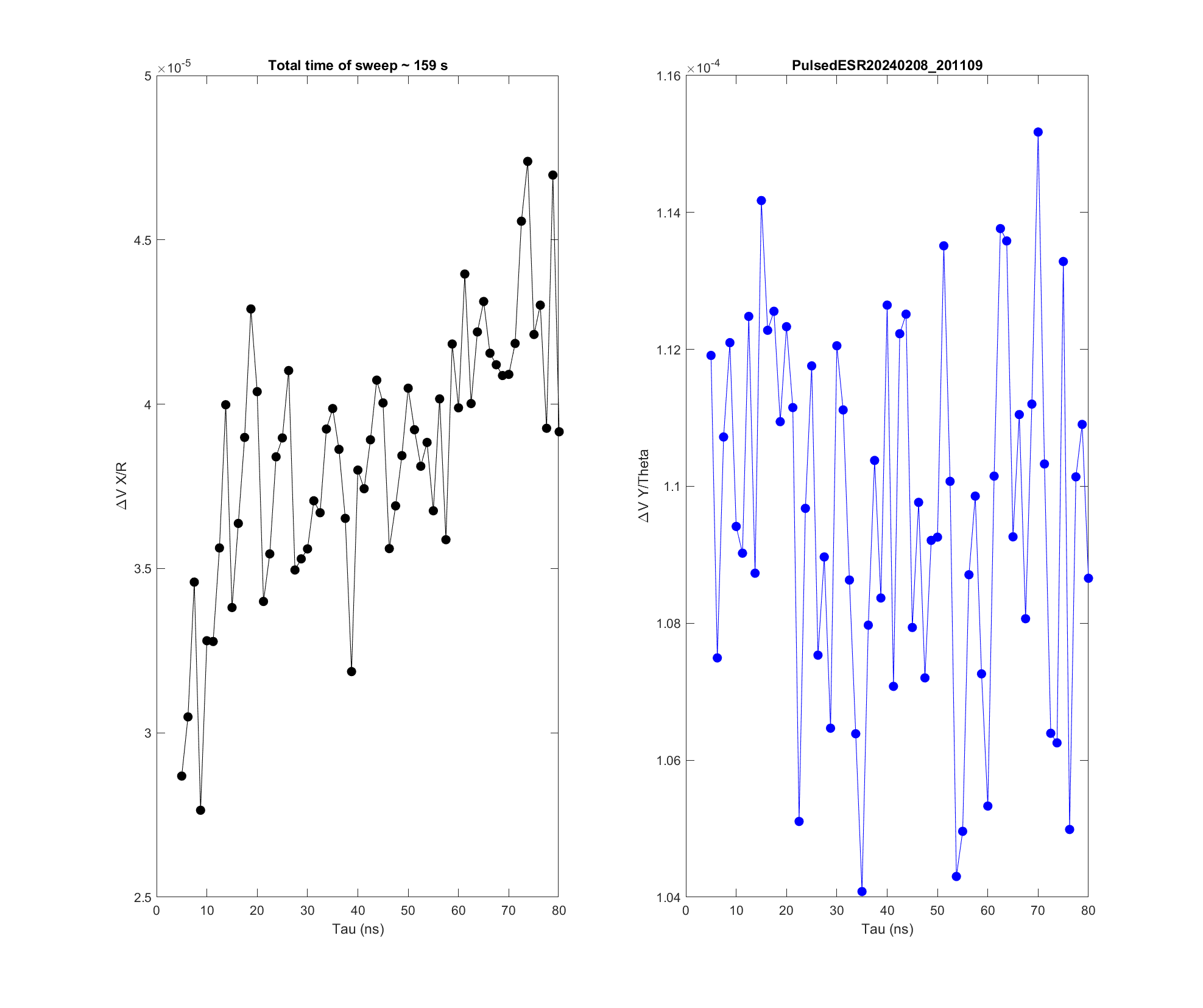

Supplement: Supplementary file 3 — Source Data [file 41467_2025_60409_MOESM3_ESM.zip › SupplementaryData1/Figure3/Fig3d/Ramsey/PulsedESR20240208_201109.png]

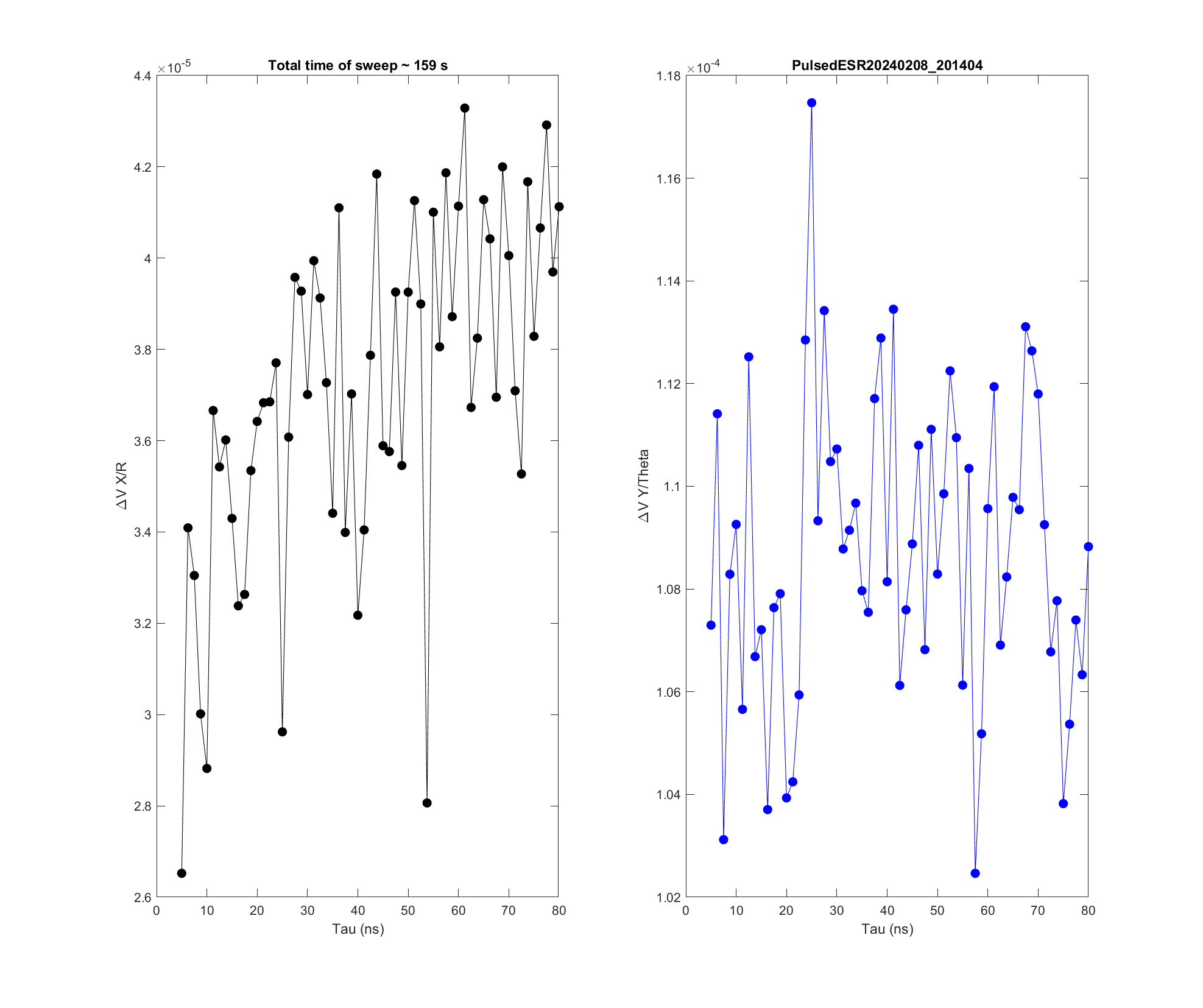

Supplement: Supplementary file 3 — Source Data [file 41467_2025_60409_MOESM3_ESM.zip › SupplementaryData1/Figure3/Fig3d/Ramsey/PulsedESR20240208_201404.png]

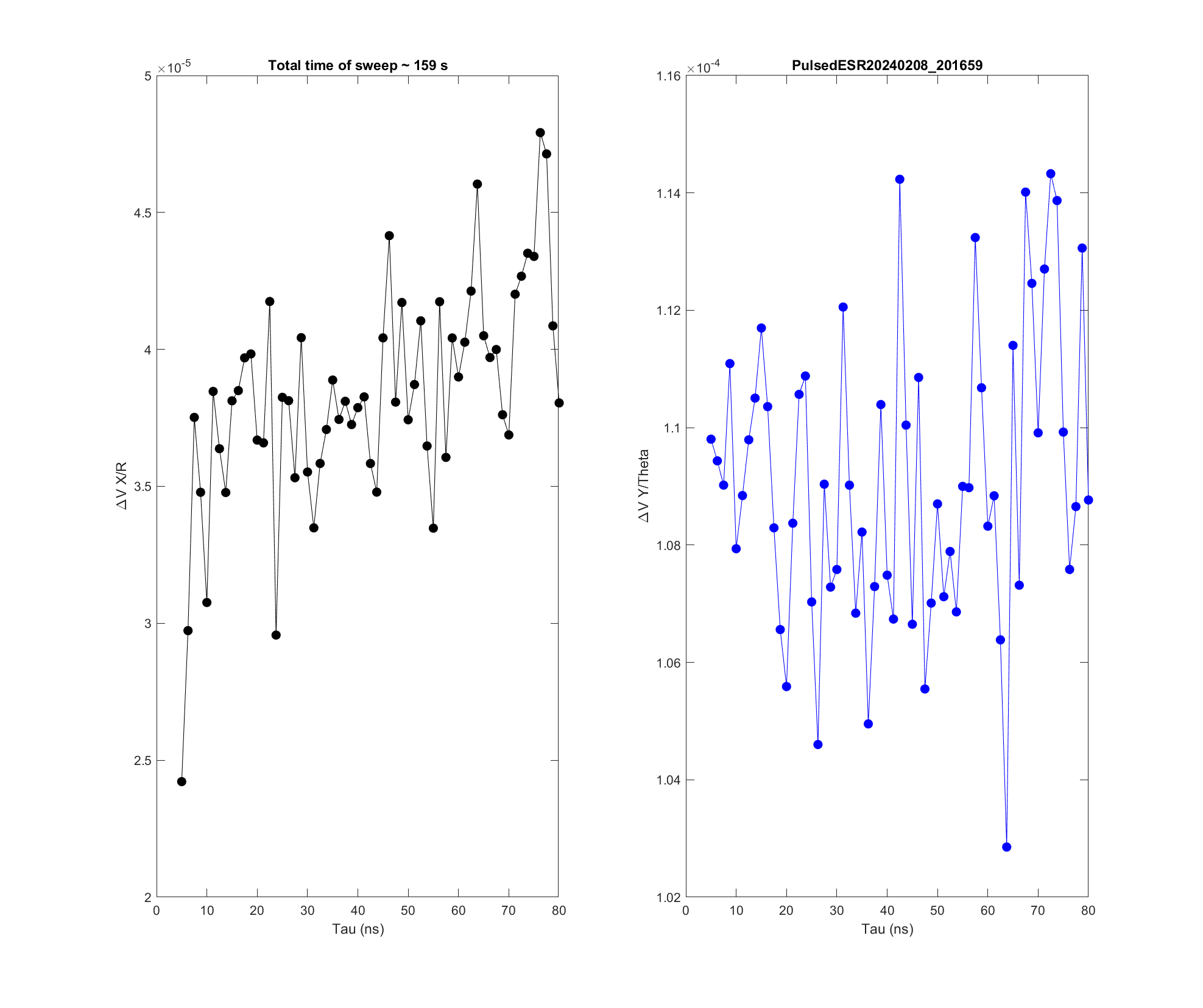

Supplement: Supplementary file 3 — Source Data [file 41467_2025_60409_MOESM3_ESM.zip › SupplementaryData1/Figure3/Fig3d/Ramsey/PulsedESR20240208_201659.png]

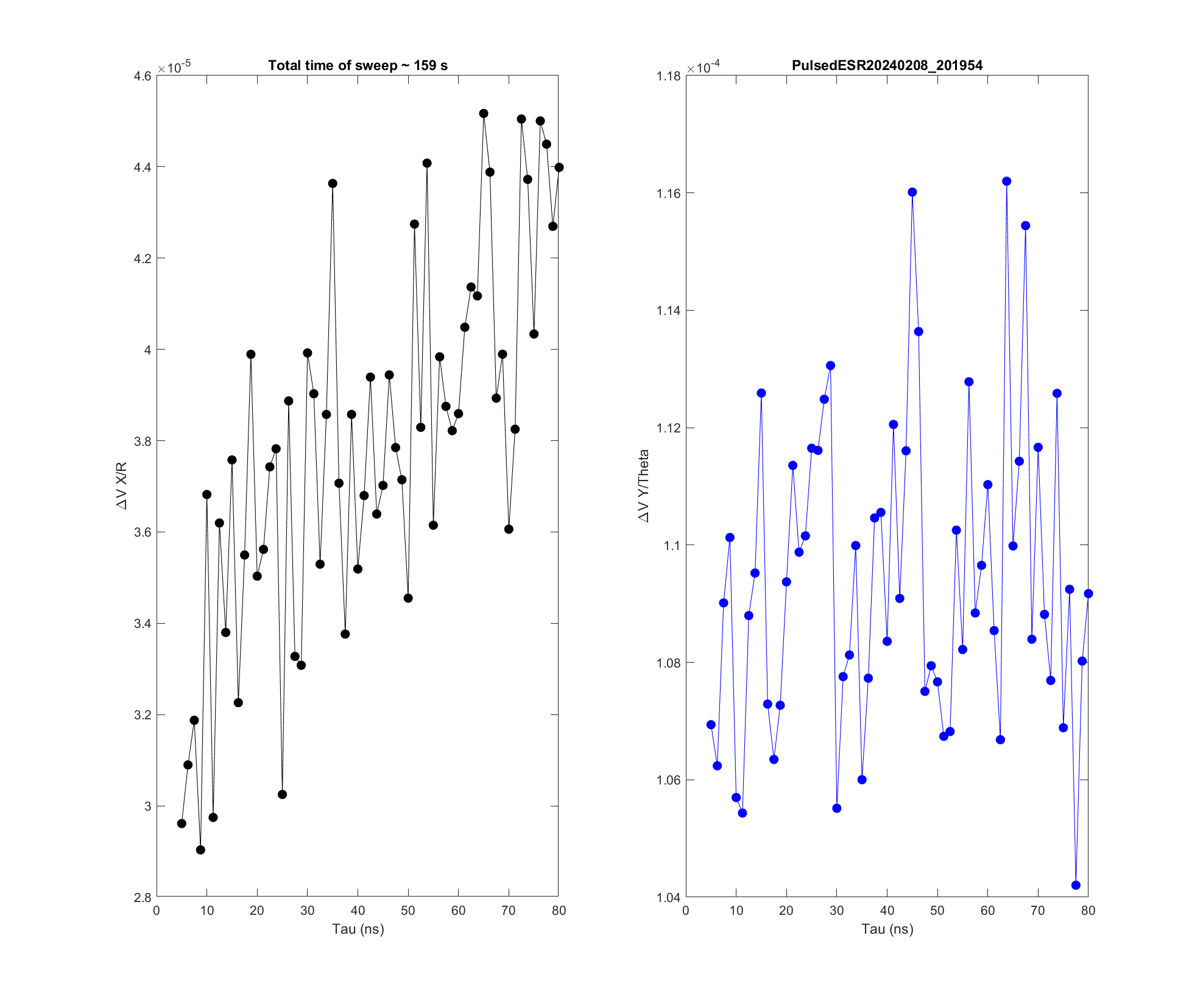

Supplement: Supplementary file 3 — Source Data [file 41467_2025_60409_MOESM3_ESM.zip › SupplementaryData1/Figure3/Fig3d/Ramsey/PulsedESR20240208_201954.png]

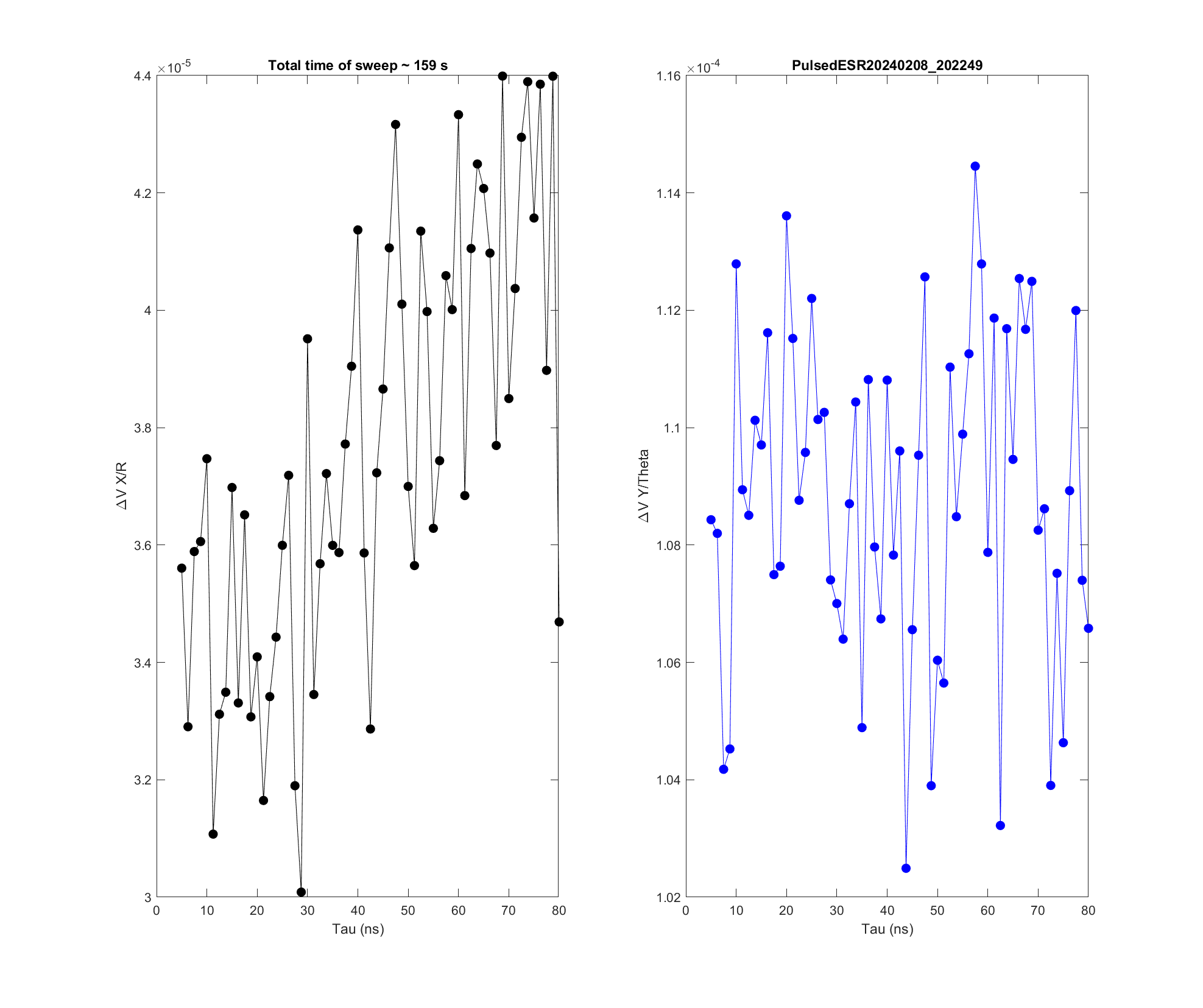

Supplement: Supplementary file 3 — Source Data [file 41467_2025_60409_MOESM3_ESM.zip › SupplementaryData1/Figure3/Fig3d/Ramsey/PulsedESR20240208_202249.png]

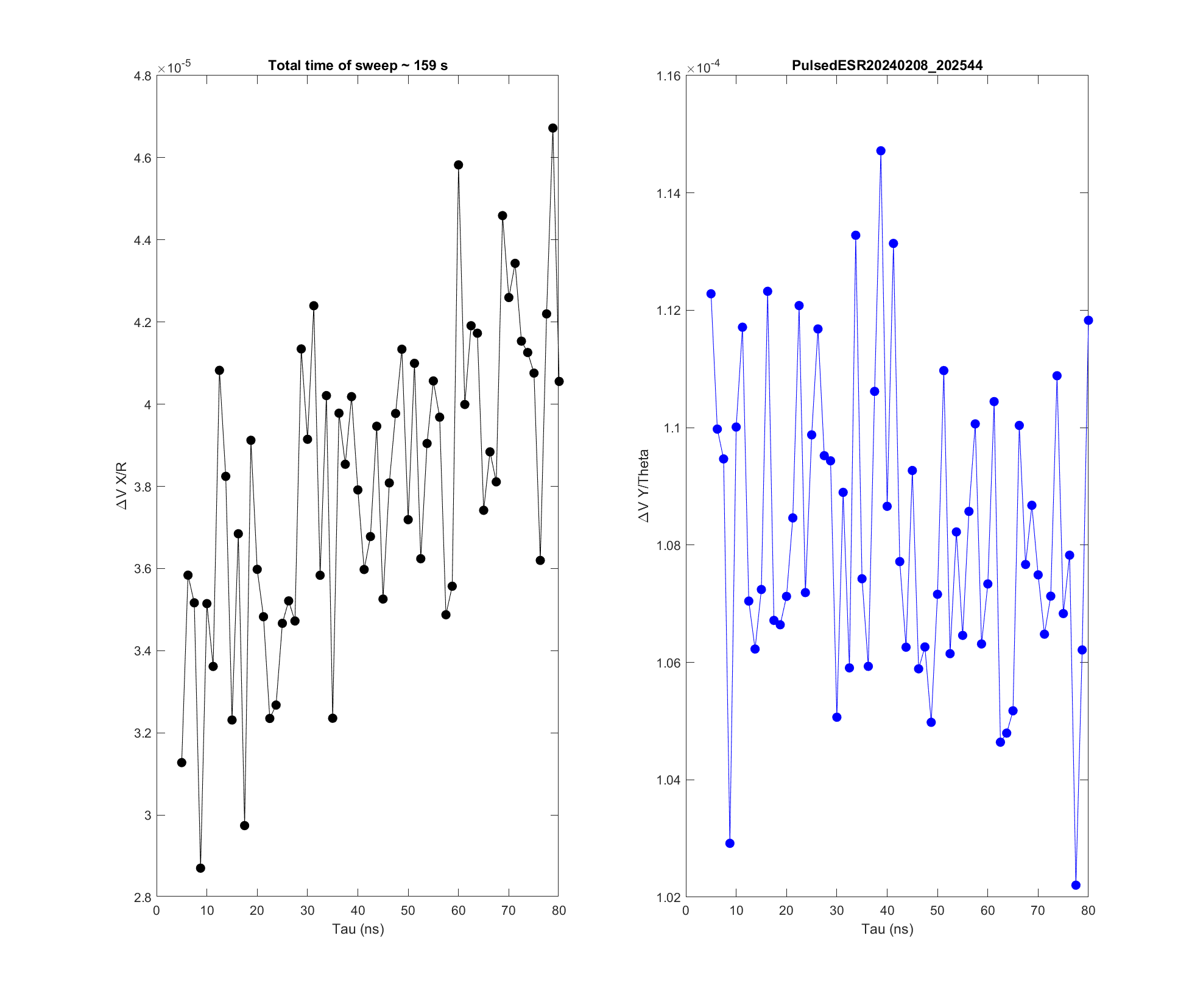

Supplement: Supplementary file 3 — Source Data [file 41467_2025_60409_MOESM3_ESM.zip › SupplementaryData1/Figure3/Fig3d/Ramsey/PulsedESR20240208_202544.png]

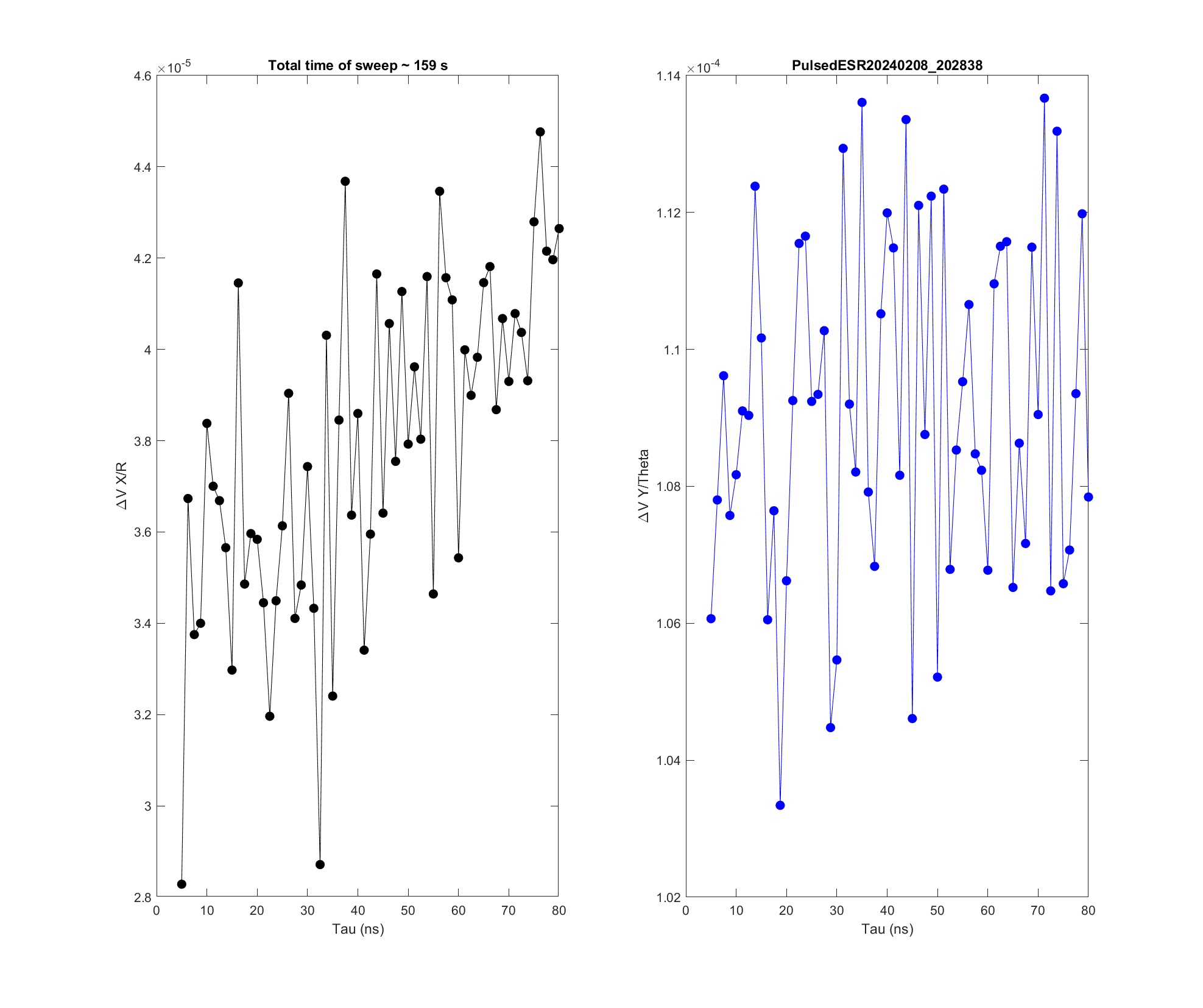

Supplement: Supplementary file 3 — Source Data [file 41467_2025_60409_MOESM3_ESM.zip › SupplementaryData1/Figure3/Fig3d/Ramsey/PulsedESR20240208_202838.png]

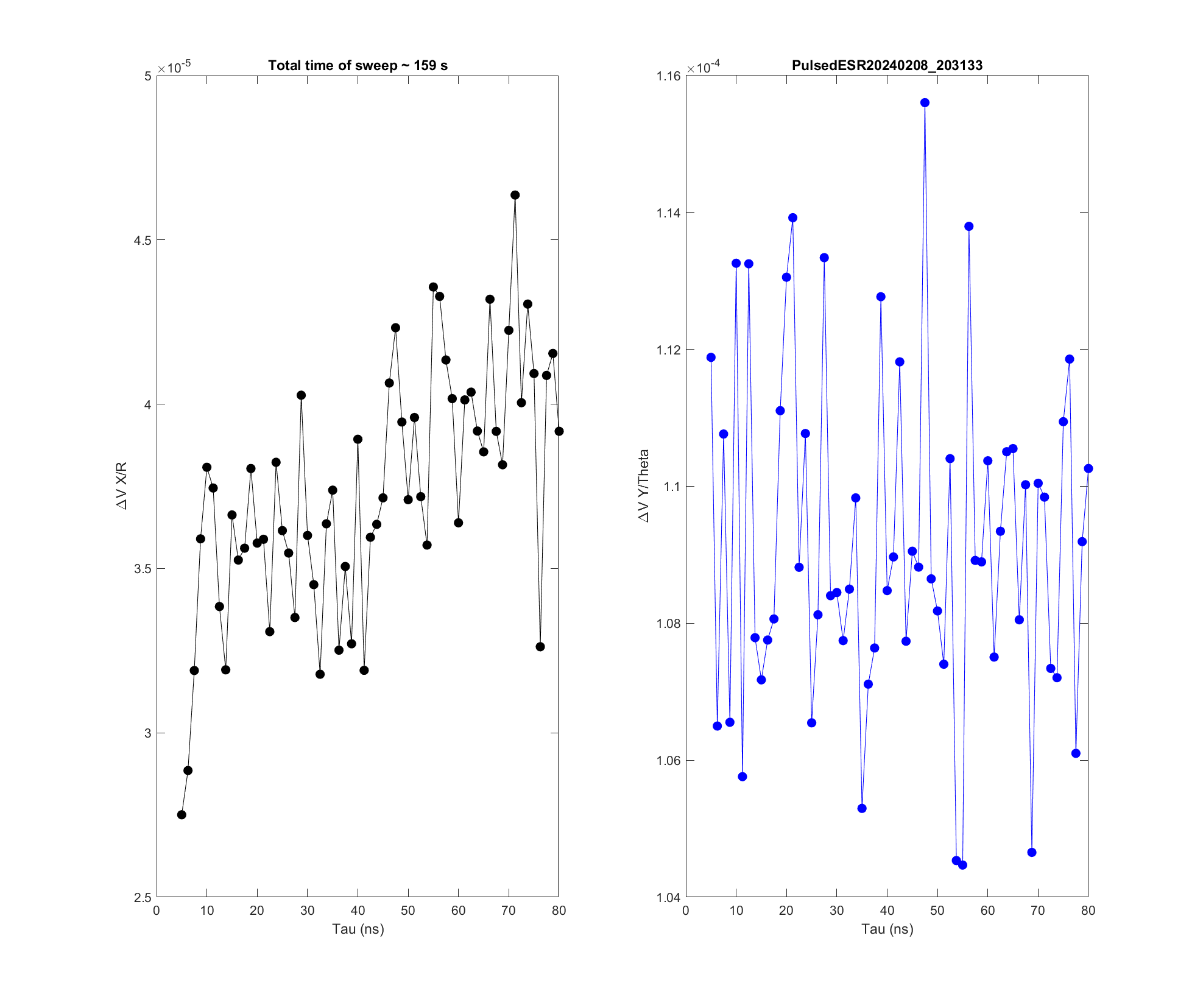

Supplement: Supplementary file 3 — Source Data [file 41467_2025_60409_MOESM3_ESM.zip › SupplementaryData1/Figure3/Fig3d/Ramsey/PulsedESR20240208_203133.png]

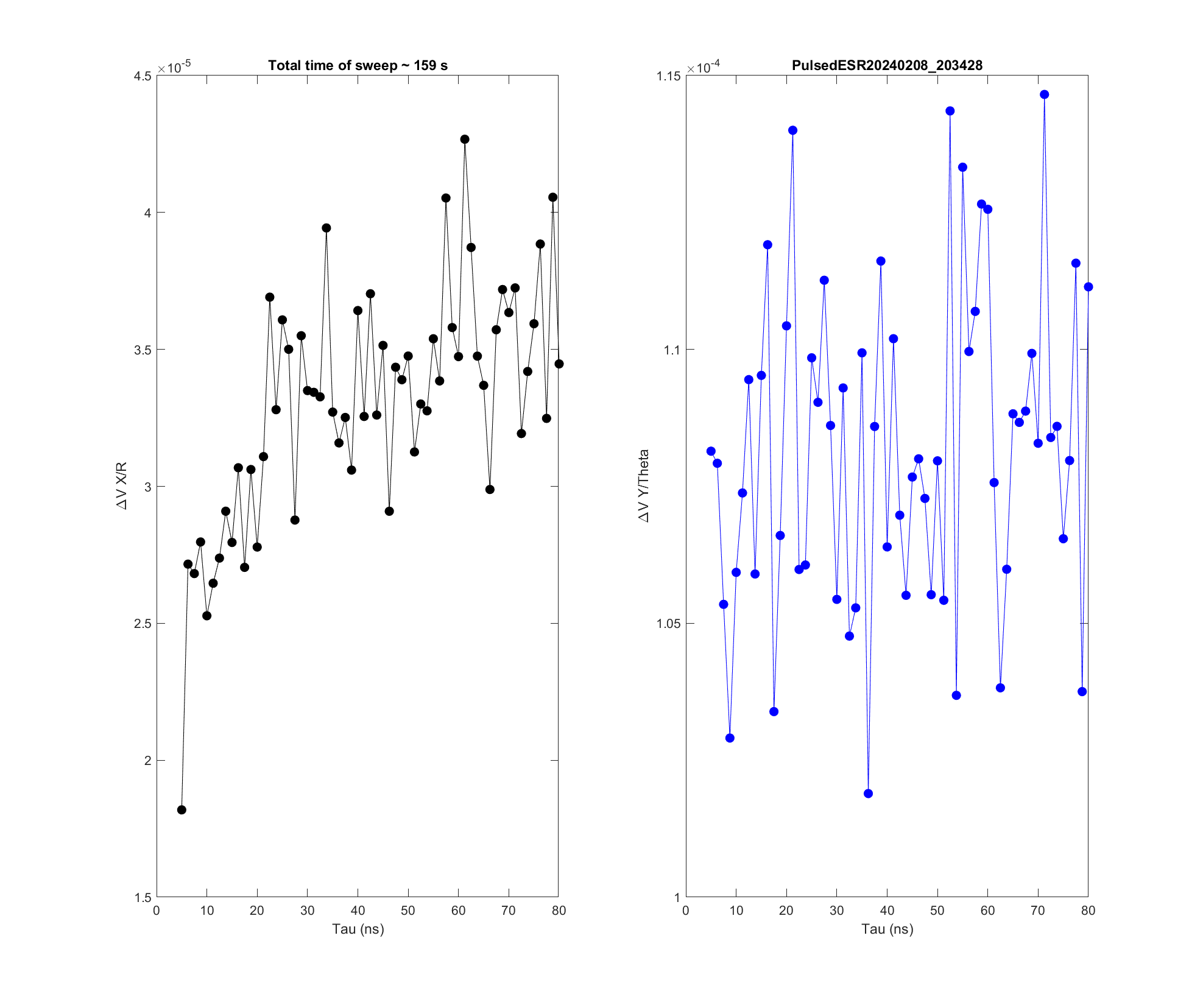

Supplement: Supplementary file 3 — Source Data [file 41467_2025_60409_MOESM3_ESM.zip › SupplementaryData1/Figure3/Fig3d/Ramsey/PulsedESR20240208_203428.png]
