# Supplementary figures and images for: Quantum spin-engineering in on-surface molecular ferrimagnets (part 1 of 2)
Source: Nat Commun. 2025 Jun 5;16:5208. doi: 10.1038/s41467-025-60409-w (PMC12137798; doi:10.1038/s41467-025-60409-w)

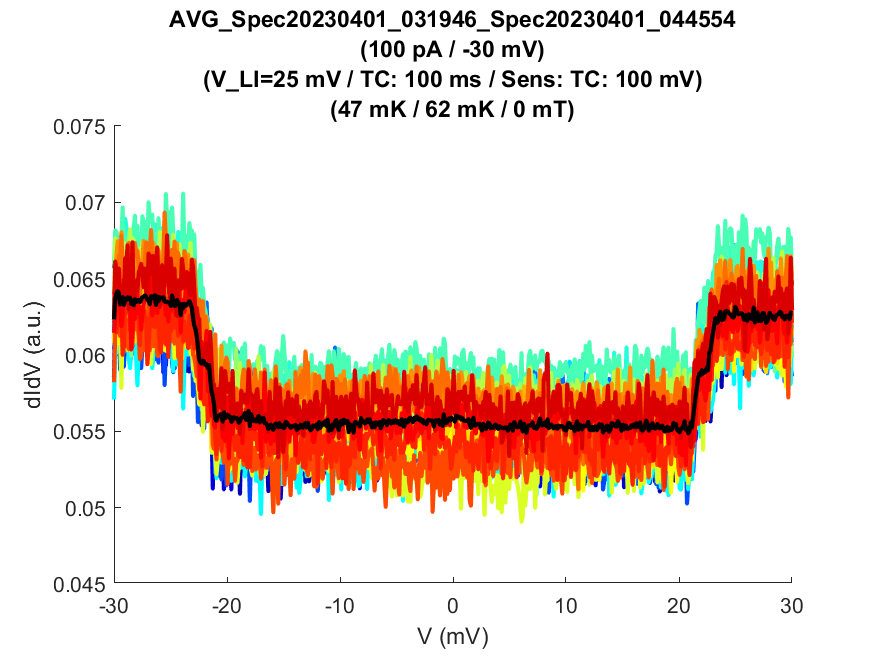

Supplement: Supplementary file 3 — Source Data [file 41467_2025_60409_MOESM3_ESM.zip › SupplementaryData1/Figure1/Fig1c_dIdVFit/AVG_Spec20230401_031946_Spec20230401_044554.png]

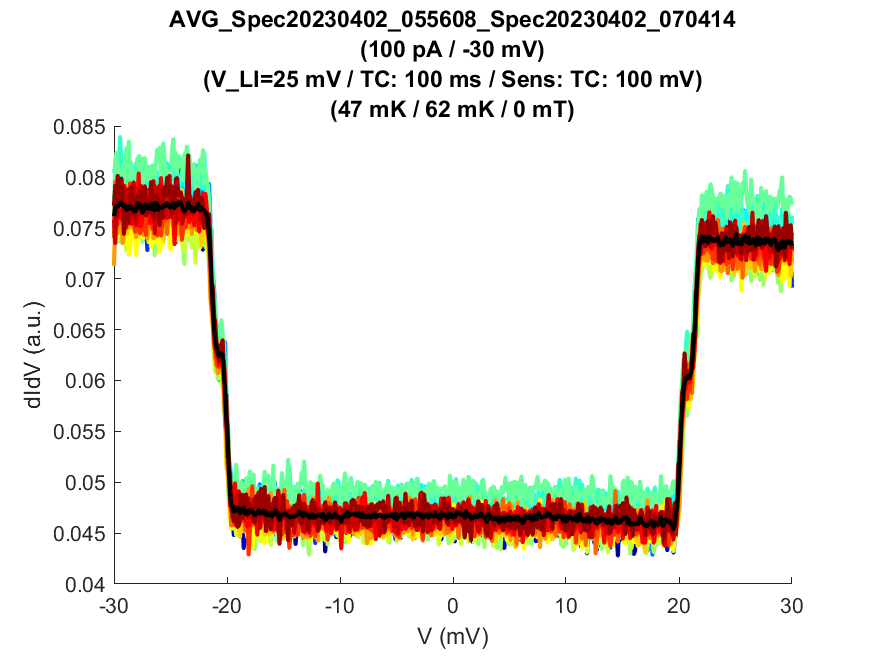

Supplement: Supplementary file 3 — Source Data [file 41467_2025_60409_MOESM3_ESM.zip › SupplementaryData1/Figure1/Fig1c_dIdVFit/AVG_Spec20230402_055608_Spec20230402_070414.png]

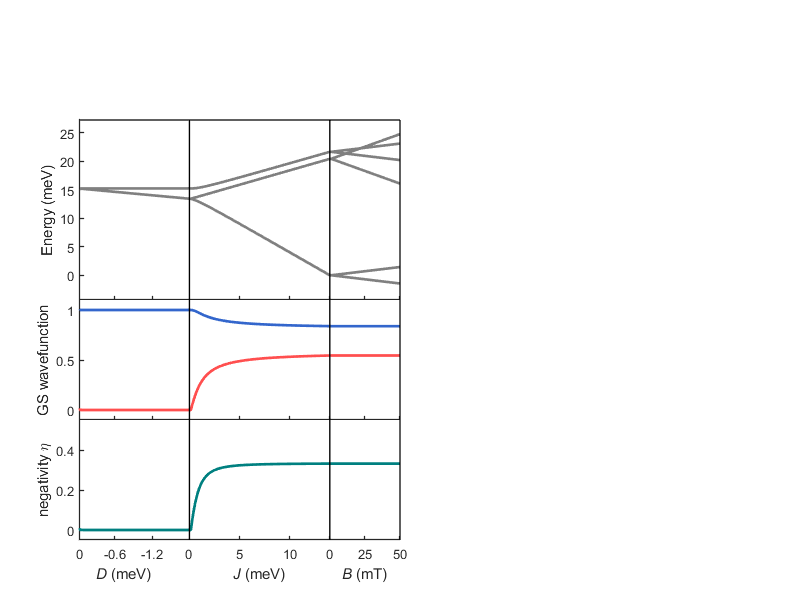

Supplement: Supplementary file 3 — Source Data [file 41467_2025_60409_MOESM3_ESM.zip › SupplementaryData1/Figure2/Fig2abef/EnergyDiagram.tif]

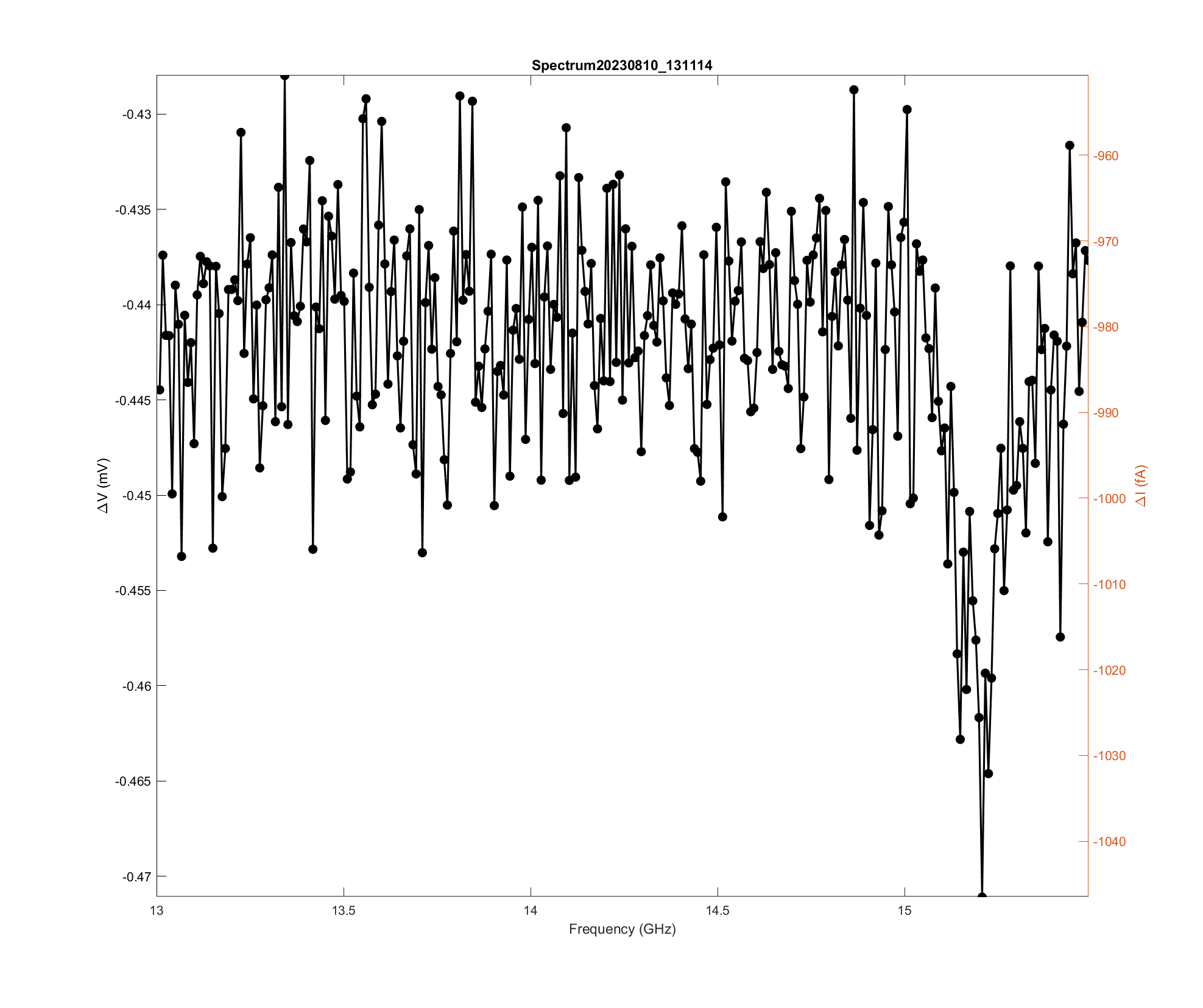

Supplement: Supplementary file 3 — Source Data [file 41467_2025_60409_MOESM3_ESM.zip › SupplementaryData1/Figure2/Fig2c/Spectrum20230810_131114.png]

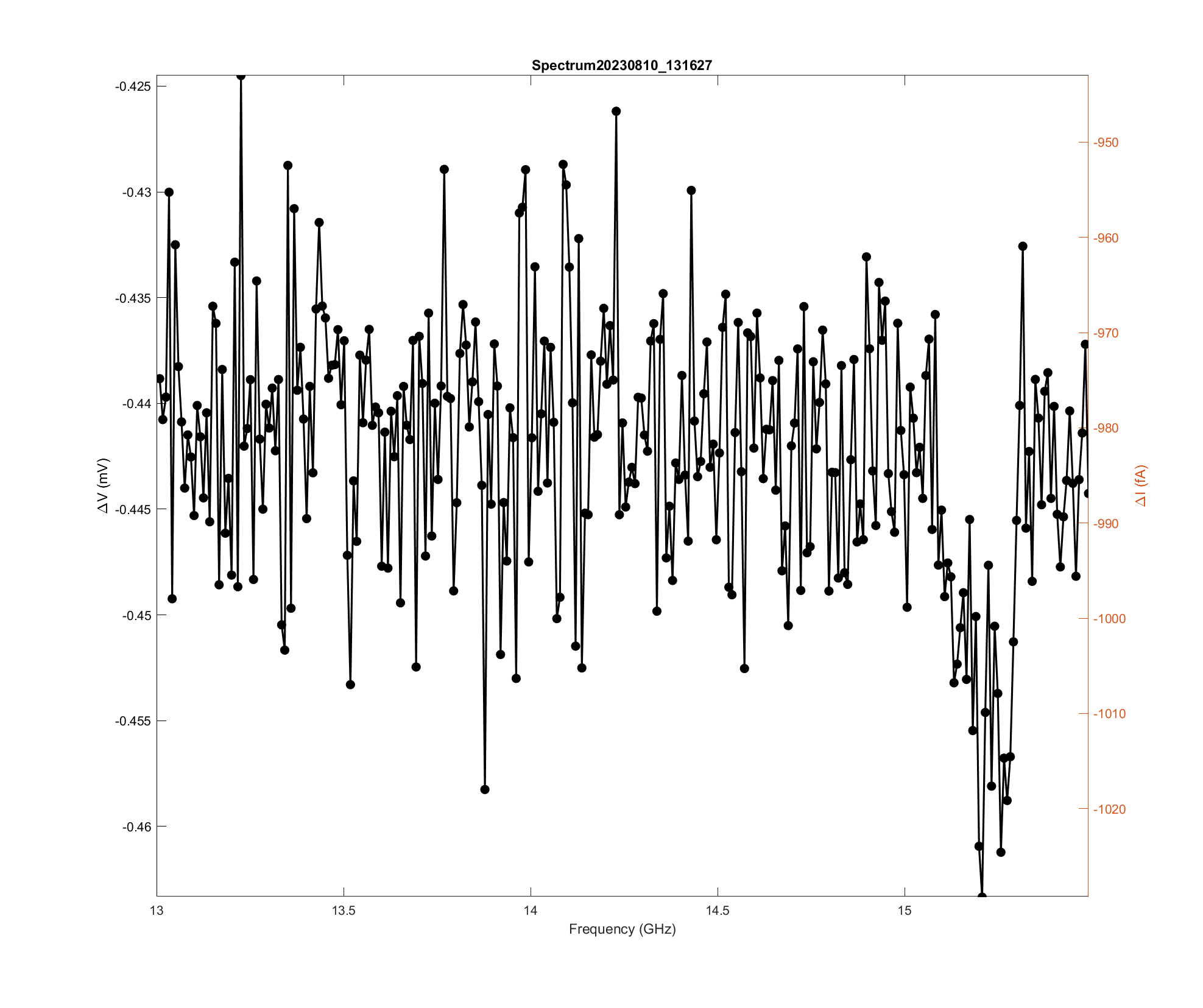

Supplement: Supplementary file 3 — Source Data [file 41467_2025_60409_MOESM3_ESM.zip › SupplementaryData1/Figure2/Fig2c/Spectrum20230810_131627.png]

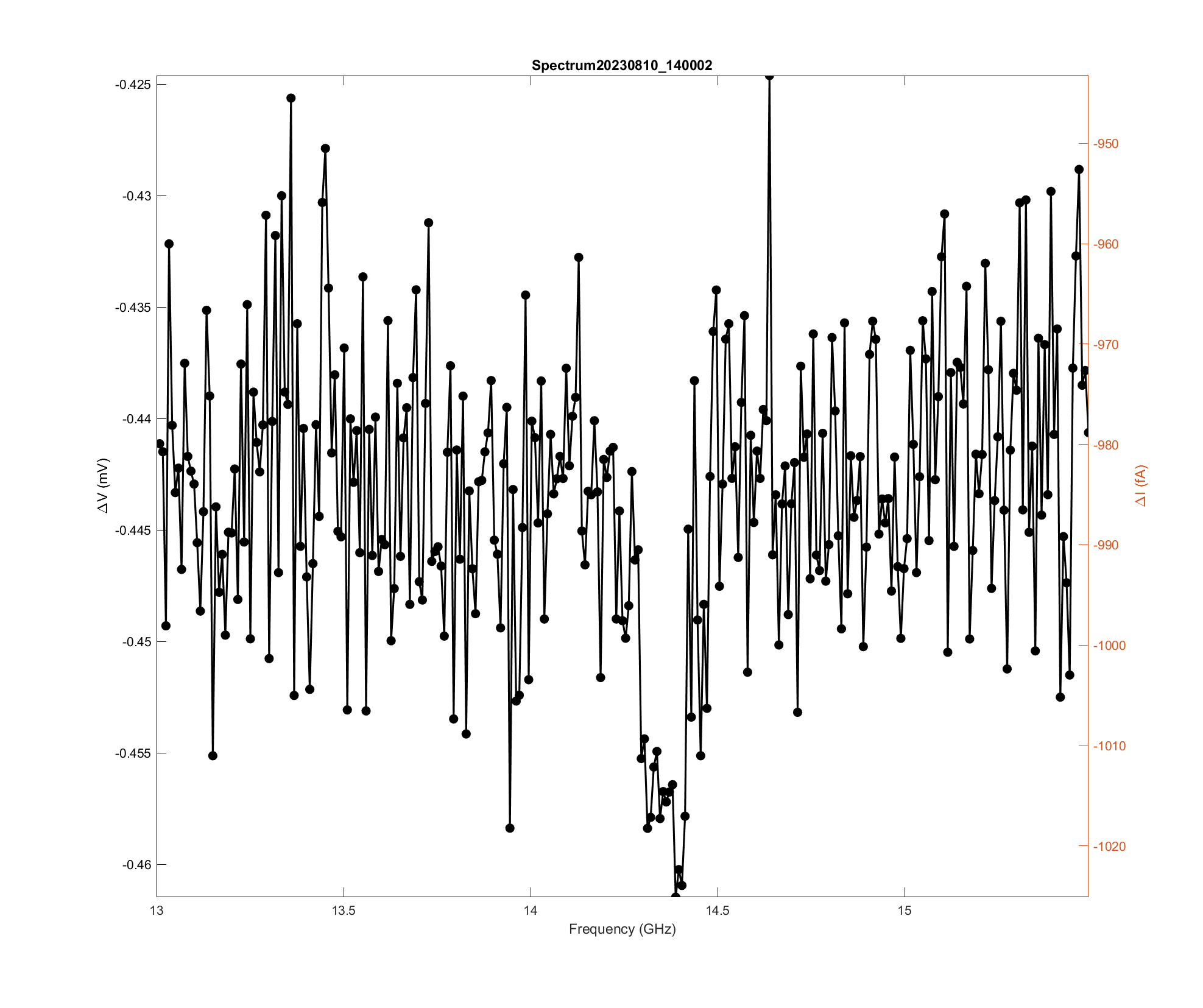

Supplement: Supplementary file 3 — Source Data [file 41467_2025_60409_MOESM3_ESM.zip › SupplementaryData1/Figure2/Fig2c/Spectrum20230810_140002.png]

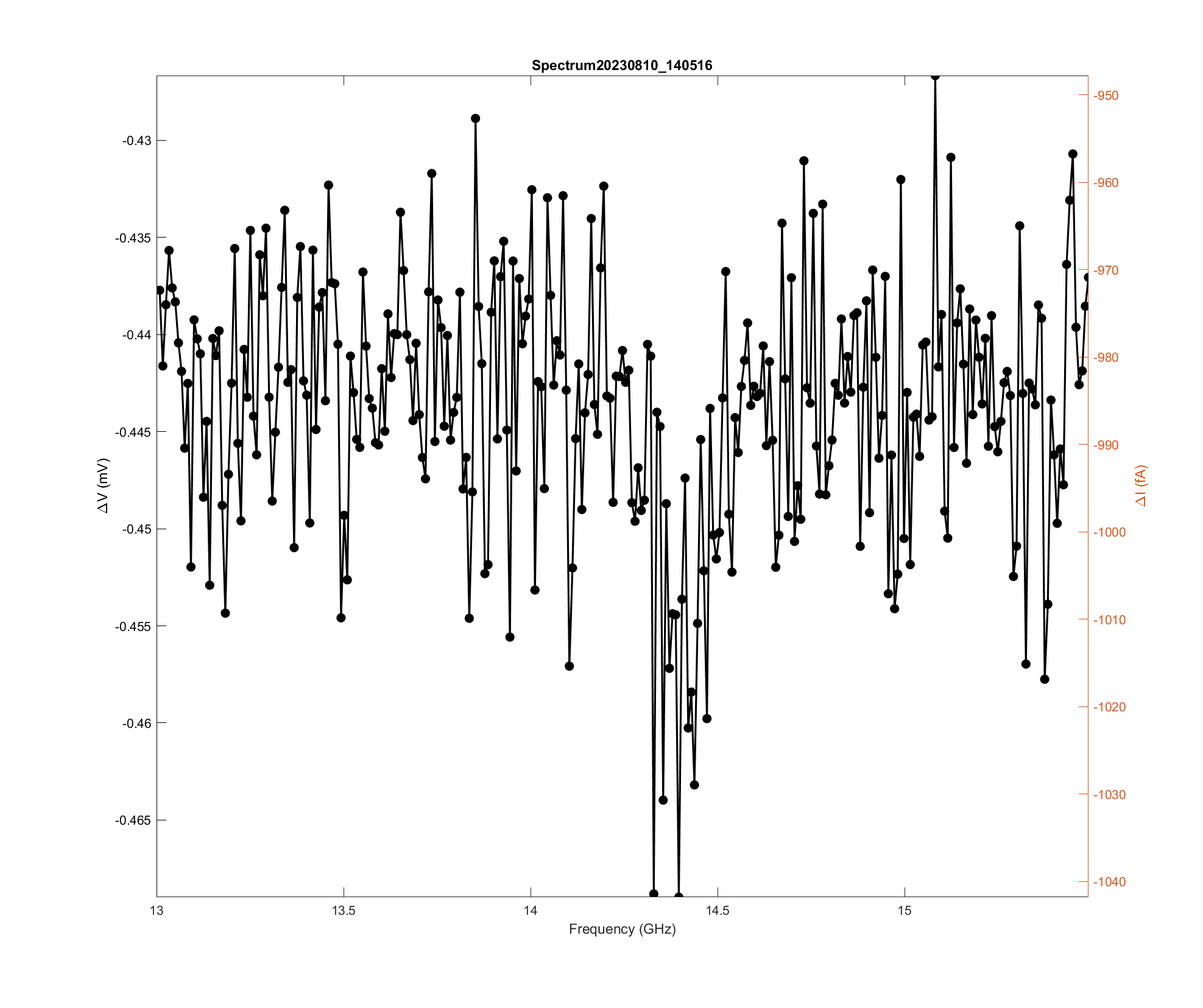

Supplement: Supplementary file 3 — Source Data [file 41467_2025_60409_MOESM3_ESM.zip › SupplementaryData1/Figure2/Fig2c/Spectrum20230810_140516.png]

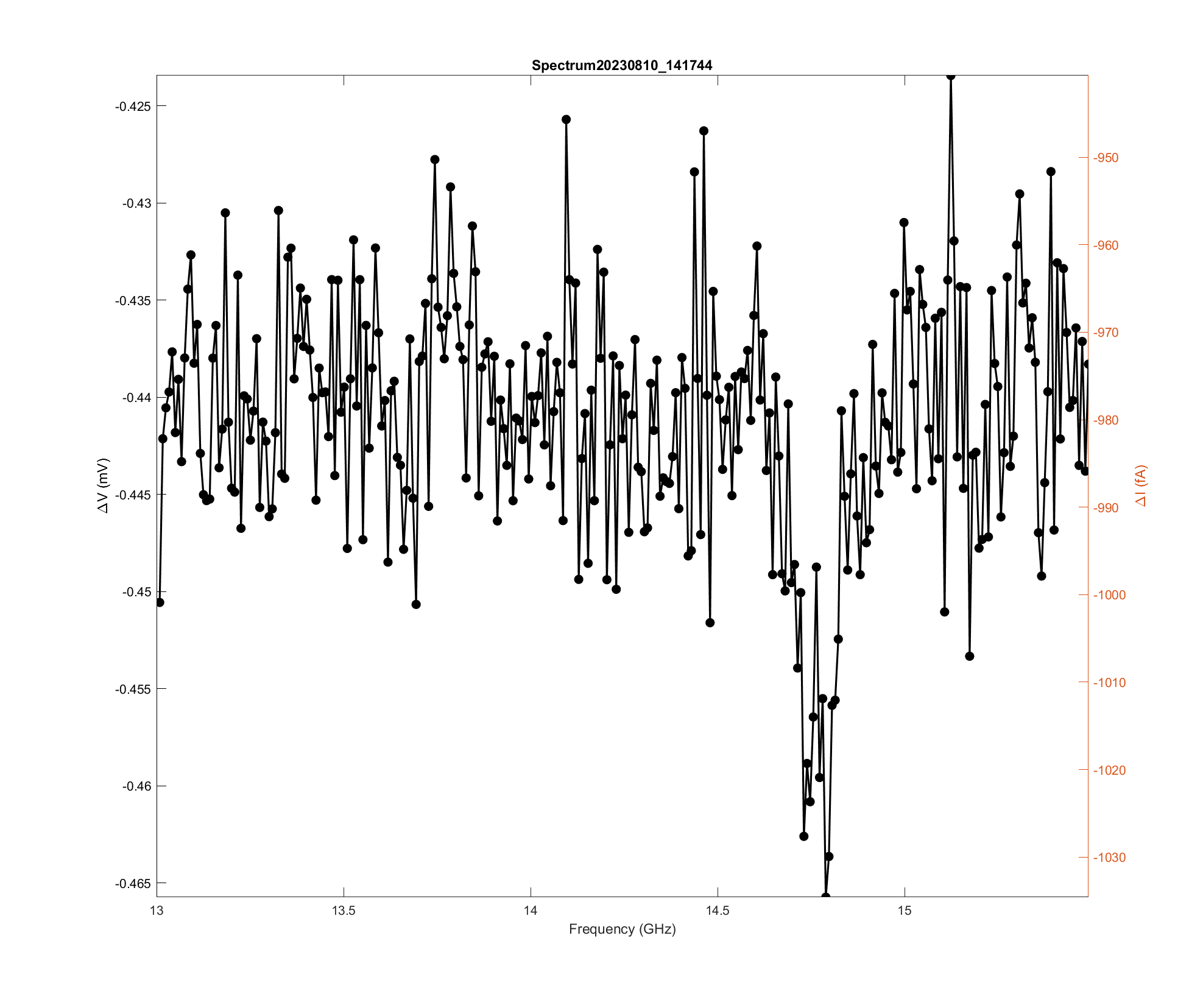

Supplement: Supplementary file 3 — Source Data [file 41467_2025_60409_MOESM3_ESM.zip › SupplementaryData1/Figure2/Fig2c/Spectrum20230810_141744.png]

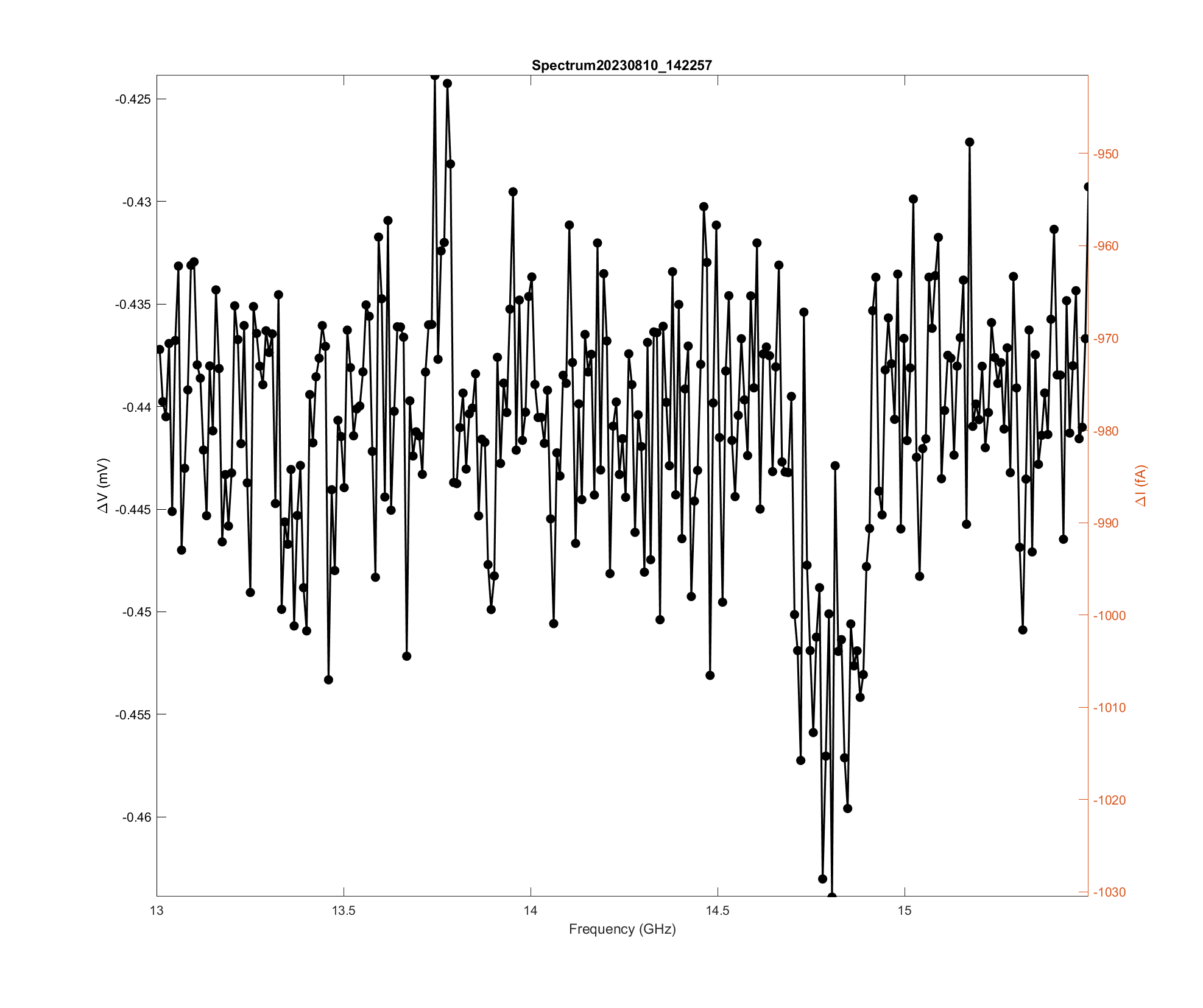

Supplement: Supplementary file 3 — Source Data [file 41467_2025_60409_MOESM3_ESM.zip › SupplementaryData1/Figure2/Fig2c/Spectrum20230810_142257.png]

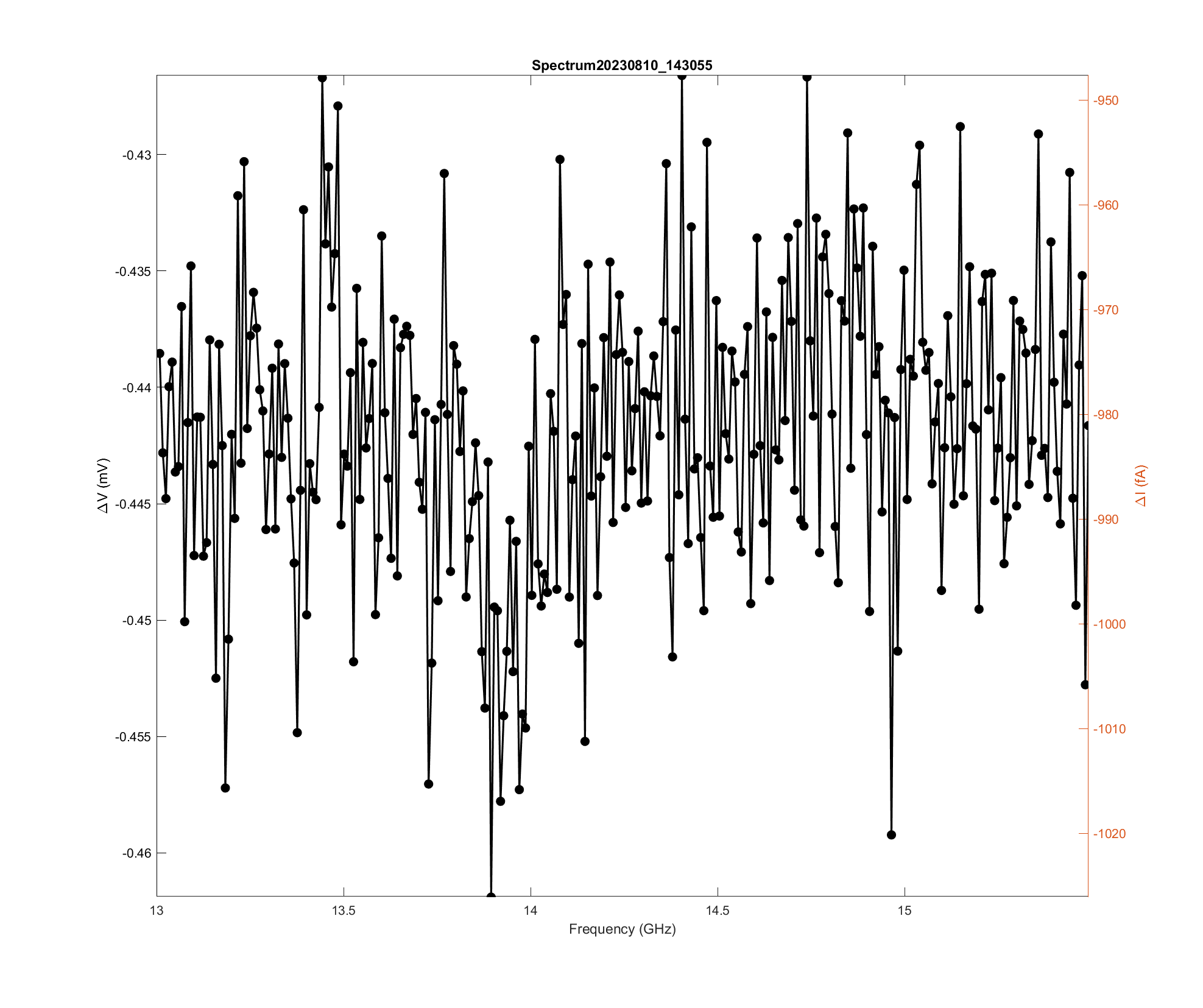

Supplement: Supplementary file 3 — Source Data [file 41467_2025_60409_MOESM3_ESM.zip › SupplementaryData1/Figure2/Fig2c/Spectrum20230810_143055.png]

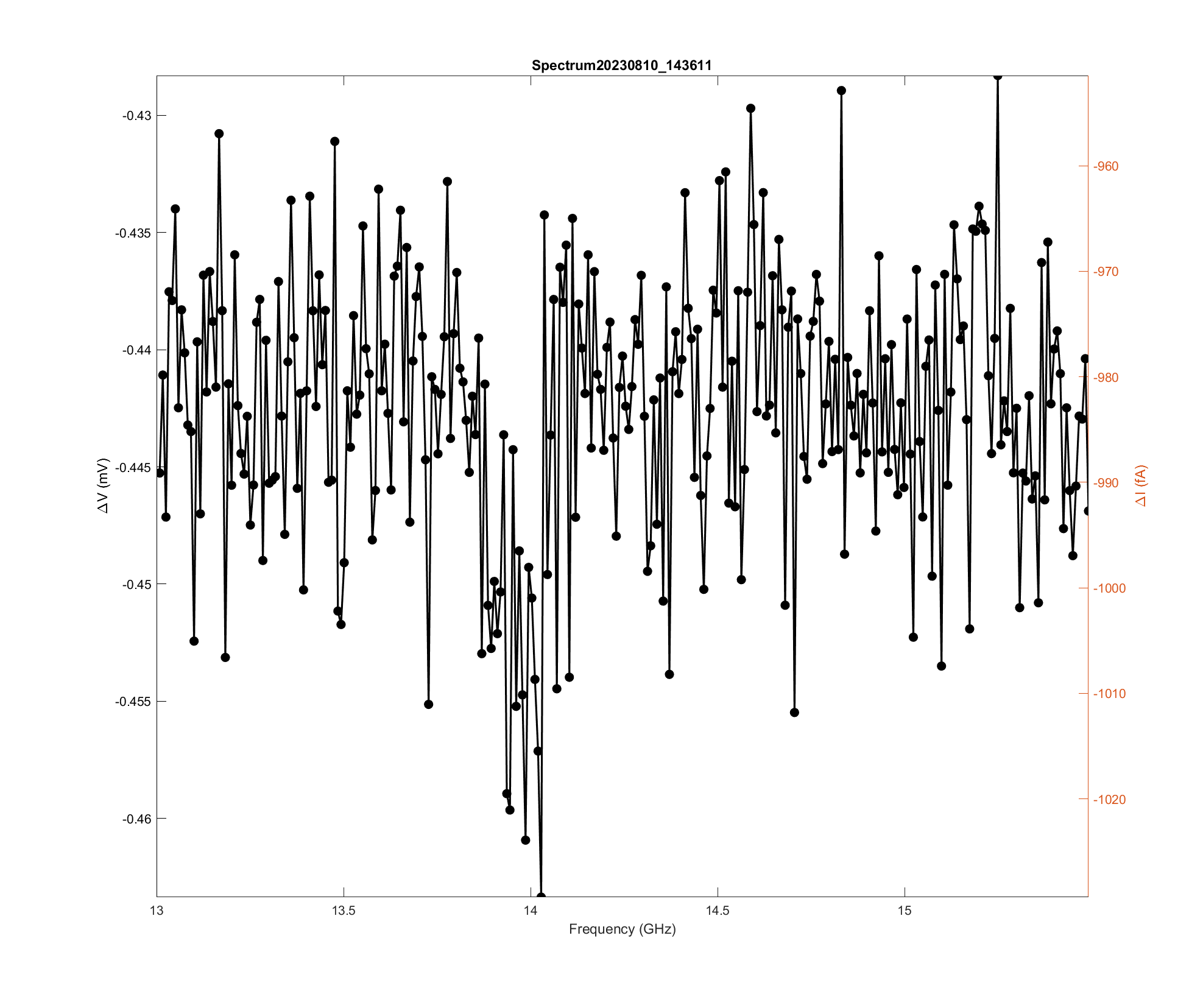

Supplement: Supplementary file 3 — Source Data [file 41467_2025_60409_MOESM3_ESM.zip › SupplementaryData1/Figure2/Fig2c/Spectrum20230810_143611.png]

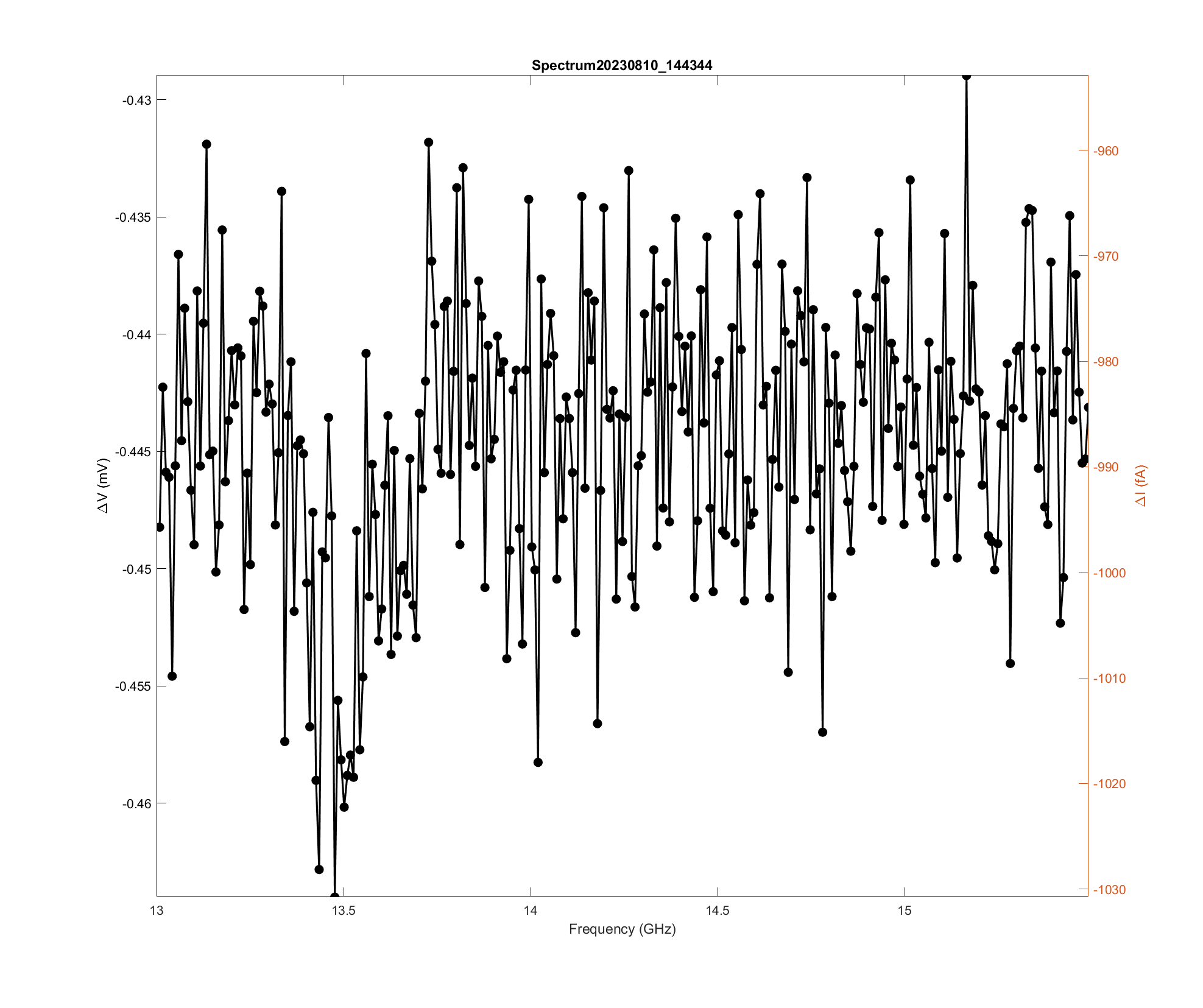

Supplement: Supplementary file 3 — Source Data [file 41467_2025_60409_MOESM3_ESM.zip › SupplementaryData1/Figure2/Fig2c/Spectrum20230810_144344.png]

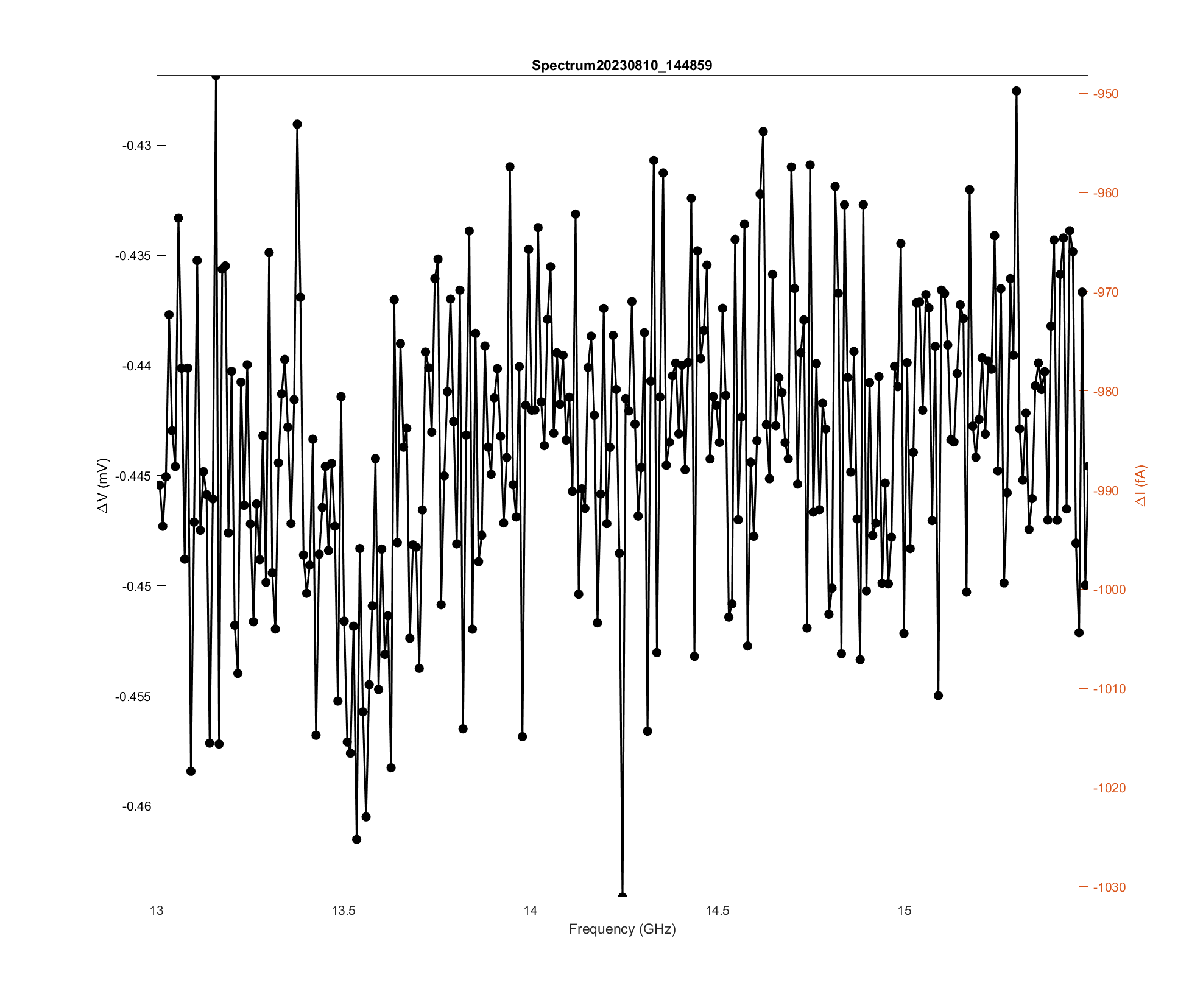

Supplement: Supplementary file 3 — Source Data [file 41467_2025_60409_MOESM3_ESM.zip › SupplementaryData1/Figure2/Fig2c/Spectrum20230810_144859.png]

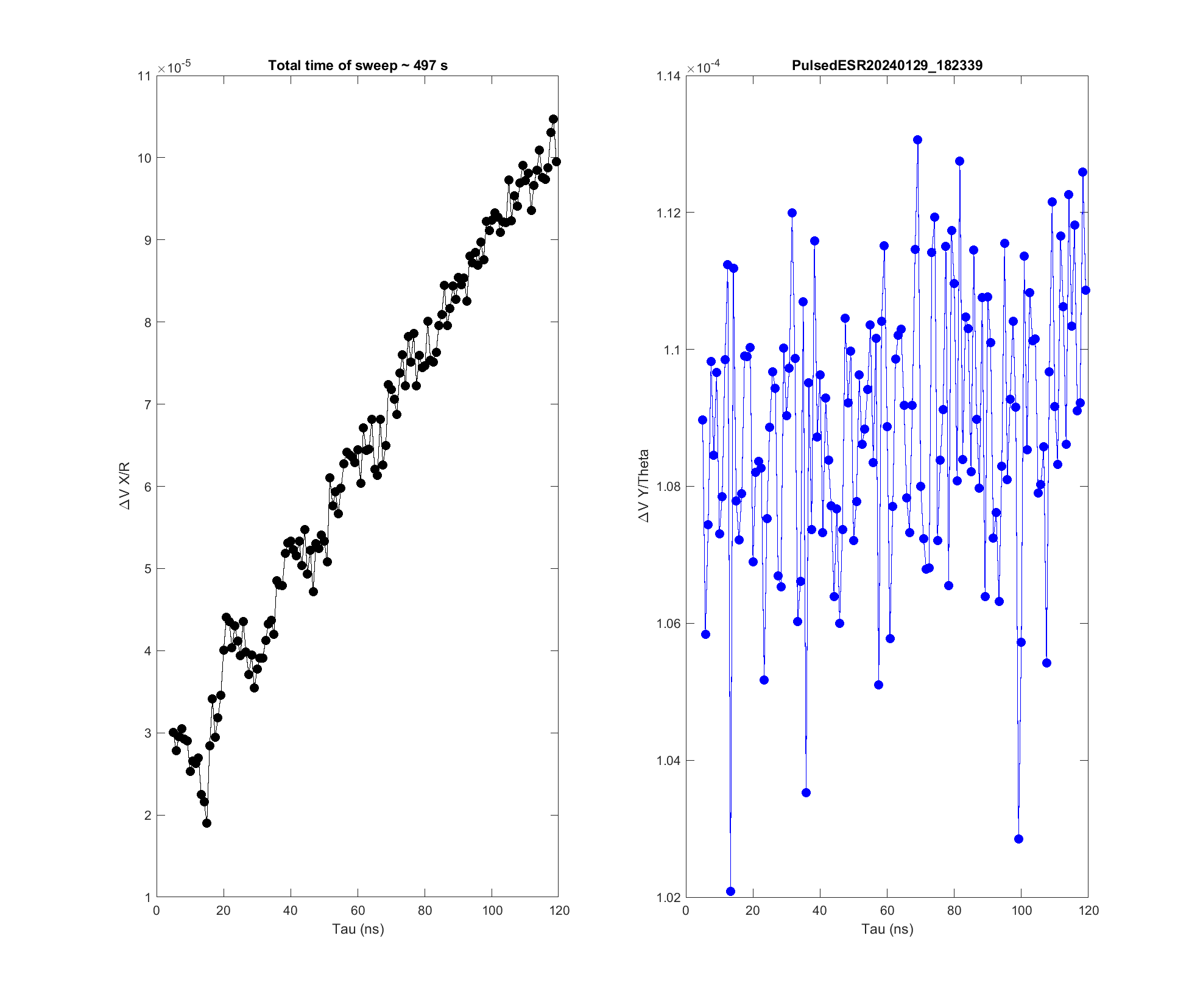

Supplement: Supplementary file 3 — Source Data [file 41467_2025_60409_MOESM3_ESM.zip › SupplementaryData1/Figure3/Fig3ab/PulsedESR20240129_182339.png]

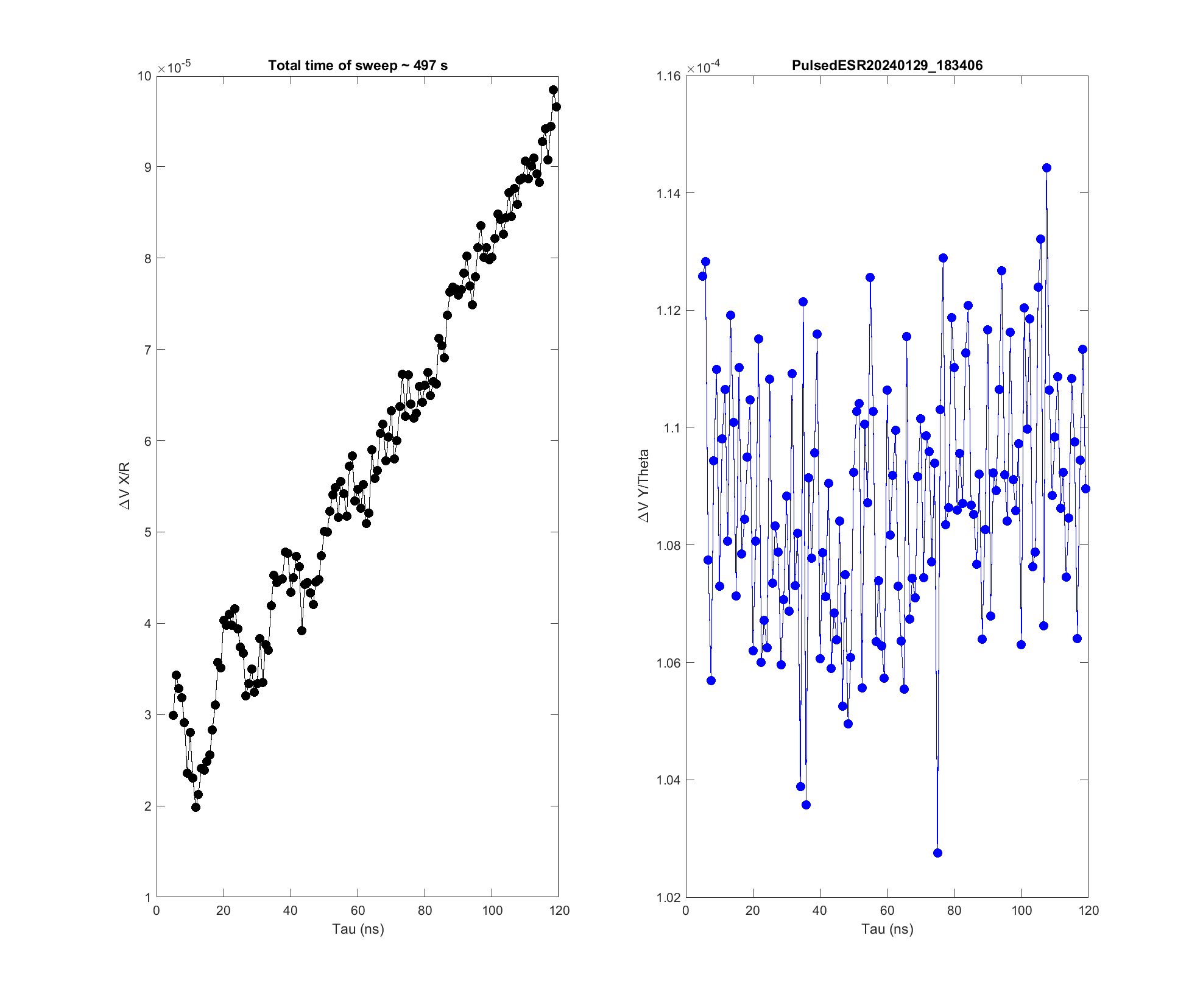

Supplement: Supplementary file 3 — Source Data [file 41467_2025_60409_MOESM3_ESM.zip › SupplementaryData1/Figure3/Fig3ab/PulsedESR20240129_183406.png]

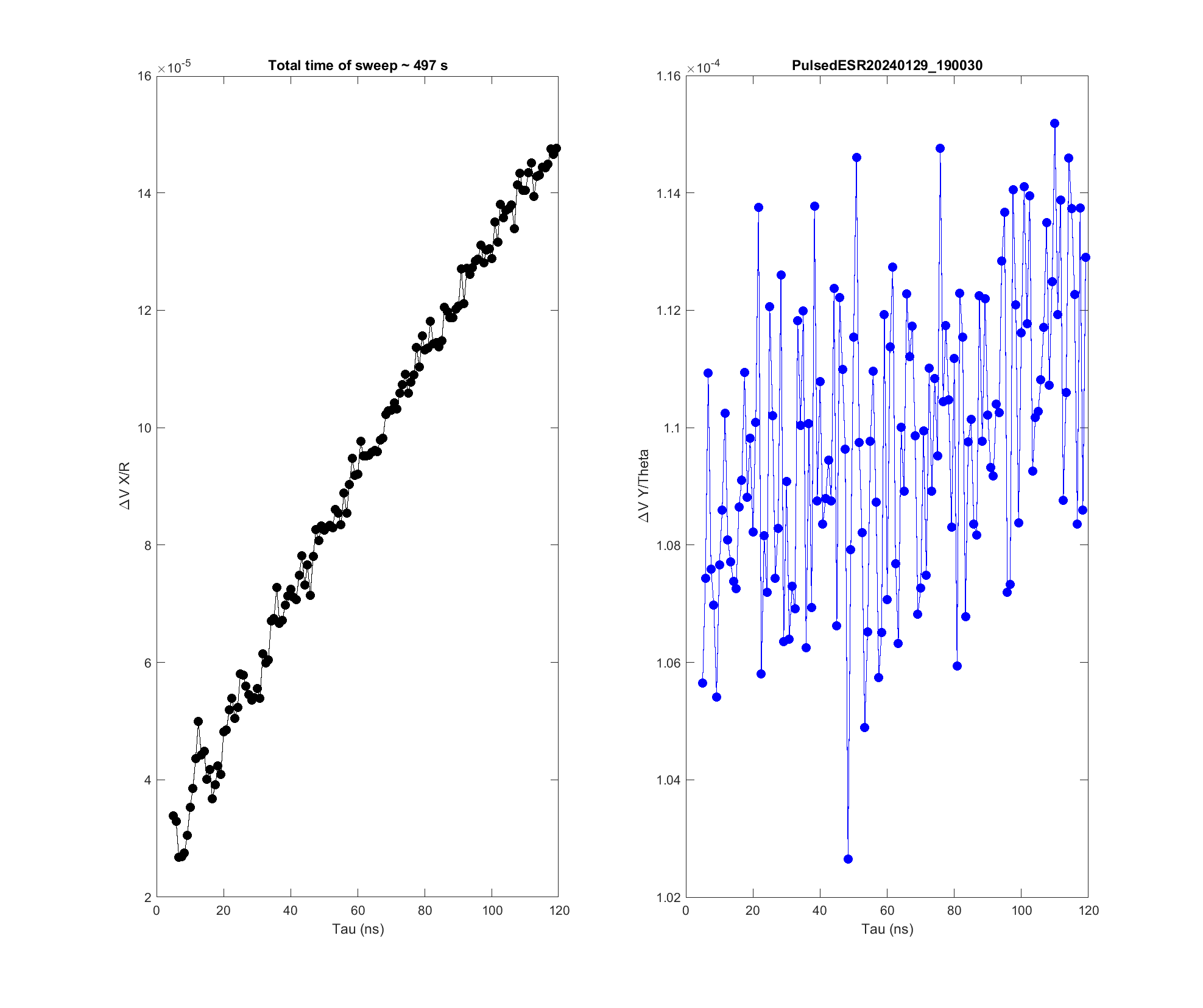

Supplement: Supplementary file 3 — Source Data [file 41467_2025_60409_MOESM3_ESM.zip › SupplementaryData1/Figure3/Fig3ab/PulsedESR20240129_190030.png]

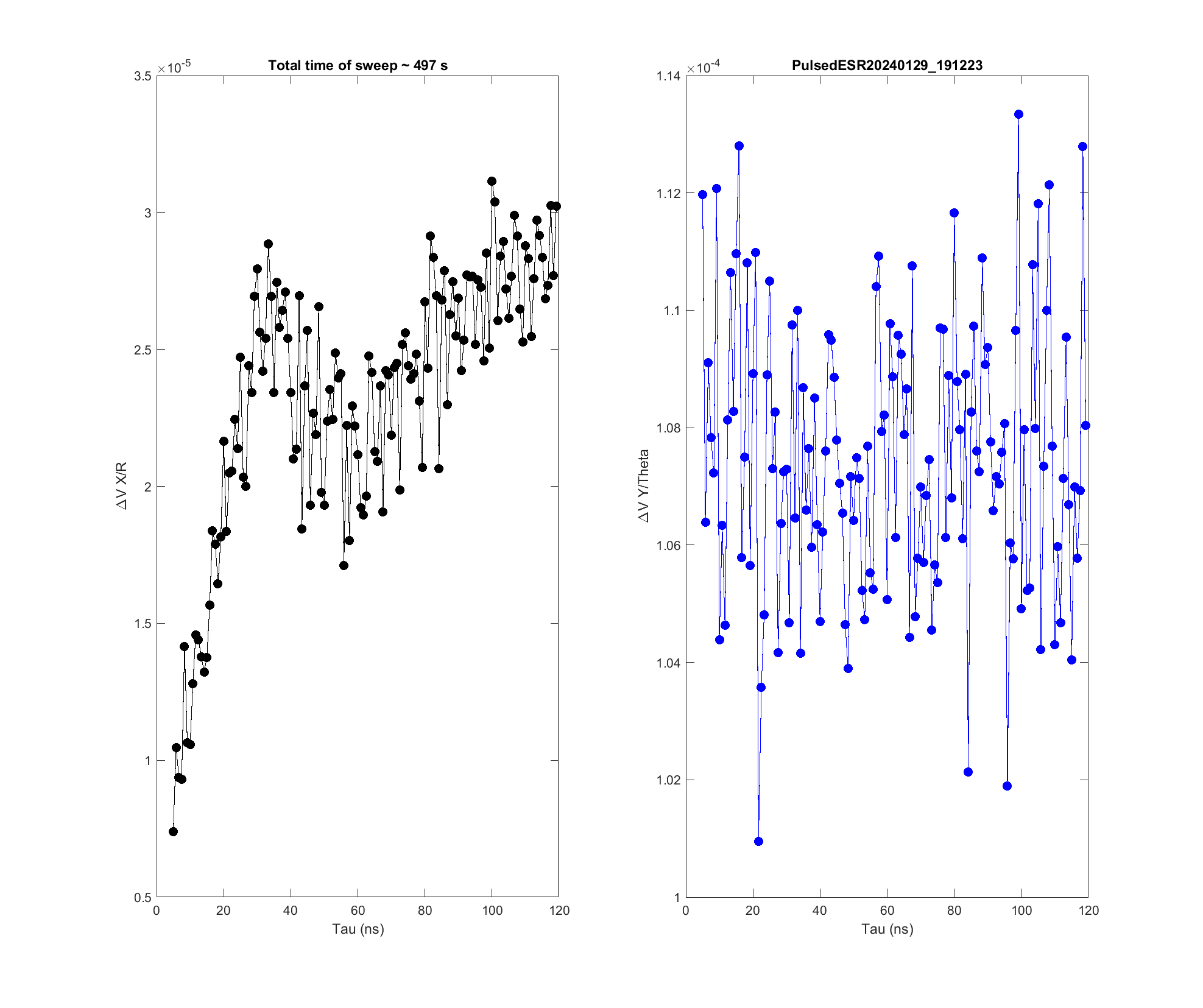

Supplement: Supplementary file 3 — Source Data [file 41467_2025_60409_MOESM3_ESM.zip › SupplementaryData1/Figure3/Fig3ab/PulsedESR20240129_191223.png]

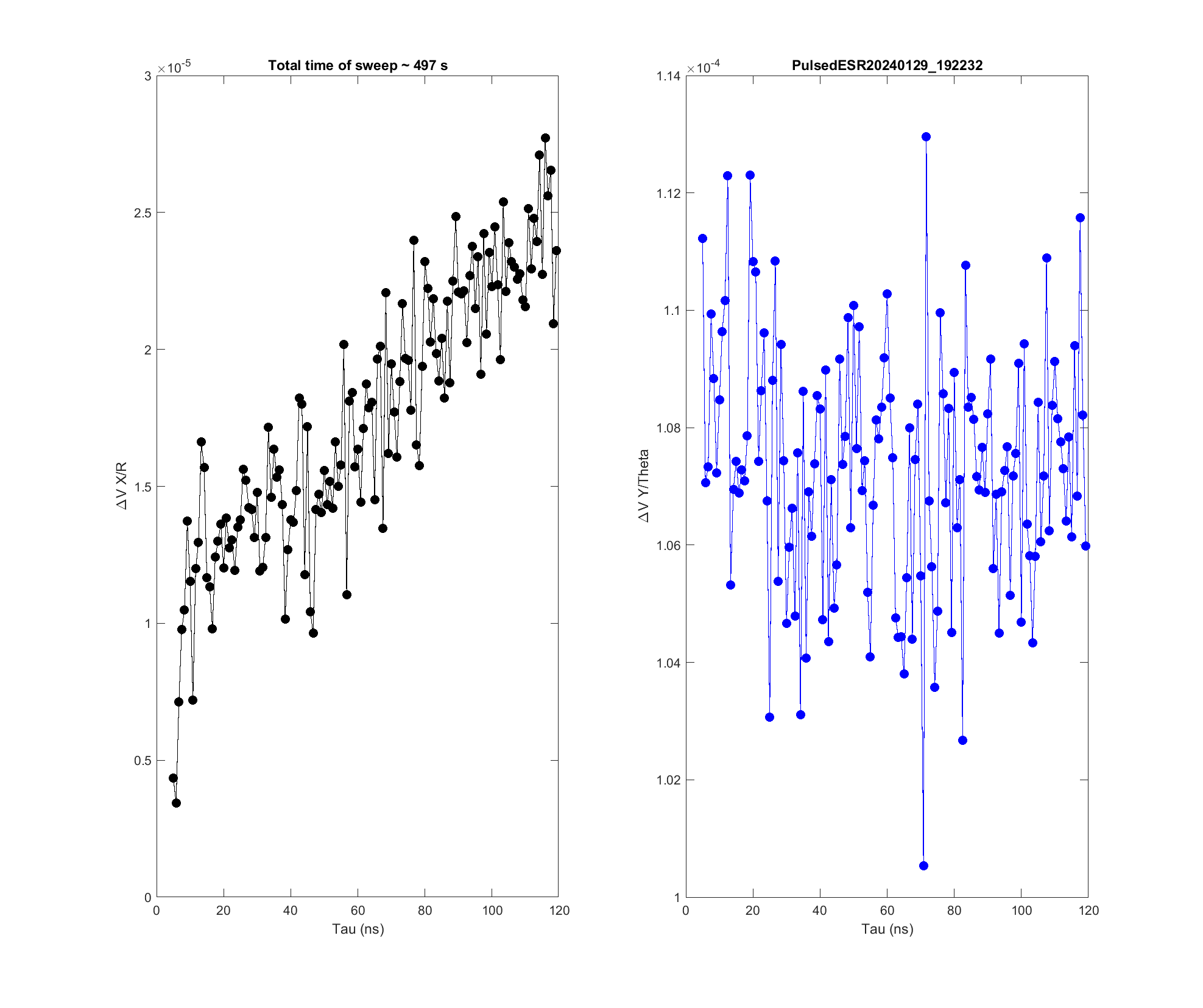

Supplement: Supplementary file 3 — Source Data [file 41467_2025_60409_MOESM3_ESM.zip › SupplementaryData1/Figure3/Fig3ab/PulsedESR20240129_192232.png]

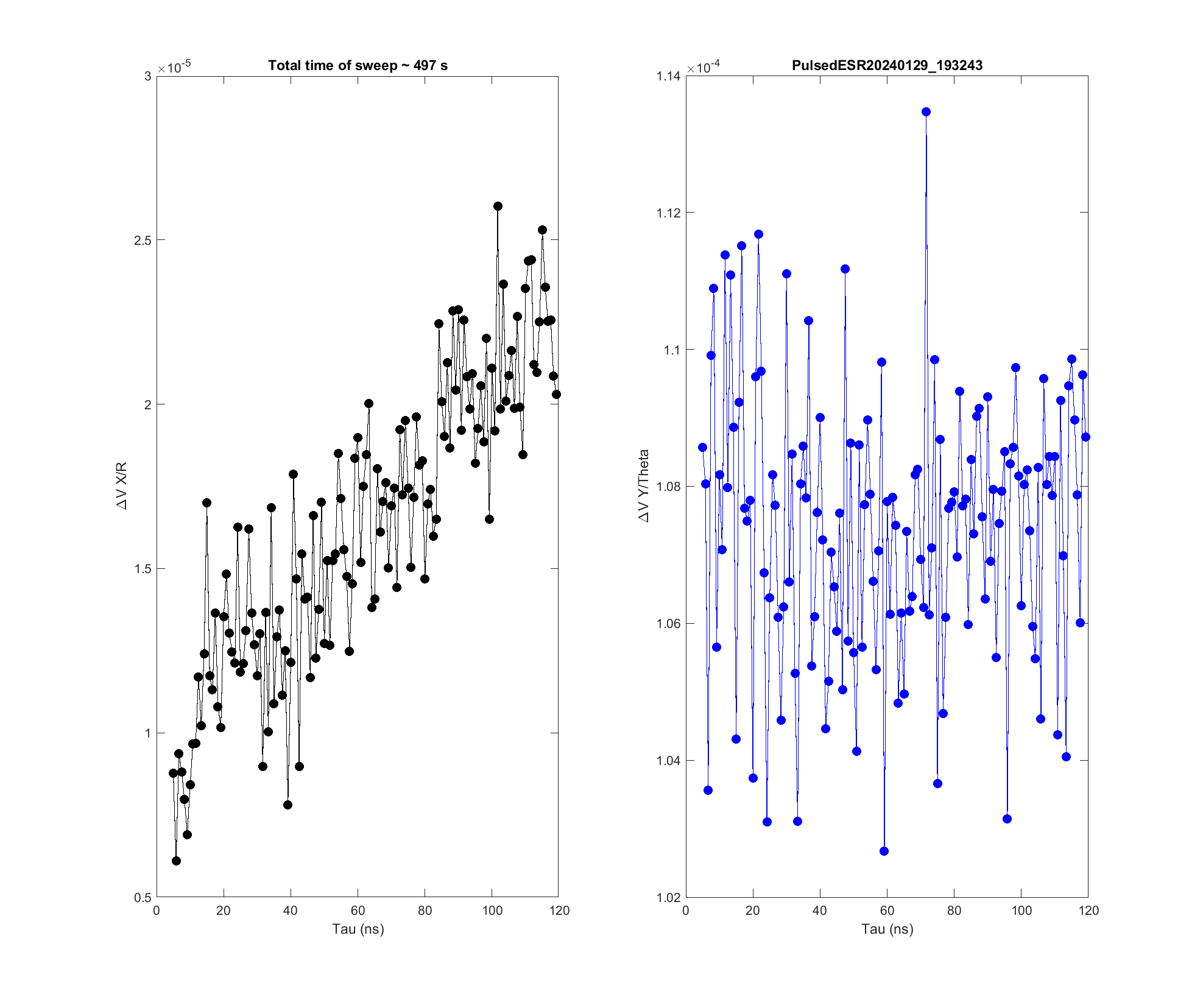

Supplement: Supplementary file 3 — Source Data [file 41467_2025_60409_MOESM3_ESM.zip › SupplementaryData1/Figure3/Fig3ab/PulsedESR20240129_193243.png]

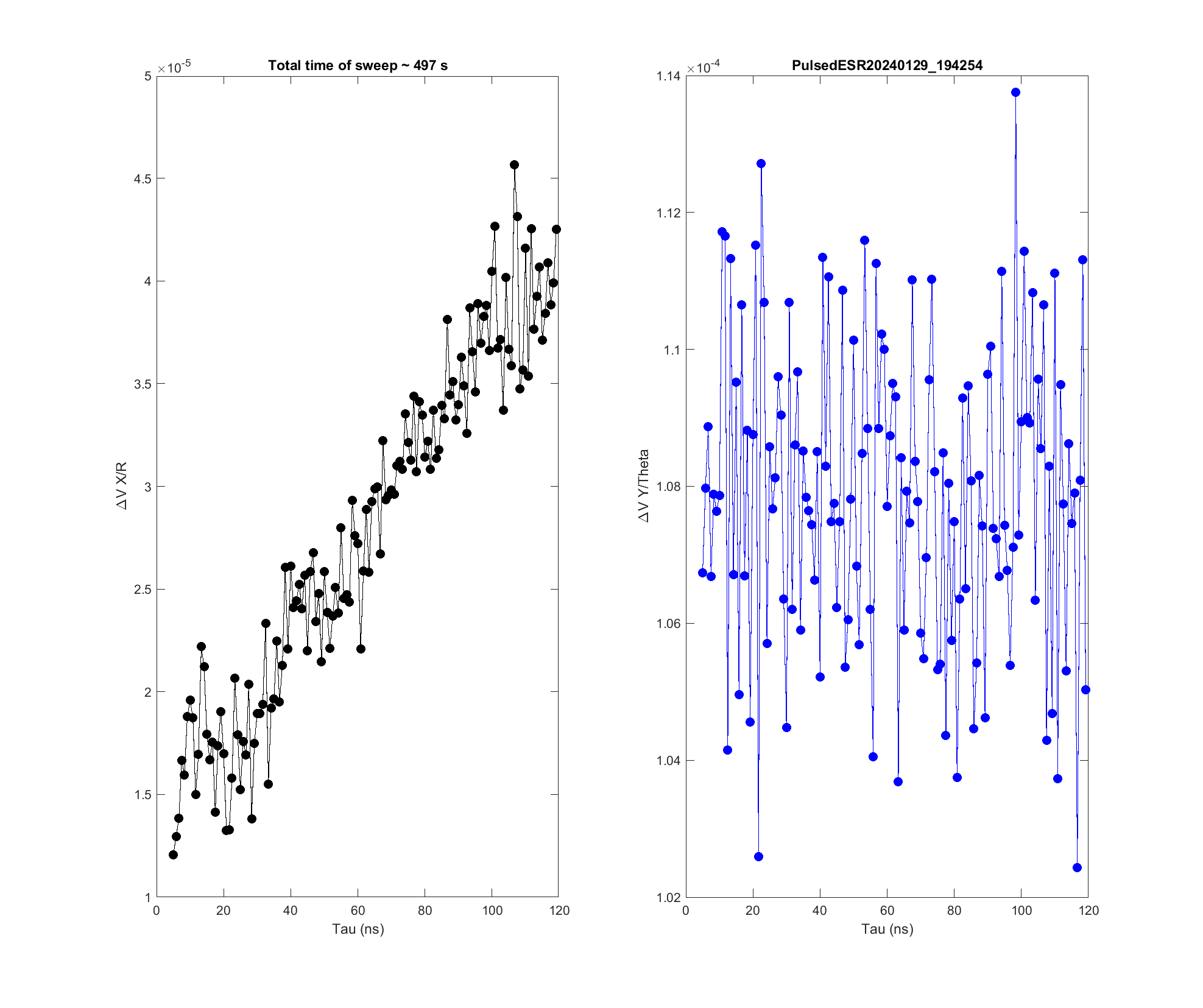

Supplement: Supplementary file 3 — Source Data [file 41467_2025_60409_MOESM3_ESM.zip › SupplementaryData1/Figure3/Fig3ab/PulsedESR20240129_194254.png]

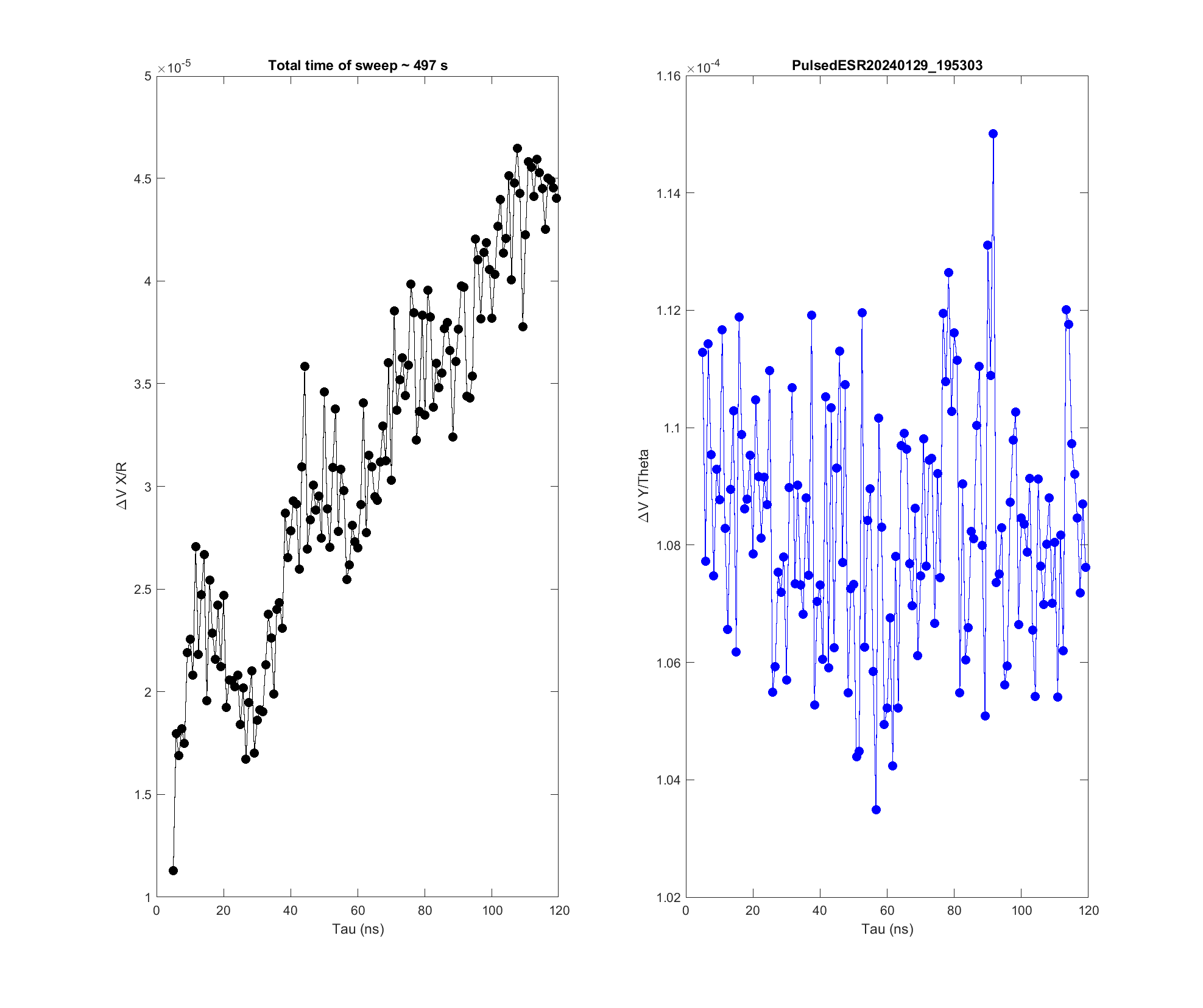

Supplement: Supplementary file 3 — Source Data [file 41467_2025_60409_MOESM3_ESM.zip › SupplementaryData1/Figure3/Fig3ab/PulsedESR20240129_195303.png]

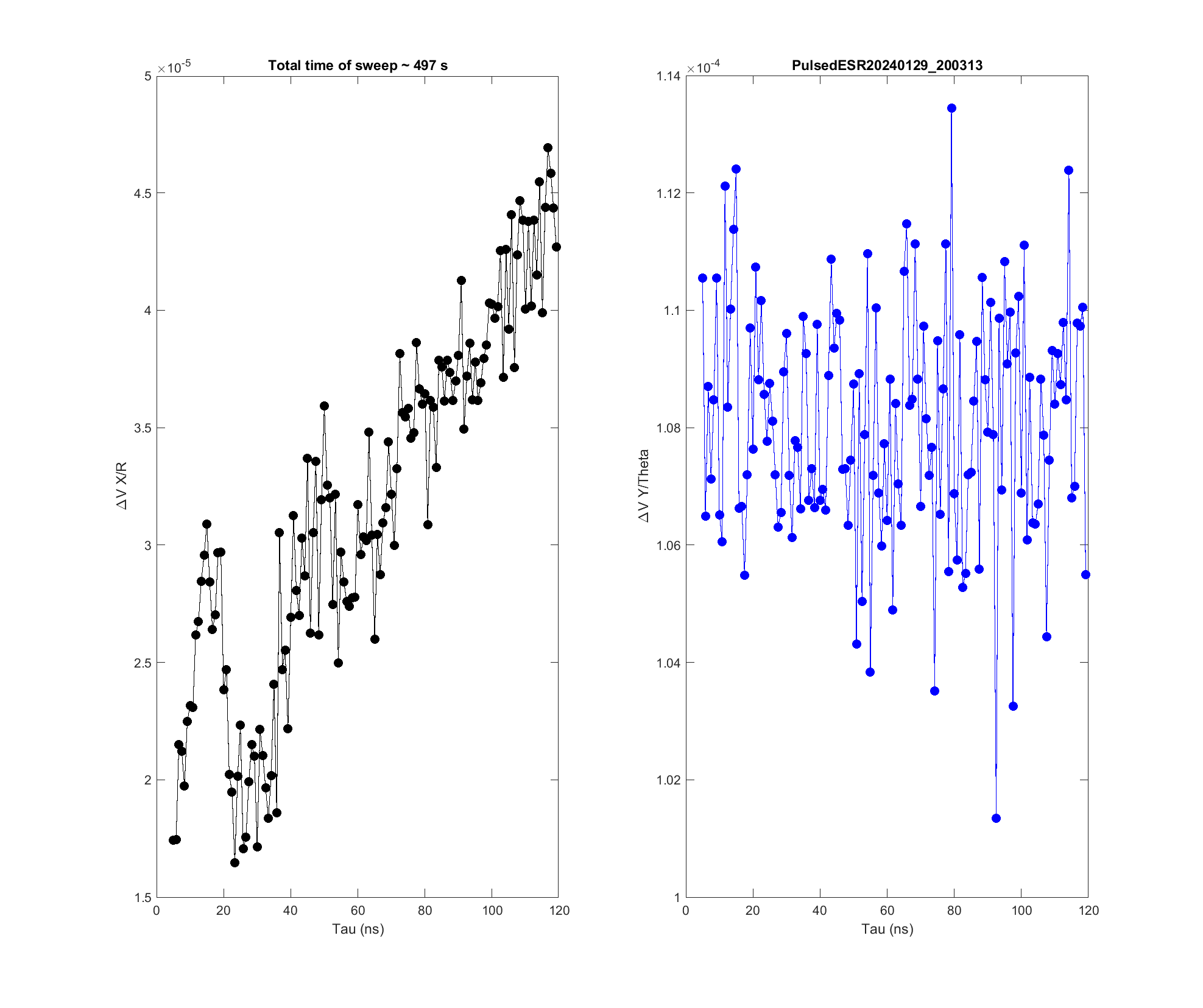

Supplement: Supplementary file 3 — Source Data [file 41467_2025_60409_MOESM3_ESM.zip › SupplementaryData1/Figure3/Fig3ab/PulsedESR20240129_200313.png]

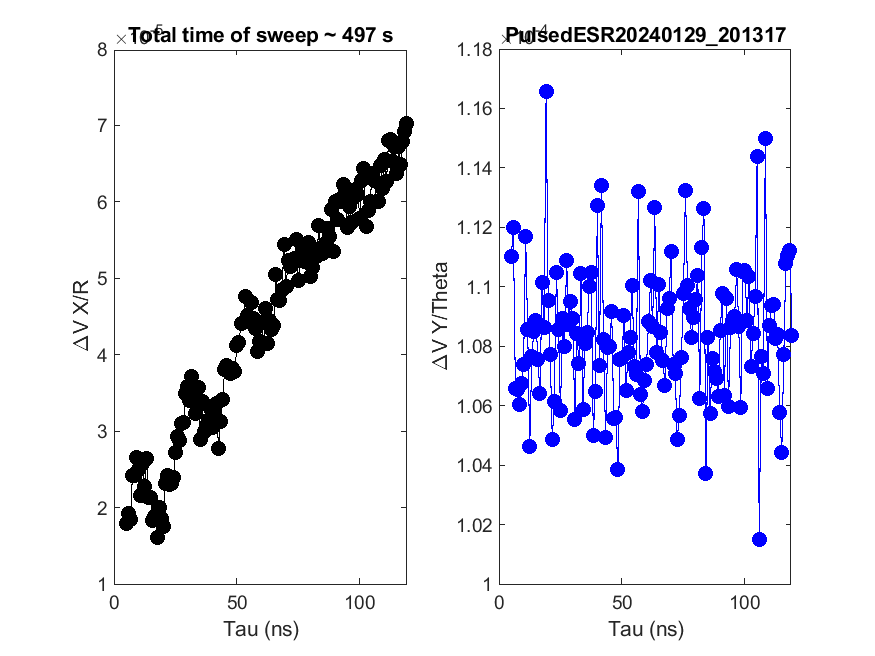

Supplement: Supplementary file 3 — Source Data [file 41467_2025_60409_MOESM3_ESM.zip › SupplementaryData1/Figure3/Fig3ab/PulsedESR20240129_201317.png]

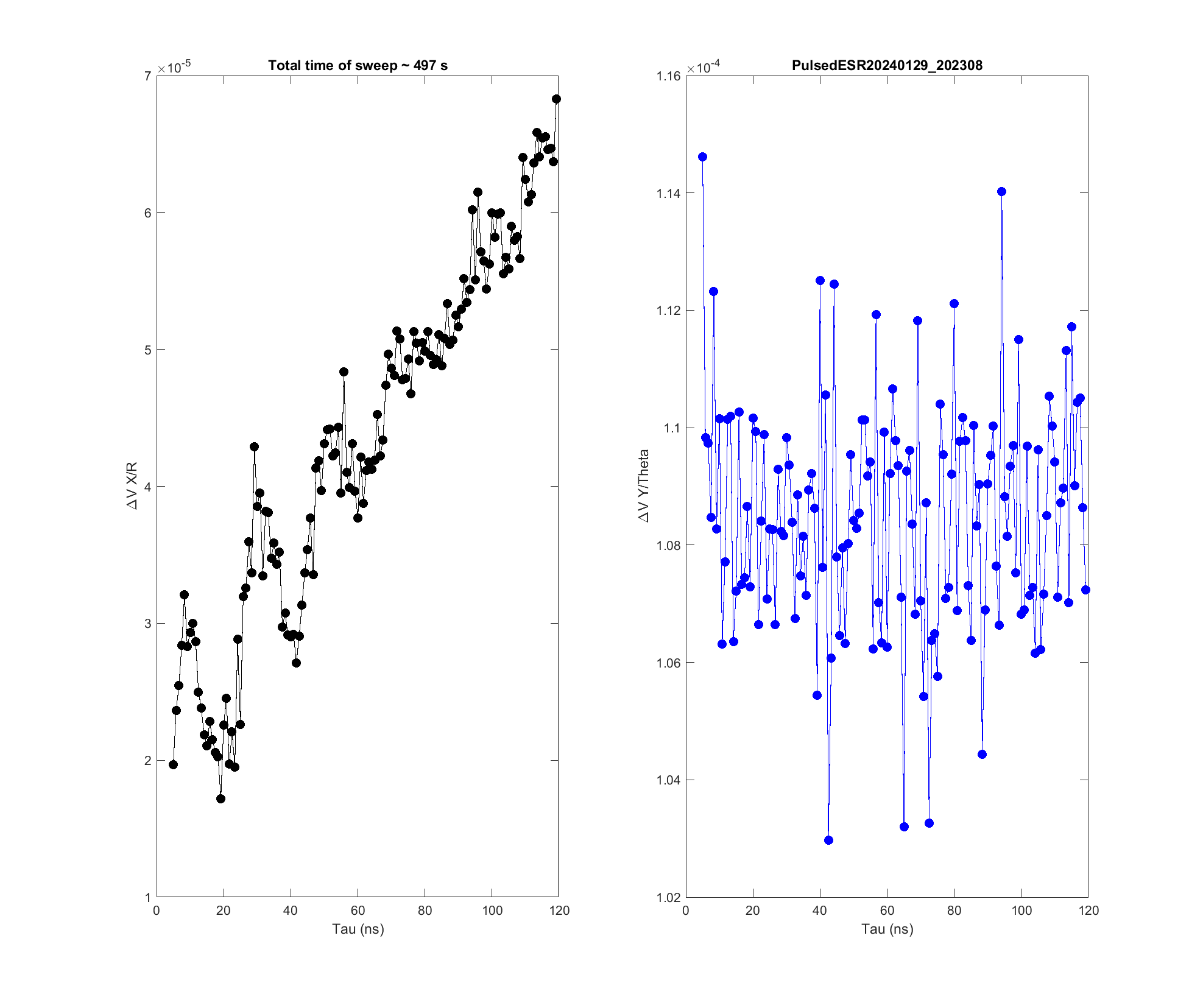

Supplement: Supplementary file 3 — Source Data [file 41467_2025_60409_MOESM3_ESM.zip › SupplementaryData1/Figure3/Fig3ab/PulsedESR20240129_202308.png]

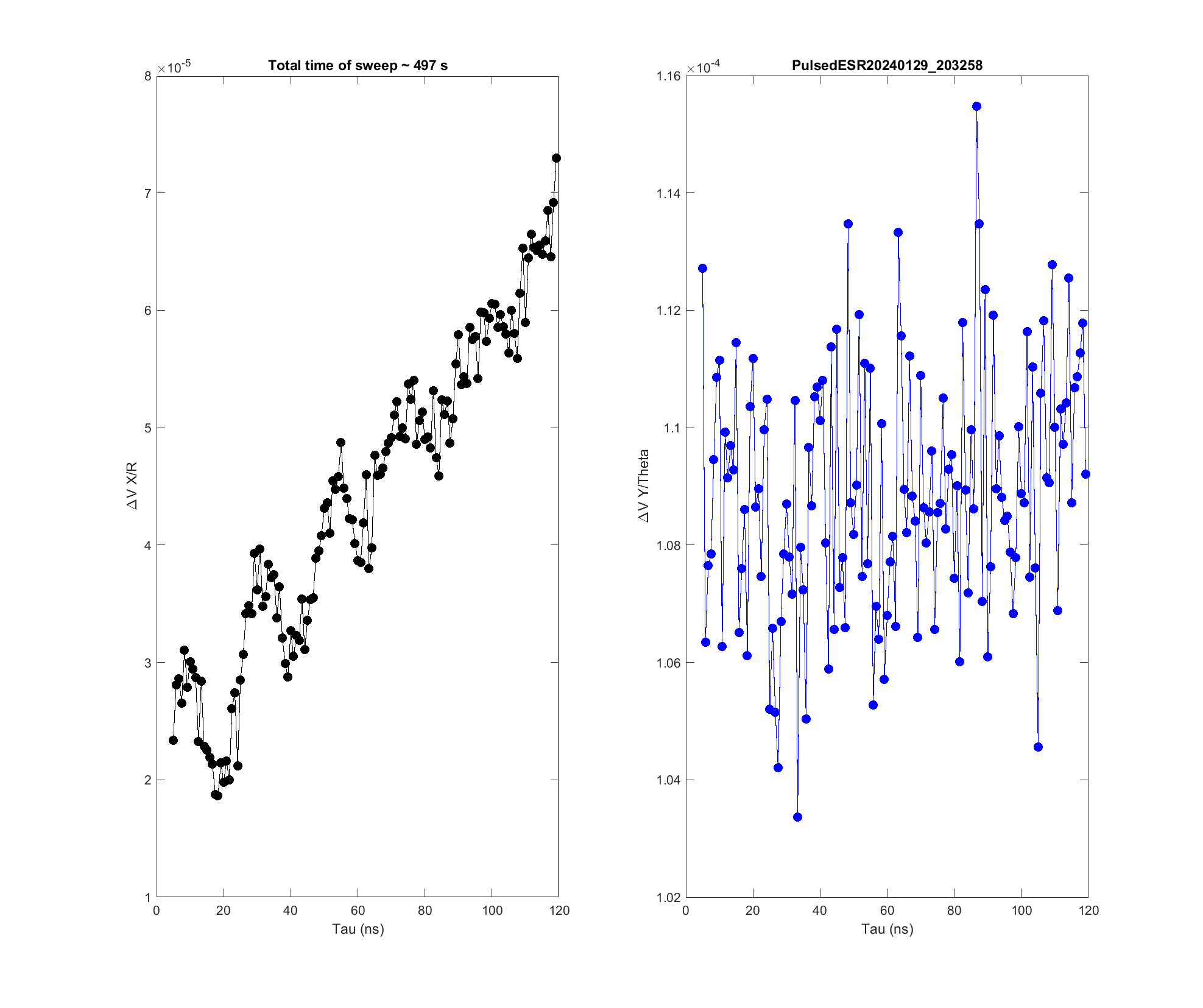

Supplement: Supplementary file 3 — Source Data [file 41467_2025_60409_MOESM3_ESM.zip › SupplementaryData1/Figure3/Fig3ab/PulsedESR20240129_203258.png]

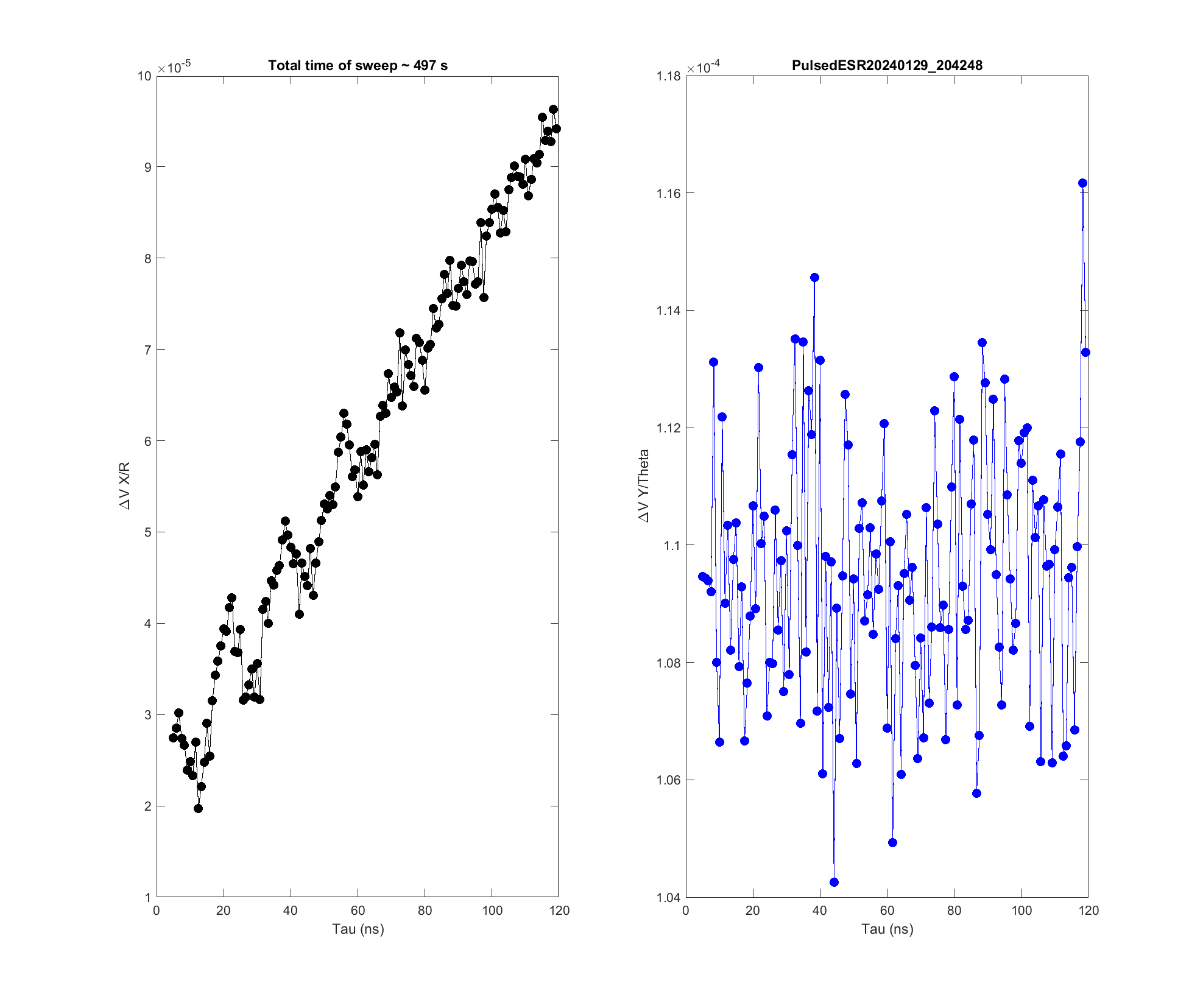

Supplement: Supplementary file 3 — Source Data [file 41467_2025_60409_MOESM3_ESM.zip › SupplementaryData1/Figure3/Fig3ab/PulsedESR20240129_204248.png]

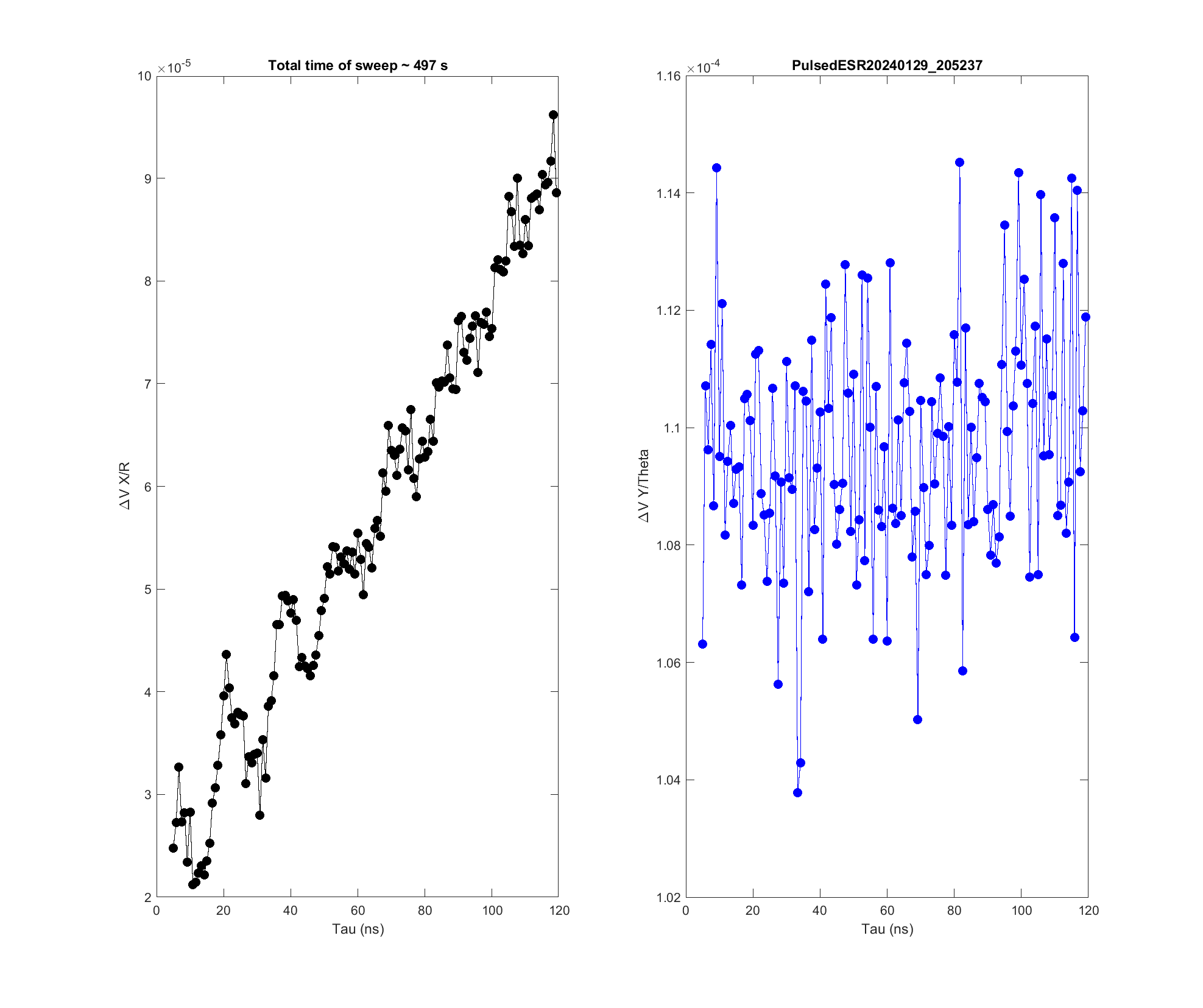

Supplement: Supplementary file 3 — Source Data [file 41467_2025_60409_MOESM3_ESM.zip › SupplementaryData1/Figure3/Fig3ab/PulsedESR20240129_205237.png]

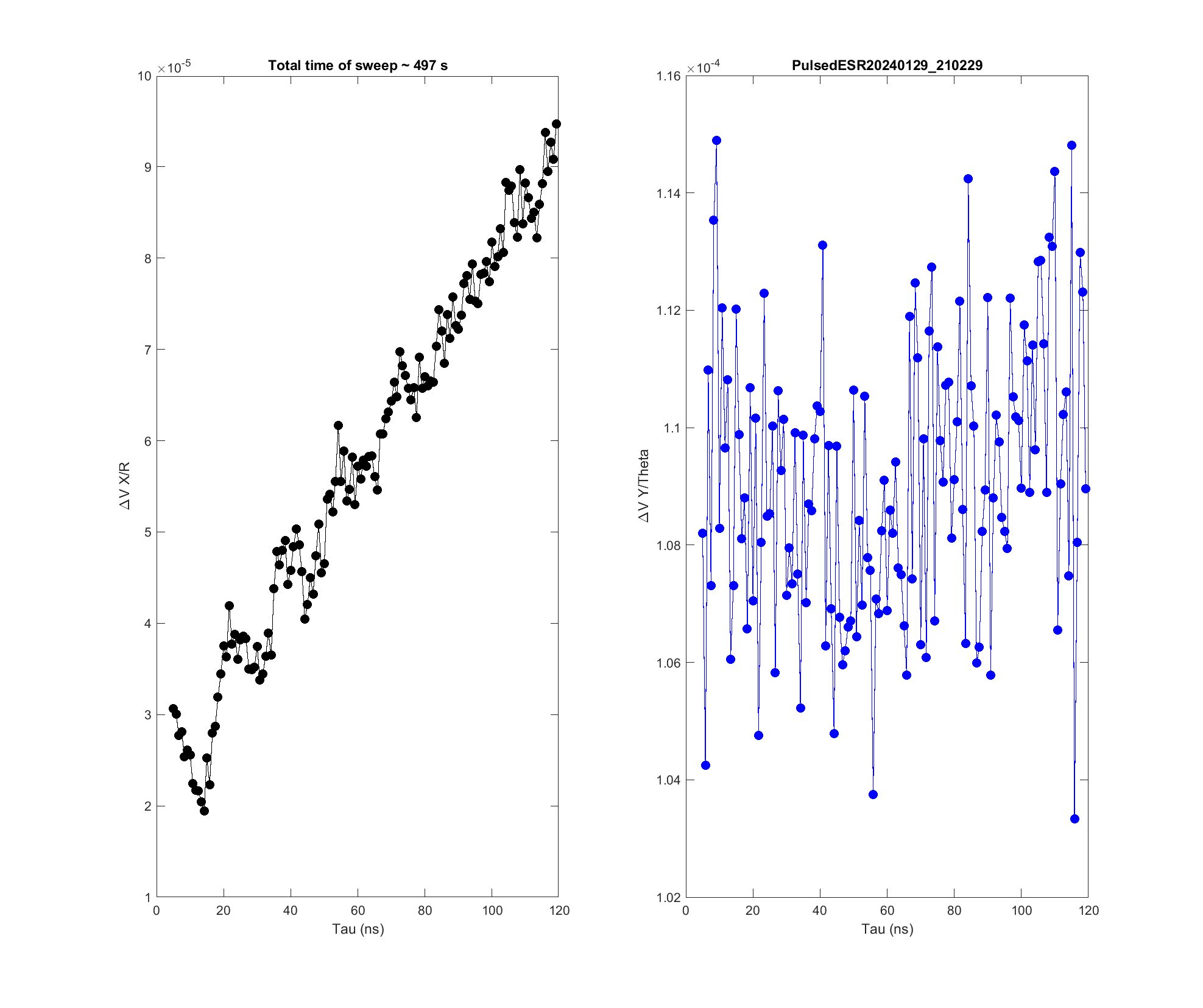

Supplement: Supplementary file 3 — Source Data [file 41467_2025_60409_MOESM3_ESM.zip › SupplementaryData1/Figure3/Fig3ab/PulsedESR20240129_210229.png]

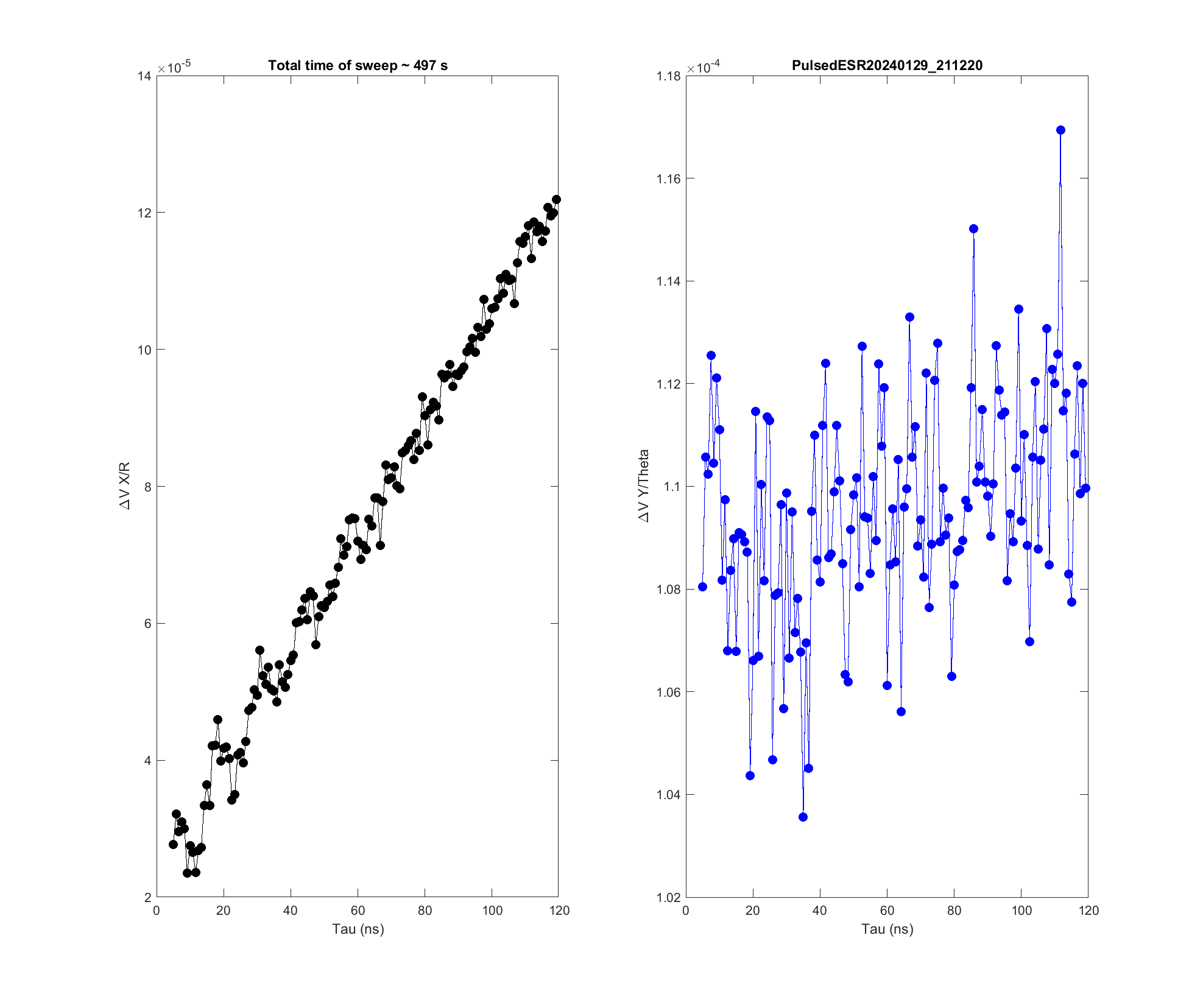

Supplement: Supplementary file 3 — Source Data [file 41467_2025_60409_MOESM3_ESM.zip › SupplementaryData1/Figure3/Fig3ab/PulsedESR20240129_211220.png]

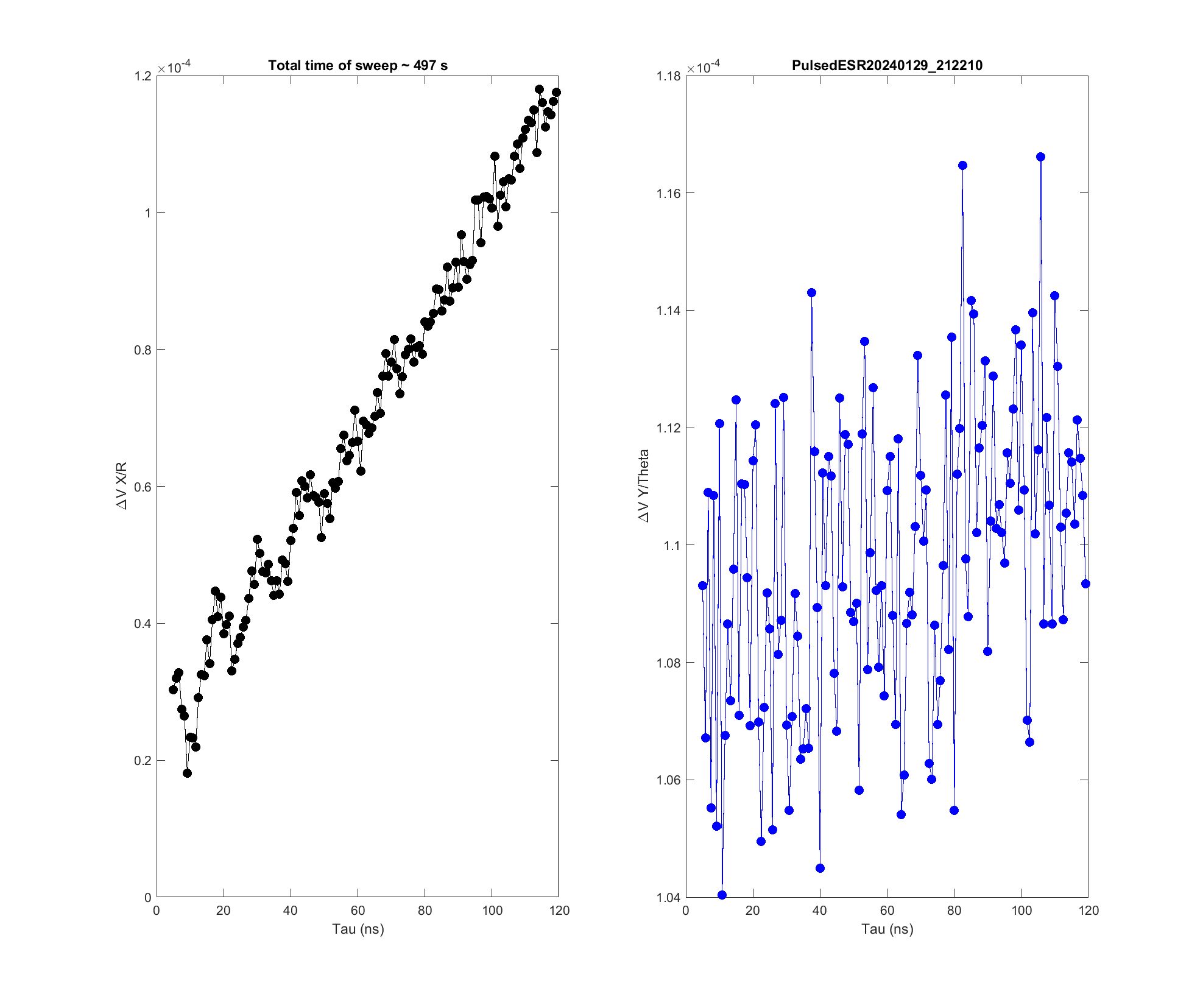

Supplement: Supplementary file 3 — Source Data [file 41467_2025_60409_MOESM3_ESM.zip › SupplementaryData1/Figure3/Fig3ab/PulsedESR20240129_212210.png]

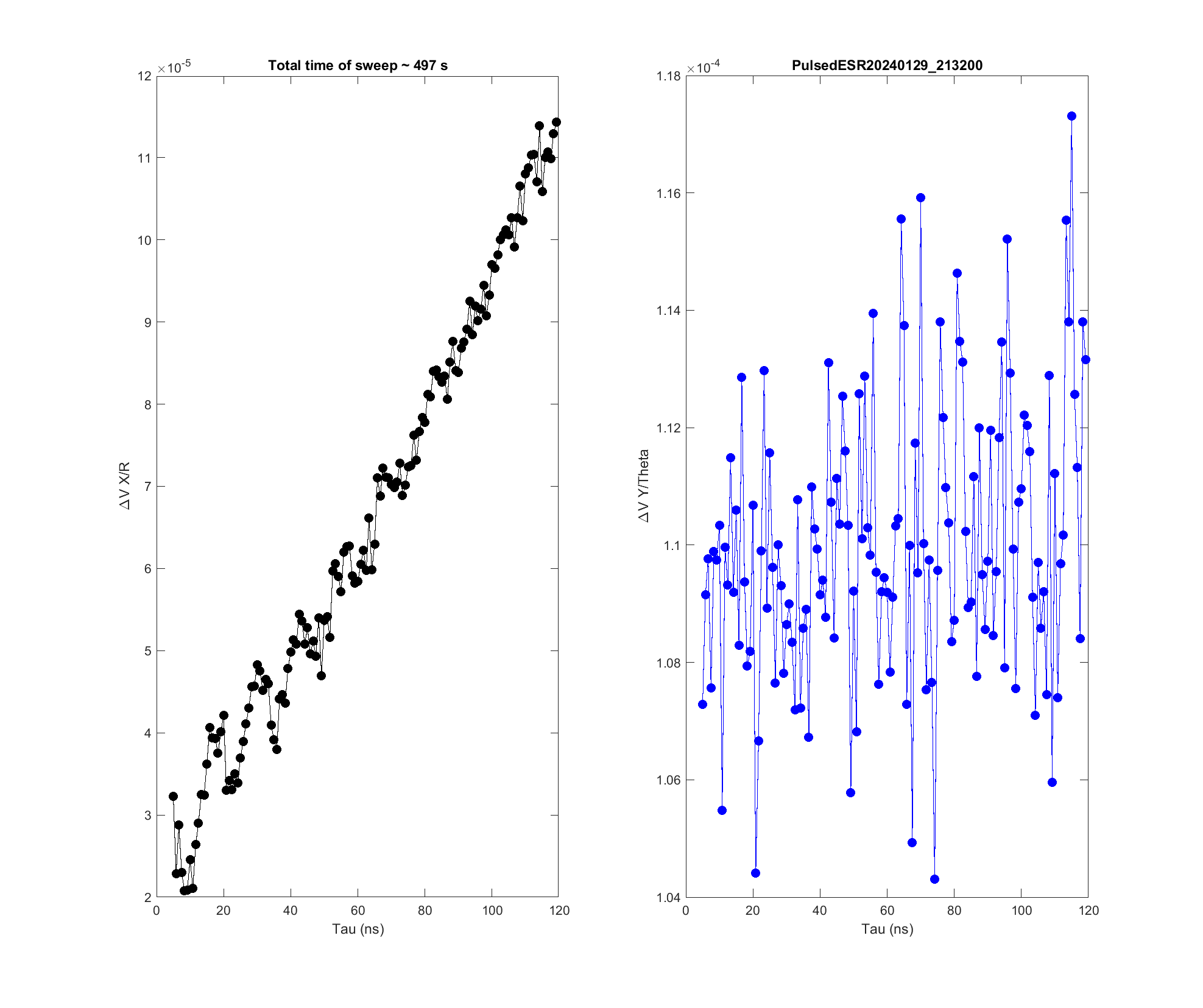

Supplement: Supplementary file 3 — Source Data [file 41467_2025_60409_MOESM3_ESM.zip › SupplementaryData1/Figure3/Fig3ab/PulsedESR20240129_213200.png]

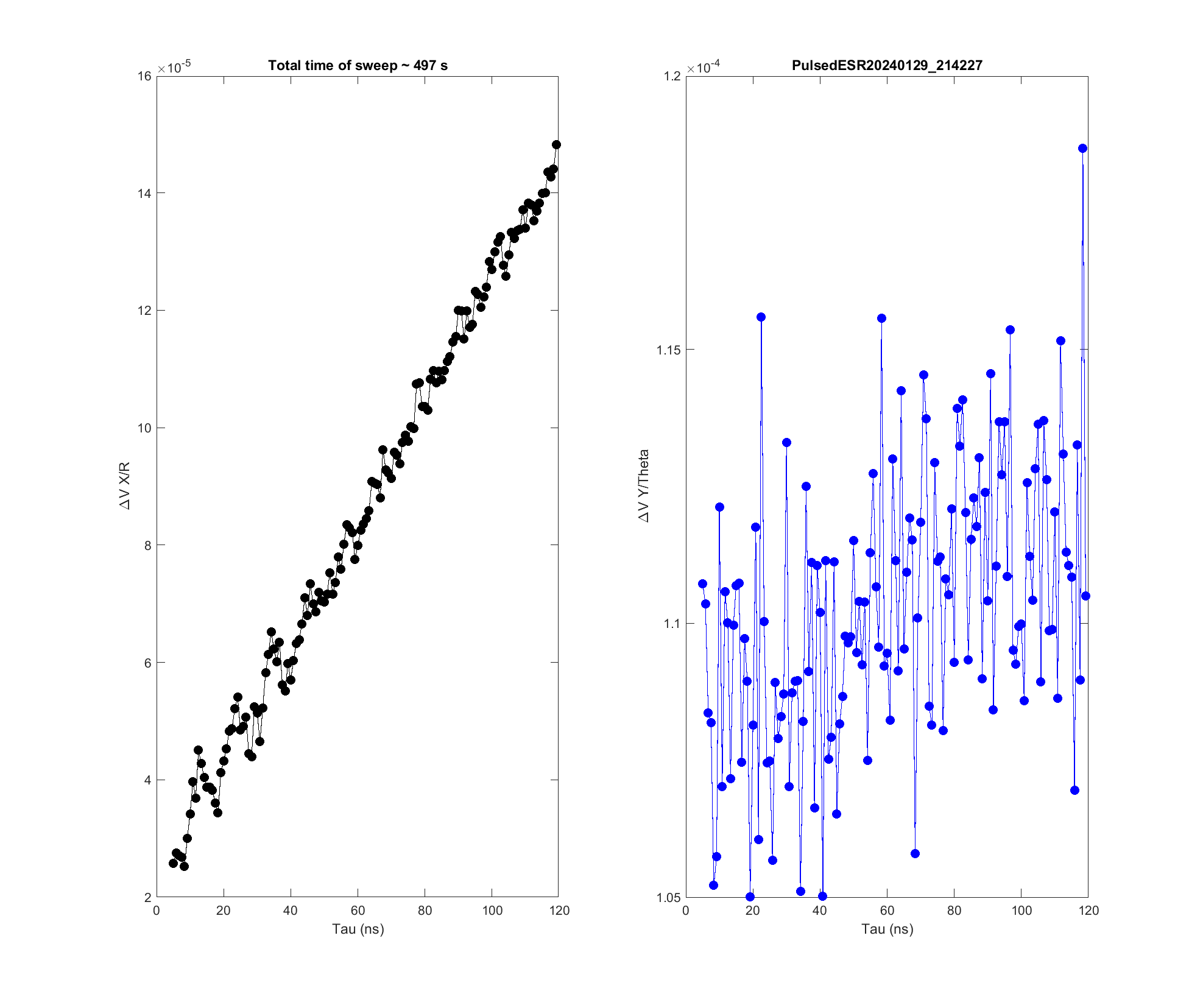

Supplement: Supplementary file 3 — Source Data [file 41467_2025_60409_MOESM3_ESM.zip › SupplementaryData1/Figure3/Fig3ab/PulsedESR20240129_214227.png]

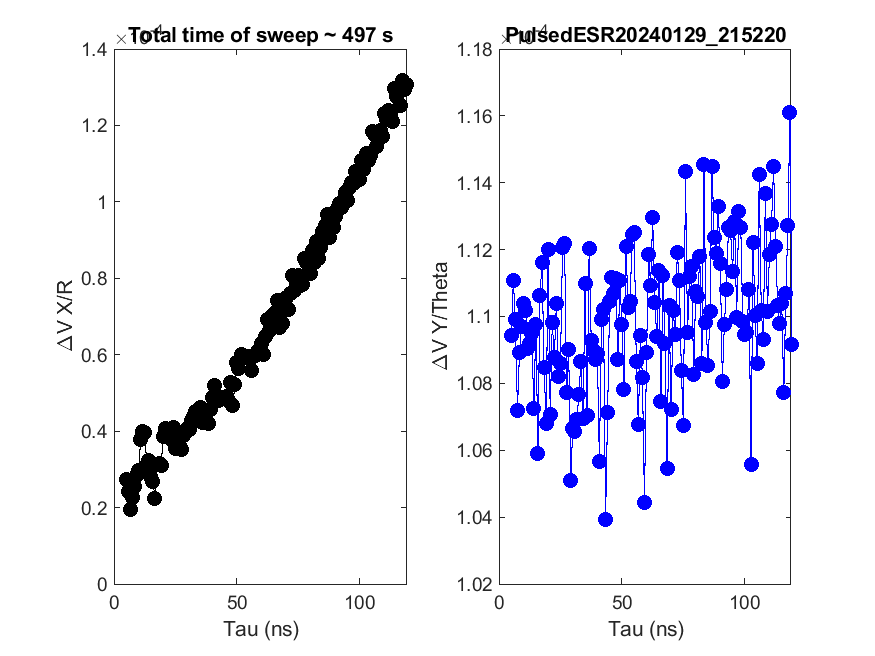

Supplement: Supplementary file 3 — Source Data [file 41467_2025_60409_MOESM3_ESM.zip › SupplementaryData1/Figure3/Fig3ab/PulsedESR20240129_215220.png]

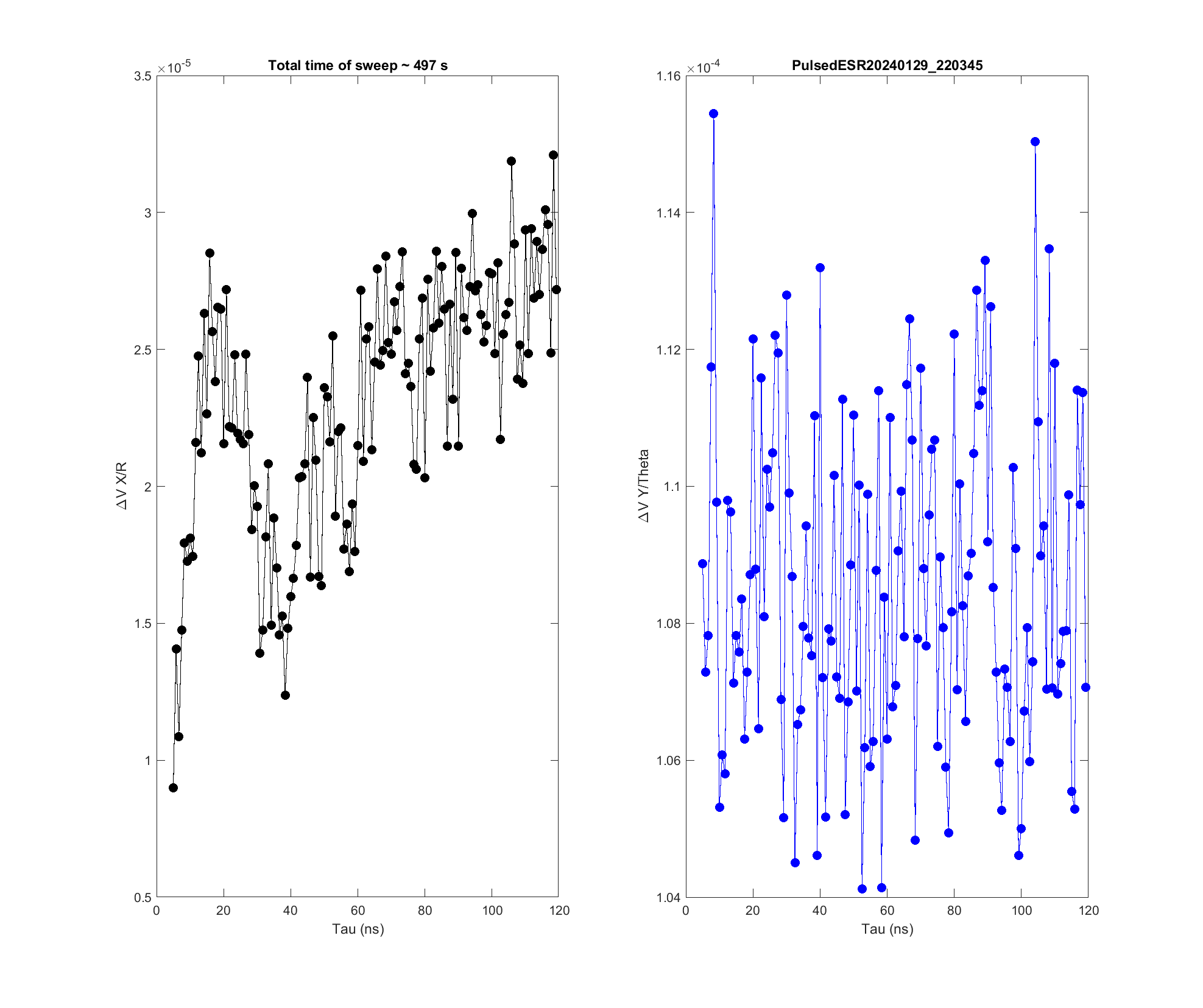

Supplement: Supplementary file 3 — Source Data [file 41467_2025_60409_MOESM3_ESM.zip › SupplementaryData1/Figure3/Fig3ab/PulsedESR20240129_220345.png]

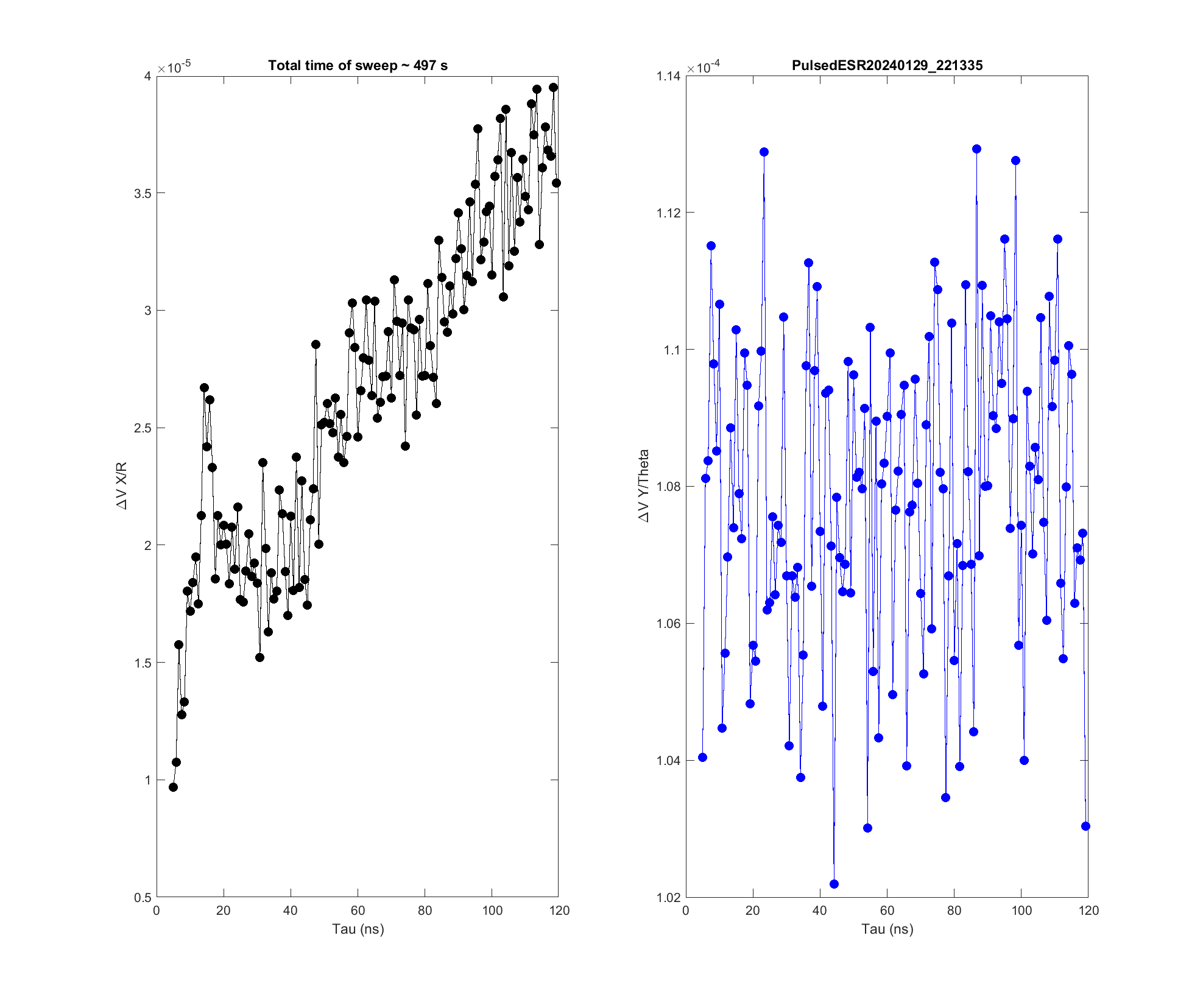

Supplement: Supplementary file 3 — Source Data [file 41467_2025_60409_MOESM3_ESM.zip › SupplementaryData1/Figure3/Fig3ab/PulsedESR20240129_221335.png]

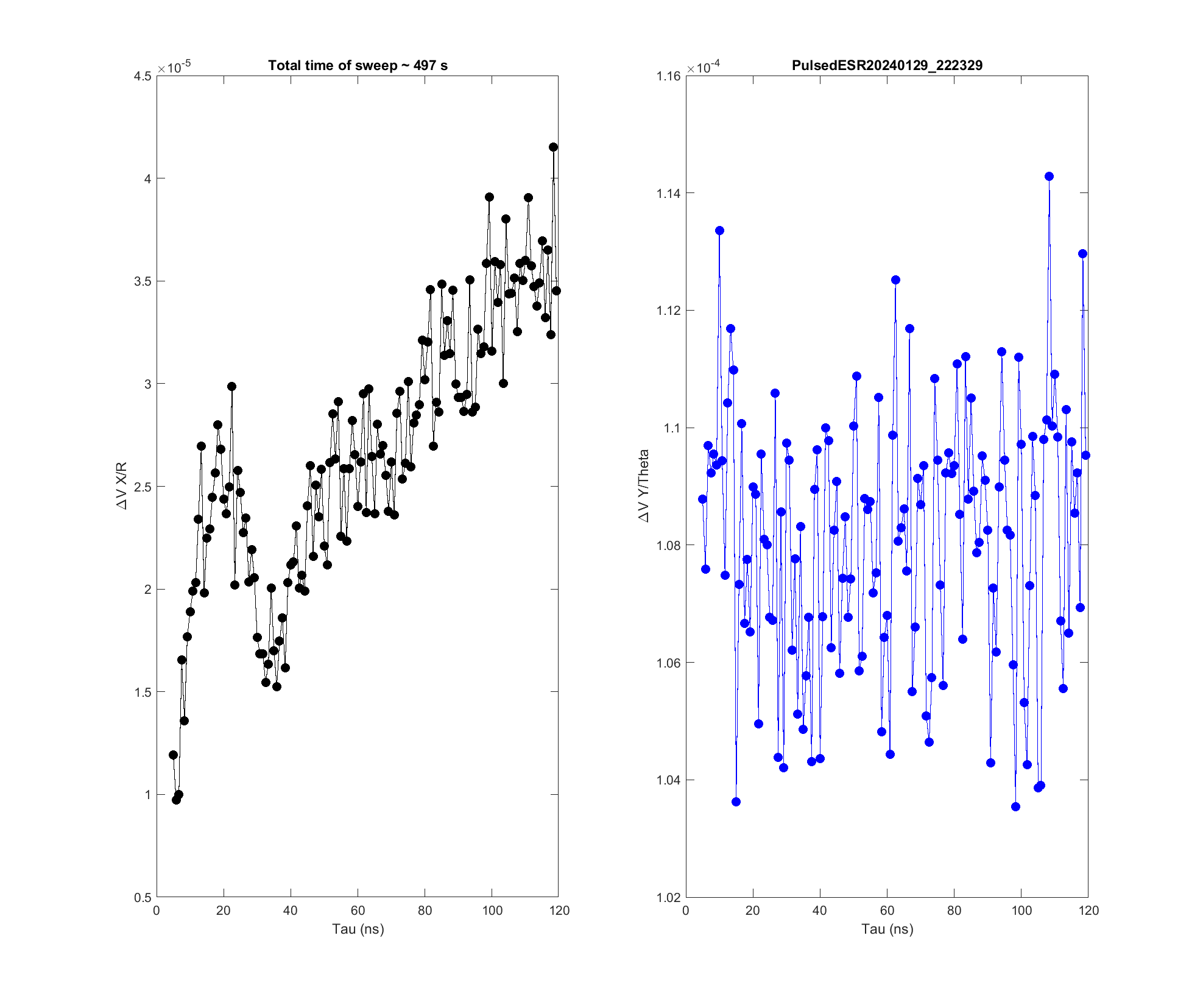

Supplement: Supplementary file 3 — Source Data [file 41467_2025_60409_MOESM3_ESM.zip › SupplementaryData1/Figure3/Fig3ab/PulsedESR20240129_222329.png]

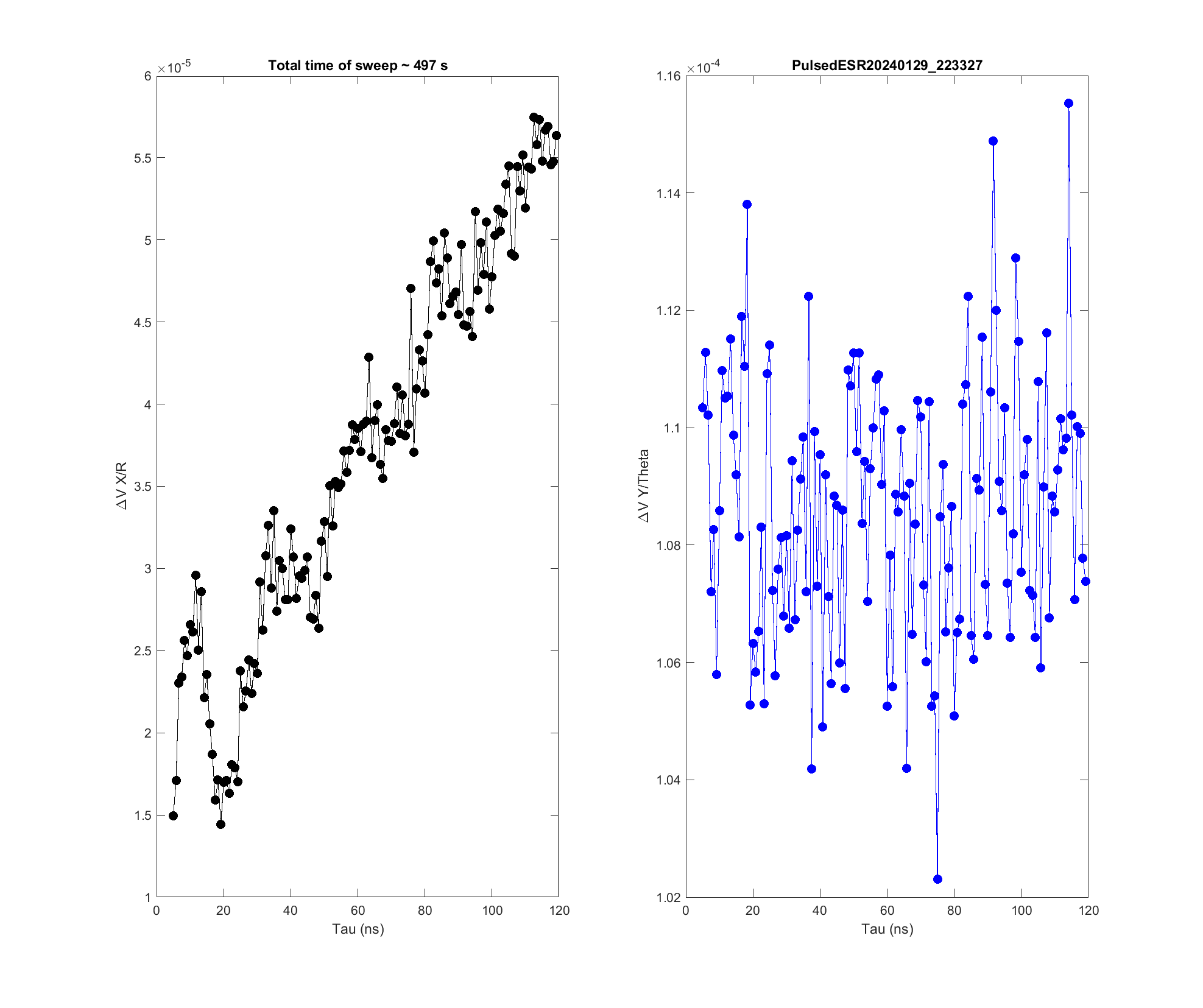

Supplement: Supplementary file 3 — Source Data [file 41467_2025_60409_MOESM3_ESM.zip › SupplementaryData1/Figure3/Fig3ab/PulsedESR20240129_223327.png]

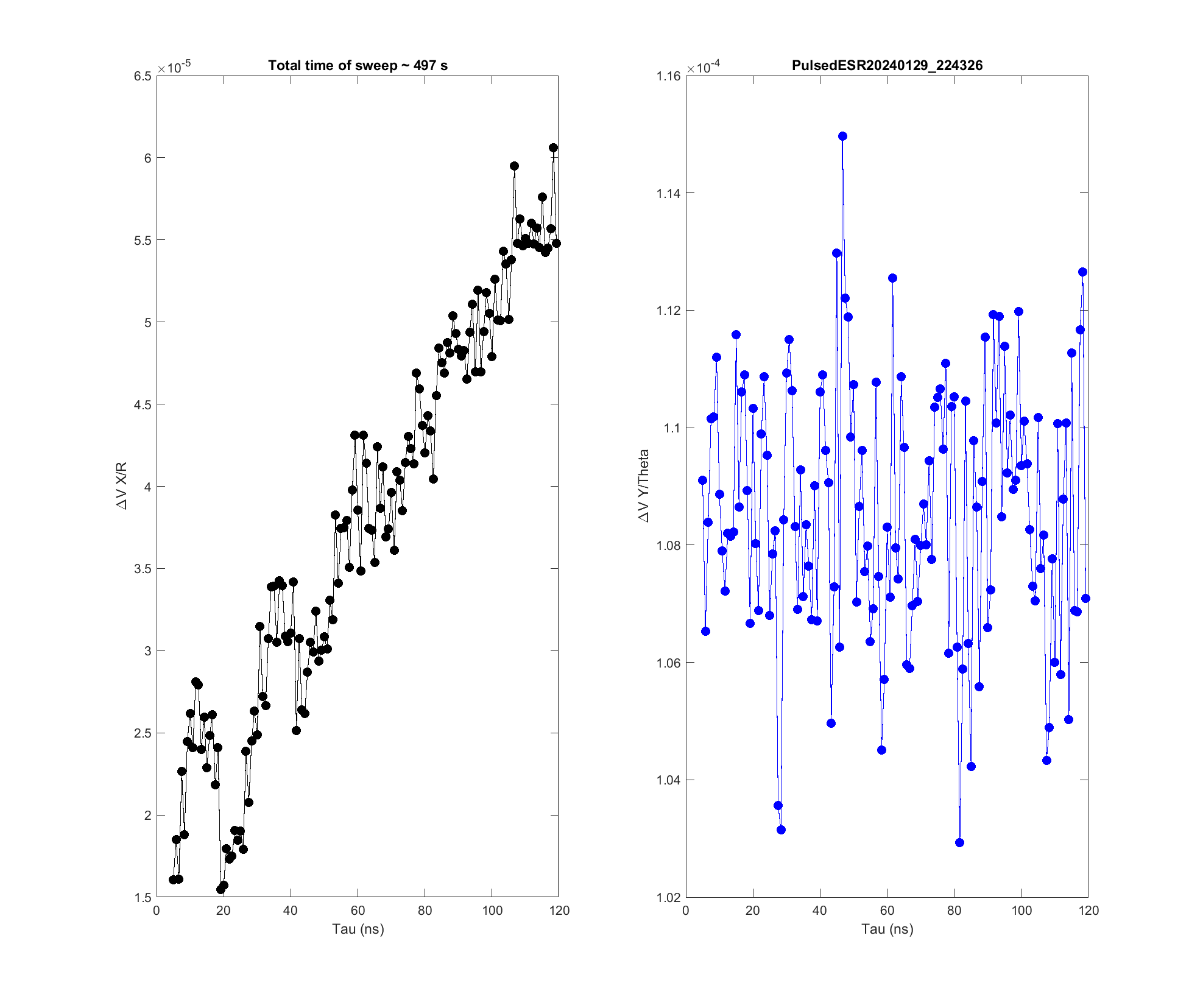

Supplement: Supplementary file 3 — Source Data [file 41467_2025_60409_MOESM3_ESM.zip › SupplementaryData1/Figure3/Fig3ab/PulsedESR20240129_224326.png]

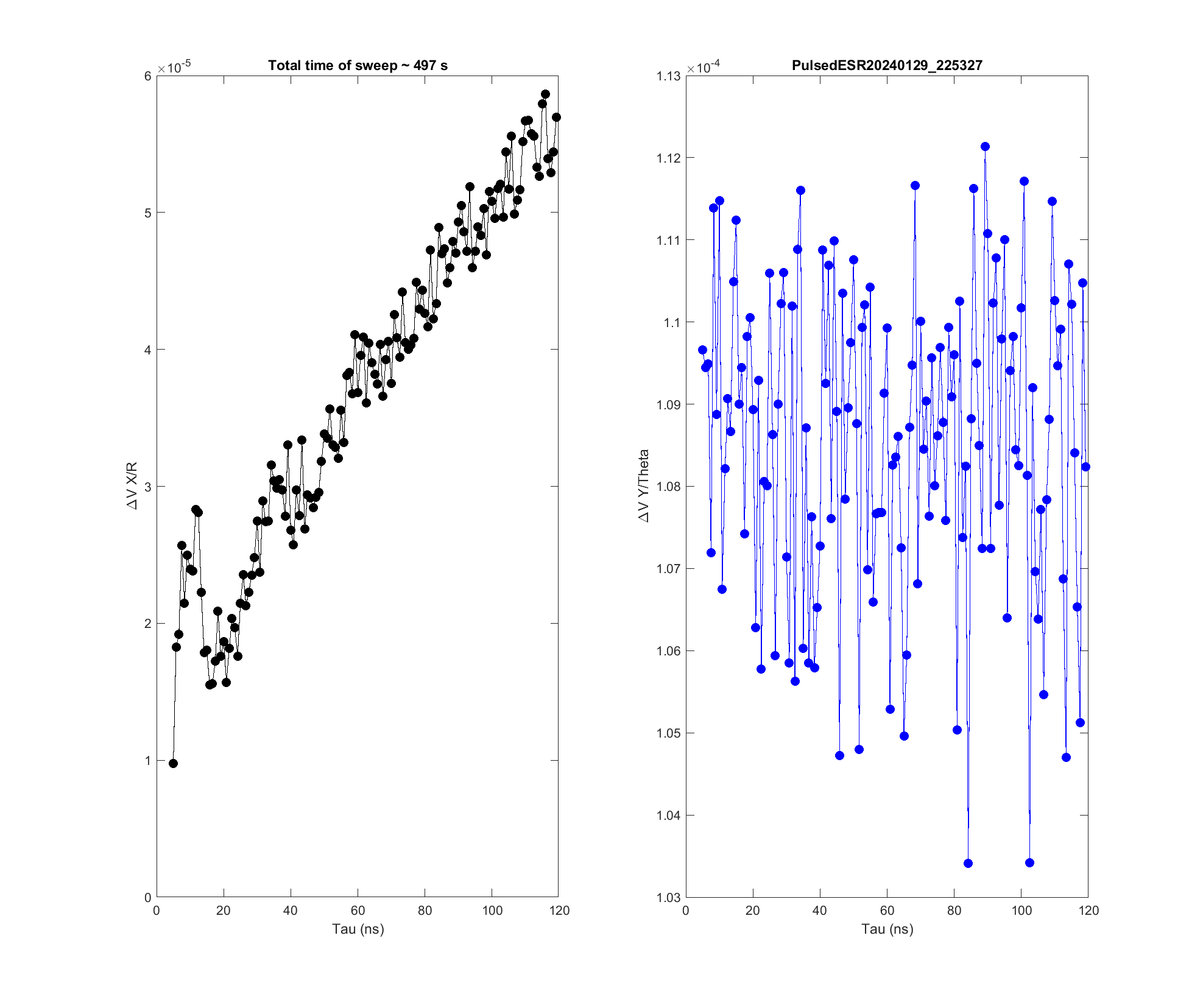

Supplement: Supplementary file 3 — Source Data [file 41467_2025_60409_MOESM3_ESM.zip › SupplementaryData1/Figure3/Fig3ab/PulsedESR20240129_225327.png]

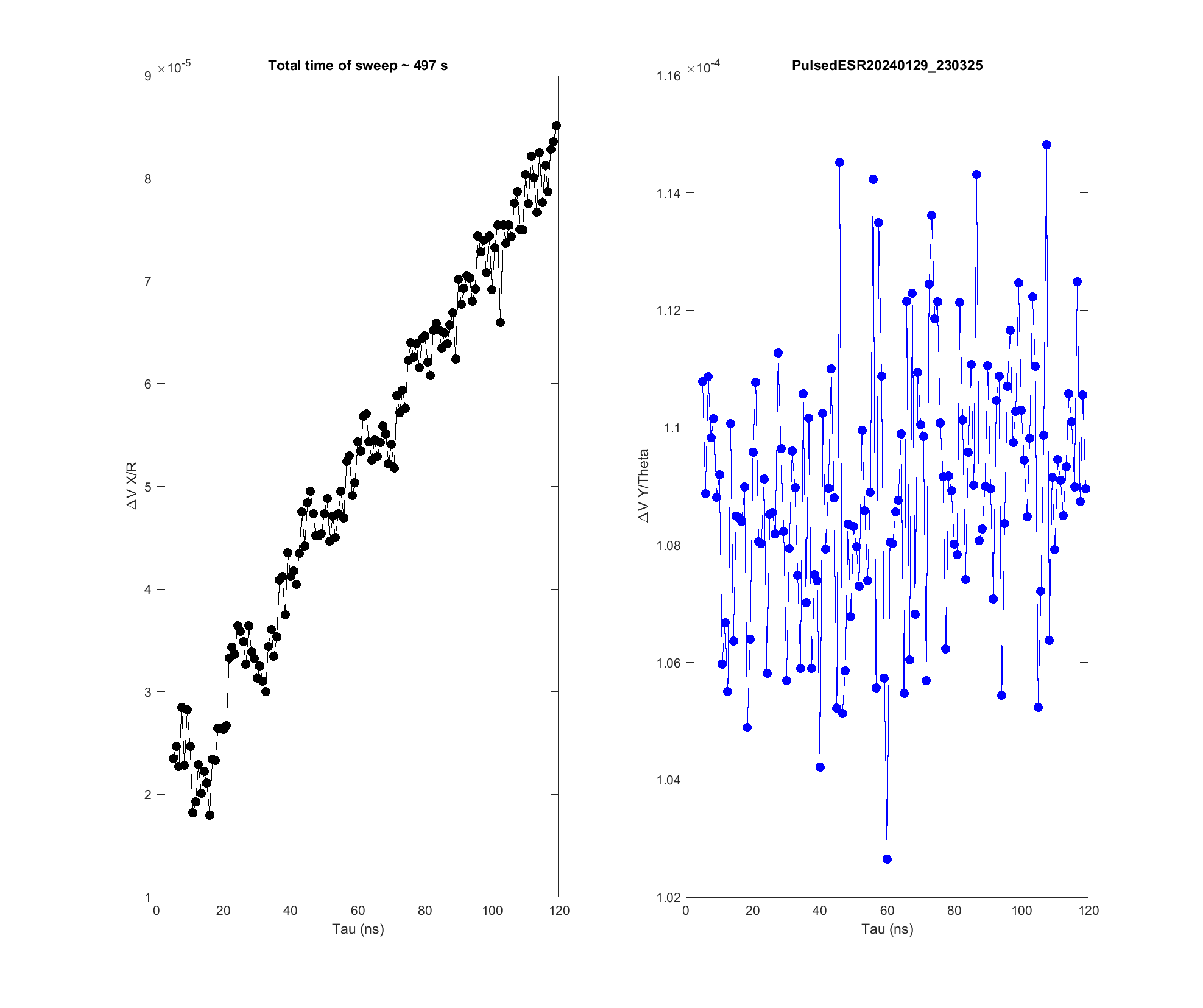

Supplement: Supplementary file 3 — Source Data [file 41467_2025_60409_MOESM3_ESM.zip › SupplementaryData1/Figure3/Fig3ab/PulsedESR20240129_230325.png]

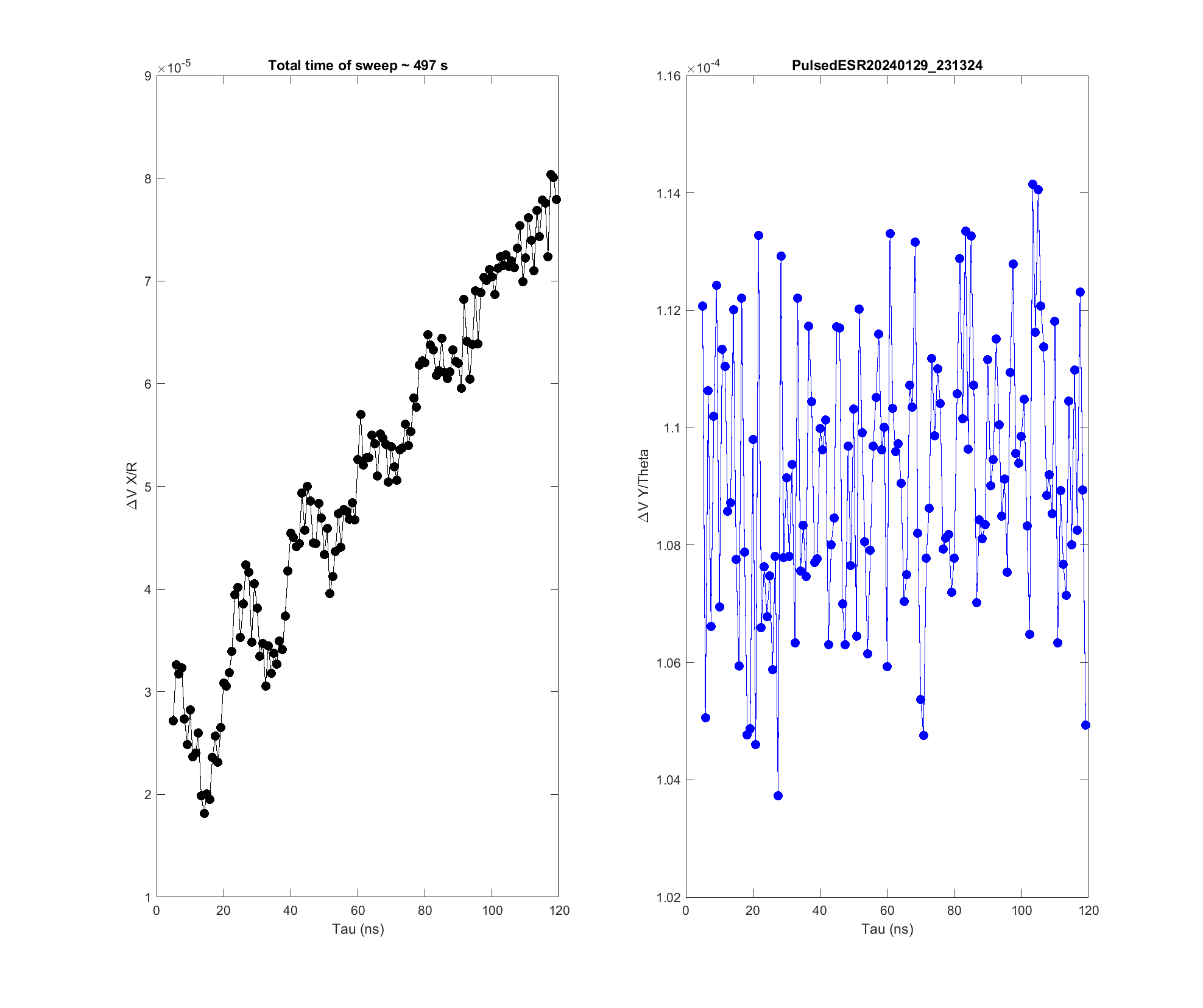

Supplement: Supplementary file 3 — Source Data [file 41467_2025_60409_MOESM3_ESM.zip › SupplementaryData1/Figure3/Fig3ab/PulsedESR20240129_231324.png]

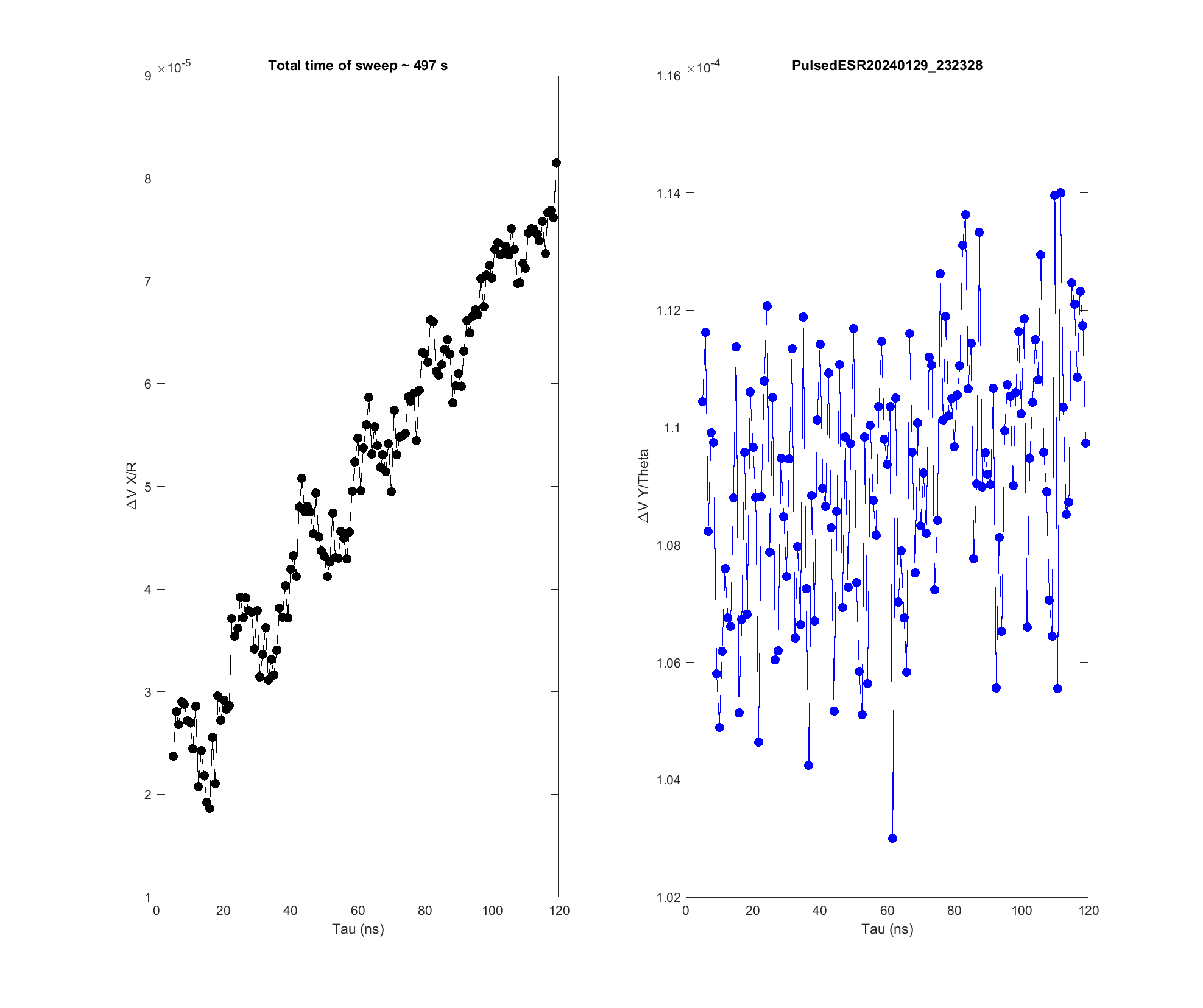

Supplement: Supplementary file 3 — Source Data [file 41467_2025_60409_MOESM3_ESM.zip › SupplementaryData1/Figure3/Fig3ab/PulsedESR20240129_232328.png]

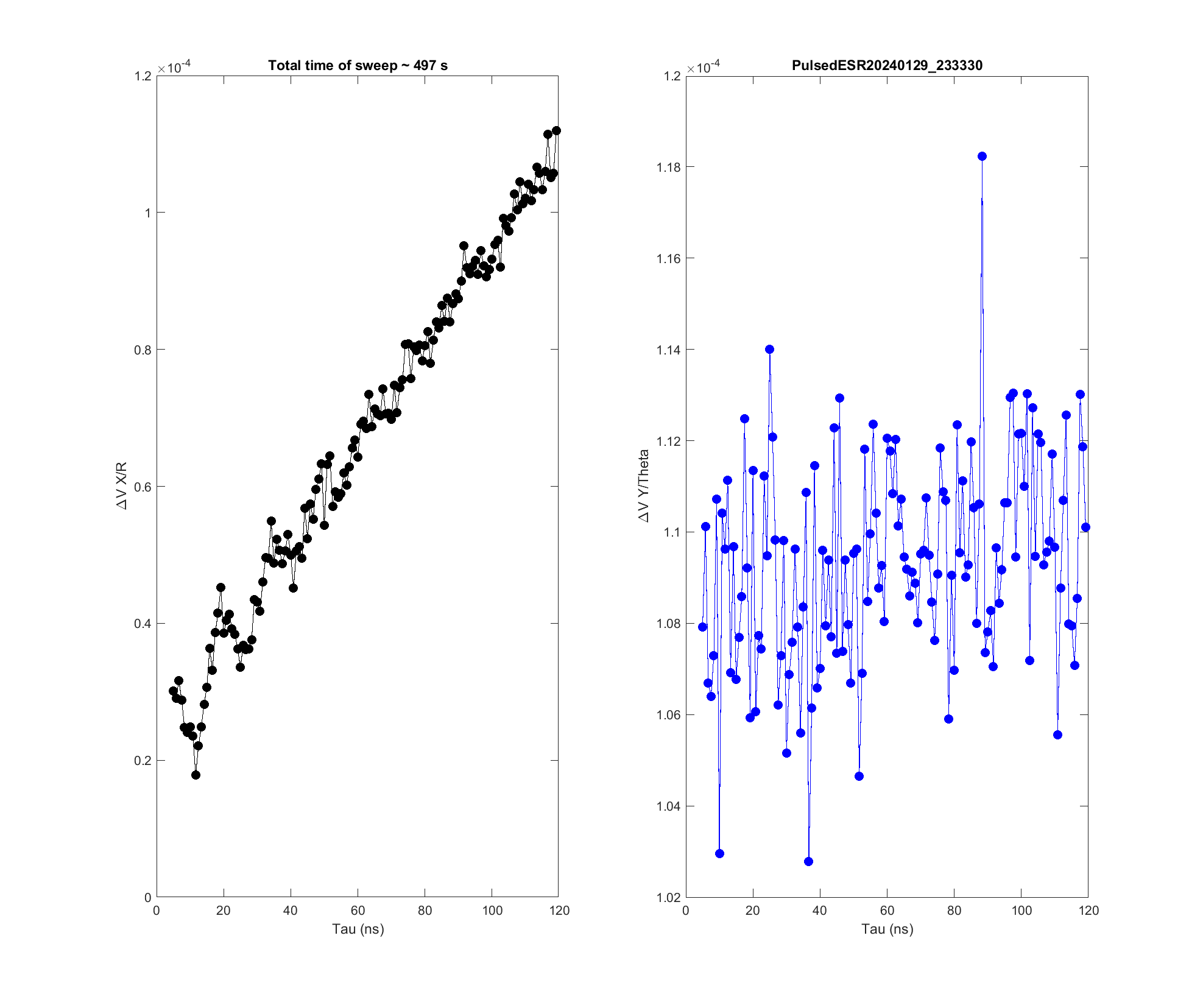

Supplement: Supplementary file 3 — Source Data [file 41467_2025_60409_MOESM3_ESM.zip › SupplementaryData1/Figure3/Fig3ab/PulsedESR20240129_233330.png]

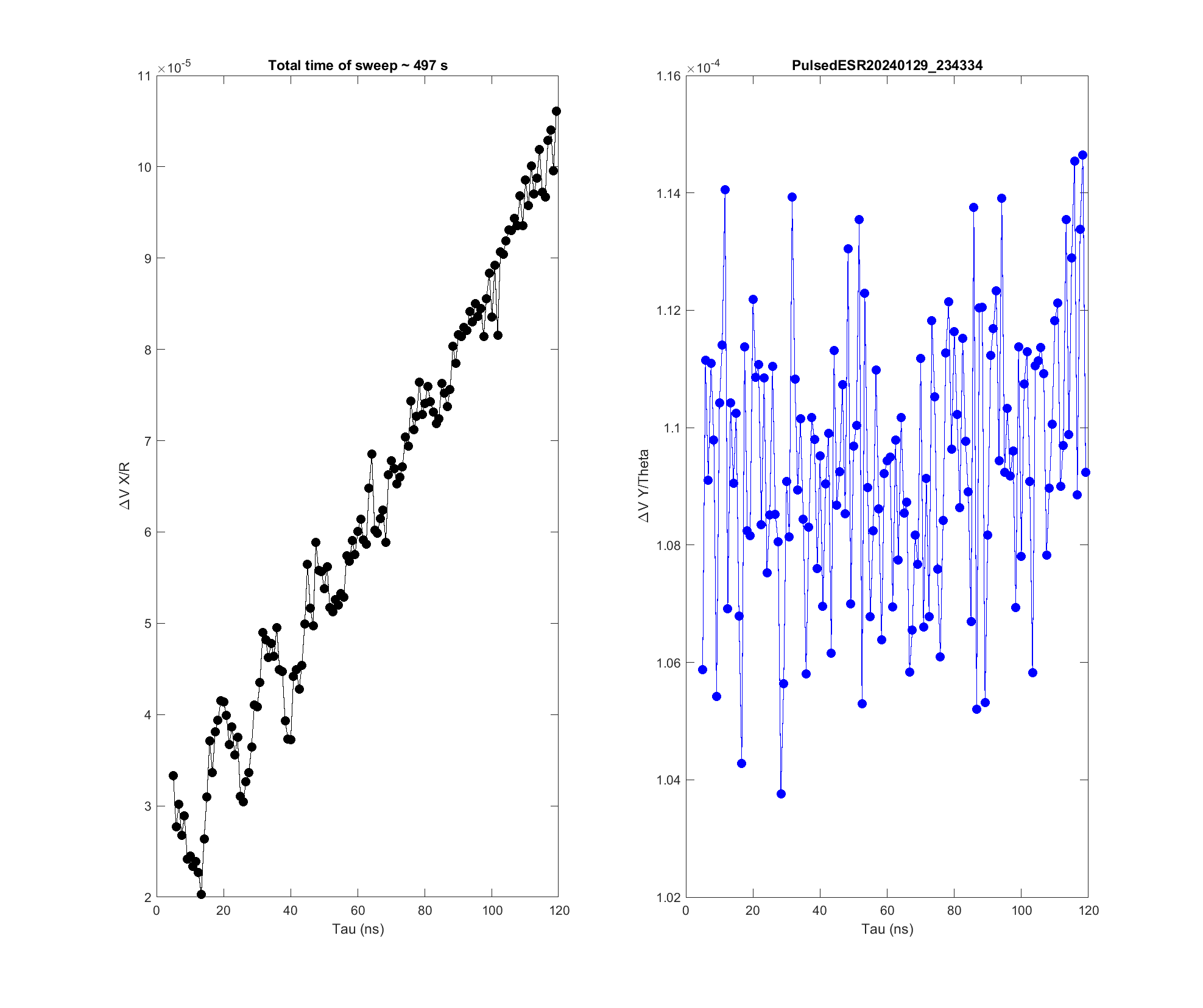

Supplement: Supplementary file 3 — Source Data [file 41467_2025_60409_MOESM3_ESM.zip › SupplementaryData1/Figure3/Fig3ab/PulsedESR20240129_234334.png]

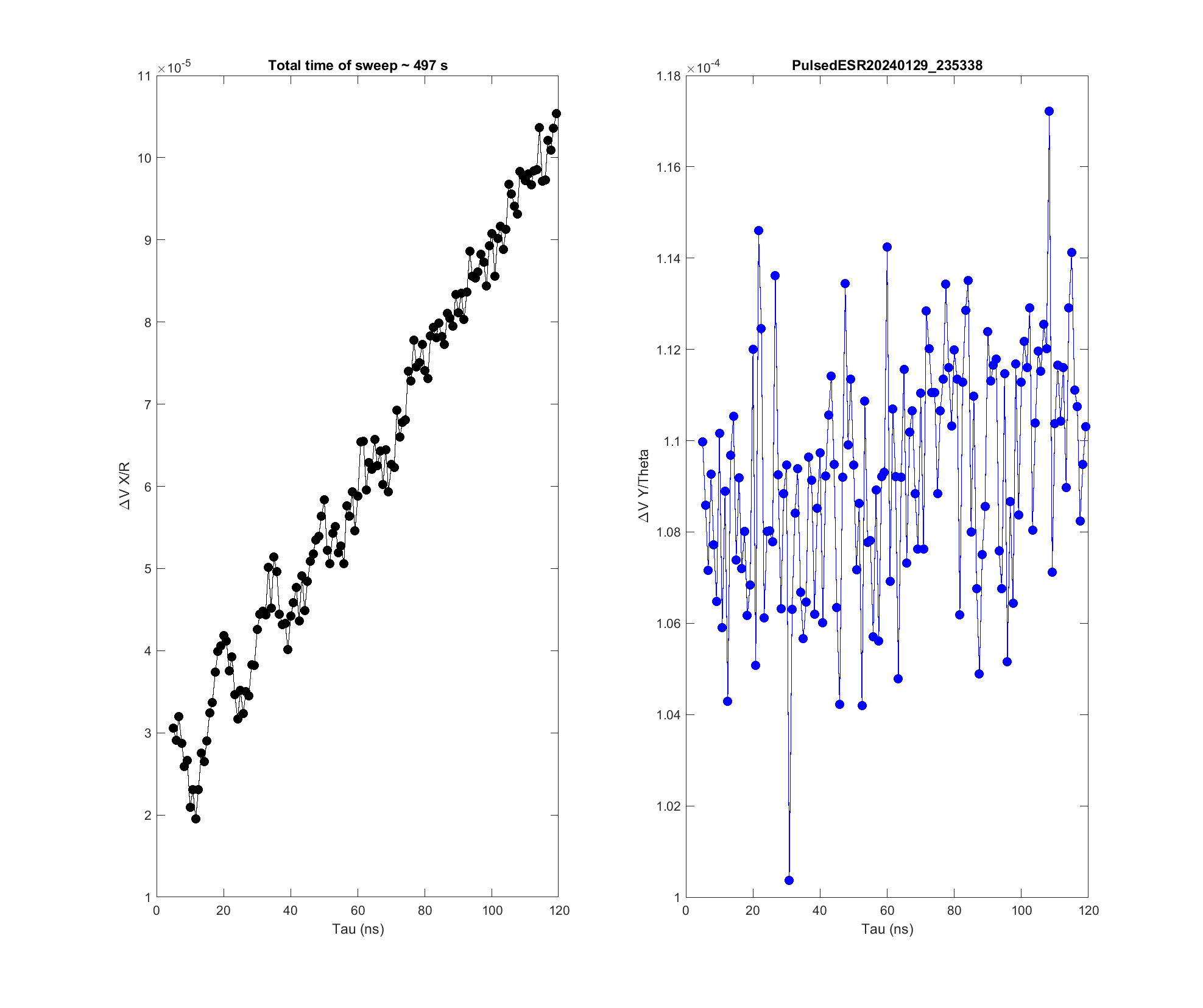

Supplement: Supplementary file 3 — Source Data [file 41467_2025_60409_MOESM3_ESM.zip › SupplementaryData1/Figure3/Fig3ab/PulsedESR20240129_235338.png]

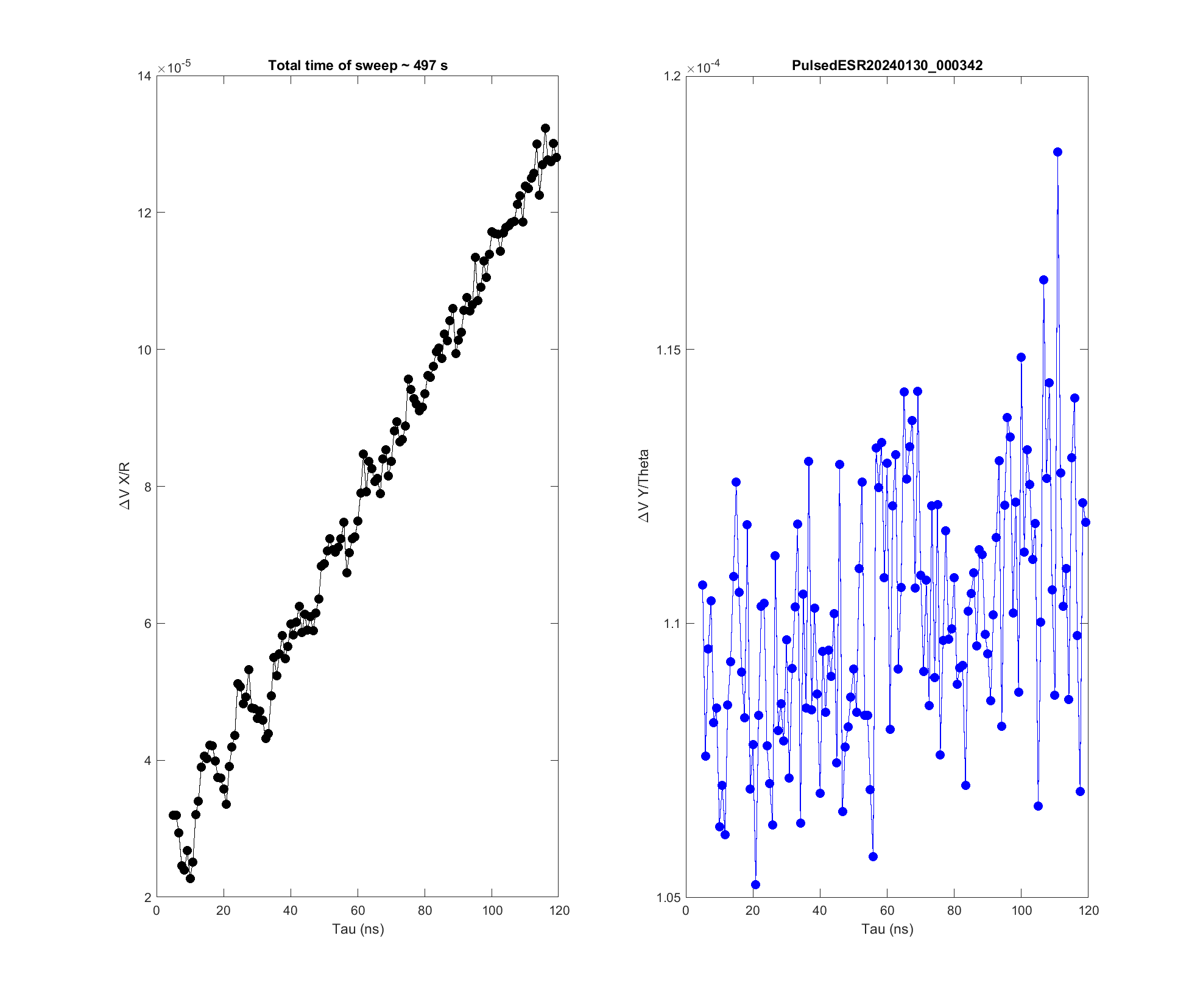

Supplement: Supplementary file 3 — Source Data [file 41467_2025_60409_MOESM3_ESM.zip › SupplementaryData1/Figure3/Fig3ab/PulsedESR20240130_000342.png]

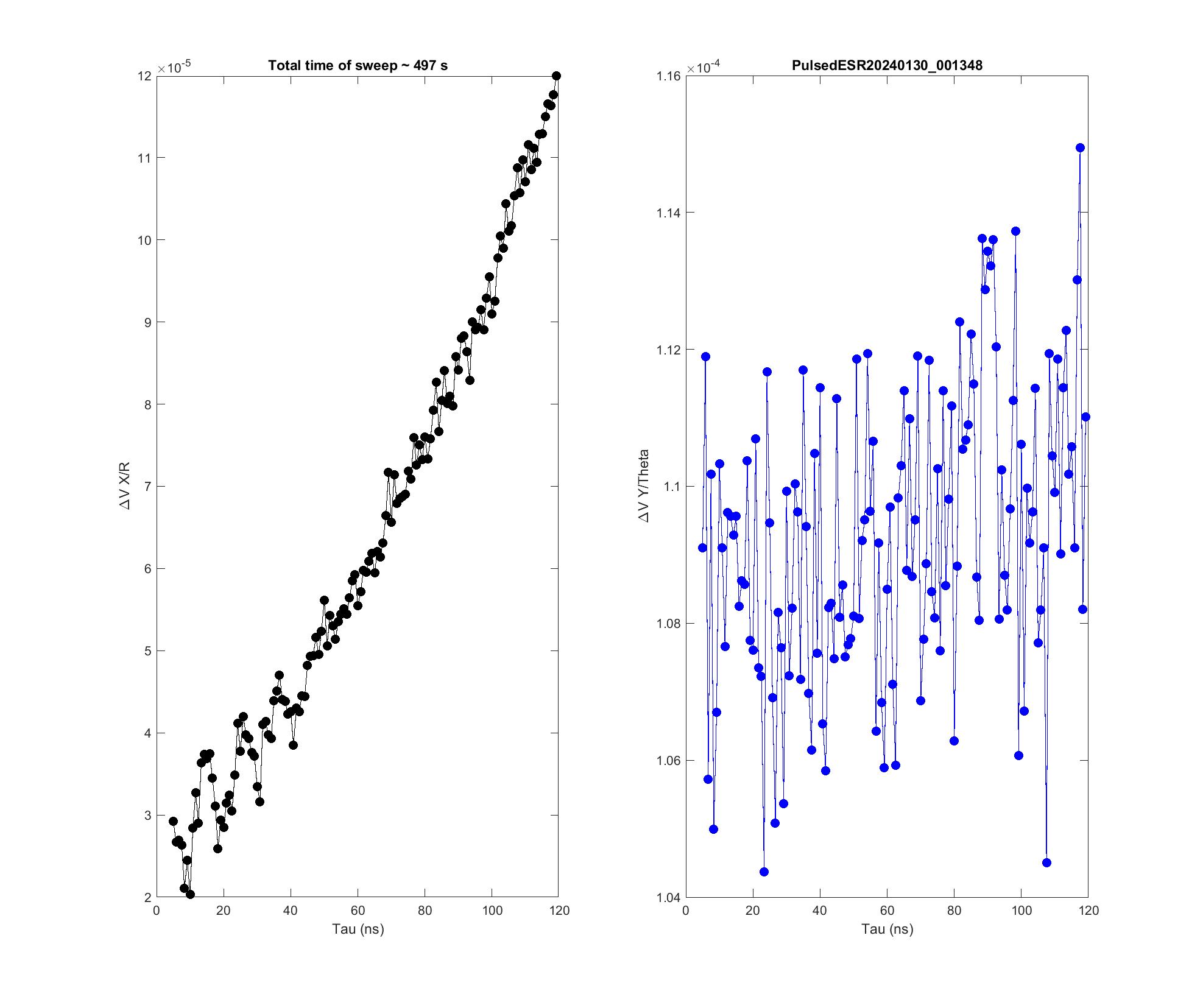

Supplement: Supplementary file 3 — Source Data [file 41467_2025_60409_MOESM3_ESM.zip › SupplementaryData1/Figure3/Fig3ab/PulsedESR20240130_001348.png]

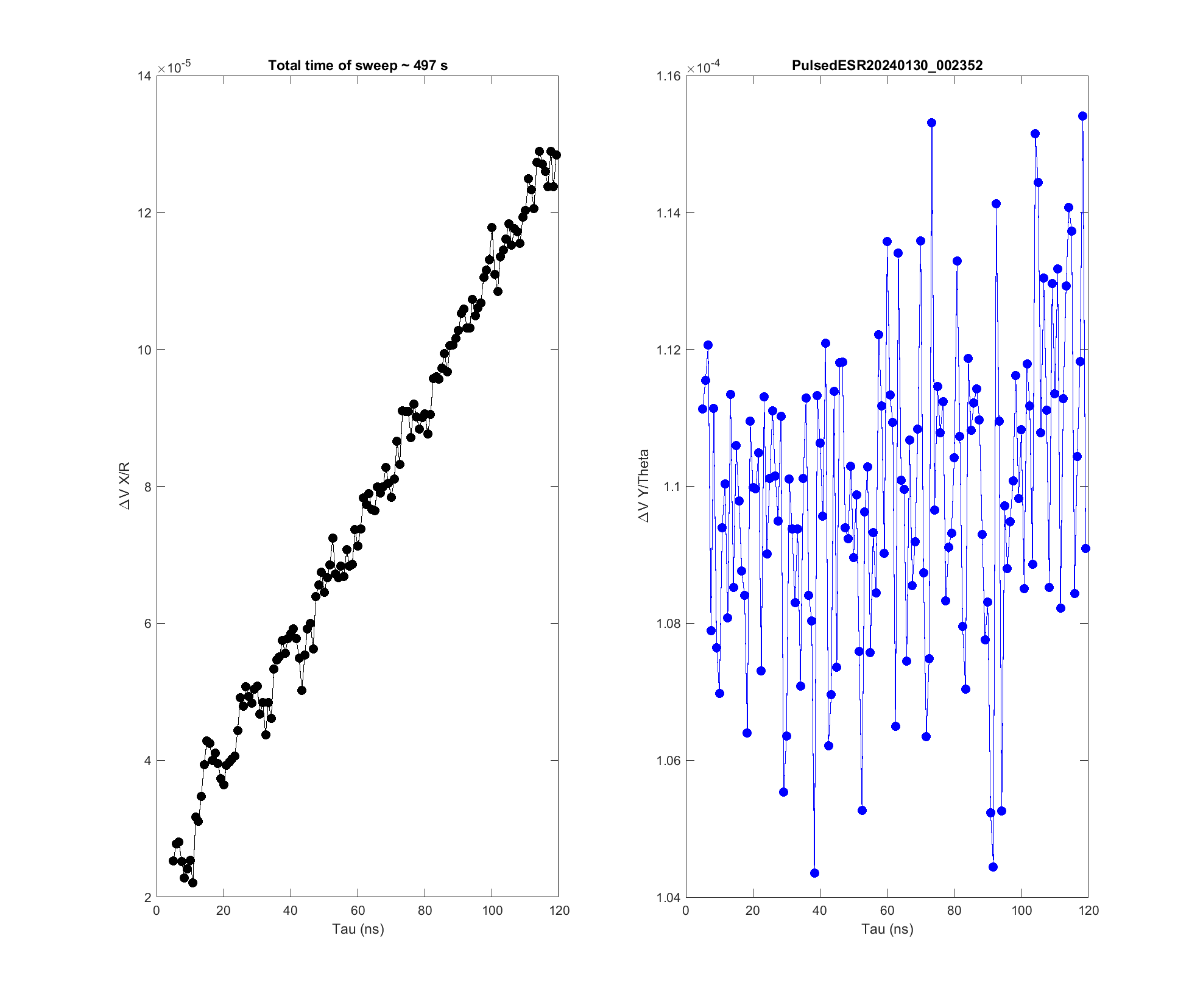

Supplement: Supplementary file 3 — Source Data [file 41467_2025_60409_MOESM3_ESM.zip › SupplementaryData1/Figure3/Fig3ab/PulsedESR20240130_002352.png]

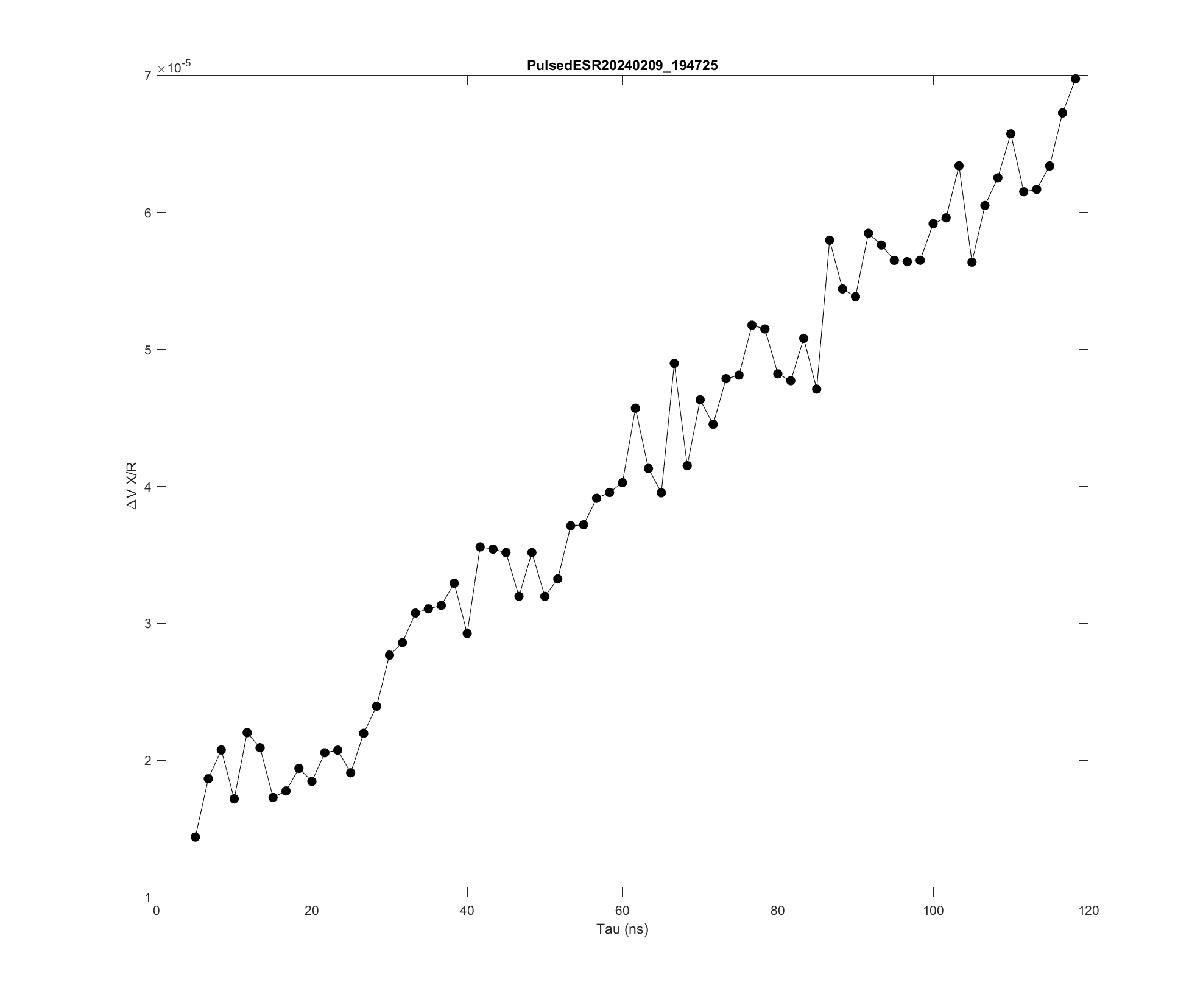

Supplement: Supplementary file 3 — Source Data [file 41467_2025_60409_MOESM3_ESM.zip › SupplementaryData1/Figure3/Fig3c/PulsedESR20240209_194725.png]

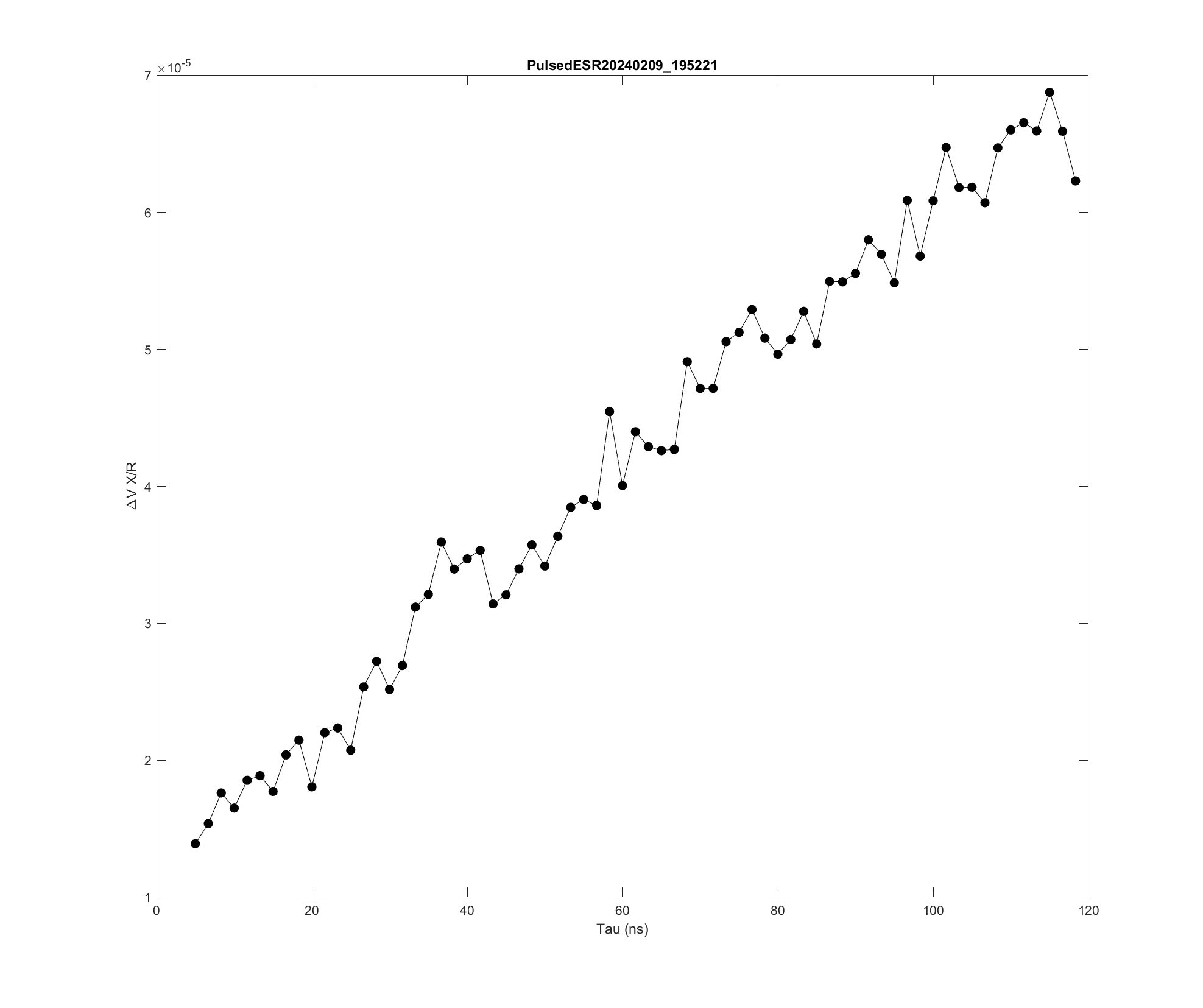

Supplement: Supplementary file 3 — Source Data [file 41467_2025_60409_MOESM3_ESM.zip › SupplementaryData1/Figure3/Fig3c/PulsedESR20240209_195221.png]

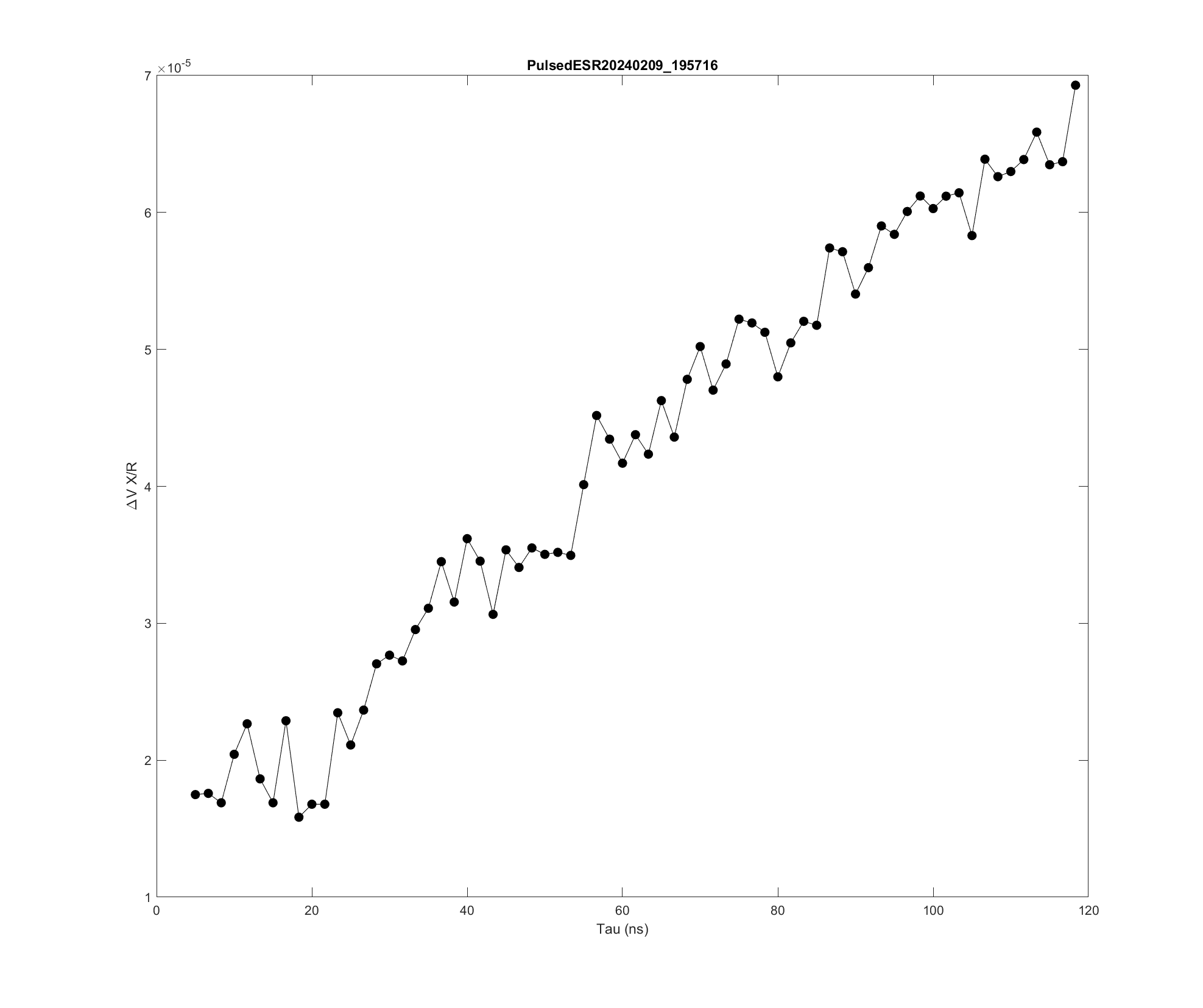

Supplement: Supplementary file 3 — Source Data [file 41467_2025_60409_MOESM3_ESM.zip › SupplementaryData1/Figure3/Fig3c/PulsedESR20240209_195716.png]

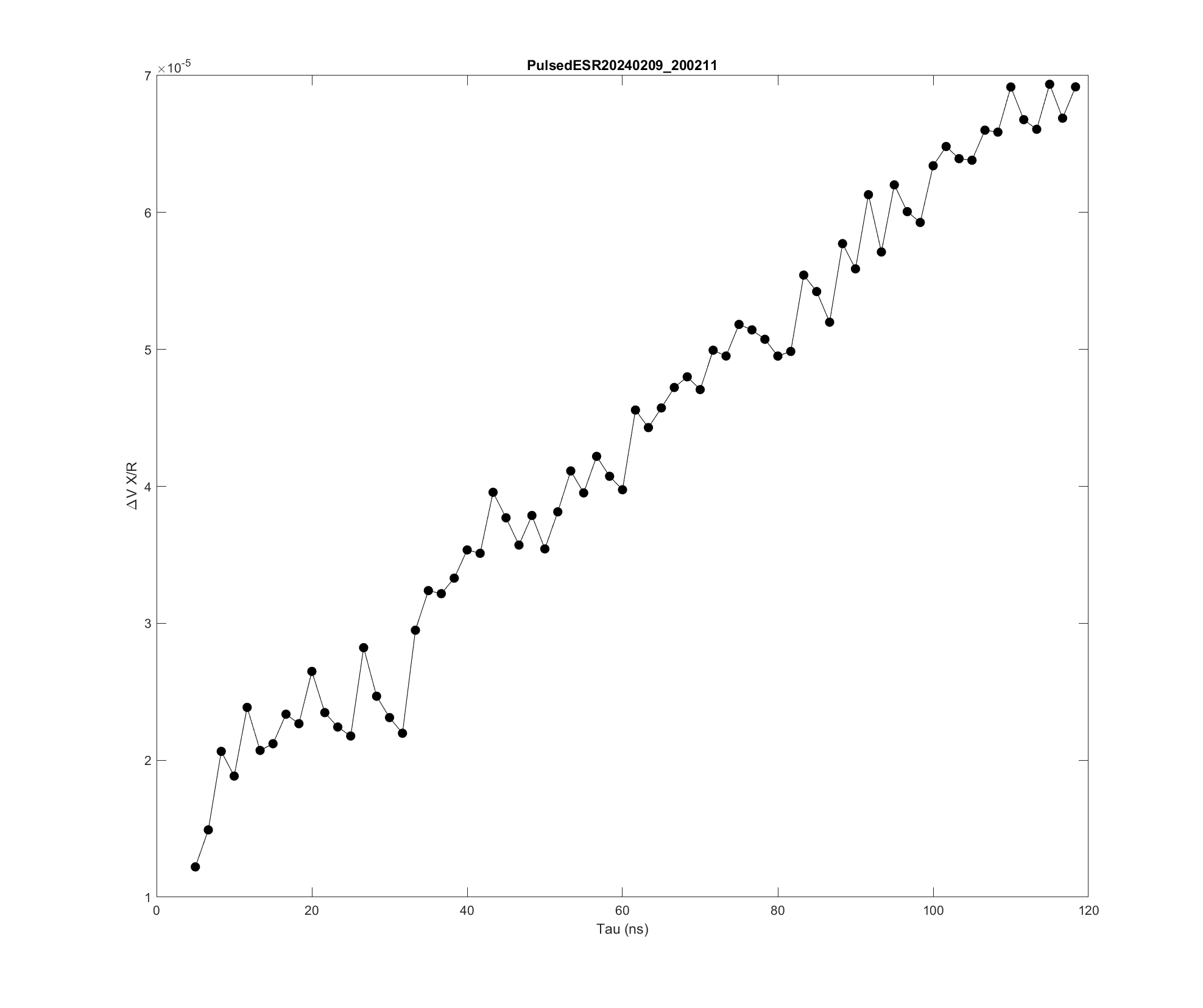

Supplement: Supplementary file 3 — Source Data [file 41467_2025_60409_MOESM3_ESM.zip › SupplementaryData1/Figure3/Fig3c/PulsedESR20240209_200211.png]

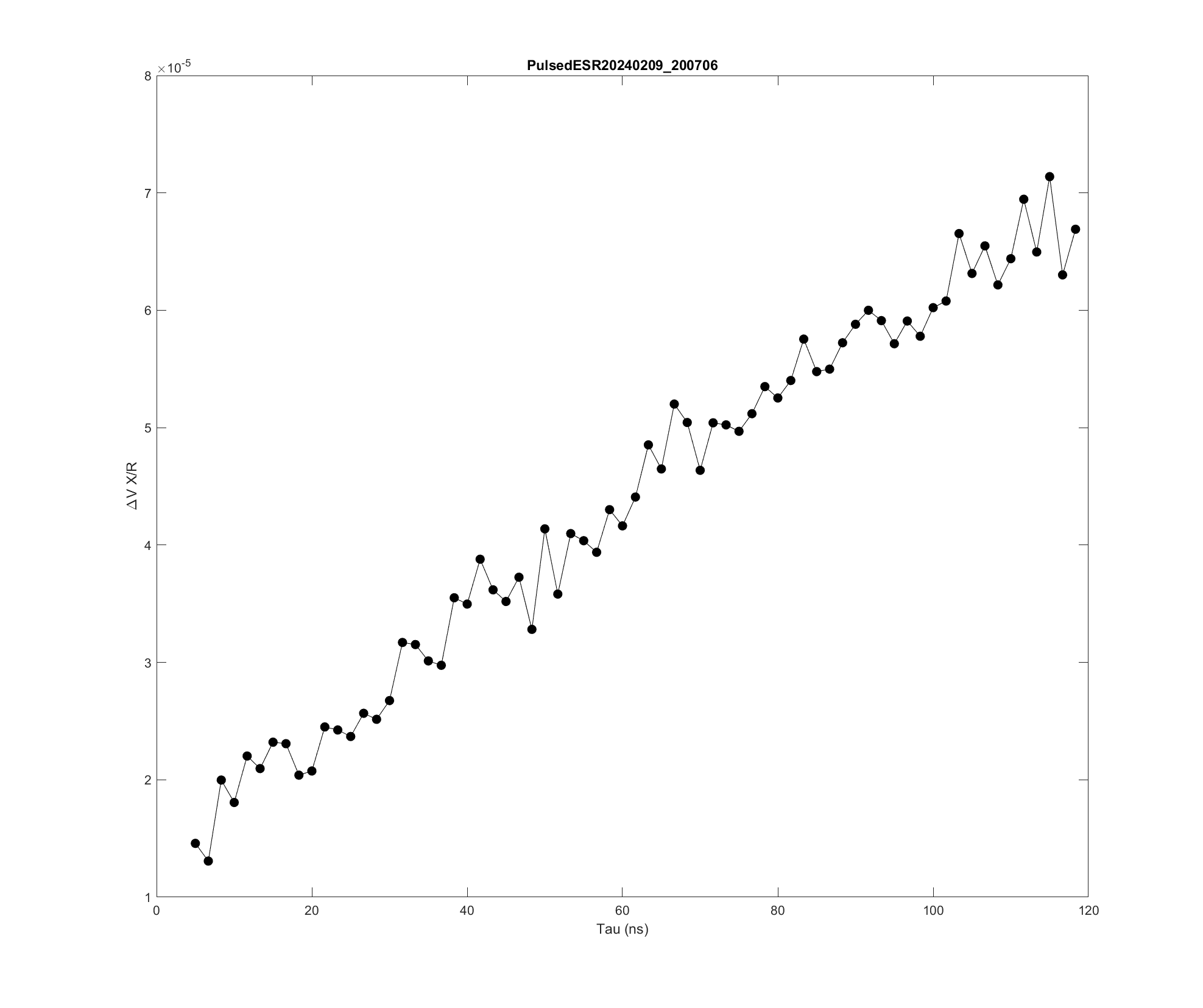

Supplement: Supplementary file 3 — Source Data [file 41467_2025_60409_MOESM3_ESM.zip › SupplementaryData1/Figure3/Fig3c/PulsedESR20240209_200706.png]

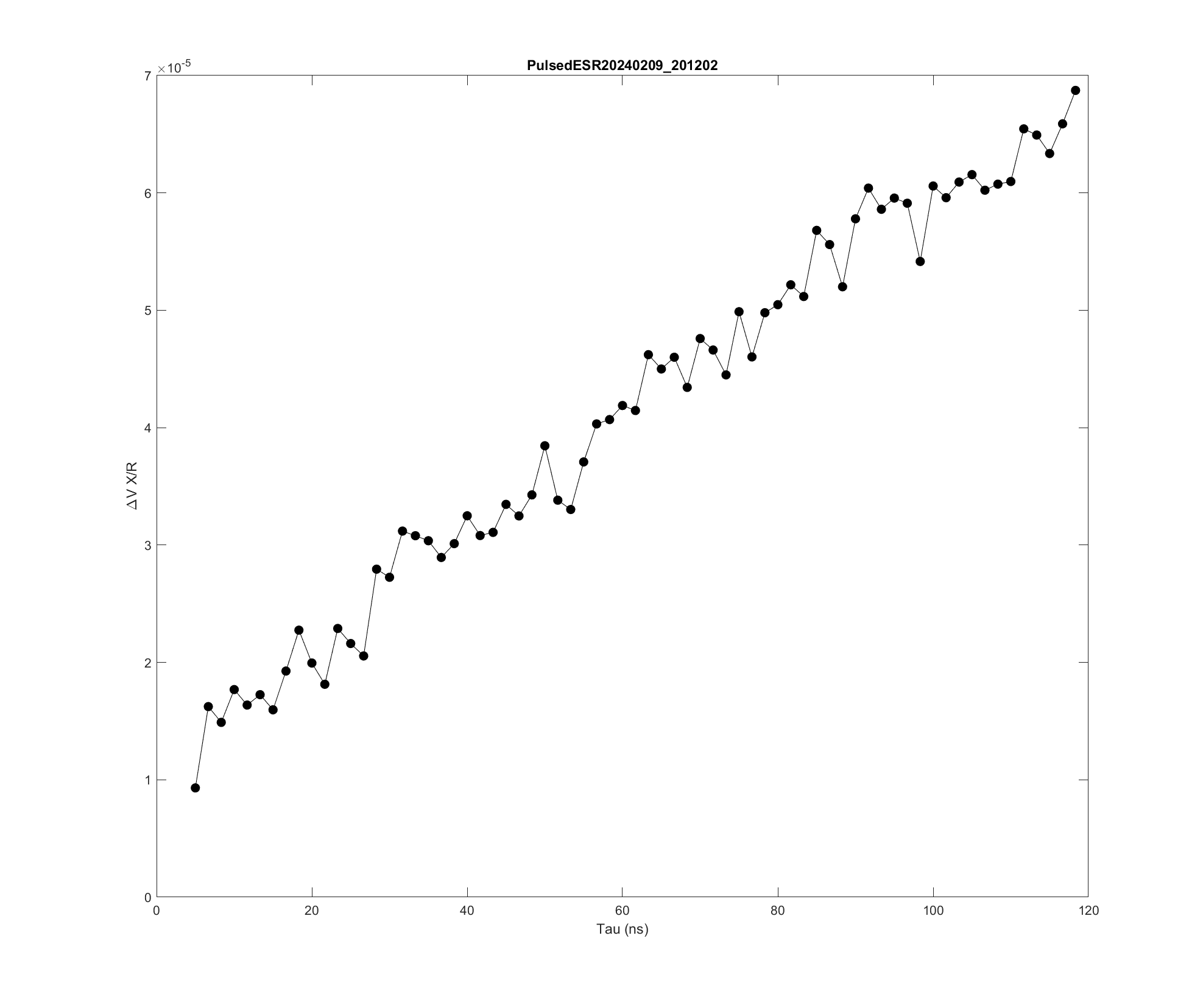

Supplement: Supplementary file 3 — Source Data [file 41467_2025_60409_MOESM3_ESM.zip › SupplementaryData1/Figure3/Fig3c/PulsedESR20240209_201202.png]

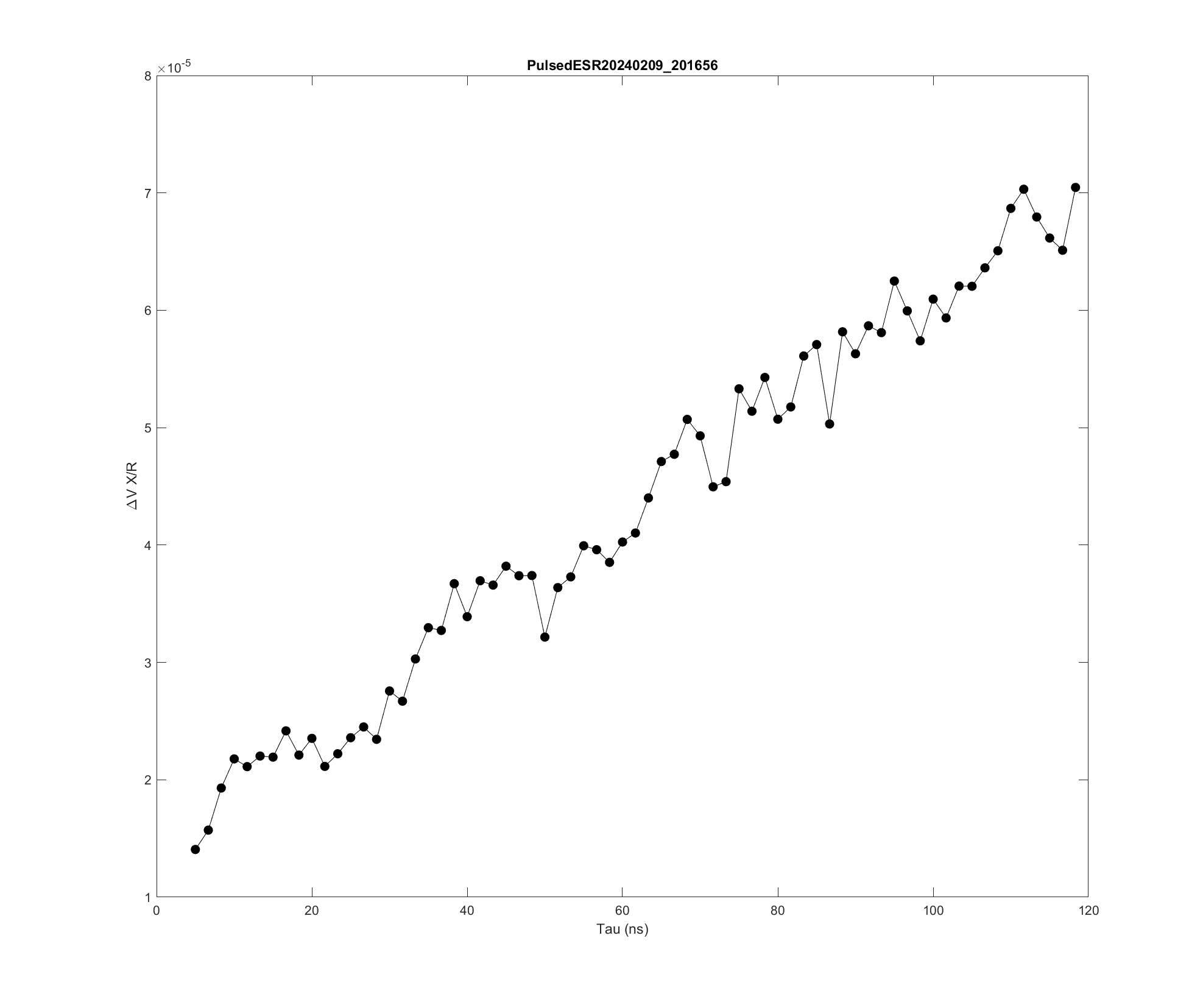

Supplement: Supplementary file 3 — Source Data [file 41467_2025_60409_MOESM3_ESM.zip › SupplementaryData1/Figure3/Fig3c/PulsedESR20240209_201656.png]

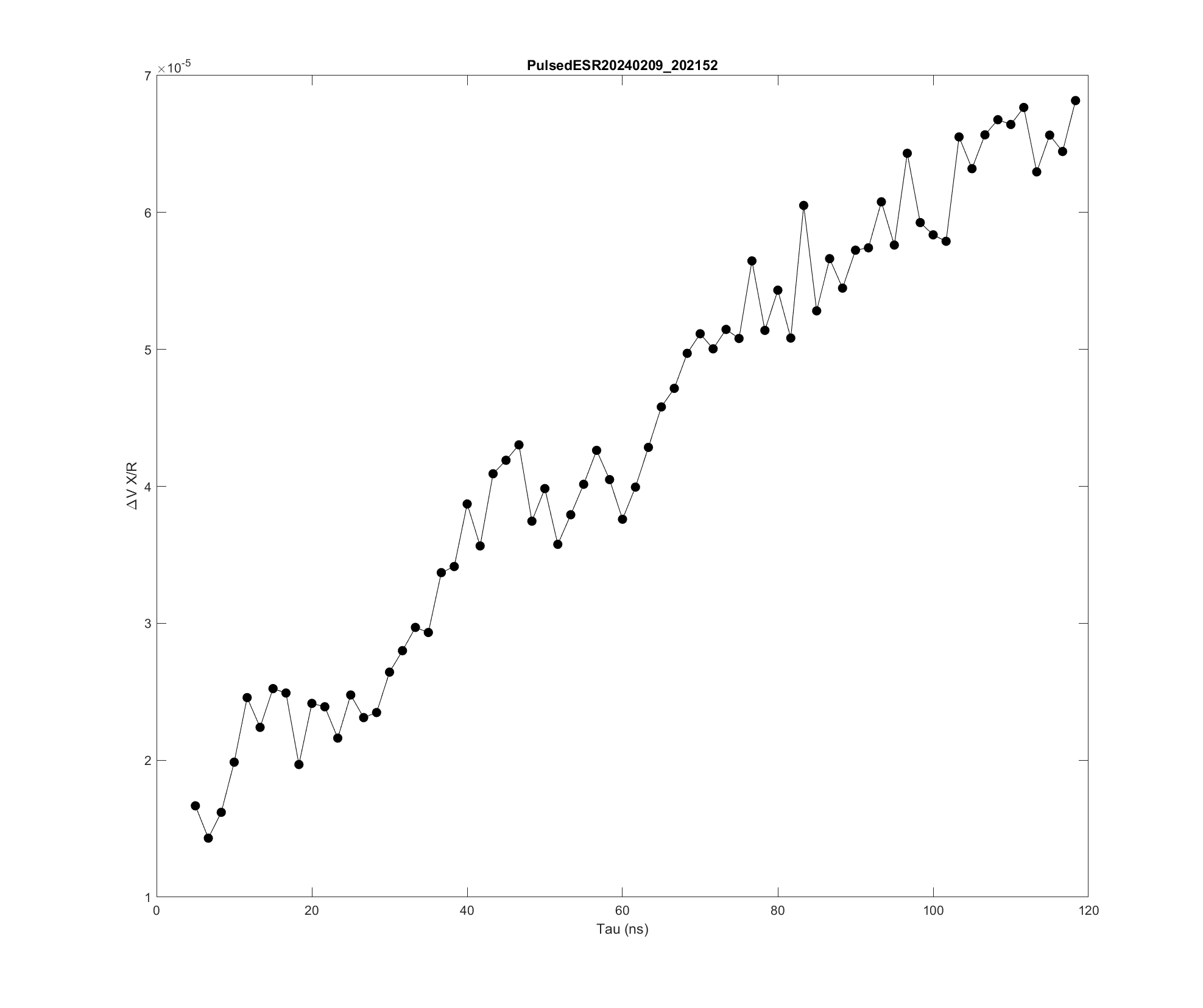

Supplement: Supplementary file 3 — Source Data [file 41467_2025_60409_MOESM3_ESM.zip › SupplementaryData1/Figure3/Fig3c/PulsedESR20240209_202152.png]

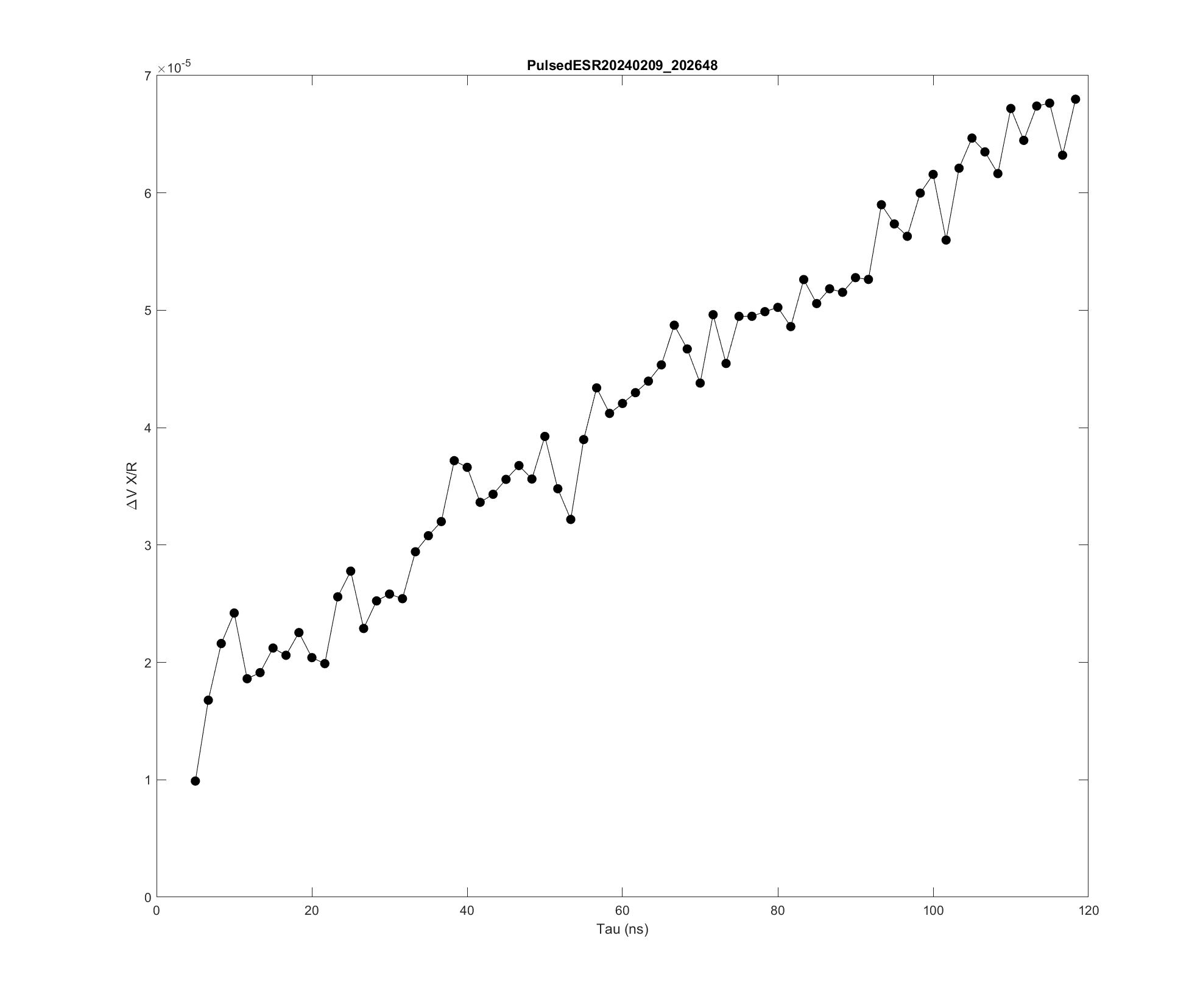

Supplement: Supplementary file 3 — Source Data [file 41467_2025_60409_MOESM3_ESM.zip › SupplementaryData1/Figure3/Fig3c/PulsedESR20240209_202648.png]

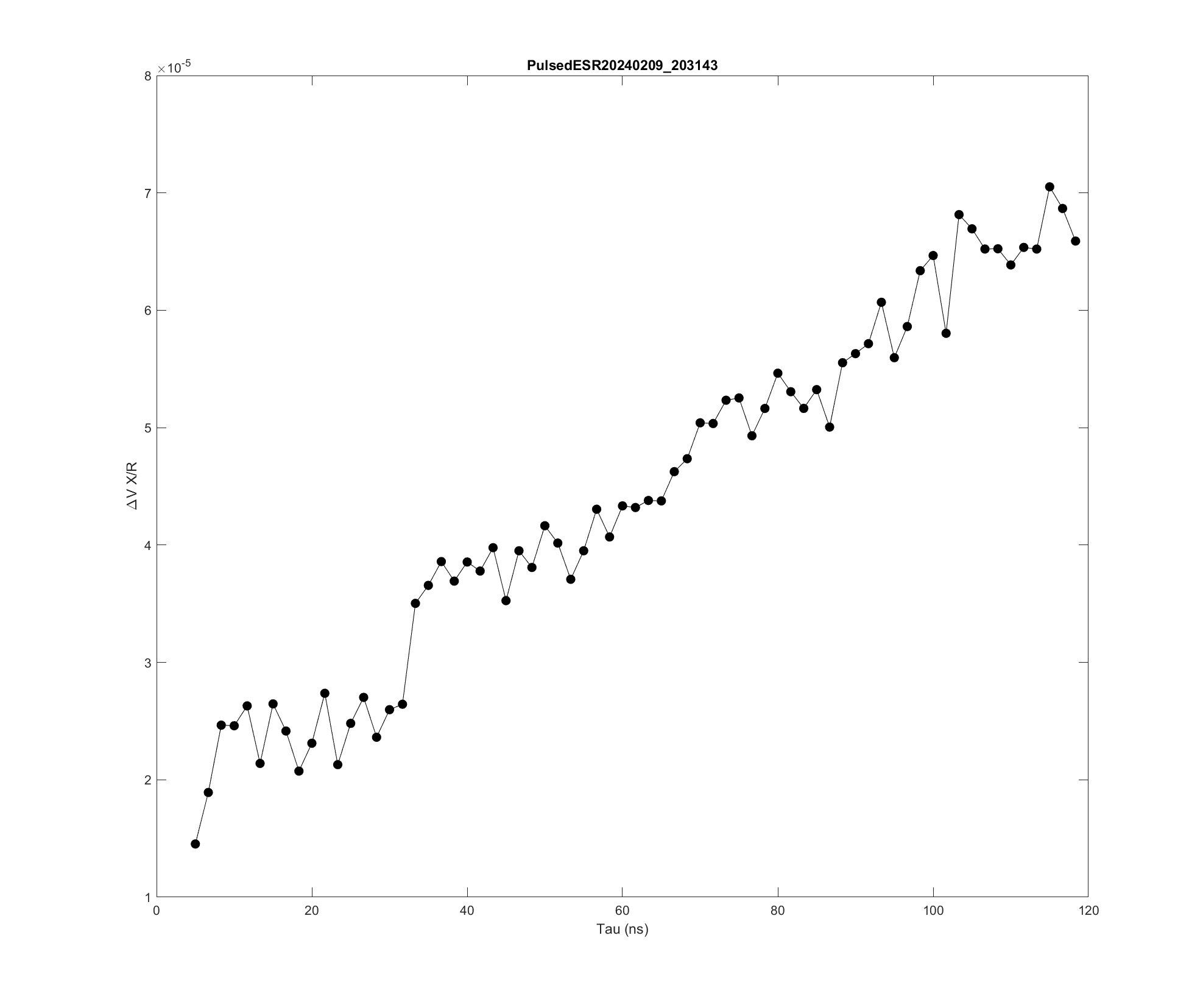

Supplement: Supplementary file 3 — Source Data [file 41467_2025_60409_MOESM3_ESM.zip › SupplementaryData1/Figure3/Fig3c/PulsedESR20240209_203143.png]

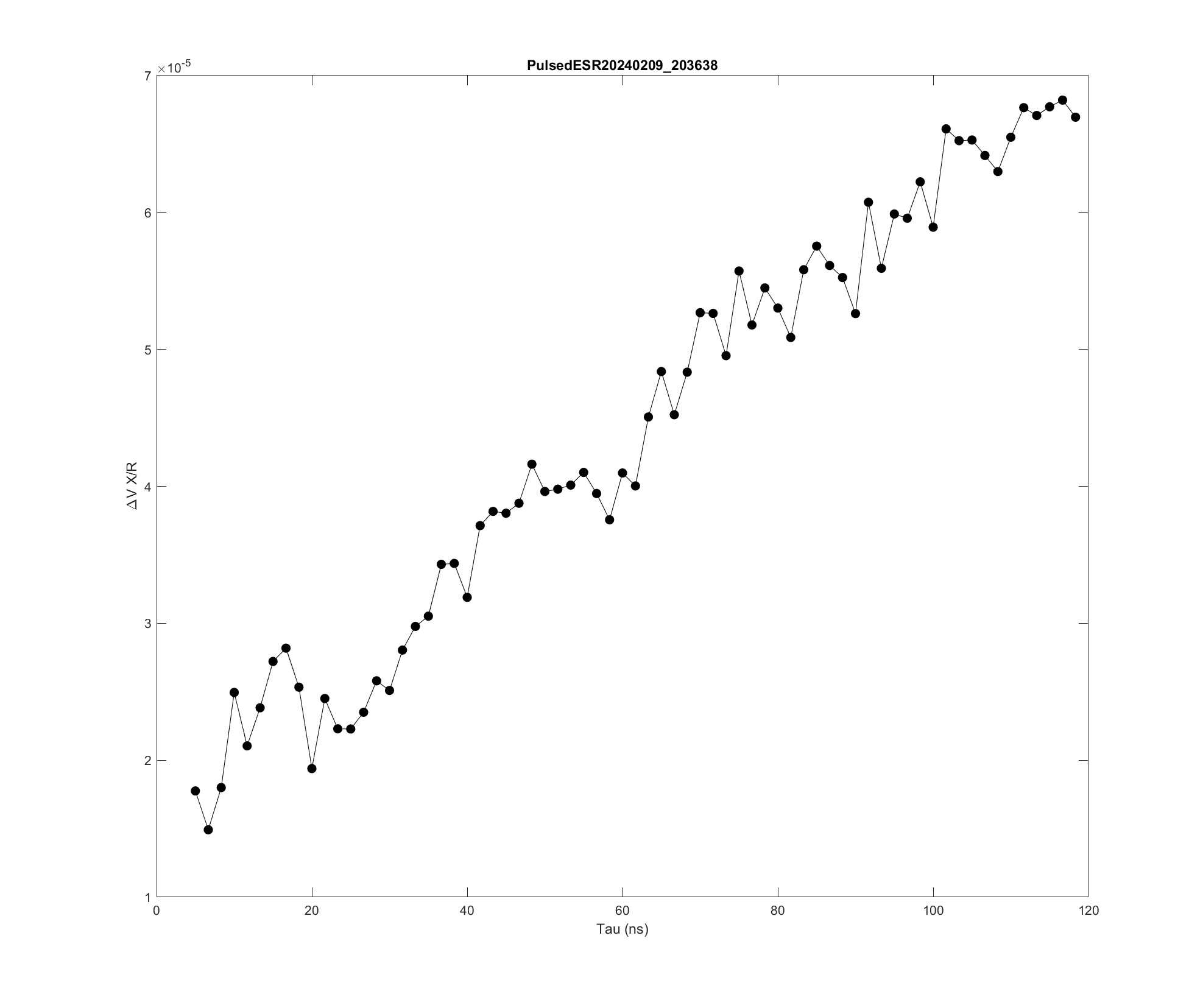

Supplement: Supplementary file 3 — Source Data [file 41467_2025_60409_MOESM3_ESM.zip › SupplementaryData1/Figure3/Fig3c/PulsedESR20240209_203638.png]

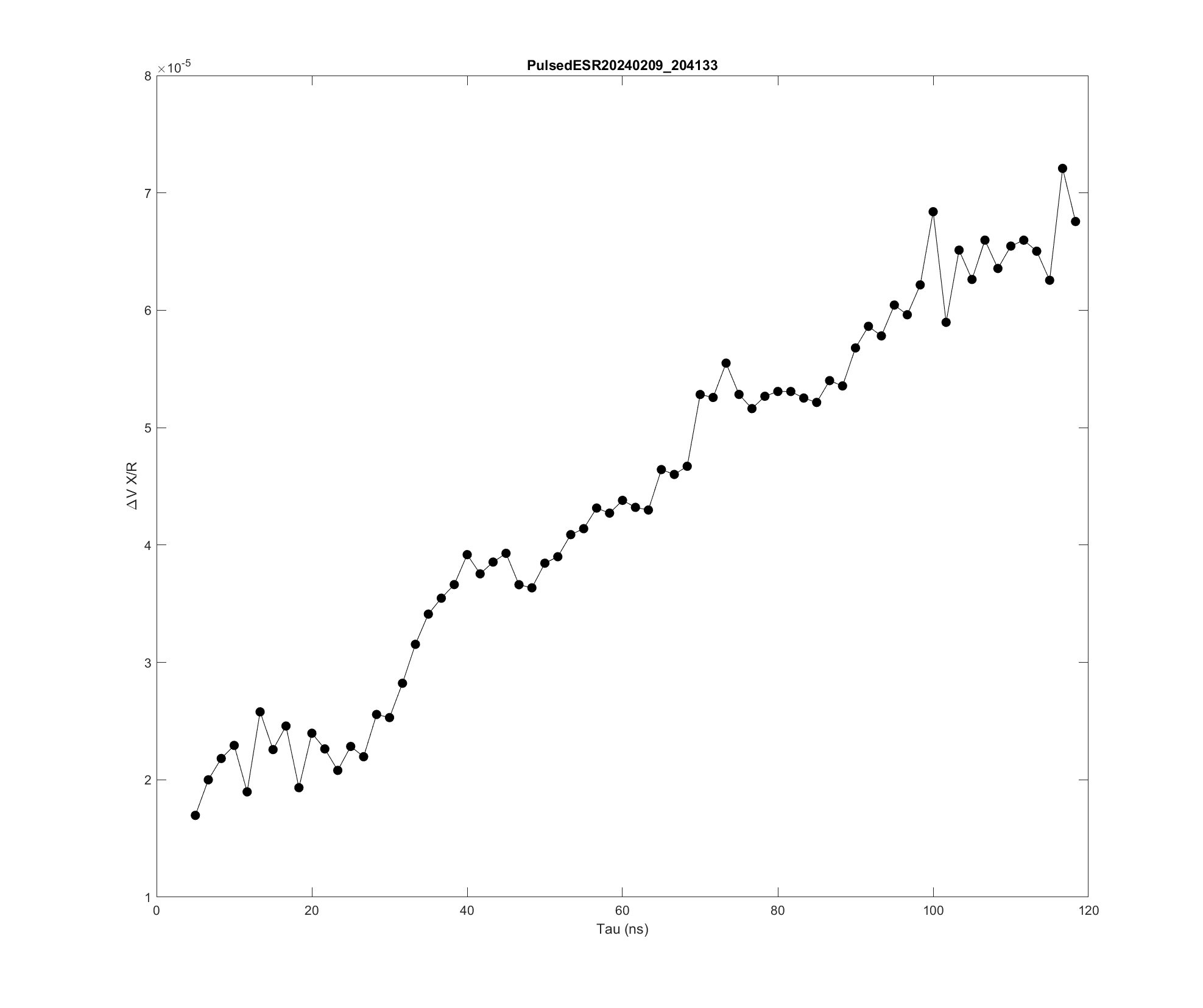

Supplement: Supplementary file 3 — Source Data [file 41467_2025_60409_MOESM3_ESM.zip › SupplementaryData1/Figure3/Fig3c/PulsedESR20240209_204133.png]

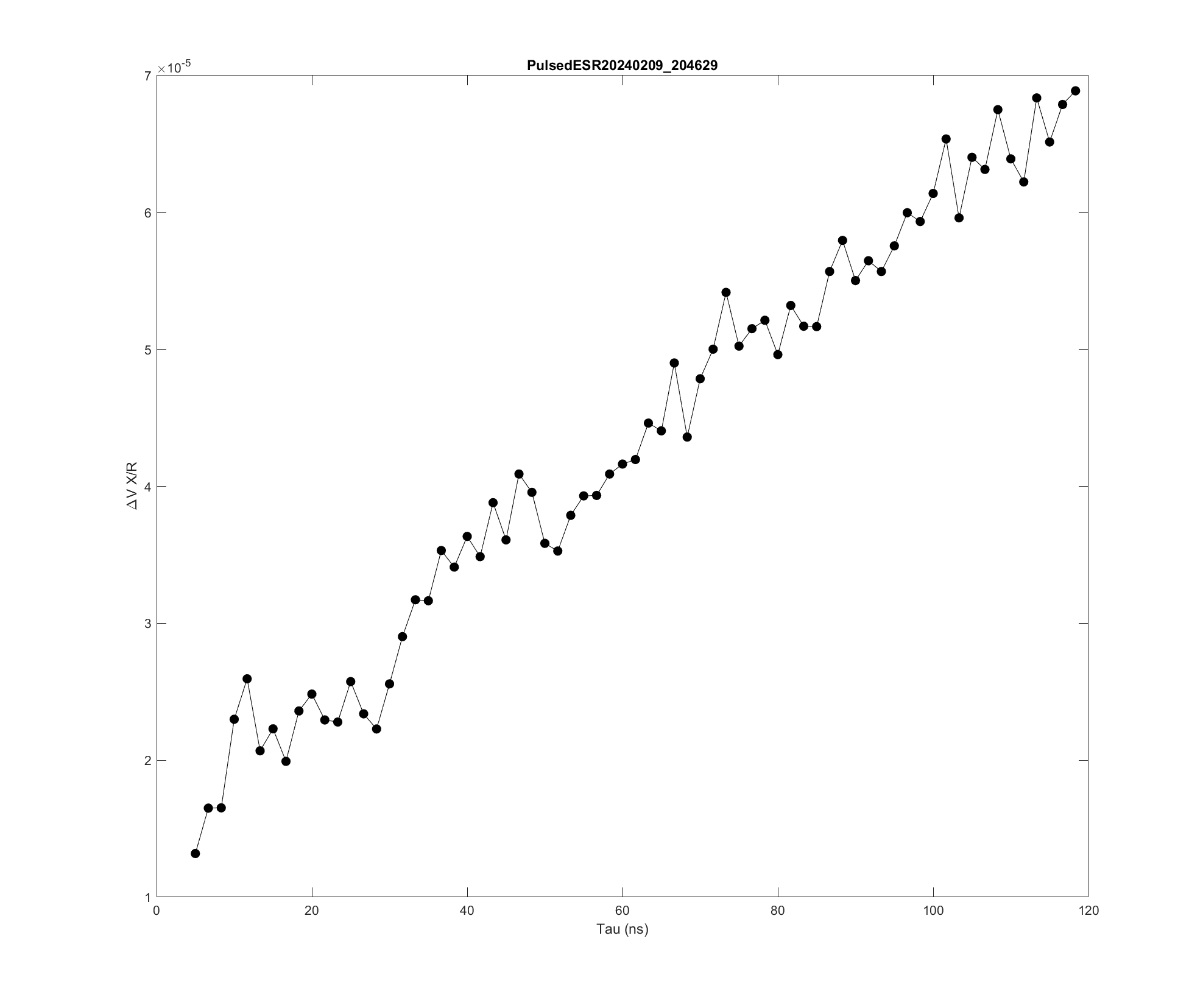

Supplement: Supplementary file 3 — Source Data [file 41467_2025_60409_MOESM3_ESM.zip › SupplementaryData1/Figure3/Fig3c/PulsedESR20240209_204629.png]

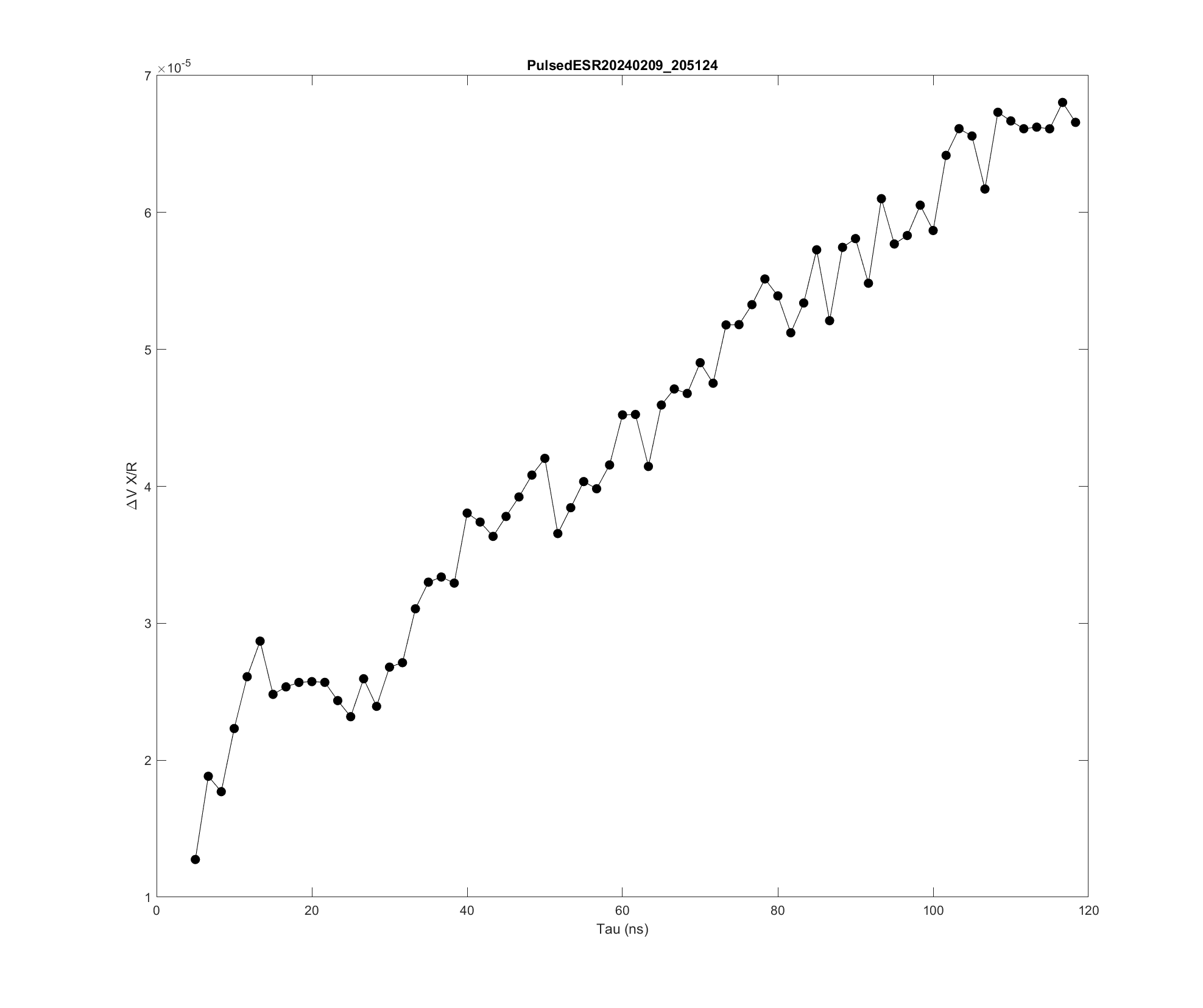

Supplement: Supplementary file 3 — Source Data [file 41467_2025_60409_MOESM3_ESM.zip › SupplementaryData1/Figure3/Fig3c/PulsedESR20240209_205124.png]

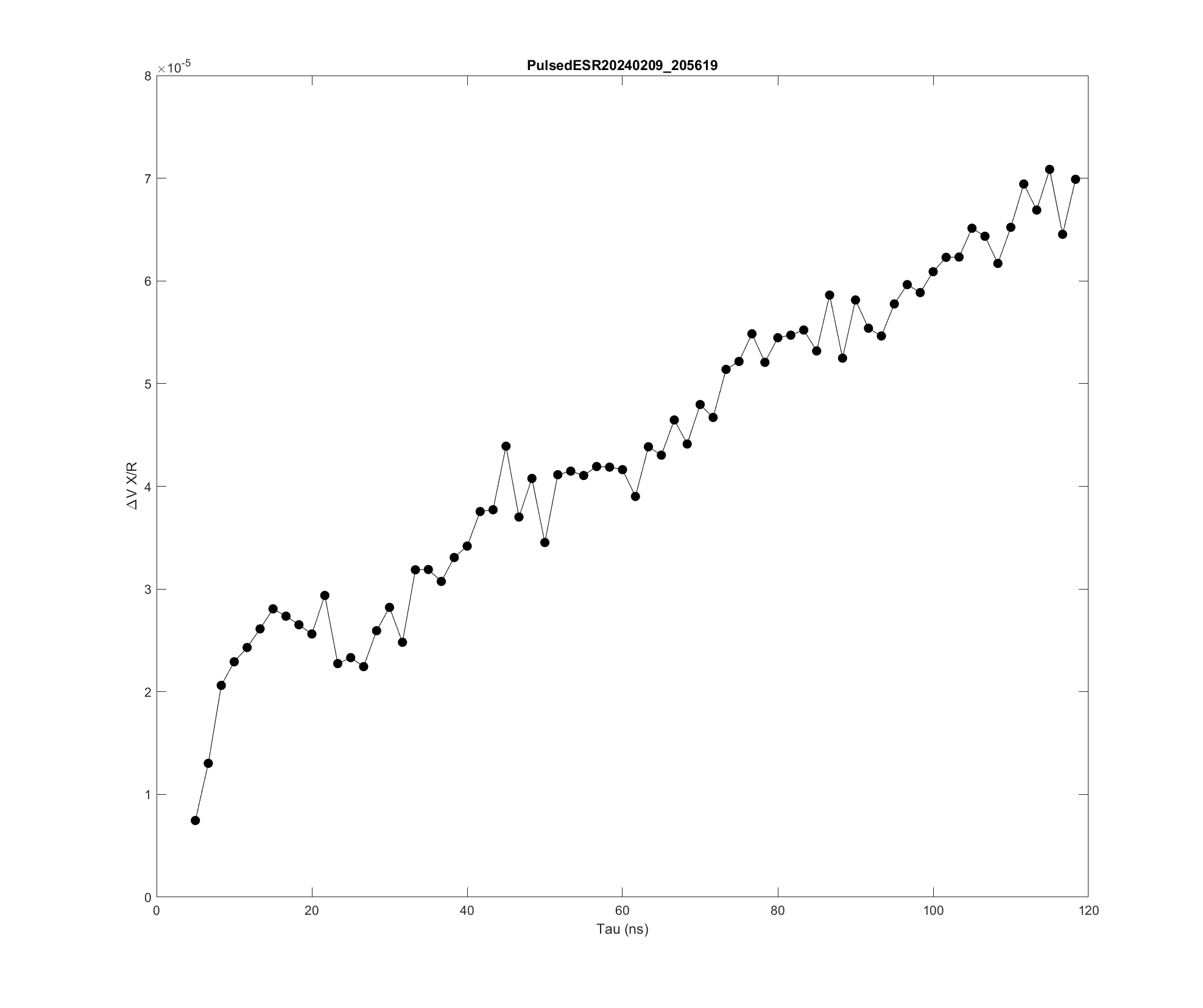

Supplement: Supplementary file 3 — Source Data [file 41467_2025_60409_MOESM3_ESM.zip › SupplementaryData1/Figure3/Fig3c/PulsedESR20240209_205619.png]

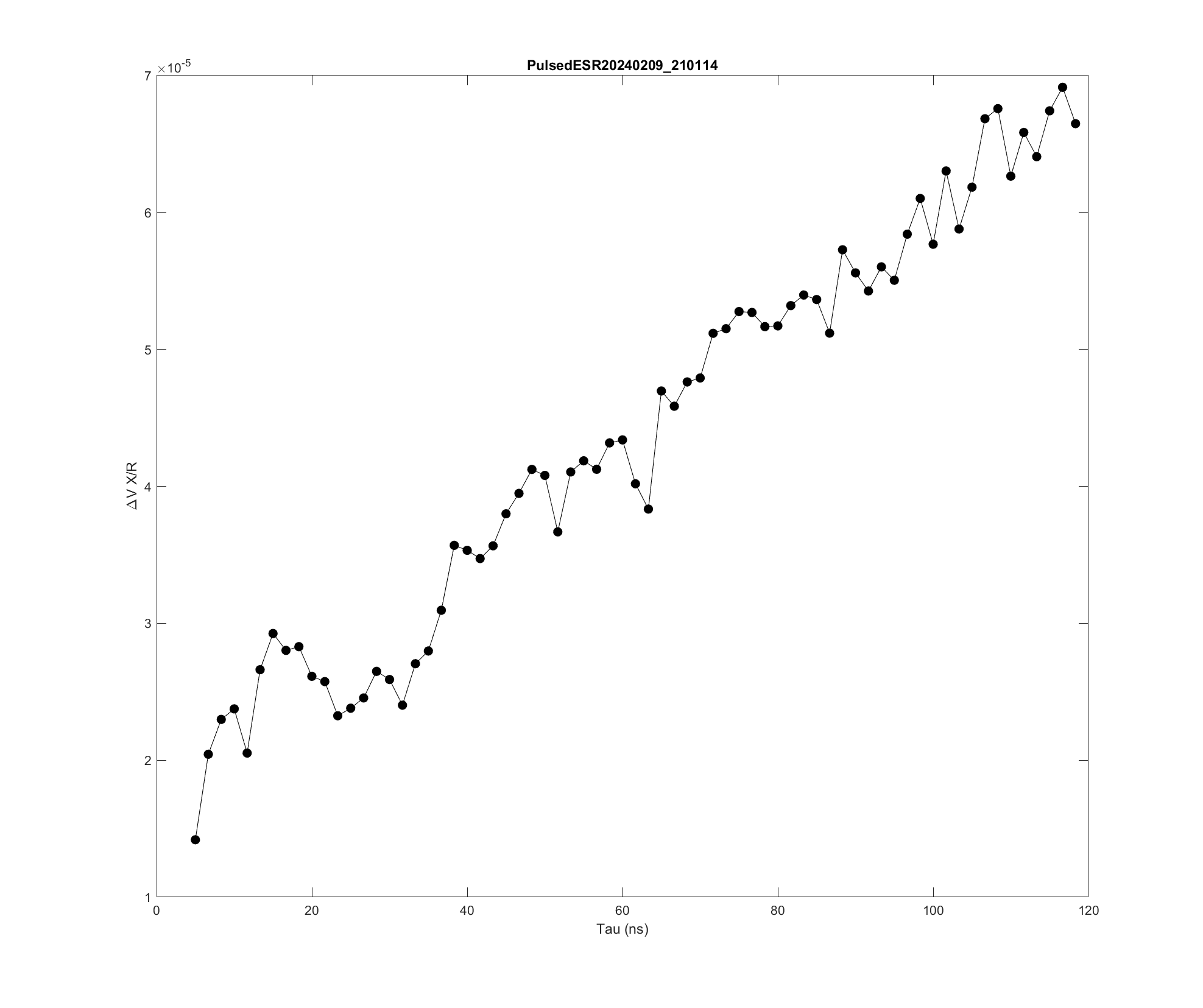

Supplement: Supplementary file 3 — Source Data [file 41467_2025_60409_MOESM3_ESM.zip › SupplementaryData1/Figure3/Fig3c/PulsedESR20240209_210114.png]

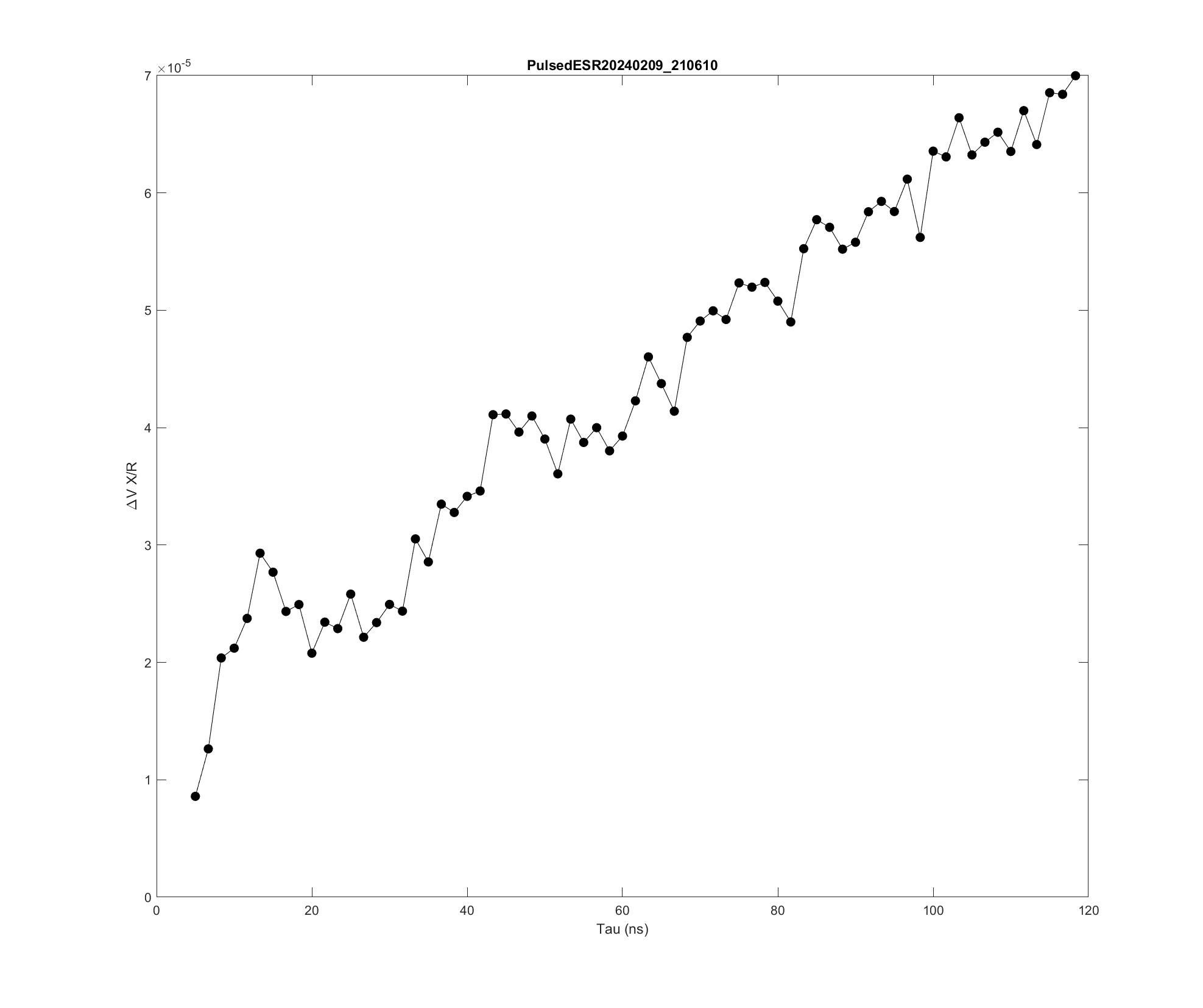

Supplement: Supplementary file 3 — Source Data [file 41467_2025_60409_MOESM3_ESM.zip › SupplementaryData1/Figure3/Fig3c/PulsedESR20240209_210610.png]

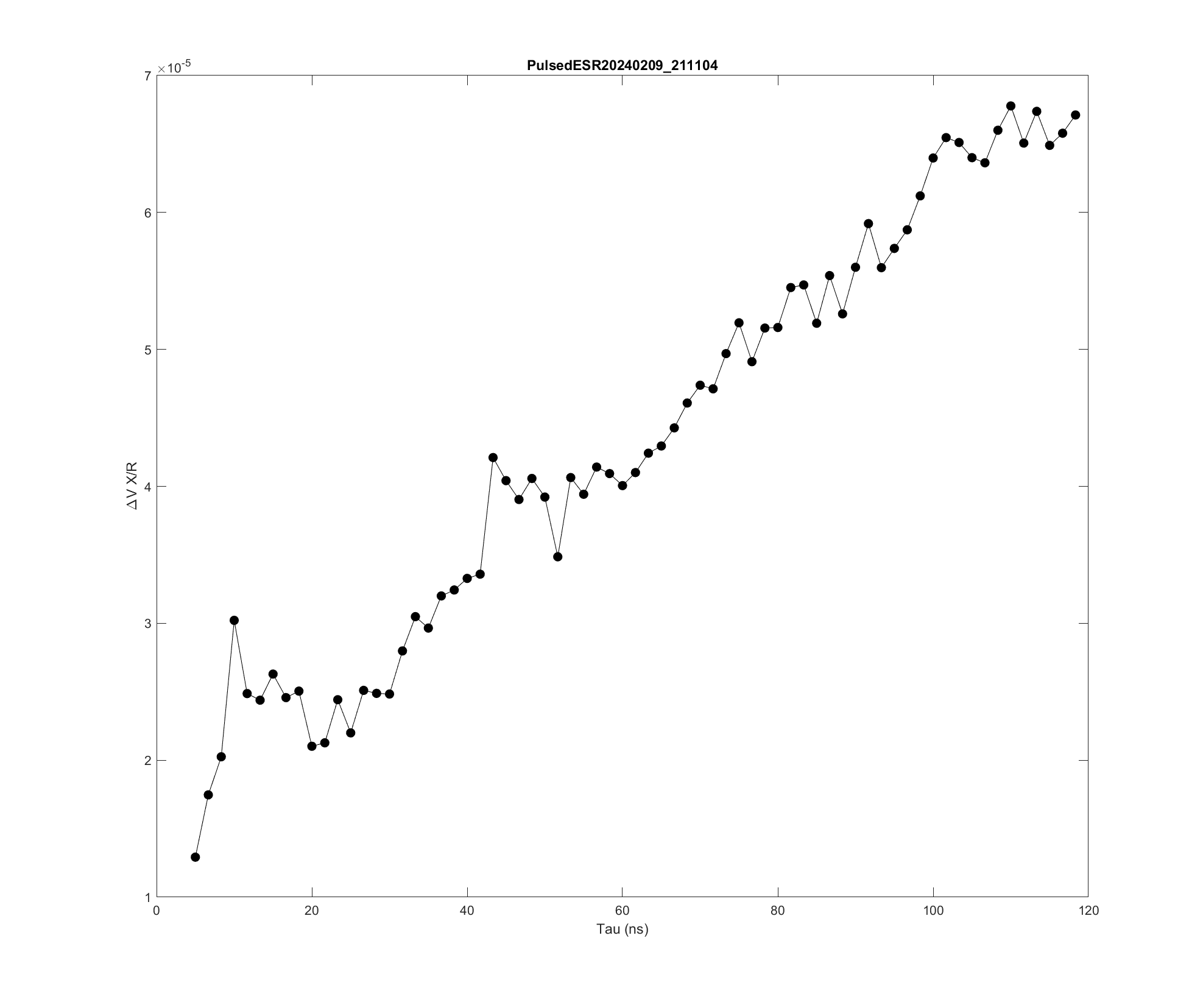

Supplement: Supplementary file 3 — Source Data [file 41467_2025_60409_MOESM3_ESM.zip › SupplementaryData1/Figure3/Fig3c/PulsedESR20240209_211104.png]

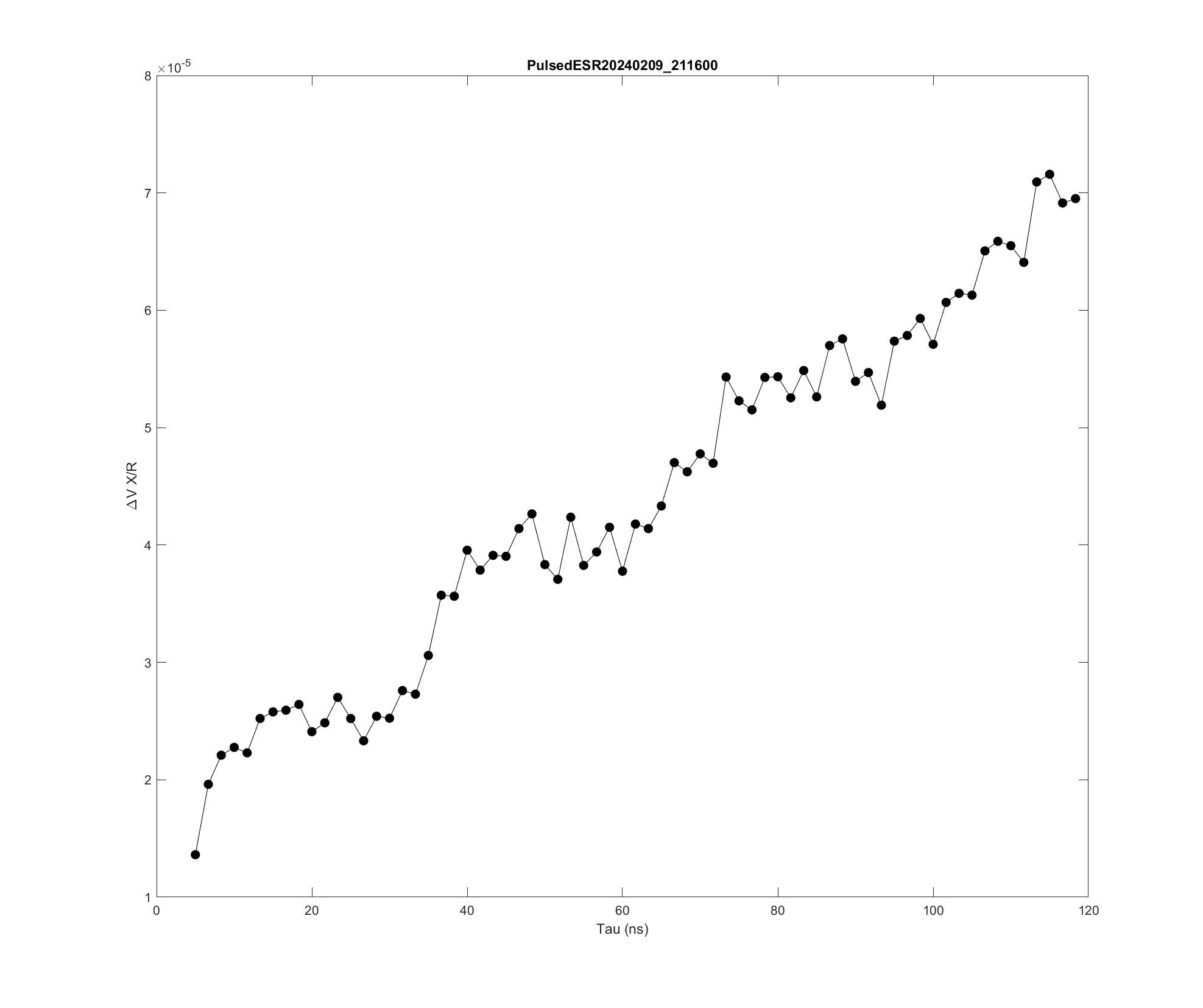

Supplement: Supplementary file 3 — Source Data [file 41467_2025_60409_MOESM3_ESM.zip › SupplementaryData1/Figure3/Fig3c/PulsedESR20240209_211600.png]

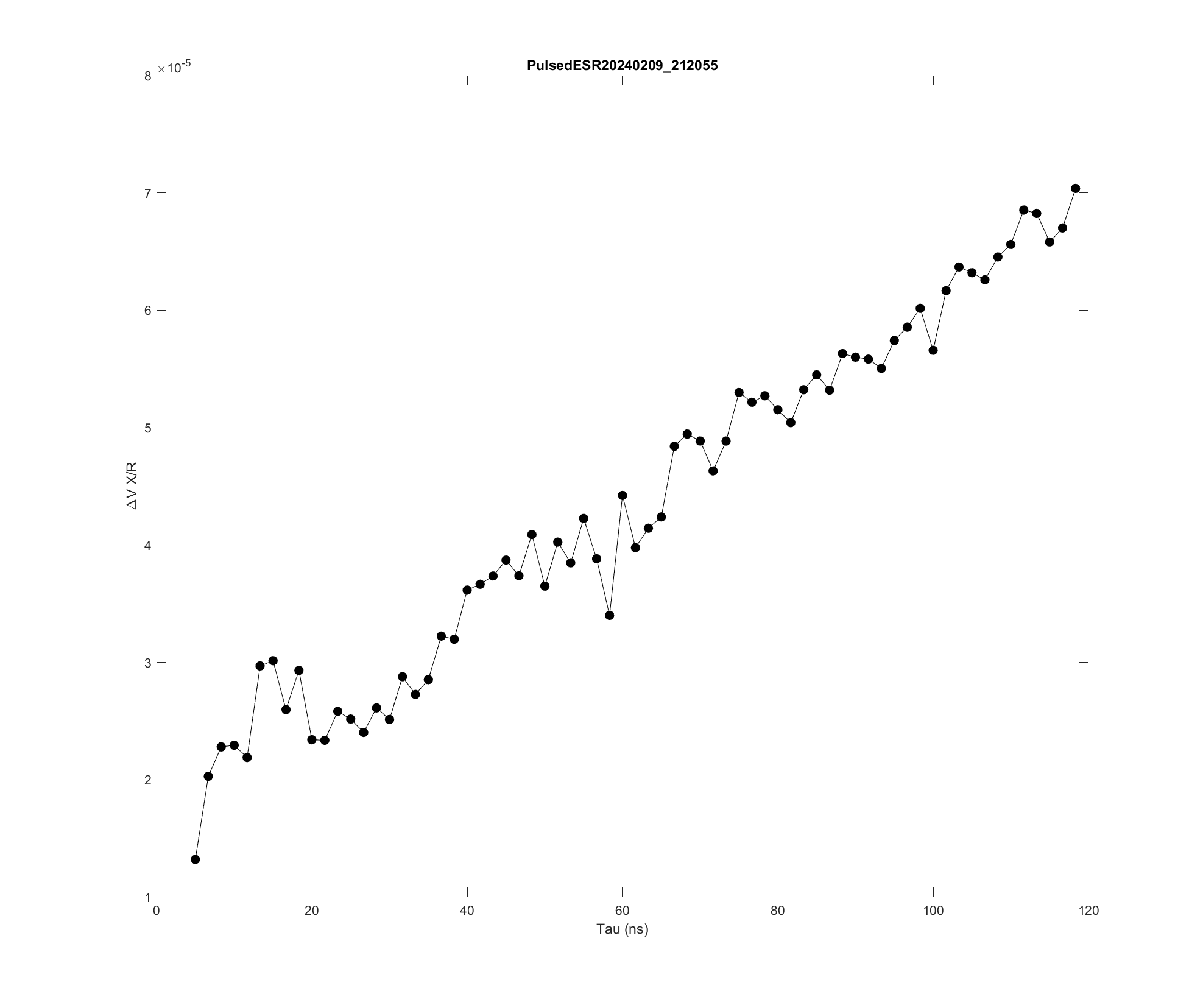

Supplement: Supplementary file 3 — Source Data [file 41467_2025_60409_MOESM3_ESM.zip › SupplementaryData1/Figure3/Fig3c/PulsedESR20240209_212055.png]

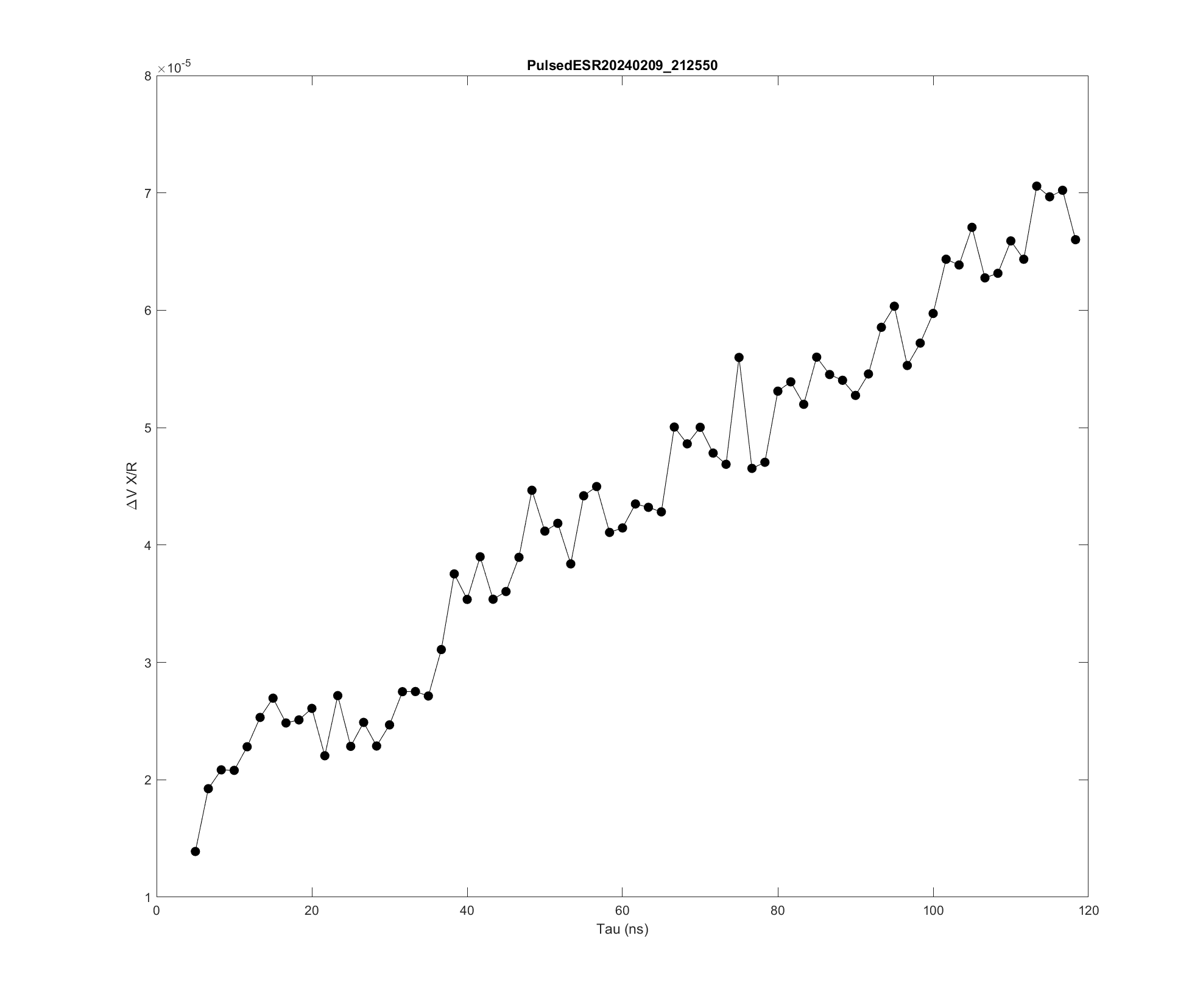

Supplement: Supplementary file 3 — Source Data [file 41467_2025_60409_MOESM3_ESM.zip › SupplementaryData1/Figure3/Fig3c/PulsedESR20240209_212550.png]

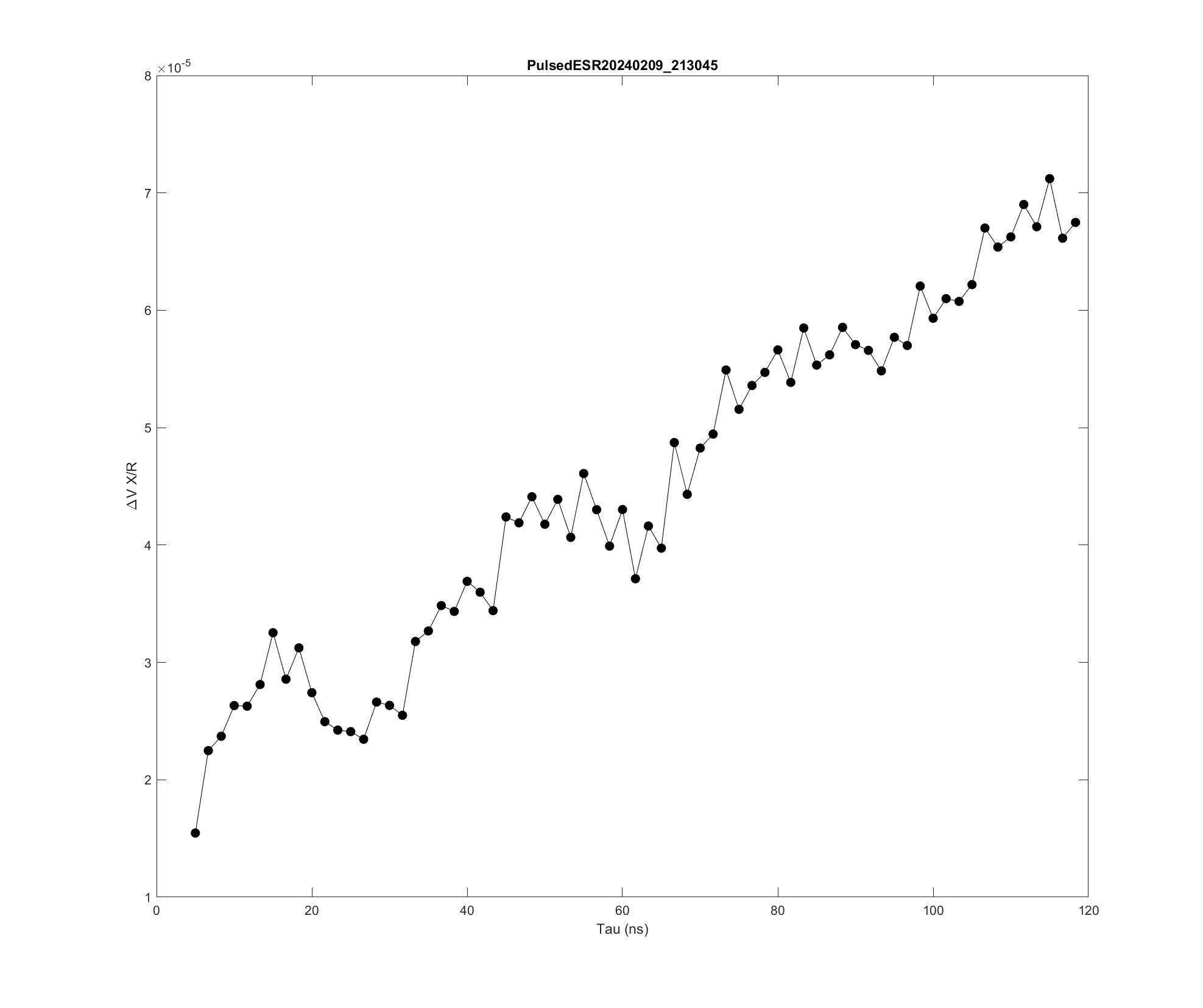

Supplement: Supplementary file 3 — Source Data [file 41467_2025_60409_MOESM3_ESM.zip › SupplementaryData1/Figure3/Fig3c/PulsedESR20240209_213045.png]

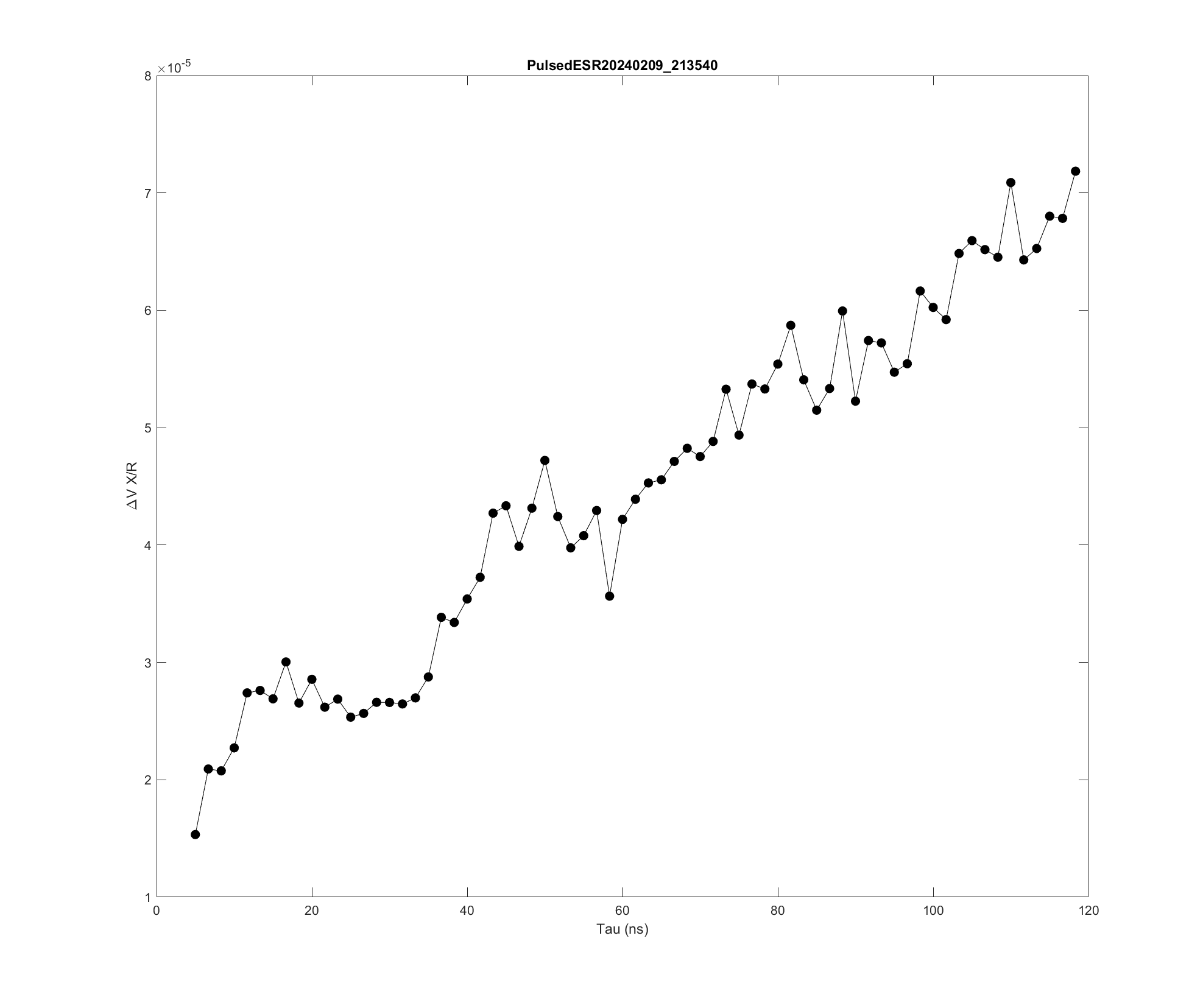

Supplement: Supplementary file 3 — Source Data [file 41467_2025_60409_MOESM3_ESM.zip › SupplementaryData1/Figure3/Fig3c/PulsedESR20240209_213540.png]

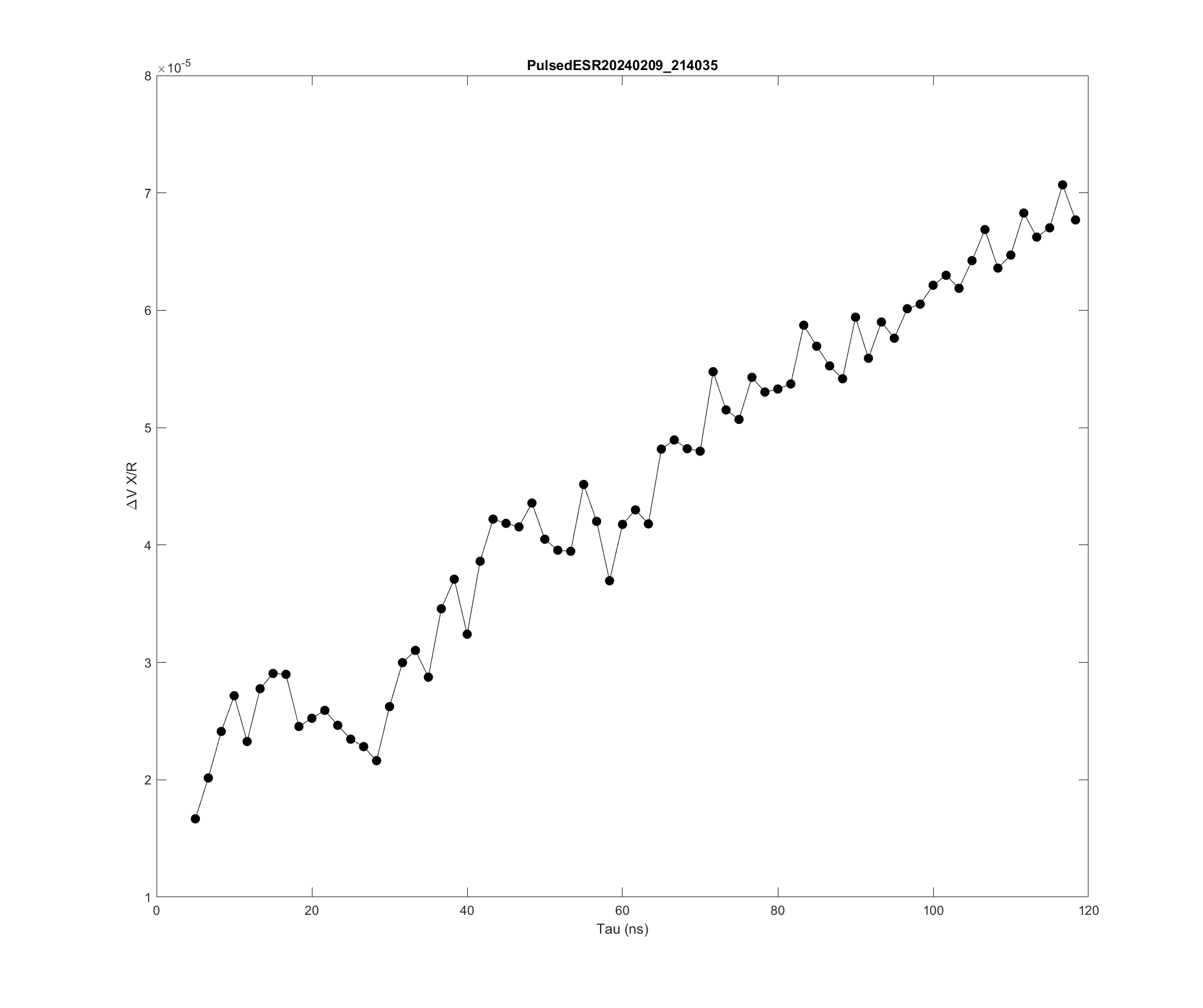

Supplement: Supplementary file 3 — Source Data [file 41467_2025_60409_MOESM3_ESM.zip › SupplementaryData1/Figure3/Fig3c/PulsedESR20240209_214035.png]

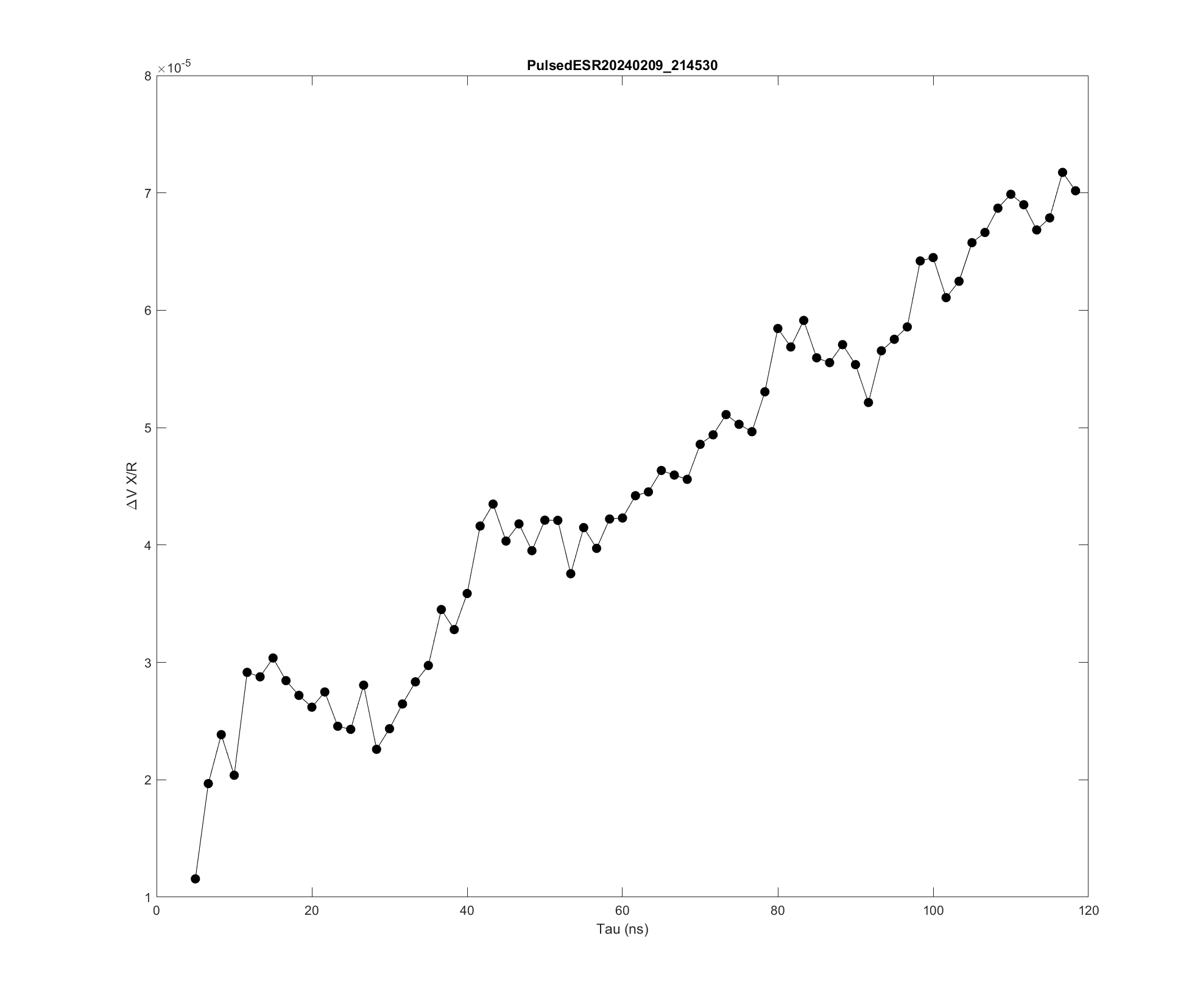

Supplement: Supplementary file 3 — Source Data [file 41467_2025_60409_MOESM3_ESM.zip › SupplementaryData1/Figure3/Fig3c/PulsedESR20240209_214530.png]

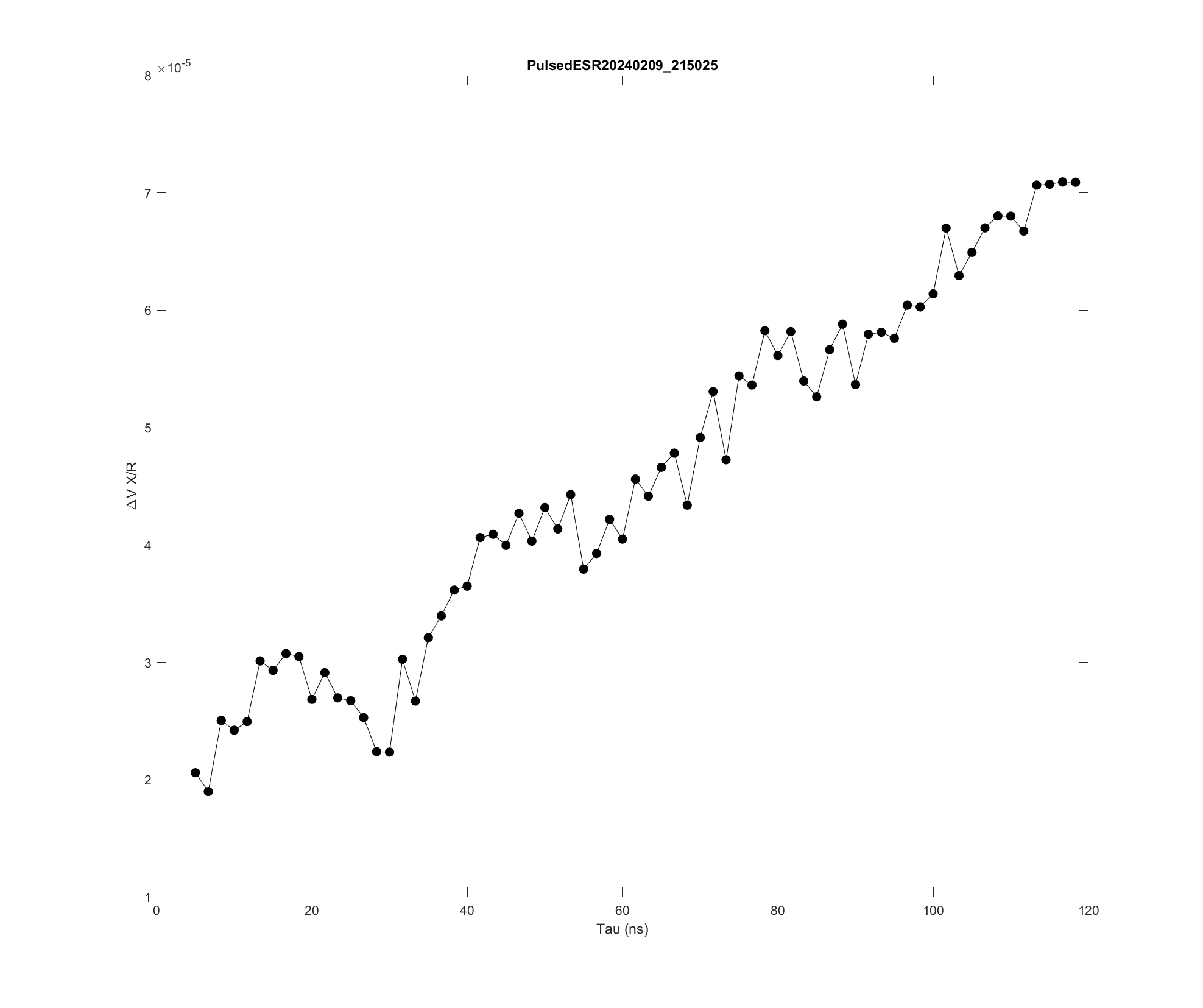

Supplement: Supplementary file 3 — Source Data [file 41467_2025_60409_MOESM3_ESM.zip › SupplementaryData1/Figure3/Fig3c/PulsedESR20240209_215025.png]

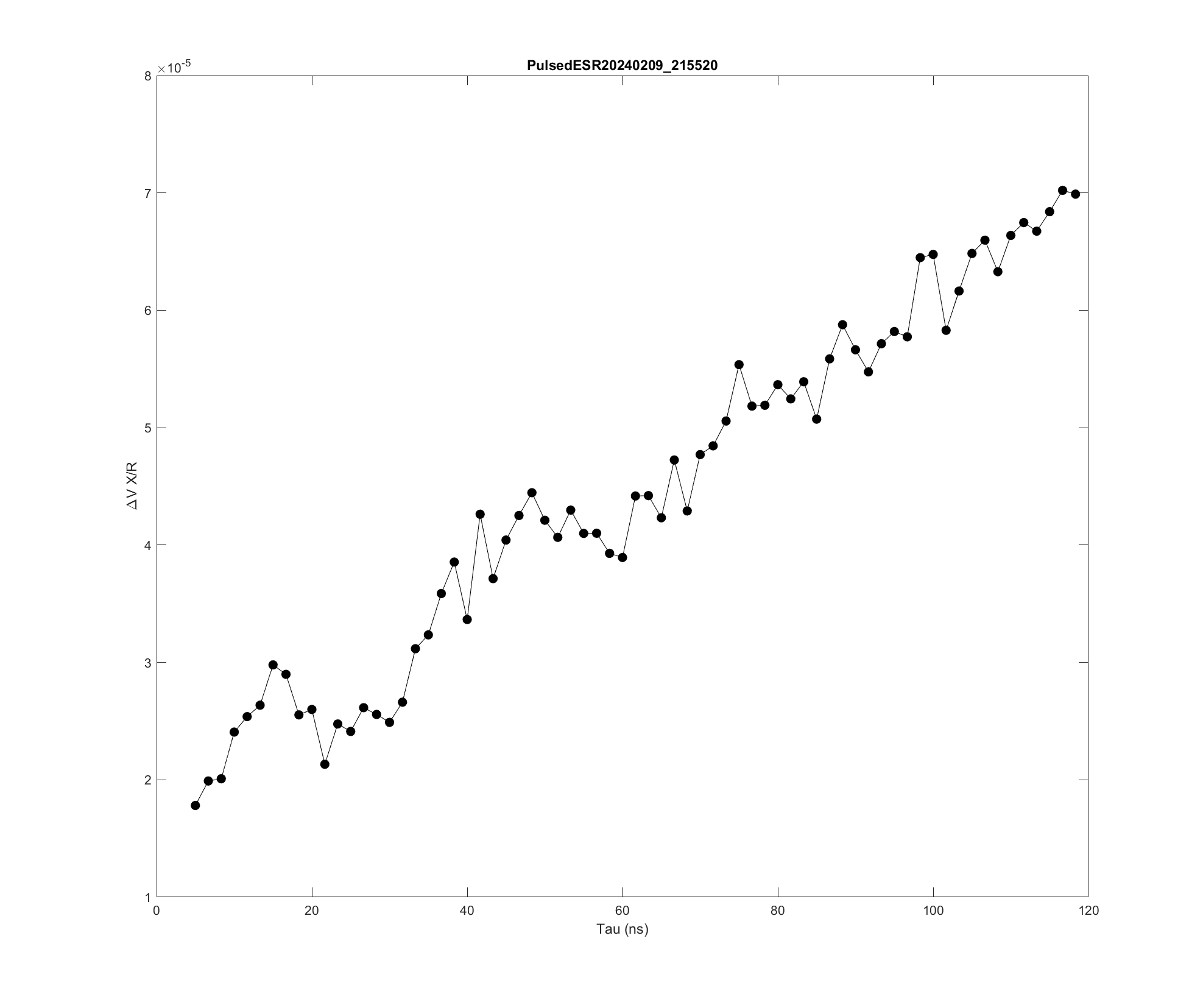

Supplement: Supplementary file 3 — Source Data [file 41467_2025_60409_MOESM3_ESM.zip › SupplementaryData1/Figure3/Fig3c/PulsedESR20240209_215520.png]

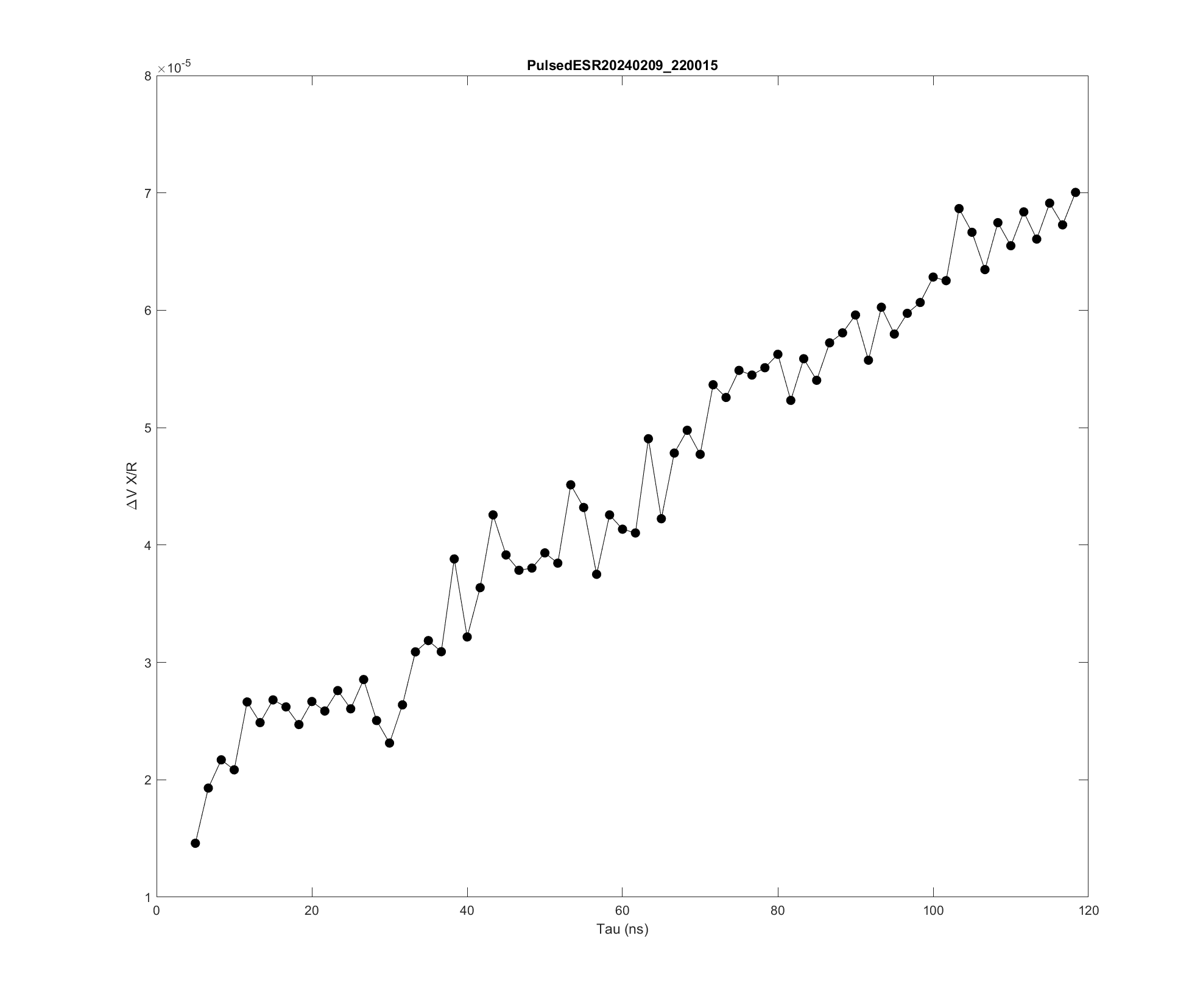

Supplement: Supplementary file 3 — Source Data [file 41467_2025_60409_MOESM3_ESM.zip › SupplementaryData1/Figure3/Fig3c/PulsedESR20240209_220015.png]

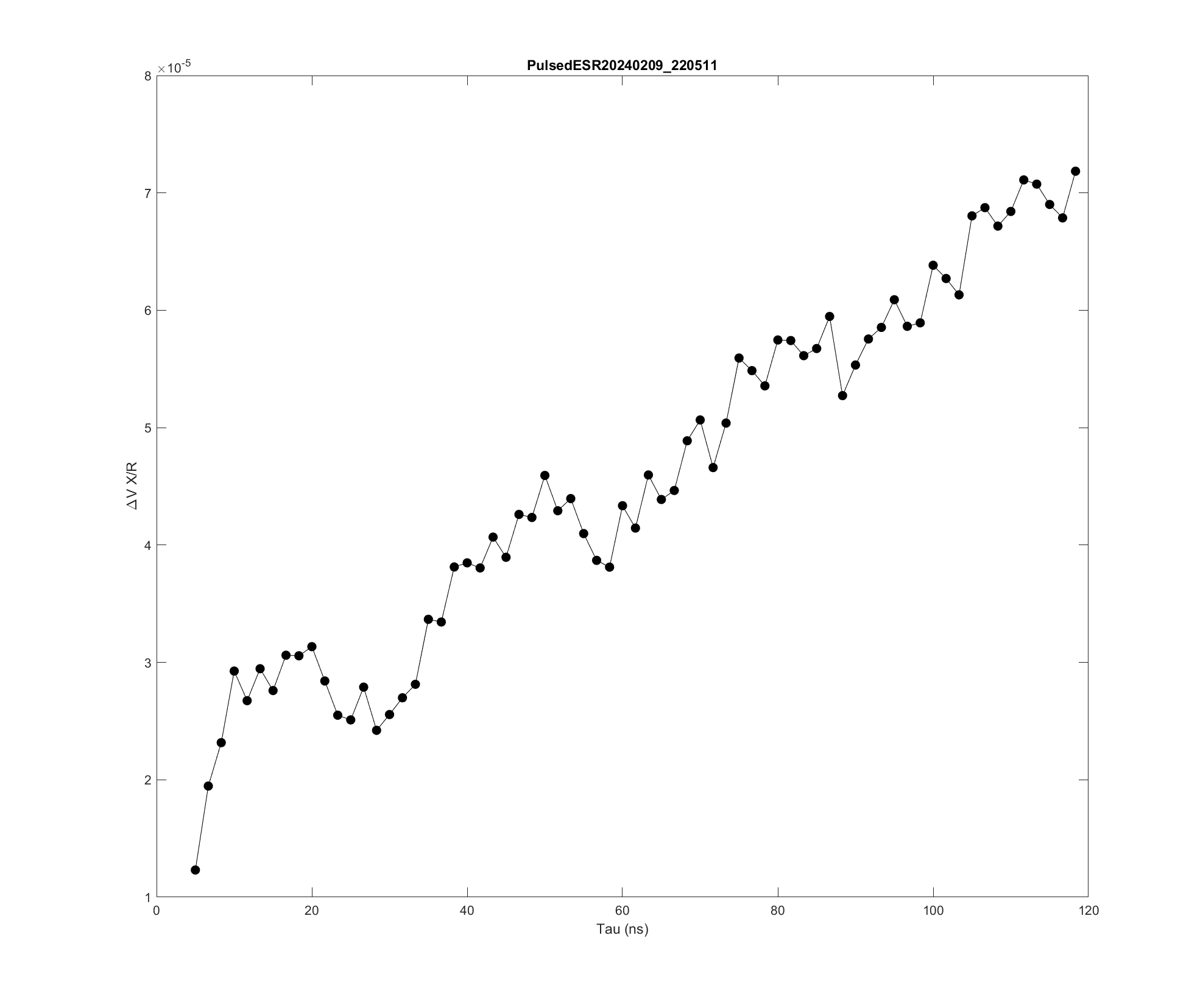

Supplement: Supplementary file 3 — Source Data [file 41467_2025_60409_MOESM3_ESM.zip › SupplementaryData1/Figure3/Fig3c/PulsedESR20240209_220511.png]

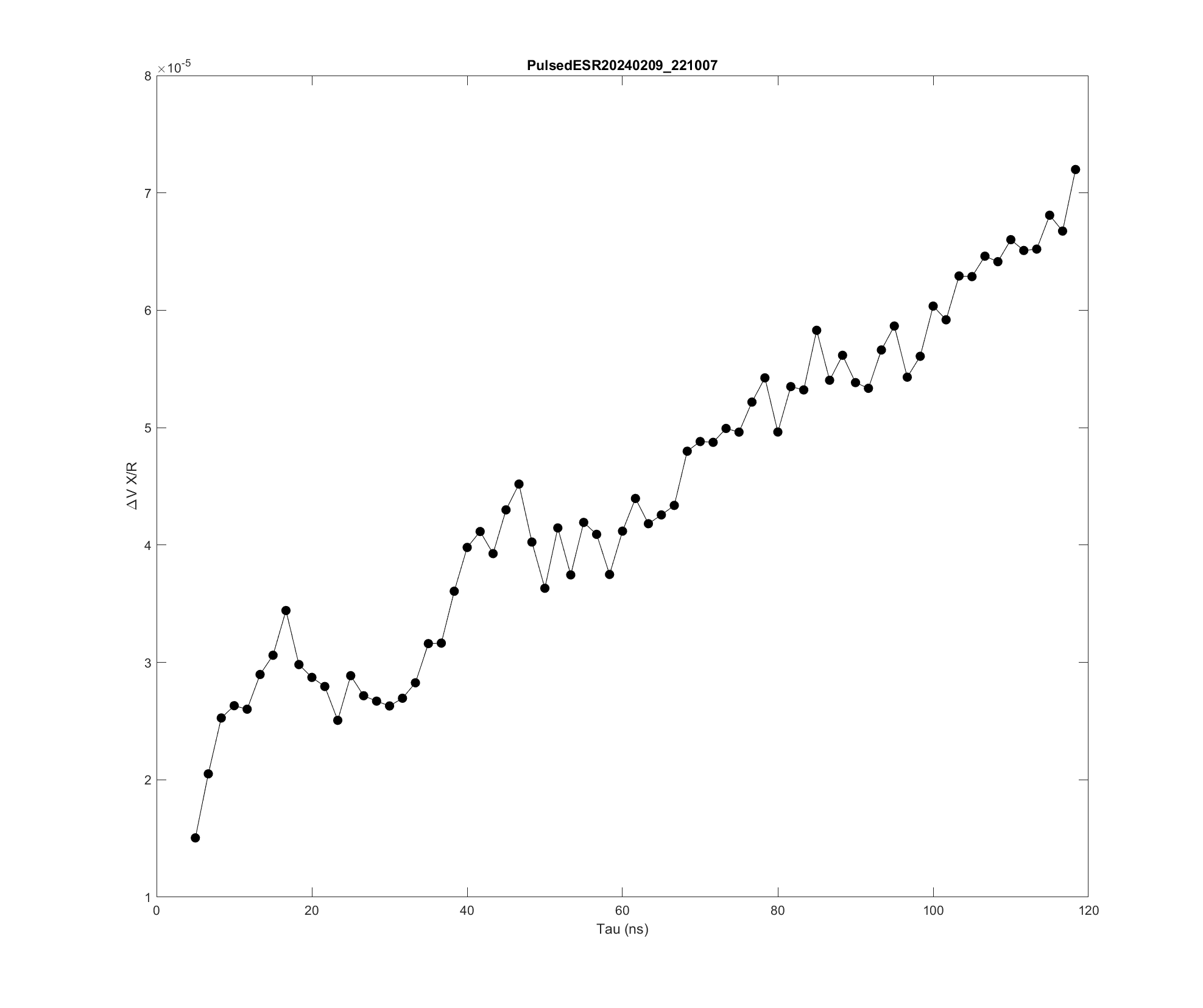

Supplement: Supplementary file 3 — Source Data [file 41467_2025_60409_MOESM3_ESM.zip › SupplementaryData1/Figure3/Fig3c/PulsedESR20240209_221007.png]

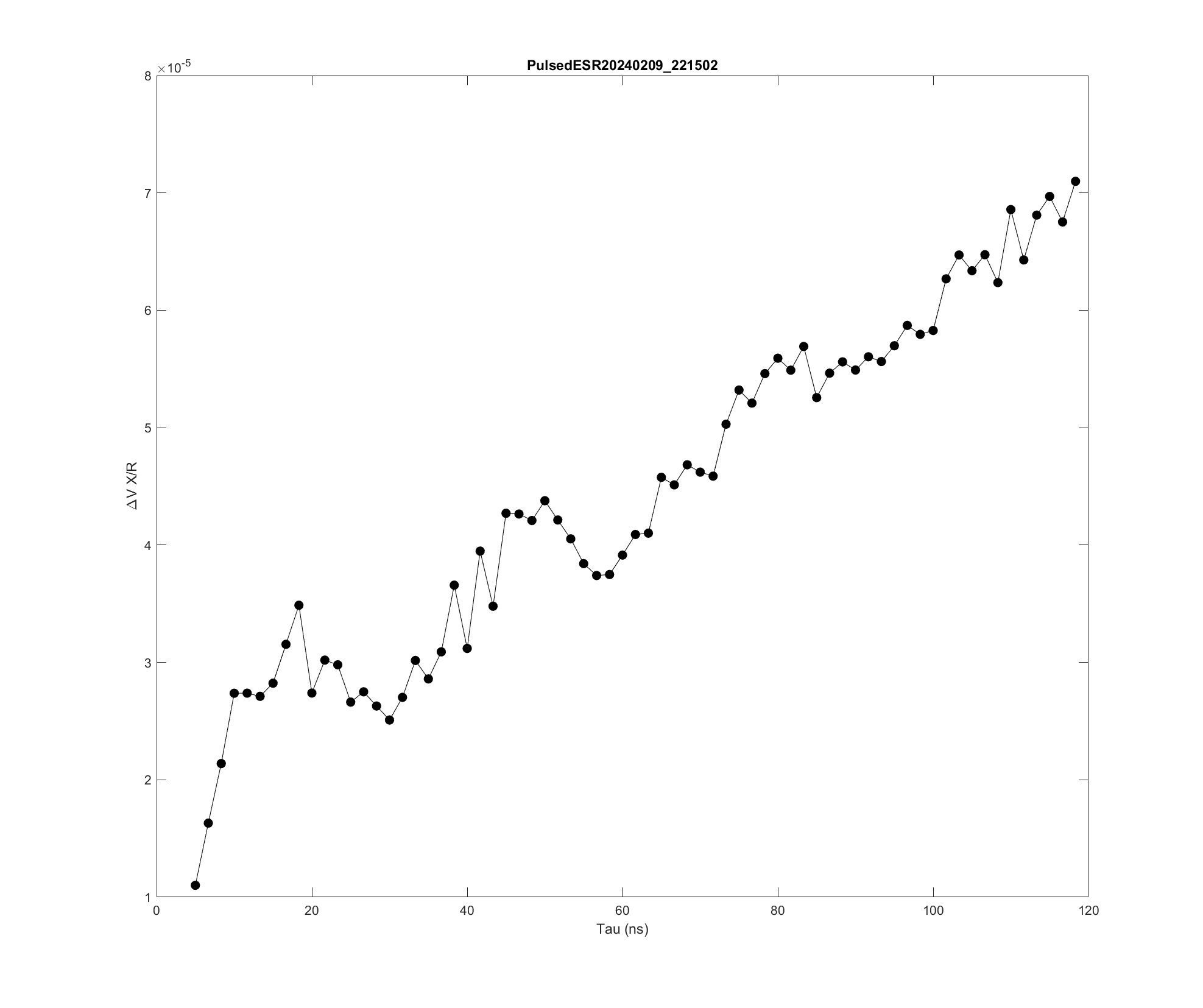

Supplement: Supplementary file 3 — Source Data [file 41467_2025_60409_MOESM3_ESM.zip › SupplementaryData1/Figure3/Fig3c/PulsedESR20240209_221502.png]

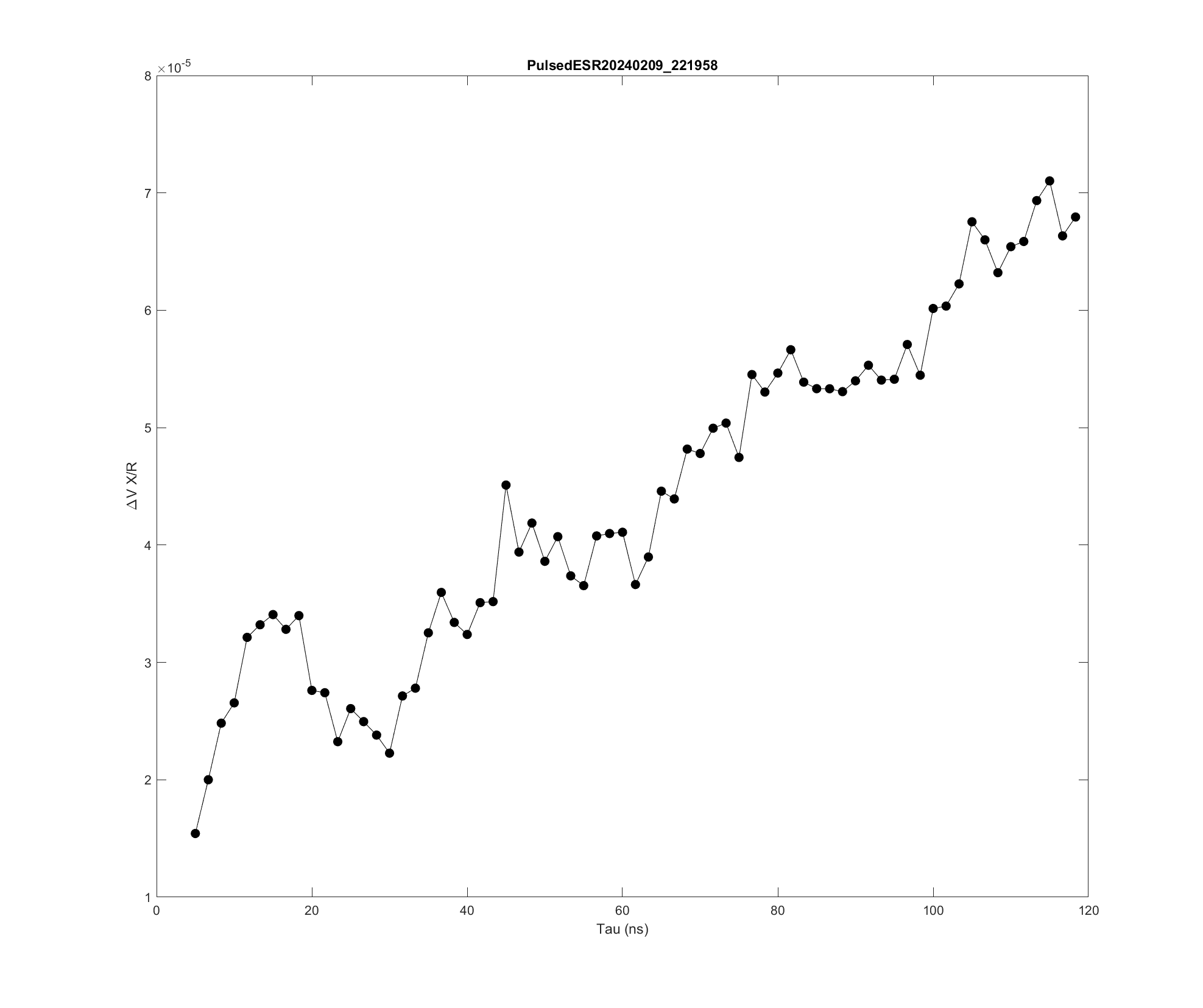

Supplement: Supplementary file 3 — Source Data [file 41467_2025_60409_MOESM3_ESM.zip › SupplementaryData1/Figure3/Fig3c/PulsedESR20240209_221958.png]

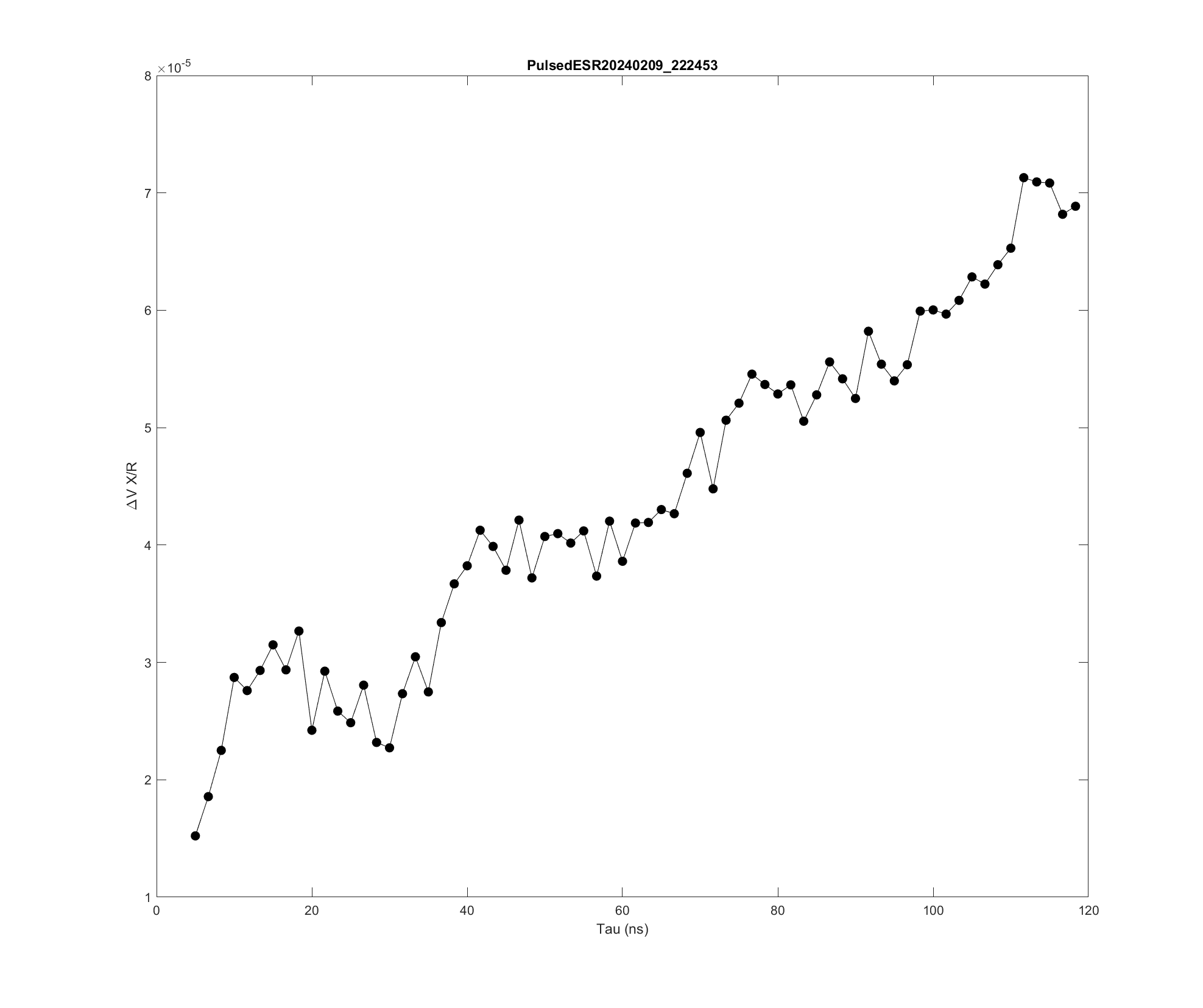

Supplement: Supplementary file 3 — Source Data [file 41467_2025_60409_MOESM3_ESM.zip › SupplementaryData1/Figure3/Fig3c/PulsedESR20240209_222453.png]

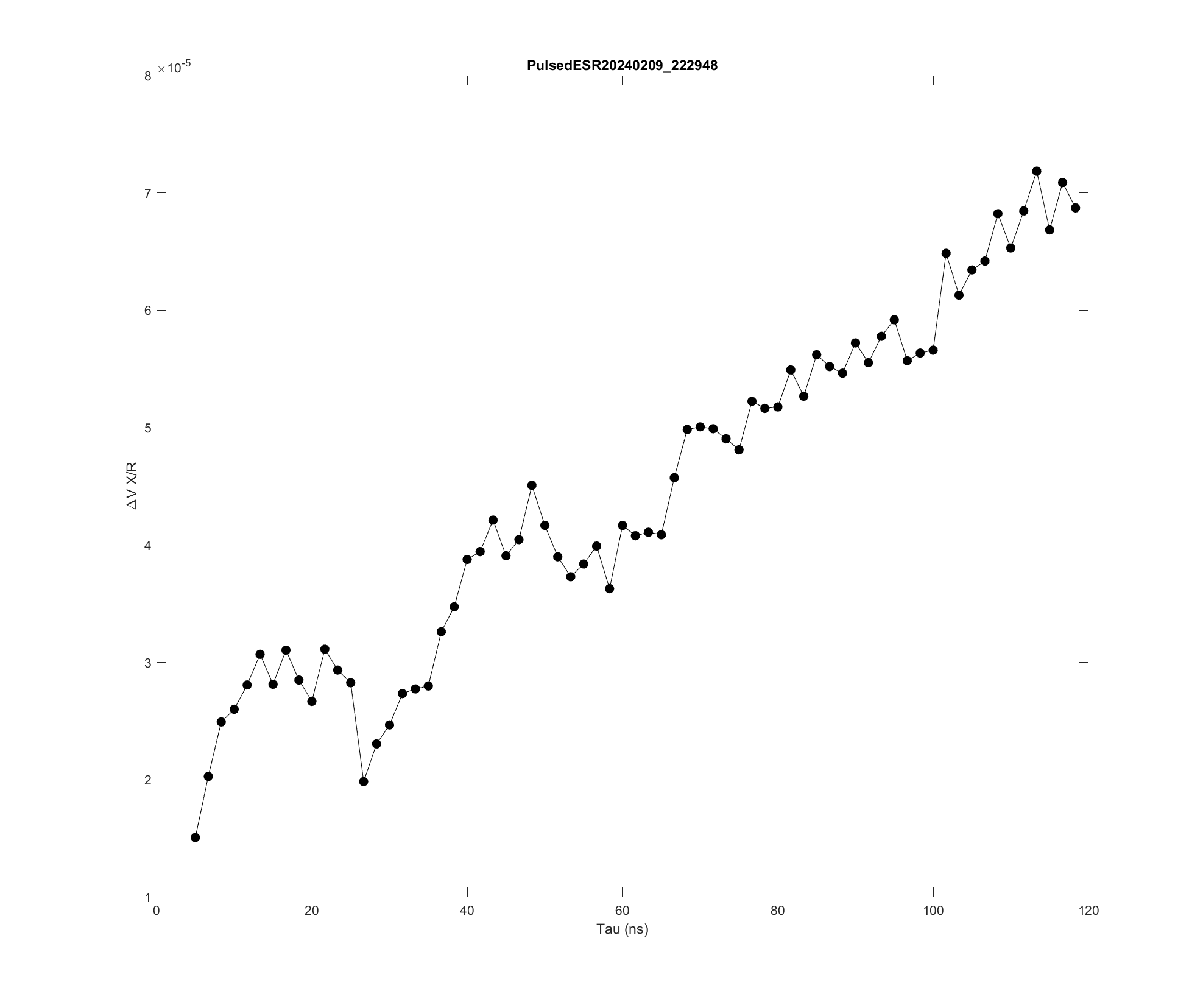

Supplement: Supplementary file 3 — Source Data [file 41467_2025_60409_MOESM3_ESM.zip › SupplementaryData1/Figure3/Fig3c/PulsedESR20240209_222948.png]

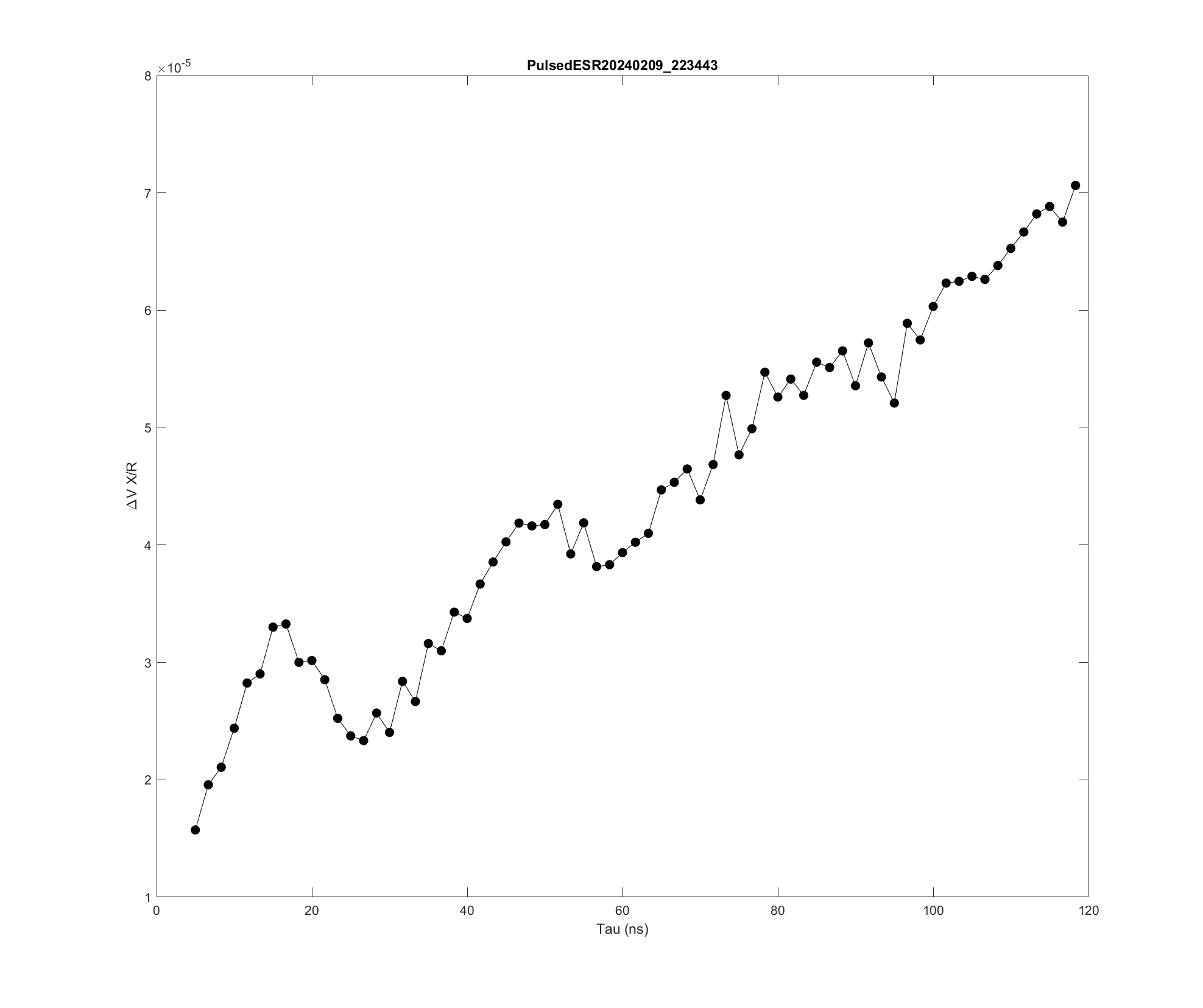

Supplement: Supplementary file 3 — Source Data [file 41467_2025_60409_MOESM3_ESM.zip › SupplementaryData1/Figure3/Fig3c/PulsedESR20240209_223443.png]

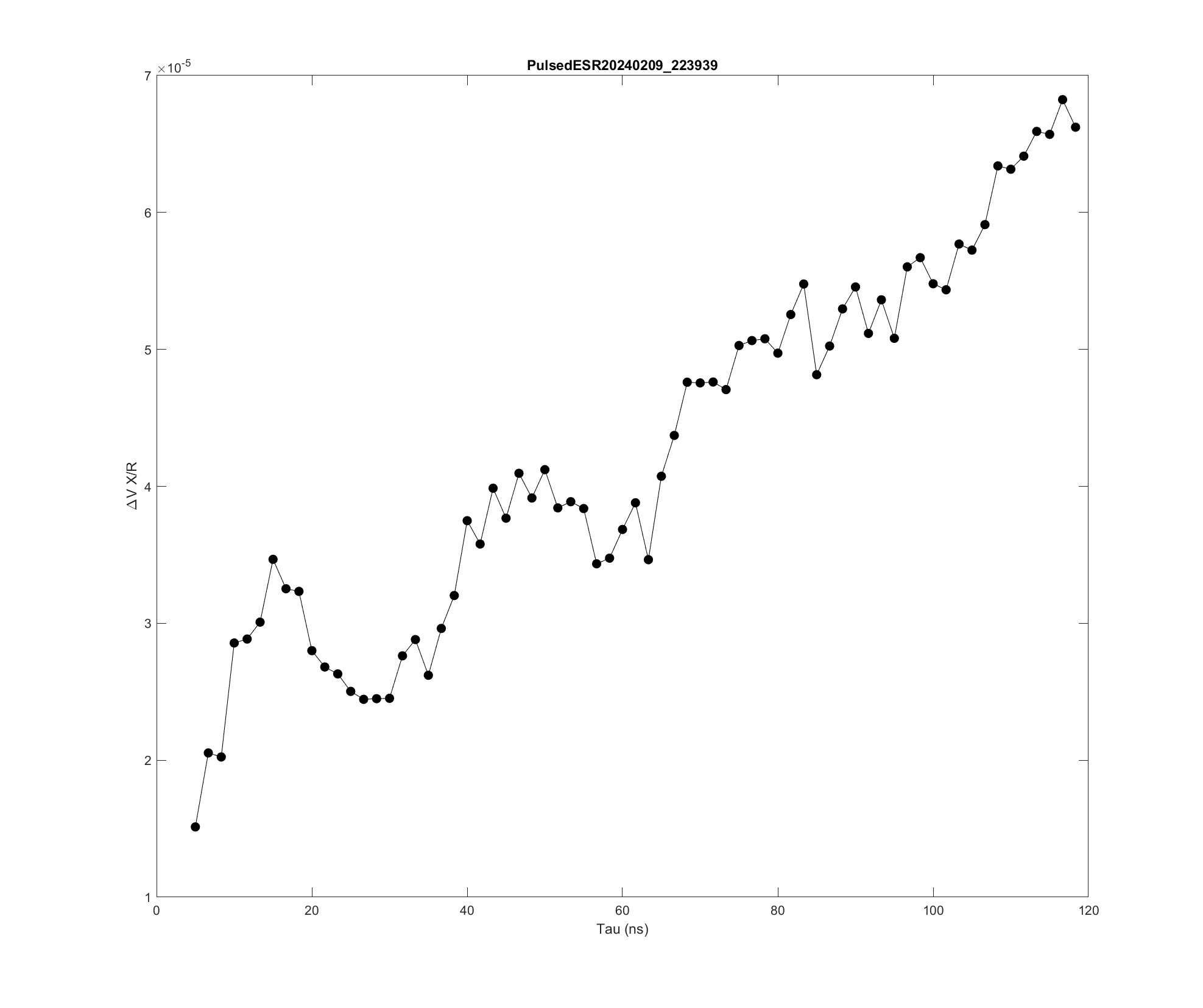

Supplement: Supplementary file 3 — Source Data [file 41467_2025_60409_MOESM3_ESM.zip › SupplementaryData1/Figure3/Fig3c/PulsedESR20240209_223939.png]

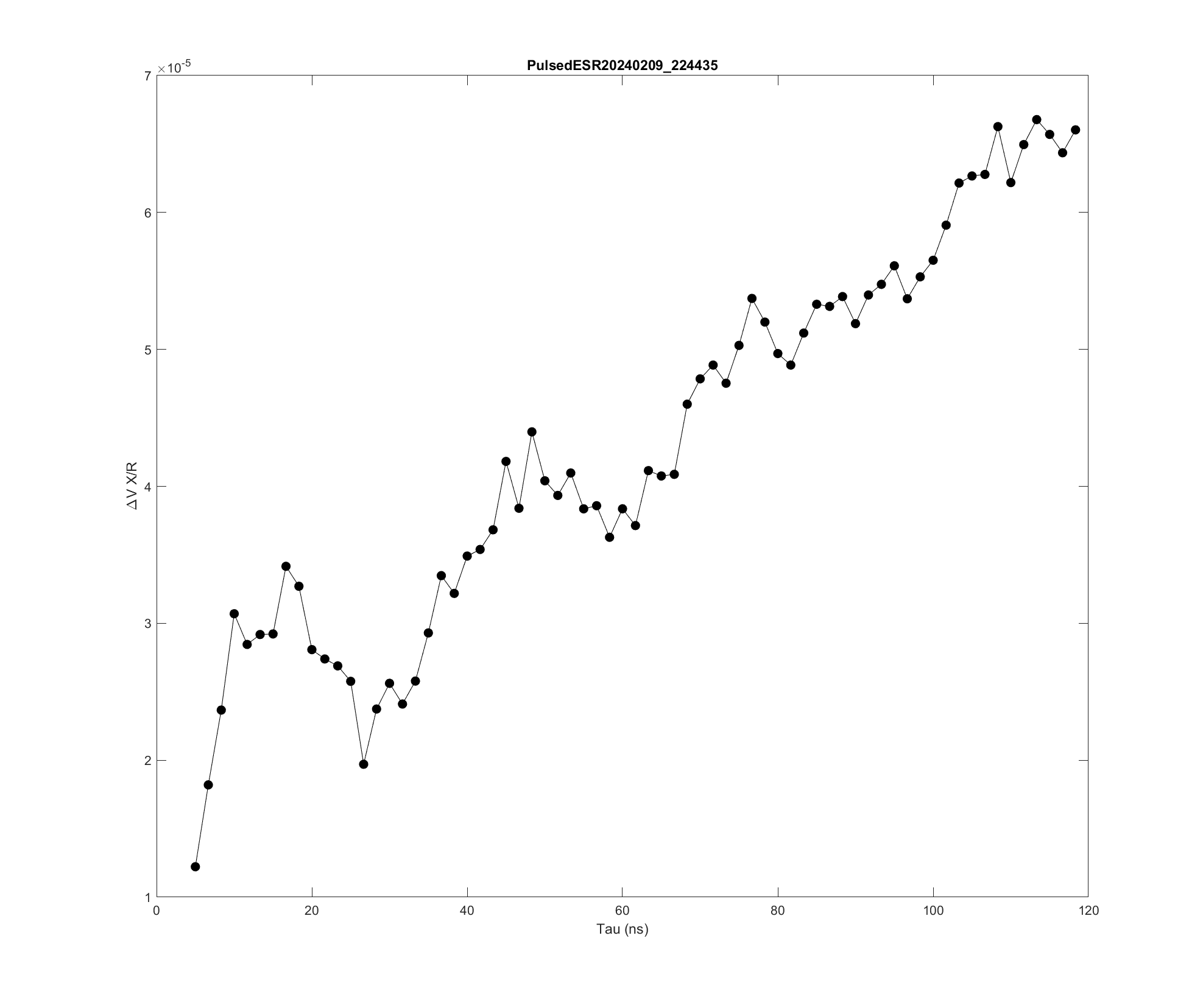

Supplement: Supplementary file 3 — Source Data [file 41467_2025_60409_MOESM3_ESM.zip › SupplementaryData1/Figure3/Fig3c/PulsedESR20240209_224435.png]

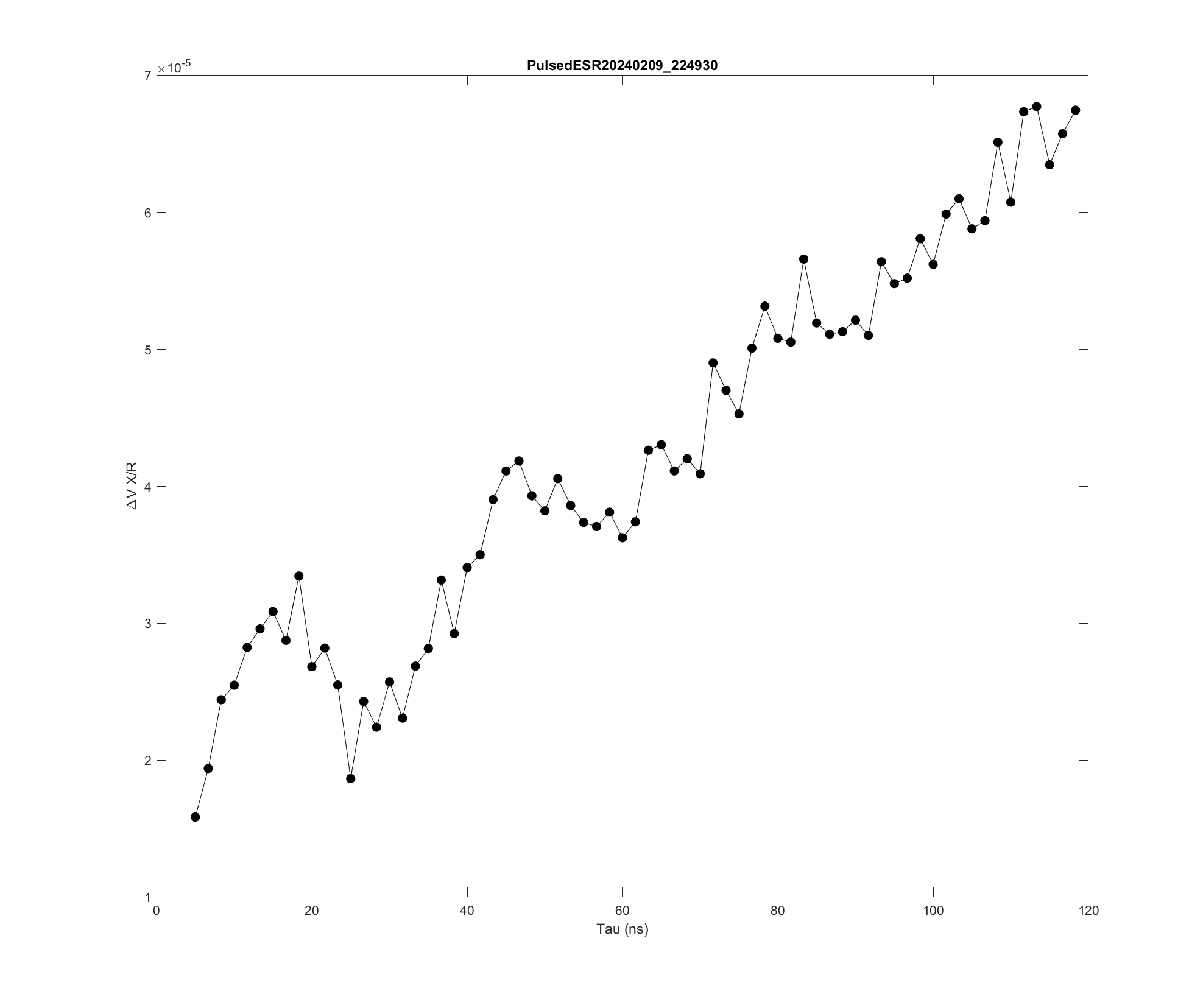

Supplement: Supplementary file 3 — Source Data [file 41467_2025_60409_MOESM3_ESM.zip › SupplementaryData1/Figure3/Fig3c/PulsedESR20240209_224930.png]

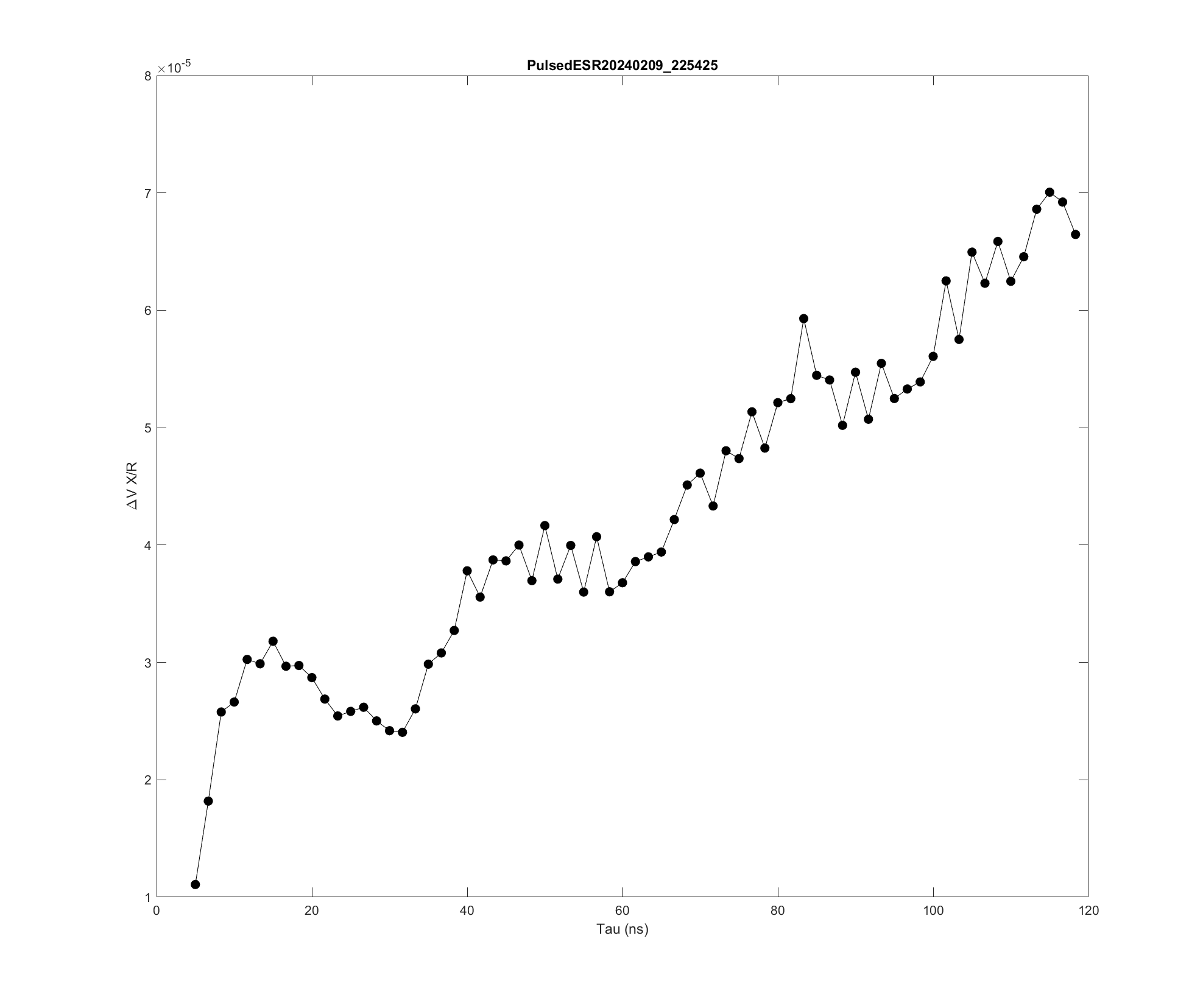

Supplement: Supplementary file 3 — Source Data [file 41467_2025_60409_MOESM3_ESM.zip › SupplementaryData1/Figure3/Fig3c/PulsedESR20240209_225425.png]

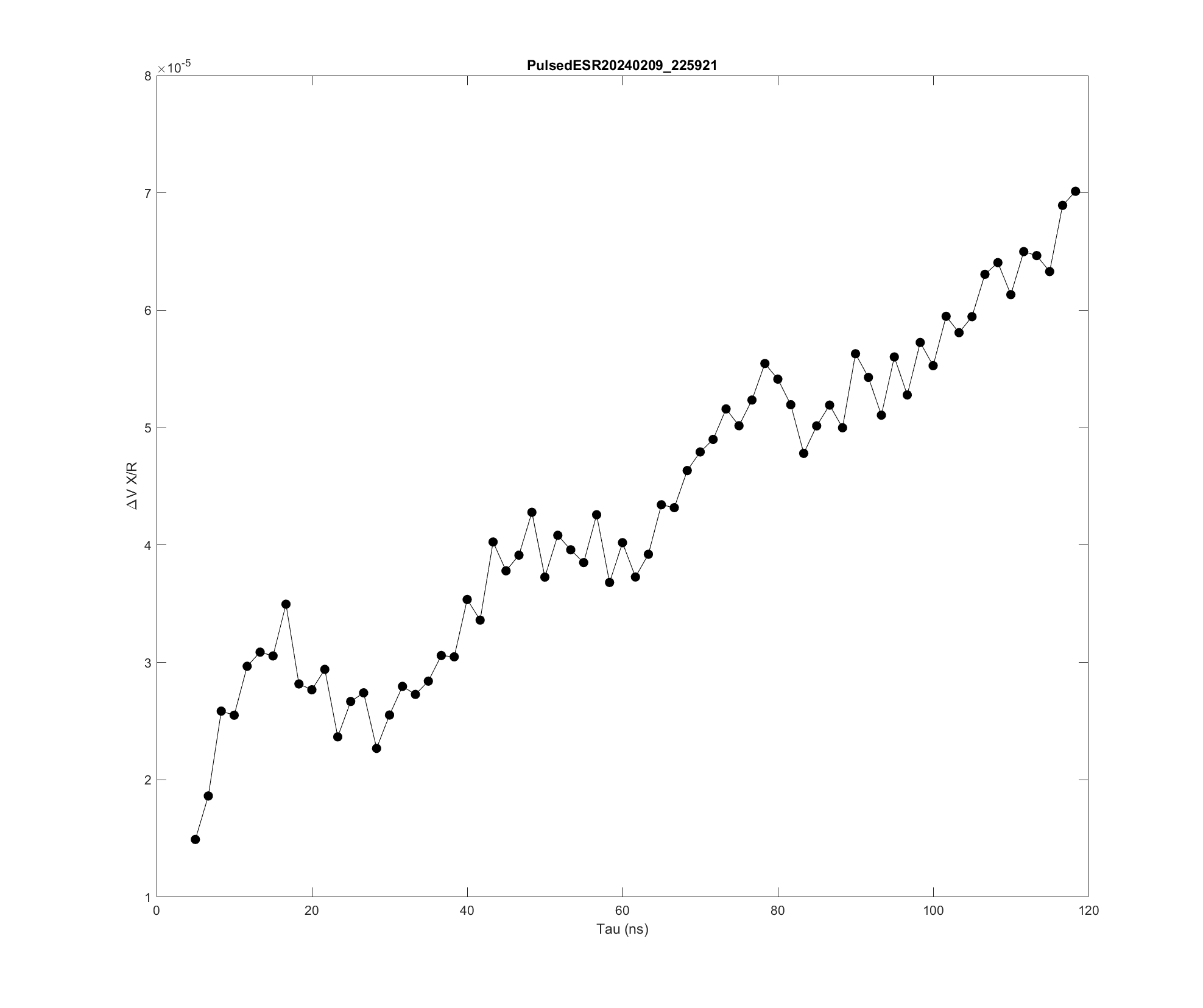

Supplement: Supplementary file 3 — Source Data [file 41467_2025_60409_MOESM3_ESM.zip › SupplementaryData1/Figure3/Fig3c/PulsedESR20240209_225921.png]

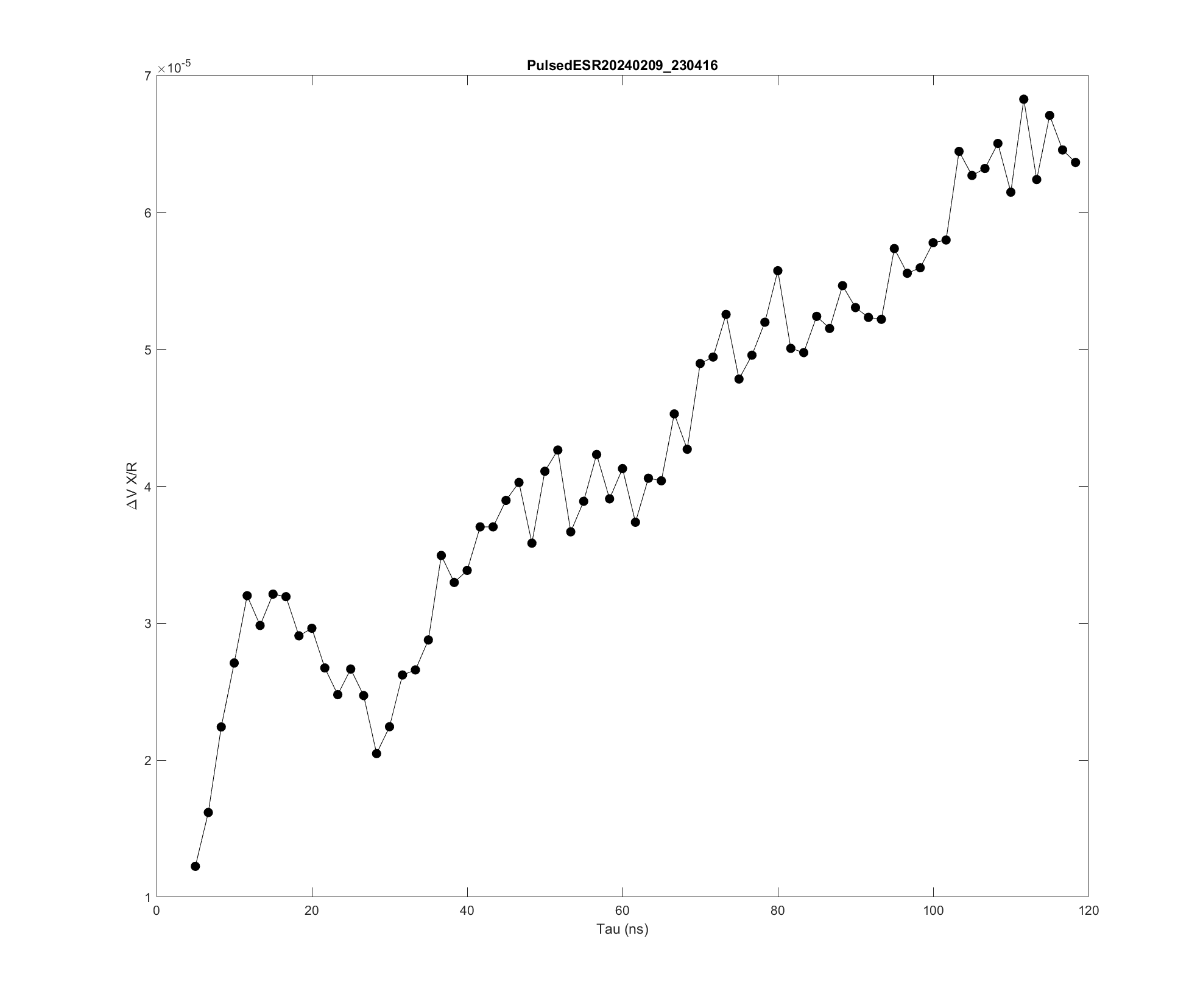

Supplement: Supplementary file 3 — Source Data [file 41467_2025_60409_MOESM3_ESM.zip › SupplementaryData1/Figure3/Fig3c/PulsedESR20240209_230416.png]

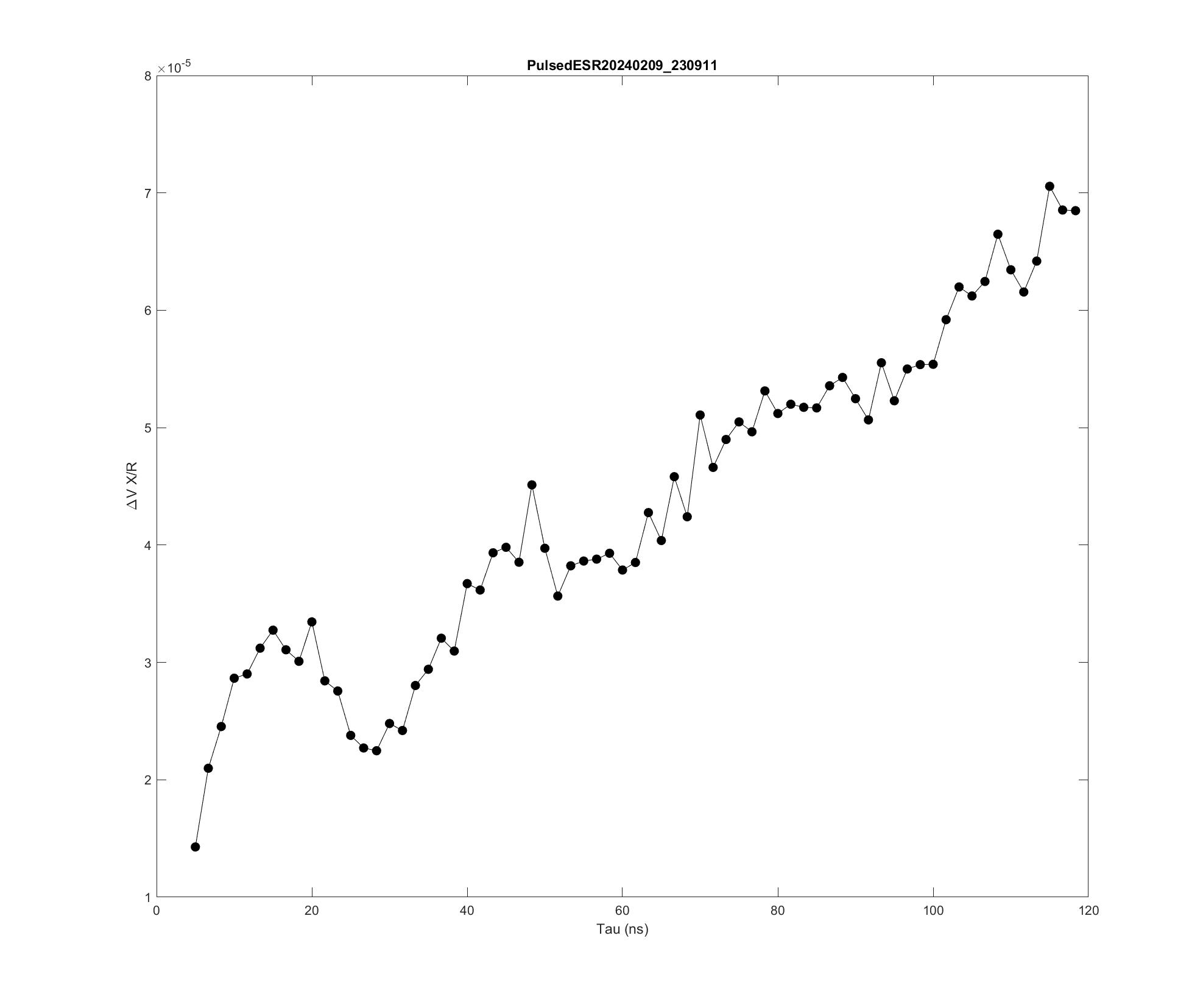

Supplement: Supplementary file 3 — Source Data [file 41467_2025_60409_MOESM3_ESM.zip › SupplementaryData1/Figure3/Fig3c/PulsedESR20240209_230911.png]

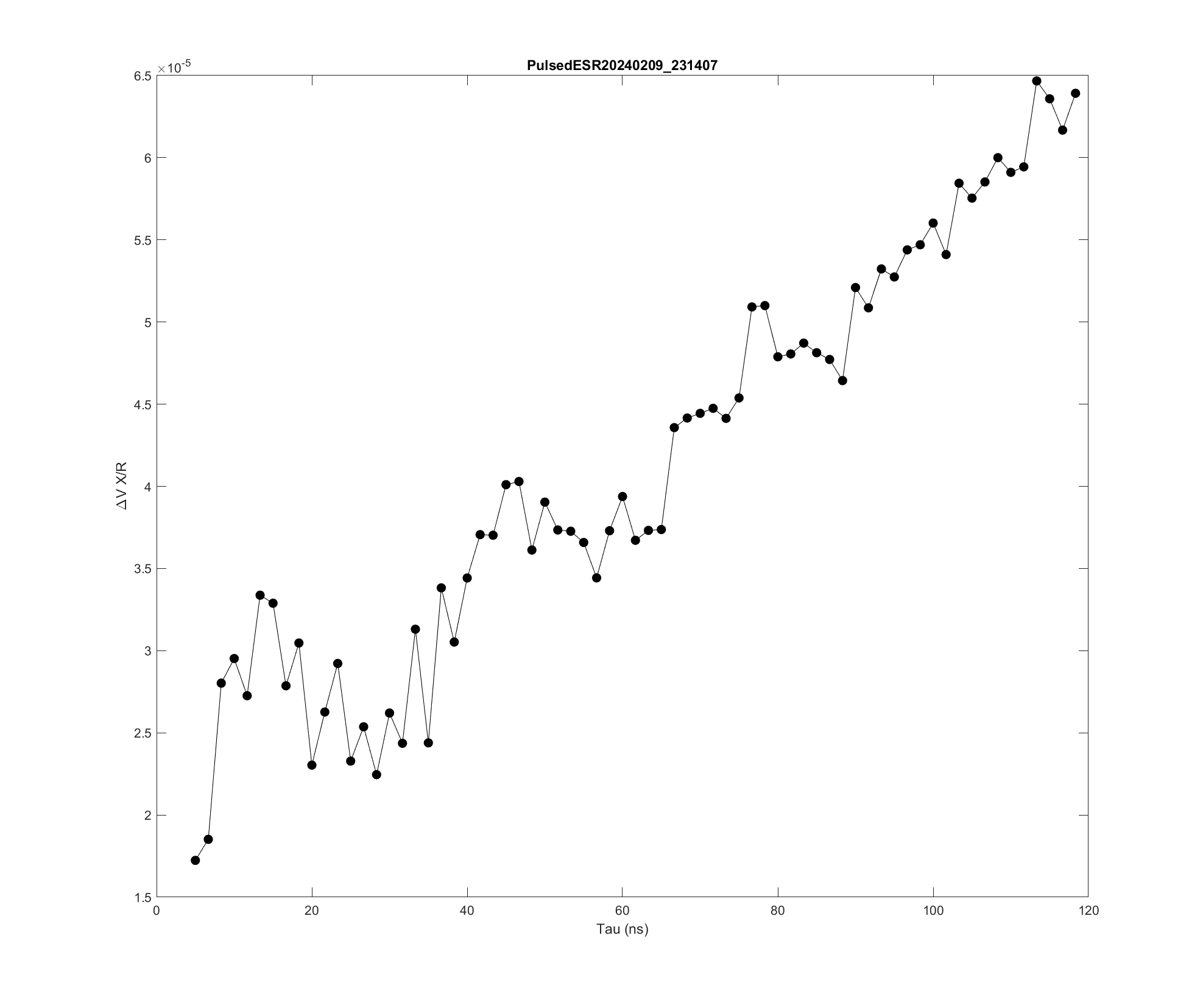

Supplement: Supplementary file 3 — Source Data [file 41467_2025_60409_MOESM3_ESM.zip › SupplementaryData1/Figure3/Fig3c/PulsedESR20240209_231407.png]

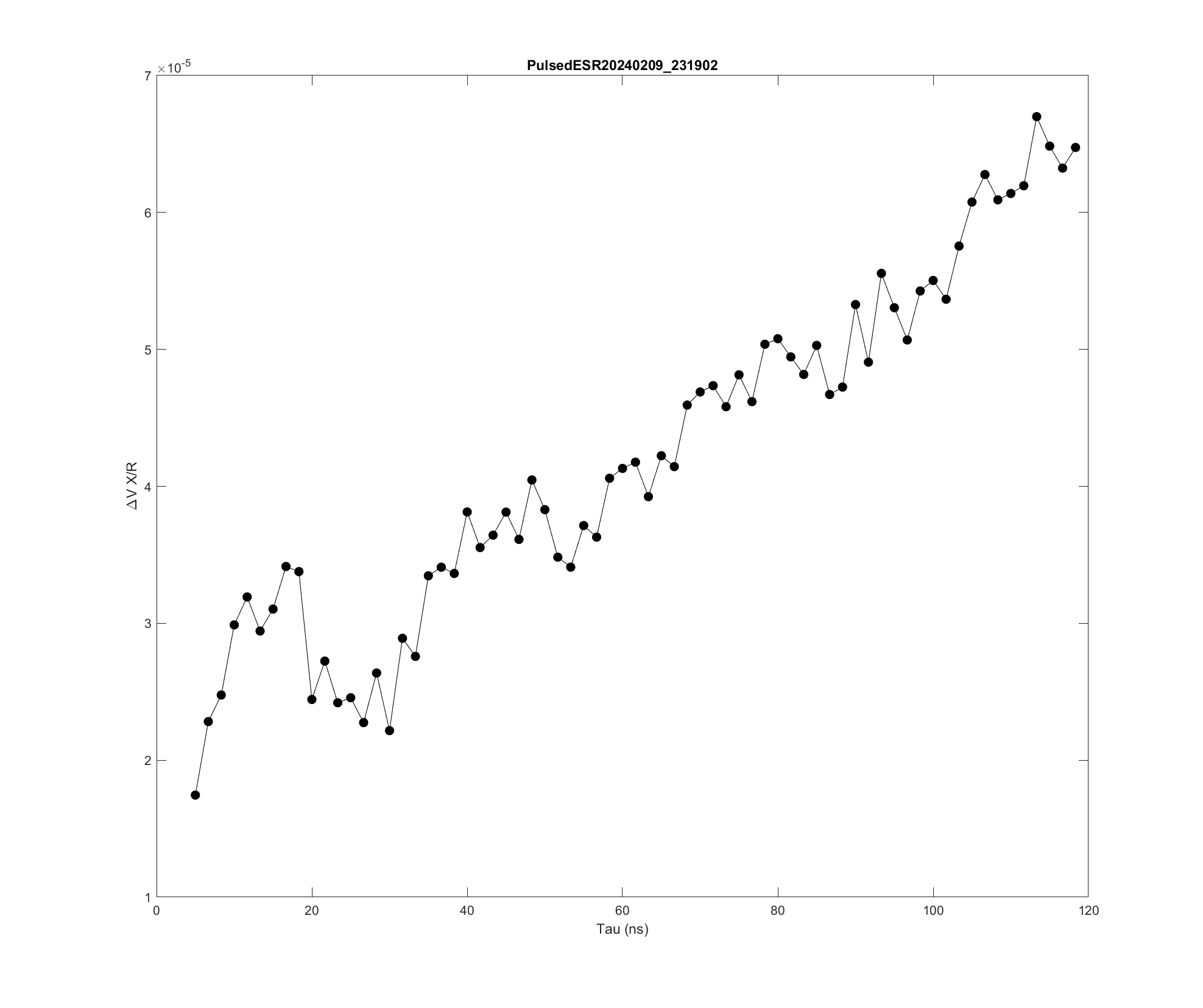

Supplement: Supplementary file 3 — Source Data [file 41467_2025_60409_MOESM3_ESM.zip › SupplementaryData1/Figure3/Fig3c/PulsedESR20240209_231902.png]

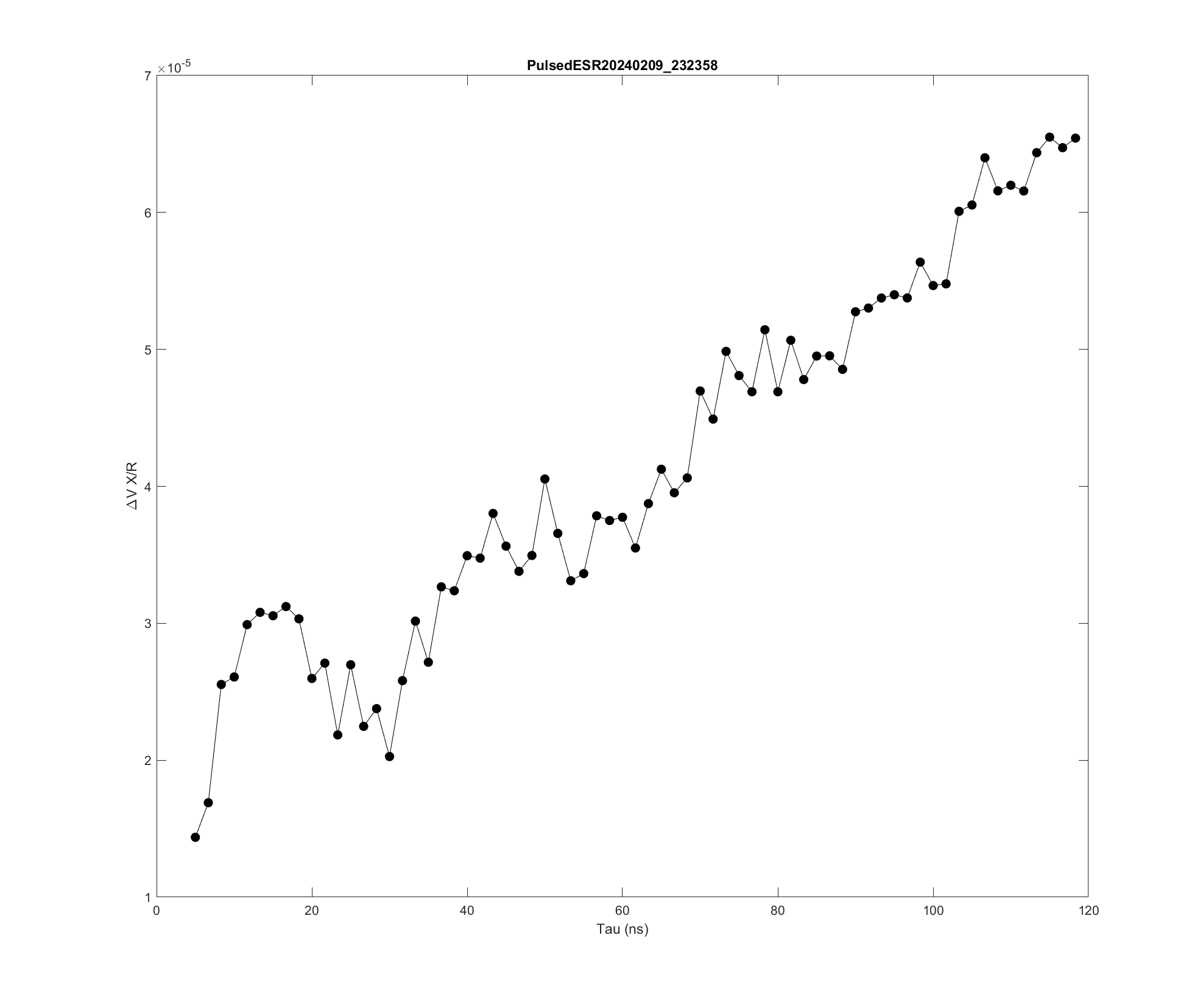

Supplement: Supplementary file 3 — Source Data [file 41467_2025_60409_MOESM3_ESM.zip › SupplementaryData1/Figure3/Fig3c/PulsedESR20240209_232358.png]

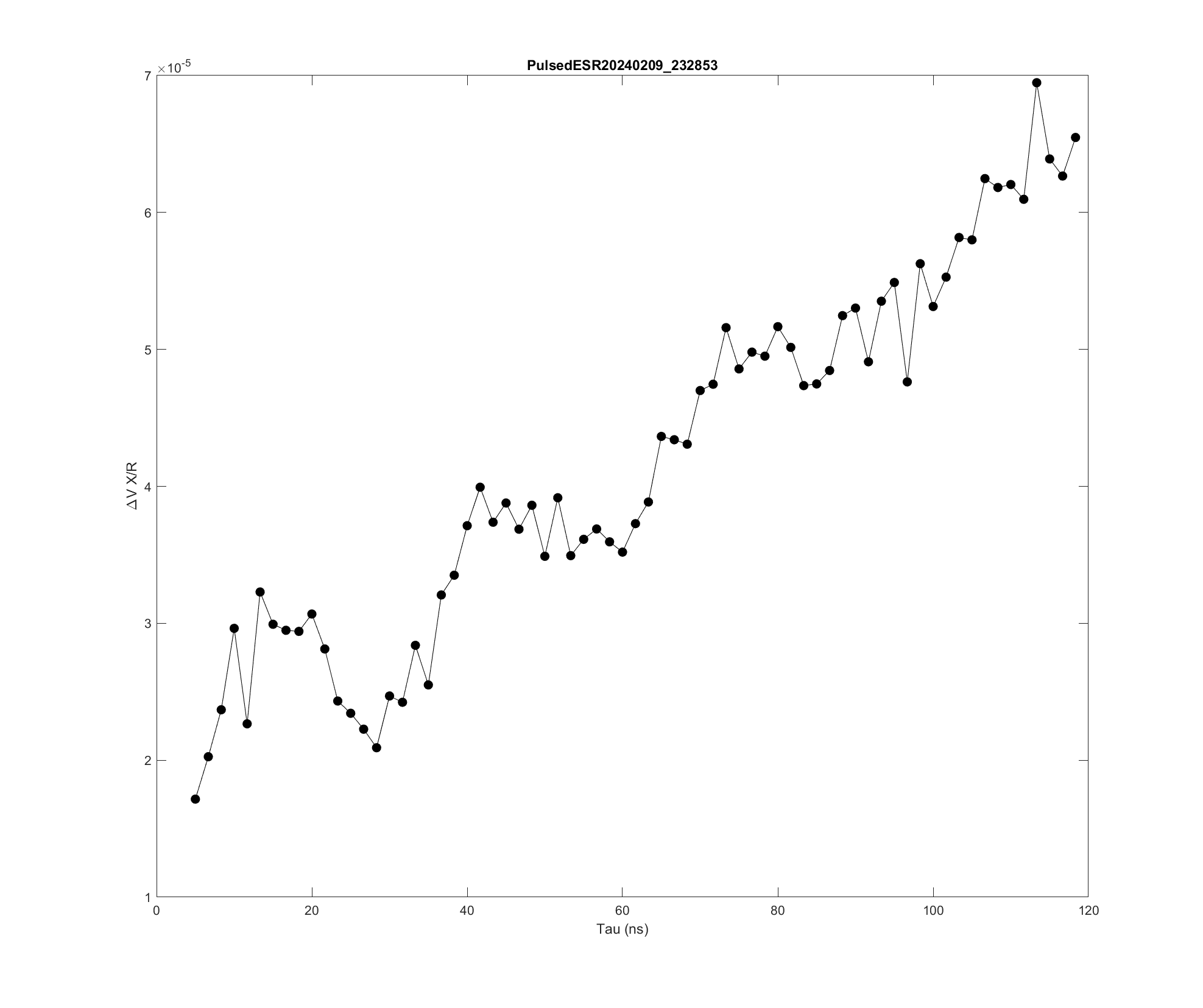

Supplement: Supplementary file 3 — Source Data [file 41467_2025_60409_MOESM3_ESM.zip › SupplementaryData1/Figure3/Fig3c/PulsedESR20240209_232853.png]

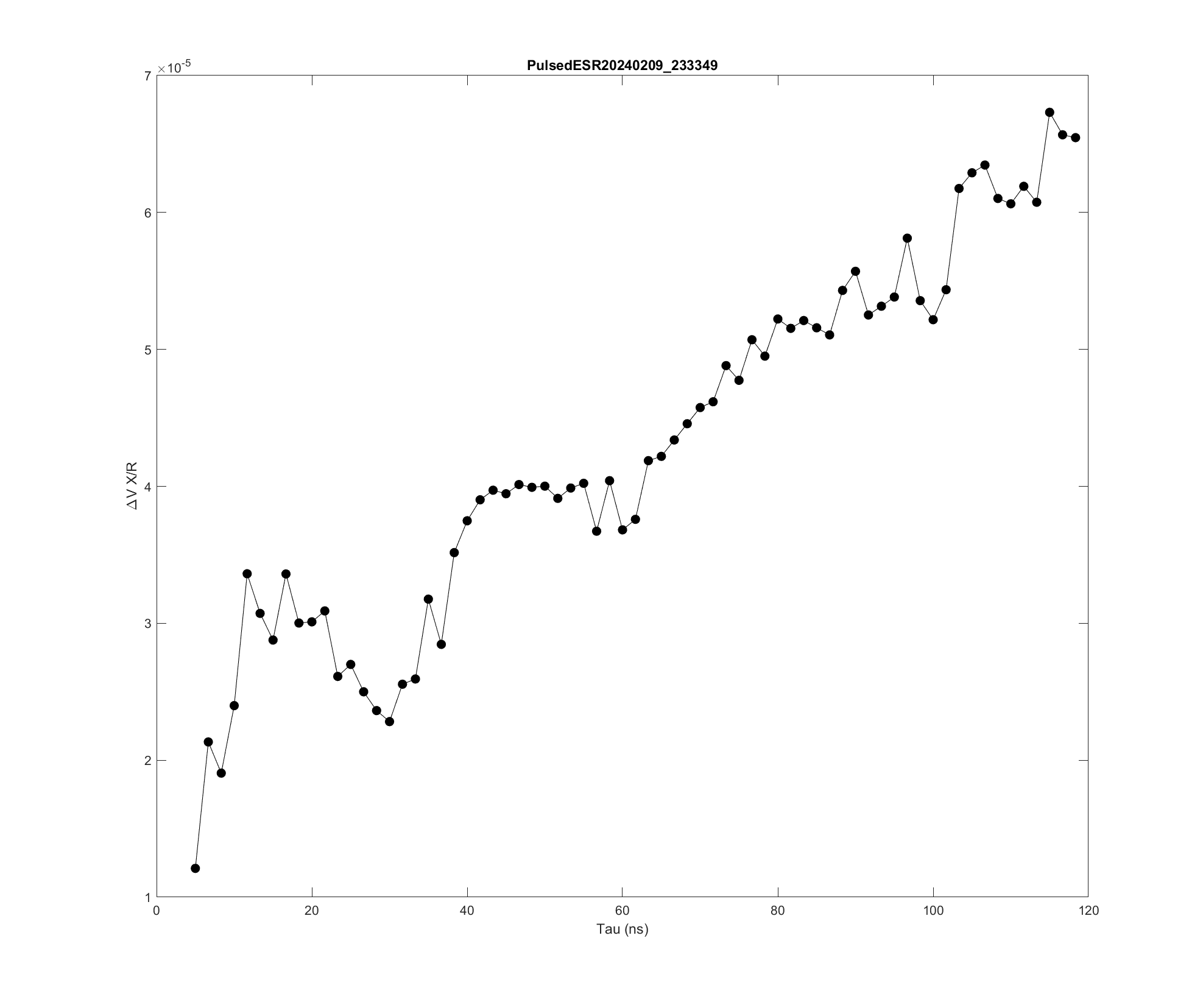

Supplement: Supplementary file 3 — Source Data [file 41467_2025_60409_MOESM3_ESM.zip › SupplementaryData1/Figure3/Fig3c/PulsedESR20240209_233349.png]

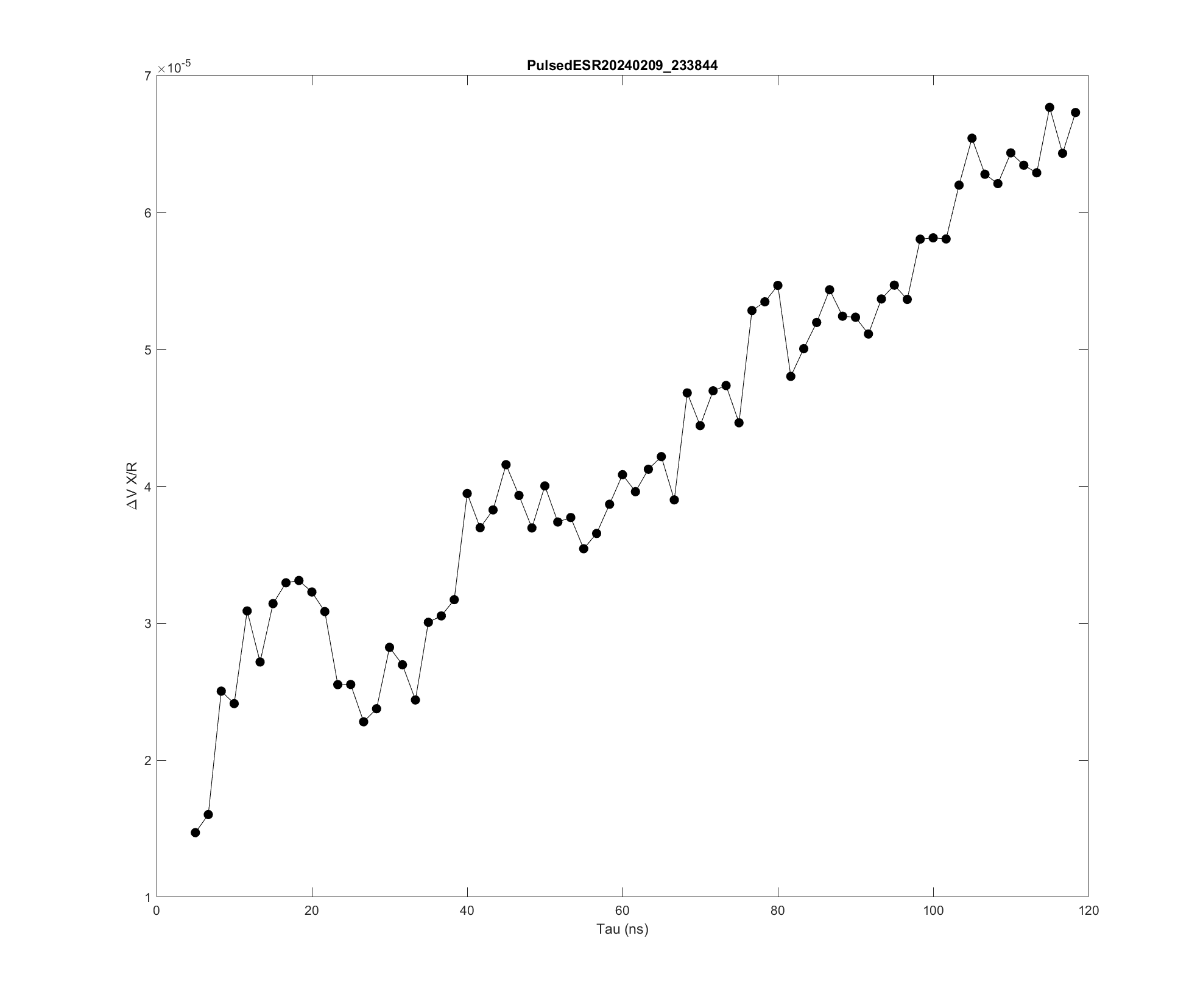

Supplement: Supplementary file 3 — Source Data [file 41467_2025_60409_MOESM3_ESM.zip › SupplementaryData1/Figure3/Fig3c/PulsedESR20240209_233844.png]

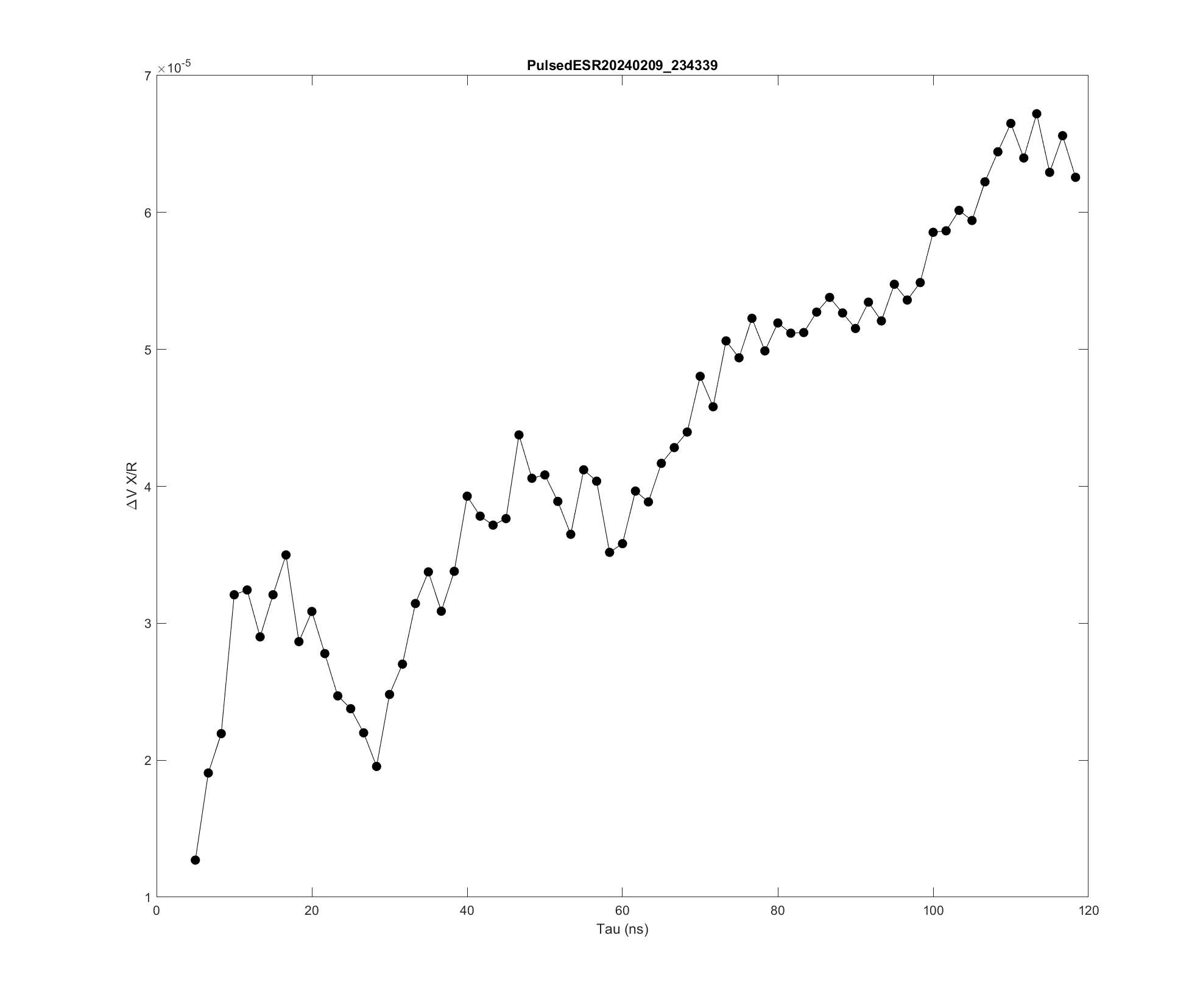

Supplement: Supplementary file 3 — Source Data [file 41467_2025_60409_MOESM3_ESM.zip › SupplementaryData1/Figure3/Fig3c/PulsedESR20240209_234339.png]

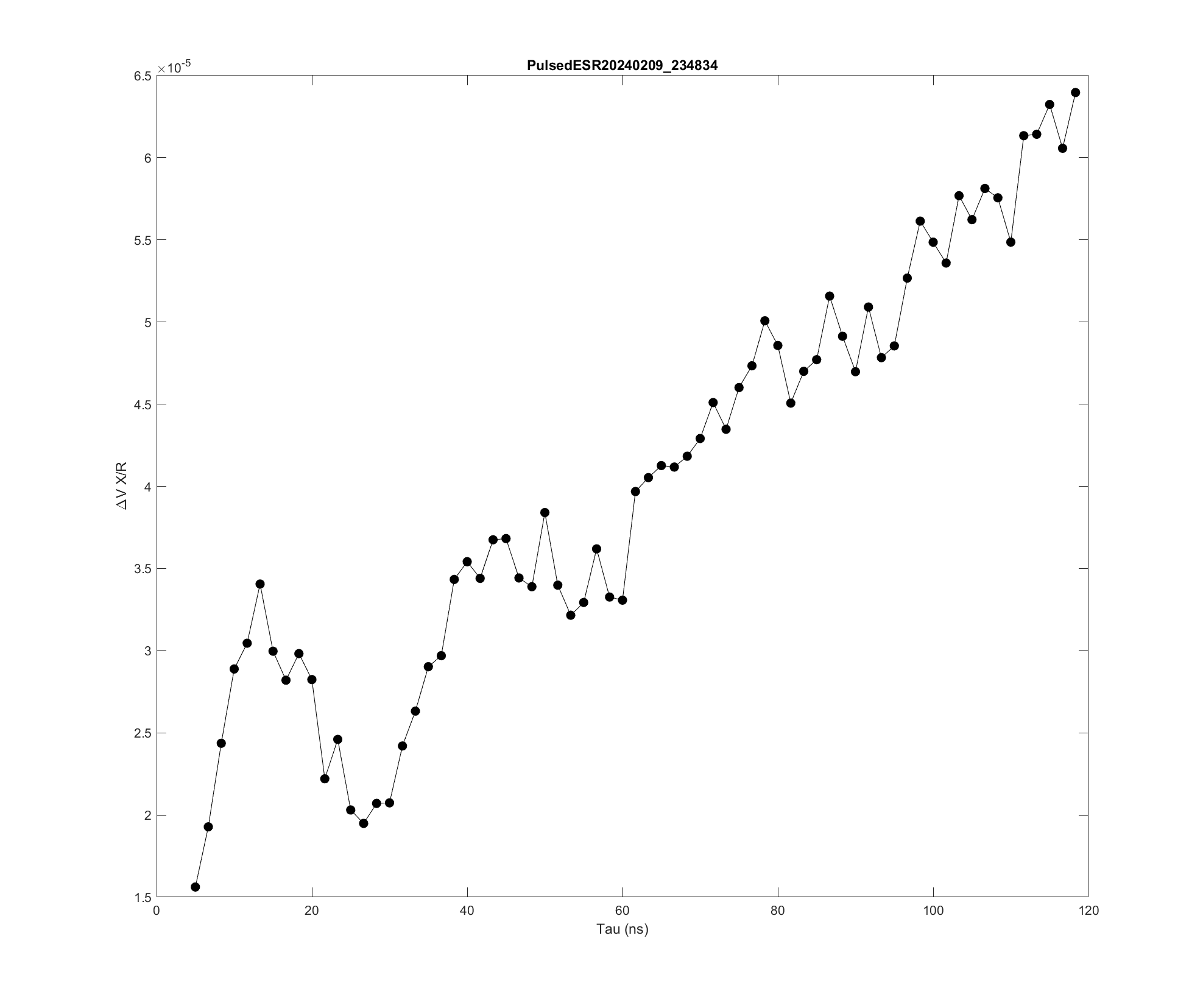

Supplement: Supplementary file 3 — Source Data [file 41467_2025_60409_MOESM3_ESM.zip › SupplementaryData1/Figure3/Fig3c/PulsedESR20240209_234834.png]

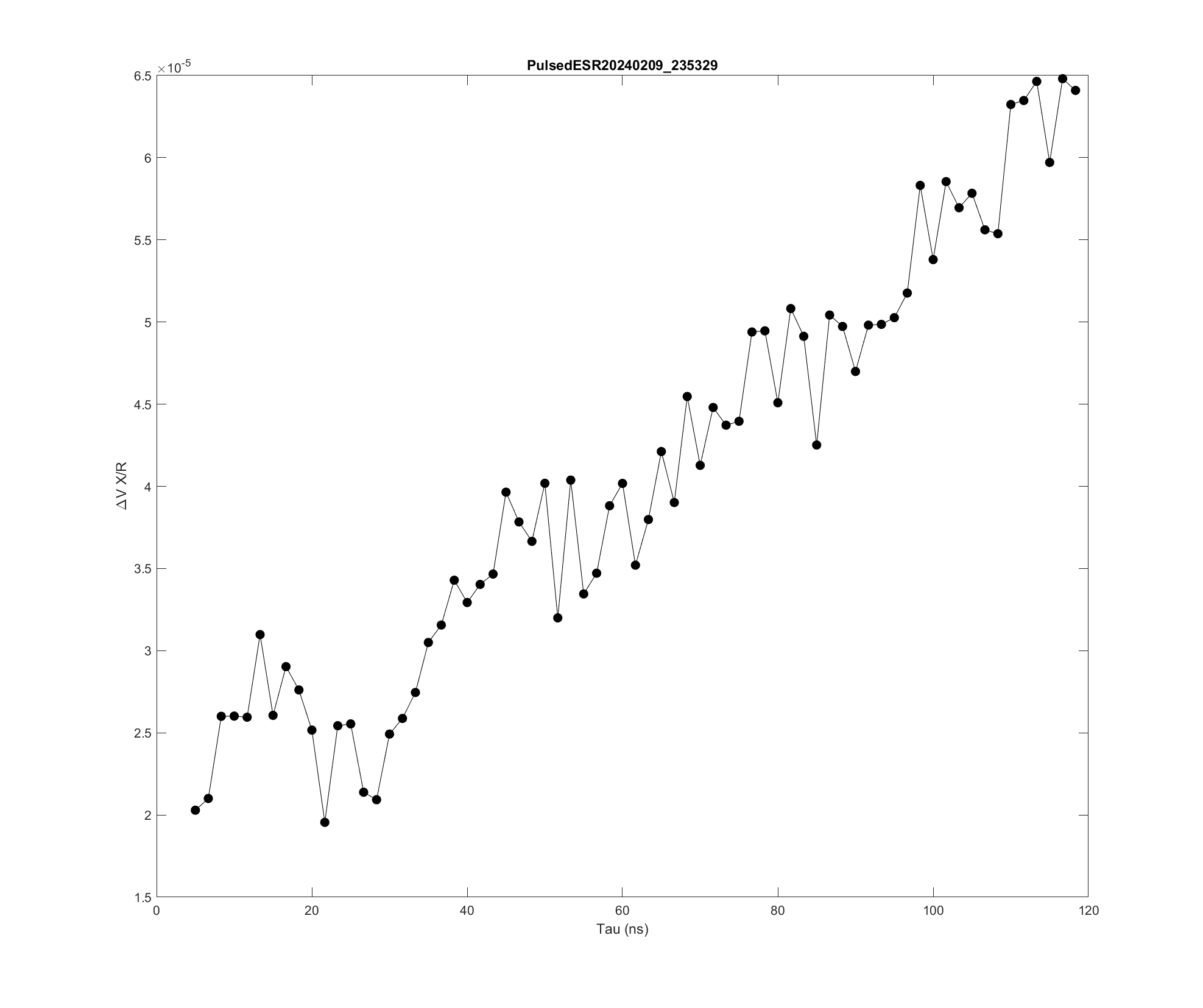

Supplement: Supplementary file 3 — Source Data [file 41467_2025_60409_MOESM3_ESM.zip › SupplementaryData1/Figure3/Fig3c/PulsedESR20240209_235329.png]

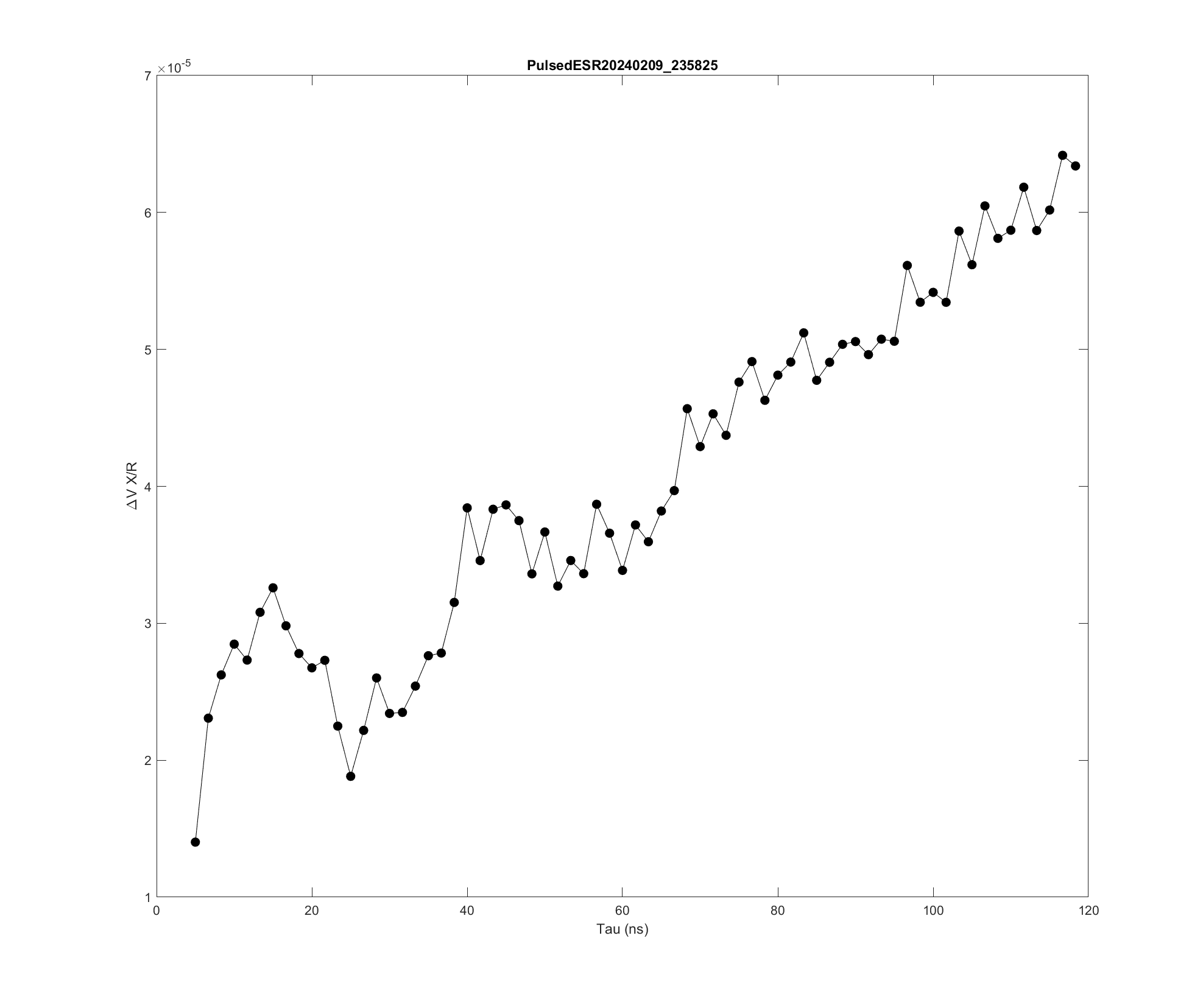

Supplement: Supplementary file 3 — Source Data [file 41467_2025_60409_MOESM3_ESM.zip › SupplementaryData1/Figure3/Fig3c/PulsedESR20240209_235825.png]
